# Valence-delocalized trithorium nanocluster superatoms with open-shell exalted diamagnetism

**DOI:** 10.1038/s41557-025-01790-3

**Published:** 2025-04-11

**Authors:** John A. Seed, Xinglan Deng, Josef Tomeček, Adam Brookfield, David Collison, Floriana Tuna, Ashley J. Wooles, George F. S. Whitehead, Nikolas Kaltsoyannis, Stephen T. Liddle

**Affiliations:** 1 Department of Chemistry, The University of Manchester, Manchester, UK; 2 Centre for Radiochemistry Research, The University of Manchester, Manchester, UK; 3 Department of Chemistry and Photon Science Institute, The University of Manchester, Manchester, UK

**Keywords:** Inorganic chemistry, Coordination chemistry

## Abstract

Quantum-confined nanoclusters can be described by the jellium model, which emphasizes closed-shell electron configurations, but an open-shell variation with jellium aromaticity has been proposed. Such clusters are termed superatoms because they behave like an atom, and they exhibit unusual properties. Superatoms feature metal–metal bonding; hence, since their discovery 40 years ago, superatoms have exclusively involved main group or transition metals, with actinides only considered computationally as dopants owing to actinide–actinide bonding being exceedingly rare. Here we report trithorium nanoclusters exhibiting three-centre-one-electron actinide–actinide bonding. Experimental and computational analysis demonstrates Robin–Day Class III 6*d*-orbital valence delocalization in these clusters. These *S* = 1/2 clusters are paramagnetic, but in external applied magnetic fields they exhibit exalted diamagnetism, evidencing actinide open-shell jellium aromaticity superatom character. Exalted diamagnetism is not normally associated with a single unpaired electron, but with a 1*S*^1^ magic number, the valence delocalization enables exalted diamagnetism, which is aromaticity, via superatom ring currents.

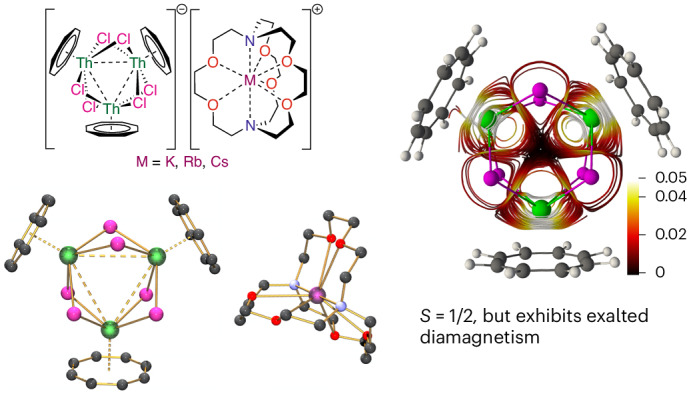

## Abstract (web summary)

Superatoms are metal clusters that collectively behave like an atom, but they usually require metal–metal bonding and thus they are based on main group or transition metals. Now it has been shown that trithorium nanoclusters with delocalized three-centre-one-electron thorium–thorium bonding exhibit exalted diamagnetism. This unveils actinide superatoms that exhibit open-shell jellium aromaticity.

## Main

The properties of metal nanoclusters can deviate substantially from bulk metals owing to quantum confinement effects^1^. While bulk metals are described by band theory, nanoclusters with free-electron metals can be described by the jellium model, where valence electrons in the nanocluster are subject to a uniform potential^2–5^. The jellium model emphasizes closed-shell electron configurations 1*S*^2^1*P*^6^1*D*^10^2*S*^2^1*F*^14^2*P*^6^1*G*^18^2*D*^10^… with electron magic numbers 2, 8, 18, 20, 34, 40, 58, 68… but an open-shell variation with magic numbers 1, 5, 13, 19, 27, 37, 49, 63… and jellium aromaticity has been proposed^6^. Such clusters have been termed superatoms because collectively they behave like an atom, extending the periodic table into a ‘third dimension’^1,5^. Superatoms have demonstrated altered properties, such as increased electron affinity, that is, superhalogens^7,8^, and anomalous magnetic susceptibilities^9^, and since they offer atom-level modifications to engineer superatom properties, they promise numerous opportunities in catalysis, materials and devices^10,11^.

Superatom clusters inherently feature metal–metal bonding, which requires sufficiently diffuse metal valence orbitals. Thus, for the decades that superatoms have been known, they have been based on main group or transition metals^1–5,7–11^. By contrast, although *f* elements, and in particular actinides, have been considered computationally as dopants into superatoms^12–14^, the relatively limited radial distribution of actinide 5*f* valence orbitals has restricted the number of compounds featuring actinide–actinide bonding^15–22^. Notably, endohedral fullerene-encapsulated Th_2_, U_2_ and Th_2_F have been prepared^19,20,22^, but those actinide–actinide bonds cannot be readily translated to synthetic molecular chemistry. Previously, we discovered that reduction of the thorium complex [Th(η^8^-C_8_H_8_)(Cl)_2_(THF)_2_] (**1**)^23^ by the cyclobutadienyl reagent [K_2_{C_4_(SiMe_3_)_4_}] (**2**)^24^ produced the diamagnetic two-electron trithorium nanocluster [{Th(η^8^-C_8_H_8_)(μ_3_-Cl)_2_}_3_{K(THF)_2_}_2_]_∞_ (**3**)^21^, which features three-centre-two-electron Th 6*d*-based metal–metal bonding. Conspicuously, while one-electron U–U and Th–Th bonds have been proposed in endohedral fullerenes^19,22^, experimental spectroscopic and magnetic verification has been lacking. Experimental isolation and confirmation of one-electron actinide–actinide bonding beyond trapped dimetal units would thus be anticipated to afford unusual properties. The isolation of **3** suggested to us that one-electron mixed-valence actinide–actinide nanocluster bonding might be accessible utilizing the 6*d*-orbitals of thorium.

In this Article, we report the preparation and isolation of the crystalline mixed-valence trithorium nanoclusters [M(2.2.2-cryptand)][{(η^8^-C_8_H_8_)Th(μ-Cl)_2_}_3_] (**4M**, M = K, **4K**; Rb, **4Rb**; Cs, **4Cs**) that exhibit three-centre-one-electron actinide–actinide bonding. Experimental and computational analysis demonstrates Robin–Day Class III formalism 6*d*-orbital valence delocalization in these clusters. These clusters are paramagnetic, which should be the dominant physicochemical behaviour, but in external applied magnetic fields, they instead exhibit exalted diamagnetic responses, experimentally evidencing actinide superatom and open-shell jellium aromaticity.

## Results and discussion

### Synthesis

**Fig. 1:**
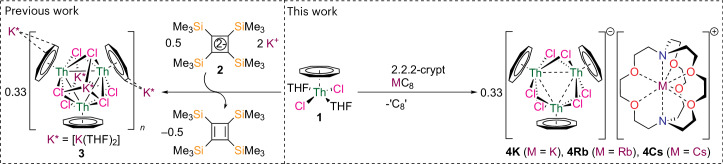
Synthesis of 3 and 4M (M = K, Rb and Cs). In previous work (ref. ^21^), treatment of **1** with **2** afforded **3**, which contains a three-centre-two-electron trithorium bonding interaction. In this work, treatment of **1** and 2.2.2-cryptand with MC_8_ reducing agents affords complexes **4M** (M = K, Rb, Cs). The fate of the excess M and 2.2.2-cryptand component was not determined.

Previously, we reported that reduction of the thorium complex [Th(η^8^-C_8_H_8_)(Cl)_2_(THF)_2_] (**1**)^23^ by the cyclobutadienyl reagent [K_2_{C_4_(SiMe_3_)_4_}] (**2**)^24^ produced the diamagnetic two-electron reduced trithorium nanocluster [{Th(η^8^-C_8_H_8_)(μ_3_-Cl)_2_}_3_{K(THF)_2_}_2_]_∞_ (**3**)^21^, which features three-centre-two-electron Th 6*d*-based metal–metal bonding (Fig. 1). In our report of **3**, its high-yield isolation (89%) resulted from rapid formation and precipitation enabled by the strongly reducing and soluble nature of **2**. Indeed, reduction of **1** to give **3** using the heterogeneous reductant KC_8_ was low yielding (10%). Targeting one-electron reduction, we modulated the slow reaction of **1** with MC_8_ (M = K, Rb, Cs) by addition of 2.2.2-cryptand to increase efficacy without over-reduction. Hence, careful addition of a 1:1 solution of **1** and 2.2.2-cryptand in THF to one equivalent of MC_8_ (M = K, Rb, Cs) in benzene produces, after work-up, analytically pure blue [M(2.2.2-cryptand)][{(η^8^-C_8_H_8_)Th(μ-Cl)_2_}_3_] (**4M**, M = K, **4K**; Rb, **4Rb**; Cs, **4Cs**) **4M** in 10–39% isolated crystalline yields (Fig. 1). Once formed, **4M** are insoluble in hydrocarbon and arene solvents, soluble with limited stability in deuterated tetrahydrofuran, and soluble and stable for several hours in 1,2-dimethoxyethane. Compounds **4M** are thermally sensitive; powdered samples are stable for weeks when stored cold, but when stored at room temperature, they slowly decompose over days, evidenced by the blue colour fading to colourless, while in solution they decompose immediately at temperatures above 35 °C. Compounds of **4M** also readily precipitate from cold tetrahydrofuran and 1,2-dimethoxyethane solutions, limiting the use of variable-temperature NMR studies (Supplementary Figs. 37–44). However, freshly prepared and recrystallized samples of **4M** were amenable to analysis in the solid state and also when dissolved (solution or frozen) in 1,2-dimethoxyethane (see later).

### Solid-state structures

**Fig. 2:**
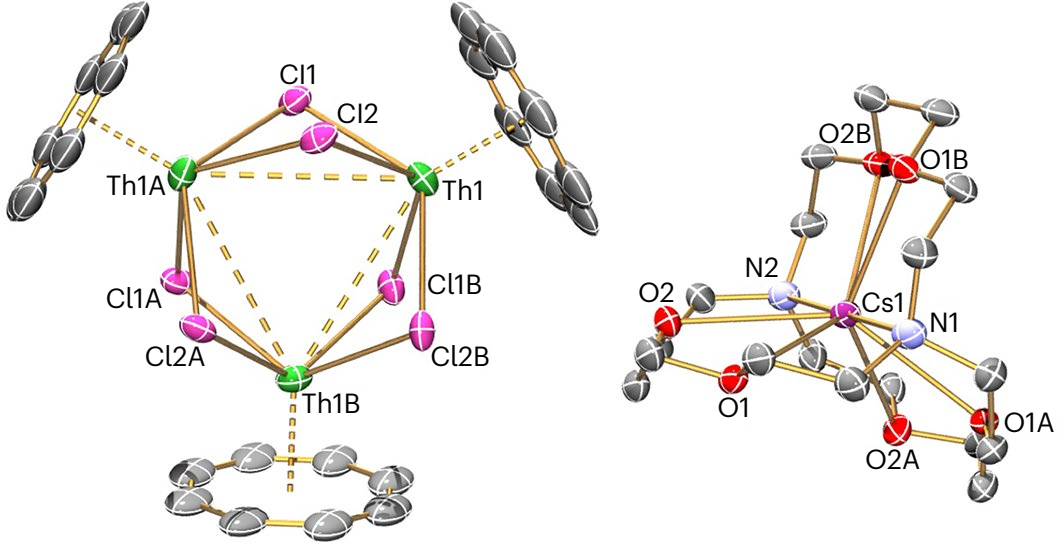
Single-crystal solid-state structure of 4Cs. Single-crystal X-ray diffraction solid-state structure of **4Cs** determined at 150 K. Displacement ellipsoids are rendered at 40% probability and hydrogen atoms are omitted for clarity. The structures of **4K** and **4Rb** are analogous (Supplementary Figs. 1 and 2), but with the Cs^+^ cation replaced by K^+^ or Rb^+^ cations, respectively. Key: thorium, green; chlorine, magenta; cesium, purple; oxygen, red; carbon, grey; nitrogen, blue.

The solid-state structures of **4M** were determined by single-crystal X-ray diffraction (Fig. 2, Supplementary Figs. 1–3 and Supplementary Table 1). Complexes **4M** were also examined by powder X-ray diffraction, but, despite affording satisfactory elemental analyses, unfortunately, the highly air-sensitive nature of these complexes precluded definitive confirmation of phase purities (Supplementary Figs. 5–7 and Supplementary Table 2). The structures of **4M** reveal separated ion pairs with [M(2.2.2-cryptand)]^+^ cation and [{(η^8^-C_8_H_8_)Th(μ-Cl)_2_}_3_]^−^ anion components. Each thorium adopts a four-legged piano-stool geometry, being η^8^-coordinated to a cyclooctatetraenyl ligand and bonded to four bridging chlorides. The three Th–Th distances in **4Cs** (Fig. 2) span the range 4.0463(2)–4.0811(2) Å ((average) av. 4.0689(3) Å), similar to those of **4K** (4.0578(11)–4.0899(11) Å, av. 4.0713(13) Å) and **4Rb** (4.0480(5)–4.0860(5) Å, av. 4.0717(9) Å). This is longer than the sum of the covalent single-bond radii of two thorium atoms of 3.5 Å (ref. ^25^), but well within the corresponding van der Waals distance of 4.74 Å, and longer than the Th–Th distances in **3** (3.9896(4)–3.9947(5) Å, av. 3.991(2) Å)^21^ and endohedral fullerene Th_2_ (3.816(6) Å)^20^ and Th_2_F (3.651(3) Å)^22^. Focusing on **4Cs**, while noting that **4M** are all similar, the cyclooctatetraenyl C–C distances are unremarkable (av. C–C distance of 1.40(2) Å) and, by the 3*σ* criterion, the average Th–C value in **4Cs** (2.719(16) Å) is not statistically different to the average Th–C distances in **3** (2.738(20) Å), **1** (2.734(2) Å) and thorium(III)- and thorium(IV)-cyclooctatetraenyl complexes^23,26,27^. The Th–Cl distances in **4Cs** (av. 2.8459(15) Å) are unexceptional, and the Th–Cl–Th angles span a narrow range 90.63(3)–91.70(3)°, where in each Th(μ-Cl)_2_Th unit, the Cl···Cl distances average 3.484(15) Å, suggesting the absence of any Cl···Cl interactions. The crystallographic data suggest that the sub-valent nature of the thorium ions in **4M** is of 6*d* rather than 5*f* character, since populating *d* orbitals typically produces small (<0.10 Å) changes to metal–ligand distances, whereas *f*-orbital population typically produces ≥0.10 Å changes.

### Electronic structure calculations

To understand the electronic structure of the trithorium components of **4M** and rationalize the characterization data, we carried out quantum chemical calculations on the [{(η^8^-C_8_H_8_)Th(μ-Cl)_2_}_3_]^–^ anion component of **4M** (**4**′) in its doublet ground state (Supplementary Tables 13 and 14). A domain-based local-pair natural orbital coupled cluster calculation yielded a T_1_ diagnostic value of ~0.0087, far below the threshold above which multi-configurational effects are considered relevant (~0.05 for open-shell systems). Hence, **4**′ has predominant single-reference character, and density functional theory (DFT) is thus an appropriate method with which to probe its electronic structure. PBE0 all-electron SARC geometry optimized **4**′ has no imaginary frequencies, and exhibits average Th–Th distances of 4.09 Å, close to the experimental average (4.07 Å). The computed properties of **4**′ reproduce the experimental magnetic, Raman, ultraviolet/visible/near infrared (UV/vis/NIR) and electron paramagnetic resonance (EPR) spectroscopic data (see later), providing experimental verification of the electronic structure of **4**′. Furthermore, an energy decomposition analysis of **4Cs** (Supplementary Fig. 123 and Supplementary Table 16) reveals, as expected, that the interaction energy of the [{(η^8^-C_8_H_8_)Th(μ-Cl)_2_}_3_]^−^ and [Cs(2.2.2-cryptand)]^+^ fragments is dominated by electrostatic interactions, which constitute 72% of the total attractive energy contributions (Supplementary Text).

**Fig. 3:**
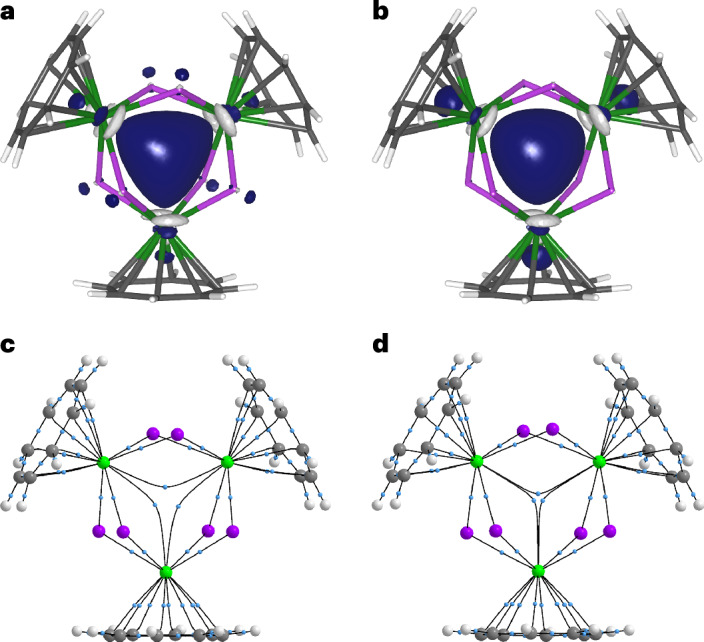
Comparison of DFT molecular orbital and quantum theory of atoms-in-molecules molecular graphs of 4′ and 3′. **a**, The highest, singly occupied molecular orbital of the [{(η^8^-C_8_H_8_)Th(μ-Cl)_2_}_3_]^–^ anion component of **4M** (**4**′). **b**, The highest, doubly occupied molecular orbital of the model [{Th(η^8^-C_8_H_8_)(μ_3_-Cl)_2_}_3_(K)_2_] of **3** (**3**′), with potassium cations omitted for clarity. **c**, Molecular graph of **4**′. **d**, Molecular graph of **3**′. The molecular orbitals are plotted at the 0.05 isosurface level, and the molecular graphs are plotted with critical point electron density thresholds of 0.01 a.u. showing bond paths (black lines) and bond critical points (blue dots). Key: thorium, green; chlorine, magenta; carbon, grey; hydrogen, white.

The DFT α-spin highest occupied molecular orbital (HOMO) of **4**′ is a three-centre-one-electron valence-delocalized singly occupied molecular orbital (SOMO; Fig. 3a), which resembles the doubly occupied HOMO of [{Th(η^8^-C_8_H_8_)(μ_3_-Cl)_2_}_3_(K)_2_] (**3**′) (Fig. 3b)^21^. Mulliken population analysis of the SOMO of **4**′ reveals it to be of 86% Th and 9% Cl character. The Th component is 62/14/7% 6*d*/5*f*/7*p* character, which is similar to the corresponding HOMO of **3**′.

The quantum theory of atoms-in-molecules (QTAIM) analysis of **4**′ (Fig. 3c) finds Th–Th bond paths that are less curved than in **3**′ (Fig. 3d). The bond path curvature reflects the accumulation of electron density in the centre of the trithorium triangle and hence the number of electrons in the SOMO of **4**′ and HOMO of **3**′. The bond critical point (BCP) properties also reflect the differences between **4**′ and **3**′. For **4**′, the electron density (*ρ*) at the Th–Th BCPs is 0.014 a.u. and the Laplacian (${{\nabla }^{2}\rho }_{{\rm{BCP}}}$) is –7 × 10^–3^ a.u. (0.022 a.u. and –9 × 10^–3^ a.u. in **3**′, respectively). The small value of ${{\nabla }^{2}\rho }_{{\rm{BCP}}}$ indicates a flat electron density distribution. Reduced Th–Th bonding in **4**′ is also reflected in the QTAIM delocalization index, which is 0.12 (0.26 in **3**′) and the Th–Th Mayer bond order (MBO) of 0.27 (0.45 in **3**′). The QTAIM and MBO data consistently find Th–Th bond properties in one-electron **4**′ that are approximately half those of two-electron **3**′, and this bonding picture is reinforced by adaptive natural density partitioning, electron density of delocalized bonds, multicentre indices, electron localization function, iso-chemical shielding surface, and Foster–Boys and Pipek–Mezey localization schemes (Supplementary Materials and Methods and Supplementary Figs. 115, 116, 119 and 120).

### Spectroscopic characterization

**Fig. 4:**
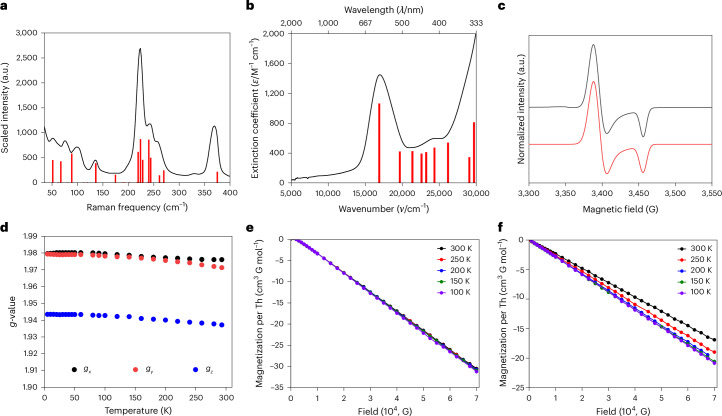
Spectroscopic and magnetic data for 4Cs and 5. **a**, Selected portion of the Raman spectrum (black line) of **4Cs** with DFT computed inelastic scattering bands (red sticks). **b**, UV/vis/NIR spectrum (black line) of **4Cs** with TD-DFT computed absorptions (red sticks). **c**, Powder EPR spectrum of **4Cs** at 50 K (black line) with simulation (red line (1 Tesla = 10,000 Gauss)). **d**, Variable-temperature (5–293 K) powder EPR spectra data for **4Cs** showing the minor variance of *g*-values over the temperature range. **e**, Isothermal magnetization versus field data per Th for **4Cs** at 100 K, 150 K, 200 K, 250 K and 300 K from 0–7 Tesla showing the uniform diamagnetic response. **f**, Isothermal magnetization versus field data per Th for **5** at 100 K, 150 K, 200 K, 250 K and 300 K from 0–7 Tesla showing the uniform normal diamagnetic response. Error bars for **e** and **f** are not shown for clarity (see Supplementary Figs. 106 and 110–113 for further details). Source data

The Raman spectra of **4M** (Fig. 4a, Supplementary Figs. 67–81 and Supplementary Table 3) exhibit inelastic scattering bands at ~53 cm^−1^, ~75 cm^−1^, ~99 cm^−1^ and ~135 cm^−1^ that involve the trithorium cores and correspond, using *D*_3*h*_ symmetry labels, to two doubly degenerate bending modes (${E}^{{\prime} }$), a symmetric breathing mode (${{\rm{A}}}_{1}^{{\prime} }$) and an antisymmetric breathing mode (${{\rm{A}}}_{1}^{{\prime} {\prime} }$, Cl atoms moving the opposite direction to the rest of the molecule). For **4**′, the corresponding calculated frequencies of 51.4/51.5 cm^–1^, 67.3/67.5 cm^–1^, 88.7 cm^–1^ and 136.0 cm^–1^ (Supplementary Fig. 117) compare well with the experimental values (Fig. 4a).

The UV/vis/NIR spectra of **4M** in DME all exhibit two broad absorptions before the onset of strong charge transfer bands that extend into the UV region (Supplementary Figs. 85–93 and Supplementary Table 4). For example, for **4Cs** (Fig. 4b), absorptions at 16,950 cm^−1^ (590 nm, *ε* = 1,450 M^−1^ cm^−1^) and 24,390 cm^−1^ (410 nm, *ε* = 596 M^−1^ cm^−1^) are observed. These features are not present in the UV/vis/NIR spectrum of **1** and are absent when samples of **4M** are re-recorded after exposure to air, suggesting that they are associated with the Th–Th bonding in **4M**. Indeed, PBE0 TD-DFT calculations on **4**′ suggest that these absorptions correspond to transitions from the 6*d*-based SOMO to vacant thorium orbitals of predominantly 5*f* and 7*s* character (Supplementary Fig. 118 and Supplementary Table 10). Focusing on the ~590 nm feature, for **4M**, the average inter-valence charge transfer *Γ*_av._ parameter (*Γ* = 1 − (Δ*ν*_1/2_/Δ*ν*_1/2°_)) is 0.64, consistent with a Robin–Day Class III formalism^28,29^ and full valence delocalization as suggested by the DFT calculations. The *Γ*_av._ value for **4M** compares to values of 0.26–0.29 (Class II) for [{Ln(C_5_Pr^i^_5_)(μ-H)}_3_(μ_3_-I)_2_] (Ln = Y, Gd)^30^, which are reminiscent of **3** and **4M**, suggesting more extensive delocalization in 6*d* than 5*d* trimetallo clusters.

Variable-temperature X-band EPR spectra of powdered and frozen solution **4M** are all similar (Supplementary Figs. 94–102 and Supplementary Tables 5–7). Focusing on powdered **4Cs**, near to axial spectra are found (Fig. 4c) with two major features of *g*_*x*_ = *g*_*y*_ (*g*_⊥_) = 1.98 and *g*_*z*_ (*g*_||_) = 1.94. There is some temperature-dependent variance of signal strength and exact *g*-value, with a slight increase in values as the temperature is lowered, but the spectra consistently have *g*_⊥_ > *g*_||_ (Fig. 4d). DFT EPR calculations on **4**′ predict *g*_*x*_ = 1.955, *g*_*y*_ = 1.958 (*g*_⊥_ = 1.957) and *g*_*z*_ (*g*_||_) = 1.911, in good agreement with experiment and correctly returning *g*_⊥_ > *g*_||_. All the experimental and computed *g*-values correspond to effective magnetic moments of 1.7 μ_B_ for **4M** consistent with the theoretical value of 1.73 μ_B_ for an *S* = 1/2 *d*^1^ ion (assuming *g* = 2).

The EPR spectra of **4M** exhibit *g*_⊥_ > *g*_||_, whereas in mono-thorium(III) EPR spectra^31–39^, the *g*-values are similar but the ordering is reversed, that is, *g*_||_ > *g*_⊥_. In mono-thorium(III) compounds, the unpaired electrons reside in $d_{z^2}$-type MOs that are parallel to their three- or fourfold principal rotation axes, whereas in **4M**, the electron resides in an MO that results from constructive overlap of three $d_{z^2}$ orbitals that are perpendicular to the threefold principal rotation axis of **4**′. That the *g*-values of **4M** have an opposite ordering to monometallic thorium(III) complexes supports the valence-delocalized description of **4M**. If the unpaired electron in **4M** resided on the C_8_H_8_ ligands, then a complex feature with multiple hyperfine components centred at *g* = 2 would be observed^40,41^. Furthermore, if the unpaired electron were of 5*f* character, its *g*-value would be very different and not observable over the higher temperature range owing to rapid relaxation^26^.

The small increases in *g*-values as the temperature is reduced provide additional insight into the Th–Th bonding in **4M**. Contraction of metal–ligand interatomic distances on cooling should increase the ‘crystal field’, thereby rendering the 1/Δ*E* term smaller and hence the shift in *g*-values from *g*_e_ should decrease, as observed experimentally. In the MO treatment of trimetallo clusters, the anionic ligands define the first-order interactions and the metal–metal bonds are a second-order effect^42^. Cooling will therefore primarily shorten and strengthen the Th–COT and Th–Cl bonds. However, the *g*-values are a direct reporter of excited states that are mixed into the SOMO by spin–orbit coupling under the influence of an applied magnetic field, and the SOMO is Th–Th bonding character and largely ‘blind’ to the Th–COT and Th–Cl interactions. Thus, the EPR data are consistent with the presence of Th–Th bonding, even as a second-order interaction, although of course it should be acknowledged that many factors can influence *g-*values.

### Magnetic characterization

Although EPR spectroscopy demonstrates the *S* = 1/2 paramagnetic nature of **4M**, in a 1 Tesla (1 Tesla = 10,000 Gauss) external field, super-conducting quantum interference device magnetometry reveals no net first-order Zeeman paramagnetic susceptibilities and no second-order Zeeman temperature-independent paramagnetism (Supplementary Figs. 103–107). Samples were scrupulously weighed (Supplementary Table 8), and **4Cs** was measured as a powder and eicosane-immobilized powder revealing agreement to within 5.4% ruling out any influence of the eicosane (Supplementary Fig. 108). Analysis of the magnetometric data revealed average standard uncertainties of ~5% in magnetization measurements, which is essentially the error of the magnetometer (Supplementary Figs. 110–113). Isothermal magnetization (*M*) versus field (*H*) data (100–300 K) for **4M** over the range 0–7 Tesla (Fig. 4e) reveal negative linear slopes (*R*^2^ = 0.9997–0.9999) with net molar diamagnetic susceptibilities of −1,400 × 10^−6^ cm^3^ mol^−1^ to −1,800 × 10^−6^ cm^3^ mol^−1^ per complex, which is up to four times the anomalous diamagnetic susceptibilities exhibited by [Pt_13_H_*n*_] (*n* = 18, 38) superatoms^9^, and can be compared with predicted standard Pascal’s constant diamagnetic correction values^43^ for **4M** of −692 × 10^−6^ cm^3^ mol^−1^ to −712 × 10^−6^ cm^3^ mol^−1^. The magnetization values (300 K) per thorium atom of −30.5 cm^3^ Gauss mol^−1^ to −42.1 cm^3^ Gauss mol^−1^ at 7 Tesla (Fig. 4e) are similar to the magnetization per platinum atom for [Pt_13_H_*n*_] superatoms^9^. The residual diamagnetism after removal of non-thorium atom contributions for **4M** is 11–17 times that expected per thorium ion and overall the diamagnetic responses of **4M** are increased 97–160% compared with expected values (Supplementary Table 9). By contrast, *M* versus *H* data for **1** (300 K) are within 1.6% of the expected value from Pascal’s constants (−245 × 10^−6^ cm^3^ mol^−1^ versus −249 × 10^−6^ cm^3^ mol^−1^, respectively) with a magnetization per thorium atom of only −16.2 cm^3^ Gauss mol^−1^ at 7 Tesla (Supplementary Fig. 109). We examined the *M* versus *H* data for the trithorium(IV)-oxo cluster [{Th(η^8^-C_8_H_8_)(μ-Cl)_2_(μ_3_-O)}_3_(Cs)_2_(DME)_3_] (**5**) (Fig. 4f and Supplementary Fig. 114), a fully characterized decomposition product isolated during our preliminary attempts to obtain **4M** (Supplementary Materials and Methods and Supplementary Figs. 4, 34–36, 64, 82–84 and 92). At 300 K, we find a net molar diamagnetic susceptibility of −731 × 10^−6^ cm^3^ mol^−1^ (*R*^2^ = 0.9998) for **5** that is within 4.8% of the value predicted from Pascal’s constants (−697 × 10^−6^ cm^3^ mol^−1^) and a magnetization per thorium atom of only −16.9 cm^3^ Gauss mol^−1^ at 7 Tesla, thus confirming that the anomalous diamagnetism of **4M** does not just result from having three thorium ions in close proximity.

### Ring current and magnetism calculations

**Fig. 5:**
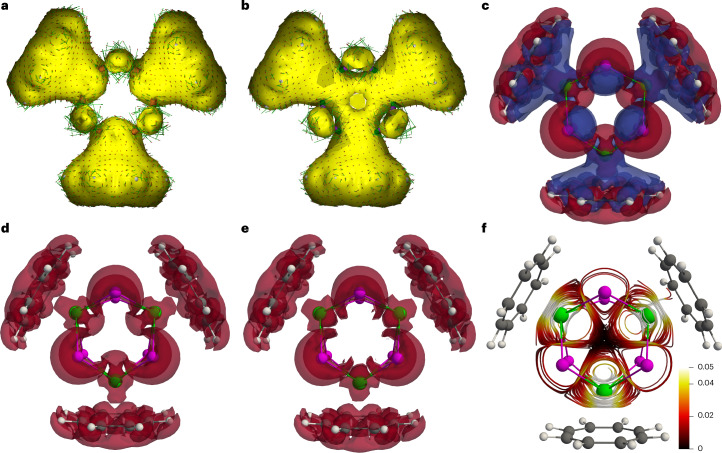
Computed AICD vectors for 4′ and 3′ and GIMIC of 4′ and 6. **a**, AICD plot for **4**′. **b**, AICD plot for **3**′. **c**, GIMIC plot of **4**′ showing diatropic (red) and paratropic (blue) components. **d**, GIMIC plot of **4**′ showing only the diatropic (red) components. **e**, GIMIC plot of **6** showing only the diatropic (red) components. **f**, Induced current density streamlines for **4**′. The AICD plots are rendered at the 0.015 a.u. isosurface level and the GIMIC representations are plotted with modulus of *j*(r) isovalues of 0.025 a.u. for diatropic (red) and paratropic (blue) regions. The streamlines vector magnitude colour scale bar is in a.u.

Plotting the anisotropy of induced current density (AICD) of **4**′ at the same isovalue as that of **3**′ (0.015 a.u.) reveals essentially no induced current density vectors within the trithorium core of **4**′ (Fig. 5a), whereas this is present for **3**′ (Fig. 5b), reflecting their one- and two-electron natures, respectively. However, the AICD plot of **4**′ reveals that ring currents are localized around the Th_3_Cl_6_ core, with additional C_8_H_8_ contributions, and that the current vectors for the three thorium ions operate in concert. Gauge-including magnetically induced current (GIMIC) calculations return current densities (*j*(r)) for **4**′ in agreement with the AICD calculations. GIMIC finds that **4**′ sustains a net diatropic current of 1.81 nA T^−1^, which is 1.92 nA T^−1^ more diatropic than the current of −0.11 nA T^−1^ computed for the *S* = 0 neutral cluster [{(η^8^-C_8_H_8_)Th(μ-Cl)_2_}_3_] (**6**, using the same coordinates as **4**′) (Supplementary Figs. 121 and 122 and Supplementary Tables 11 and 12).

The *j*(r) of **4**′ produce a complex interplay of paratropic and diatropic currents across topologically diverse **4**′. The overall *j*(r) topology of the Th_3_Cl_6_ unit is diatropic, and that is bounded by a paratropic region and then the outer sides of the C_8_H_8_ rings provide an additional layer of diatropism (Fig. 5c). However, probing local *j*(r) effects is deterministic, being conditional on integration box selection. Therefore, we computed the net isotropic magnetic susceptibility of **4Cs**. While the computed value of −729.59 × 10^−6^ cm^3^ mol^−1^ for **4Cs** is approximately half the experimental value of **4Cs**, given that these magnetic susceptibility calculations are limited to scalar (spin–orbit neglected) relativistic effective core potentials and are computed as the derivative of the energy in the limit of zero magnetic field, that the calculation returns a substantially negative (diamagnetic), rather than expected positive (paramagnetic), susceptibility for an *S* = 1/2 molecule is striking, especially when considering that diamagnetism is normally weak and paramagnetism is normally a dominant effect. For comparison, the analogous value for hypothetical diamagnetic *S* = 0 [M(2.2.2-cryptand)]^+^[**6**] of −557.65 × 10^−6^ cm^3^ mol^−1^ is less diamagnetic than the value computed for *S* = 1/2 **4Cs**, emphasizing the otherwise counterintuitive increase in diamagnetic response when an electron is added to the Th_3_Cl_6_ core.

Thus, the experimental and computed data present a consistent picture. The ground state of **4**′ exhibits a valence-delocalized three-centre-one-electron SOMO, which results from the constructive overlap of three $6d_{z^2}$-based orbitals to produce a HOMO that has the appearance of an *s* orbital. When **4**′ is placed in an external applied magnetic field, the SOMO ground state is polarized from the centre of **4**′ across the Th_3_Cl_6_ unit, generating concerted ring currents over the latter. In turn, that produces exalted diamagnetism^44,45^ (Fig. 5d), whose delocalized continuum is disrupted at the thorium-chloride intersections when the valence-delocalized electron is absent in hypothetical *S* = 0 **6** (Fig. 5e), consistent with the reduction of diamagnetic susceptibility when the unpaired electron is removed from **4**′ in silico. While a single unpaired electron would be expected to align parallel to the external applied field, the global ring current around the Th_3_Cl_6_ core evidently results in substantial antiparallel alignment^9^. Thus, **4**′ is diatropic despite its *S* = 1/2 nature, consistent with the magnitude of exalted diamagnetism further supported by the current streamlines (Fig. 5f). To put the exalted diamagnetism (*Λ*; Supplementary Table 9) of **4M** into context^44^, their *Λ* values (not including the in-principle ‘cancelled’ *χ*_P_ contribution of 1,250 × 10^−6^ cm^3^ mol^−1^) are 688 × 10^−6^ cm^3^ mol^−1^ to 1,108 × 10^−6^ cm^3^ mol^−1^, which compares to values of −4 × 10^−6^ cm^3^ mol^−1^, 0 cm^3^ mol^−1^, 13.7 × 10^−6^ cm^3^ mol^−1^, 34 × 10^−6^ cm^3^ mol^−1^, 267.4 × 10^−6^ cm^3^ mol^−1^ and 640.1 × 10^−6^ cm^3^ mol^−1^ for **1** (Supplementary Fig. 109), cyclohexane^46^, benzene^46^, **5** (Supplementary Fig. 114), [14]annulene^46^ and [18]annulene^46^, respectively. Exalted diamagnetism is normally a hallmark of closed-shell jellium superatoms^9,47–49^. However, it has also been predicted for open-shell superatoms as jellium aromaticity^6^, where the aromaticity results from the highest superatomic shell being half-filled with same-spin electrons that presents extra stability; this differs from spherical aromaticity^50^ since it results from delocalization of electrons inside metal clusters, whereas the latter results from delocalization of electrons on the surface of a sphere. The characterization data support the molecular thorium valence-delocalized description of **4M**, where the large radial distribution of 6*d*-orbitals evidently enables valence delocalization and superatom character in an external applied field, in contrast to transition metal superatoms that tend to delocalize using *s* orbitals because their radially less expansive *d* orbitals remain localized, arguing against spherical aromaticity, metallic character, conductance and Knight shift effects. The superatom contributions to the exalted diamagnetic responses of **4M** correspond to ~50–62% of their anomalous diamagnetism and being *S* = 1/2, **4M** has a 1*S*^1^ magic number^6^, which is consistent with the SOMO of **4**′ having the overall appearance of an *s* orbital.

## Conclusion

We have reported the synthesis and characterization of three mixed-valence trithorium nanoclusters, which each feature a singly occupied three-centre-one-electron valence-delocalized trithorium metal–metal bond. These *S* = 1/2 clusters are paramagnetic, but in external applied magnetic fields, they exhibit diamagnetic susceptibilities owing to exalted diamagnetism overwhelming the intrinsic paramagnetic character, which experimentally evidences open-shell jellium aromatic character. Exalted diamagnetism, which is an experimental observable resulting from aromaticity, is not normally associated with a single unpaired electron, but here with a magic number of 1*S*^1^, the valence delocalization enables exalted diamagnetism by the global superatom ring currents in these trithorium clusters. The discovery of actinide open-shell jellium superatoms allows us to classify these one-electron trithorium clusters as superalkali, or even supercoinage, atoms, where for almost a century Group 1 and 11 elements have also been known to exhibit anomalously strong diamagnetism^51^. This suggests that *f*-element metal–metal bonding through the superatom concept shares underpinning analogies to other element groups, and is thus poised for further elaboration in the periodic table’s ‘third dimension’.

## Methods

### General

Experiments were carried out under a dry, oxygen-free dinitrogen atmosphere using Schlenk-line and glove-box techniques. All solvents and reagents were rigorously dried and deoxygenated before use. Compounds were variously experimentally characterized by single-crystal and powder X-ray diffraction, NMR, FTIR, Raman, UV/vis/NIR and EPR spectroscopies, SQUID magnetometry and elemental analysis. Compounds were theoretically probed using CCSD, DFT, TD-DFT, ELF, QTAIM, AdNDP, EDDB_F_, NBO7, AICD, ICSS, GIMIC and EDA calculations. Further details are available in Supplementary Materials and Methods.

## Online content

Any methods, additional references, Nature Portfolio reporting summaries, source data, extended data, supplementary information, acknowledgements, peer review information; details of author contributions and competing interests; and statements of data and code availability are available at 10.1038/s41557-025-01790-3.

## Supplementary information

Supplementary Information Supplementary Materials and Methods (general experimental details, preparations and computational details), Supplementary Text (additional discussional points), Figs. 1–123, Tables 1–16 and References 1–80.

Supplementary Data 1 Crystallographic data for **4K**; CCDC number 2374489.

Supplementary Data 2 Crystallographic data for **4Rb**; CCDC number 2374490.

Supplementary Data 3 Crystallographic data for **4Cs**; CCDC number 2374491.

Supplementary Data 4 Crystallographic data for **5**; CCDC number 2376515.

## Source data

Source Data Fig. 4 Source data for Fig. 4a–f.

## Data Availability

The details of the experimental methods, characterization techniques, and computational studies and associated references are available in the Supplementary Information. Source data are available from the authors on reasonable request. Crystallographic data for the structures reported in this Article have been deposited at the Cambridge Crystallographic Data Centre, under depositions numbers CCDC 2374489 (**4K**), 2374490 (**4Rb**), 2374491 (**4Cs**) and 2376515 (**5**). Copies of the data can be obtained free of charge via https://www.ccdc.cam.ac.uk/structures/. Source data are provided with this paper.
