# Supplementary material for: Valence-delocalized trithorium nanocluster superatoms with open-shell exalted diamagnetism
Source: Nat Chem. 2025 Apr 11;17(7):1035–41. doi: 10.1038/s41557-025-01790-3 (PMC12226342; doi:10.1038/s41557-025-01790-3)
Supplement: Supplementary file 1 — Supplementary Materials and Methods (general experimental details, preparations and computational details), Supplementary Text (additional discussional points), Figs. 1–123, Tables 1–16 and References 1–80. [file 41557_2025_1790_MOESM1_ESM.pdf]

nature chemistry

Supplementary information https://doi.org/10.1038/s41557-025-01790-3

Valence-delocalized trithorium nanocluster superatoms with open-shell exalted diamagnetism

In the format provided by the authors and unedited

Table of Contents

Supplementary Materials and Methods……………………………………………………………1
Supplementary Text…………………………………………………………………………….8
Supplementary Figures………………………………………………………………………...10
Supplementary Tables…………………………………………………………………………71
Supplementary References…………………………………………………………………….80

Supplementary Materials and Methods

General Experimental Details

All manipulations were carried out using Schlenk techniques, or an MBraun UniLab glovebox, under an atmosphere of dry dinitrogen. Solvents were dried by passage through activated alumina towers and degassed before use. All solvents were stored over potassium mirrors except for ethers, which were stored over activated 4 Å sieves. Deuterated solvents were distilled from potassium, degassed by three freeze-pump-thaw cycles and stored under dinitrogen. Glassware was silylated before use. The compounds $ThCl_4(THF)_{3.5}$,[1] MC_8 (M = K, Rb, Cs),[2] $C_8H_8K_2$,[3] [Th(η^8-$C_8H_8)_2$],[4] and [Th(η^8-C_8H_8)$(Cl)_2(THF)_2$] (**1**)[5] were prepared by the modified procedures described below. 2.2.2-cryptand was purchased from Sigma Aldrich and dried under dynamic vacuum (1×10^{-3} mbar) at 50 °C for 24 hours prior to use.

Crystals were examined using a Rigaku XtalLAB Synergy-S diffractometer, equipped with a HyPix 6000HE photon counting pixel array detector with mirror-monochromated CuKα (λ = 1.5418 Å) or MoKα (λ = 0.71073 Å) radiation. Intensities were integrated from a sphere of data recorded on narrow (0.5°) frames by ω rotation. Cell parameters were refined from the observed positions of all strong reflections in each data set. Gaussian grid face-indexed absorption corrections with a beam profile correction were applied. The structures were solved by direct methods using SHELXT,[6] and all non-hydrogen atoms were refined by full-matrix least-squares on all unique F^2 values with anisotropic displacement parameters with exceptions noted in the respective cif files. Except where noted, Hydrogen atoms were refined with constrained geometries and riding thermal parameters. CrysAlisPro[7] was used for control and integration, and SHELXL[8] and Olex2[9] were employed for structure refinement. ORTEP-3[10] and POV-Ray[11] were employed for molecular graphics. Crystallographic details for **4M** and **5** are shown in Supplementary Table 1.

Powder X-ray diffraction (PXRD) data of microcrystalline samples of **4M** were mounted with a minimum amount of Fomblin® onto a 0.1 mm glass fibre and collected at 100 K using a Rigaku FR-X rotating anode single crystal X-ray diffractometer using Cu Kα radiation (λ = 1.5418 Å) with a Hypix-6000HE detector and an Oxford Cryosystems nitrogen flow gas system. Data were collected between 3–50 °θ, with a detector distance of 150 mm and a beam divergence of 1.0 mRad.[12] X-ray data were collected using CrysAlisPro.[7] The instrument was calibrated using the collected data, with the instrument model refined using diffraction peak positions measured at multiple detector angles. The data were reduced and integrated using CrysAlisPro.[7] Le Bail profile analysis was performed using JANA2020.[13-15] Initial unit cell parameters were taken from the corresponding single crystal data. Comparison of unit cell values obtained from PXRD Le Bail refinement results for **4M** with those expected from SCXRD data are shown in Supplementary Table 2.

^1H and $^{13}C\{^1H\}$ spectra were recorded on a Bruker 400 spectrometer operating at 400.1 and 125.8 MHz, respectively or on a JEOL JNM-ECZ 400 MHz spectrometer operating at 399.78 and 100.52 MHz, respectively; chemical shifts are quoted in ppm and are relative to tetramethylsilane (^1H, ^{13}C). Samples were prepared in the glovebox and placed in J. Young PTFE 5mm screw-topped borosilicate NMR tubes.

FTIR spectra were recorded on a Bruker Alpha spectrometer with a Pt-ATR module in a glovebox.

Raman spectra were recorded on a Horiba XploRA Plus Raman microscope using either a 785, 638, or 532 nm laser with a power of 1.5 mW. The number of scans and acquisition time were varied for each complex to inhibit sample decomposition. The power was adjusted using a power filter for each complex to inhibit sample decomposition. Experimental parameters are in Supplementary Table 3.

UV/Vis/NIR spectra were recorded on a Perkin Elmer LAMBDA 1050 spectrometer where data were collected in 1cm path length cuvettes and were run versus the appropriate reference solvent. Selected UV/Vis/NIR experimental information is detailed in Supplementary Table 4.

Continuous-wave (c.w.) EPR spectra were measured using an X-band (9.4 GHz) Bruker EMX Plus spectrometer under 100 kHz magnetic field modulation and 2 G modulation amplitude at 293-5 K. Powdered samples were loaded into 3.9 mm (OD) quartz tubes in a glovebox under dinitrogen, and the tubes were flame sealed under vacuum using a MAPP-oxygen torch prior to experiments. Field corrections were applied to the raw data using Bruker strong pitch (g = 2.0028) as a reference. Spectra were simulated using the Easyspin 6.0 Software package.[16]

Variable-temperature magnetic moment data were recorded with various applied direct current (DC) external fields on a Quantum Design MPMS3 superconducting quantum interference device magnetometer using doubly recrystallized powdered samples. Measurements were performed in dc scan mode using 40 mm scan length and 6 s scan time. Samples were checked for purity and data reproducibility between several independently prepared batches for each compound examined where great care was taken to ensure accurate weighing of materials. Samples were crushed with a mortar and pestle under an argon atmosphere and immobilized in an eicosane matrix within a borosilicate glass NMR tube to prevent sample reorientation during measurements. The tube was flame-sealed under dynamic vacuum (1×10^{-3} mbar) to a length of approximately 3 cm and mounted in the centre of a drinking straw, with the straw fixed to the end of an MPMS3 sample rod. Care was taken to ensure complete thermalization of the sample before each data point was measured by employing delays at each temperature point as well as a slow cooling rate (2.5 K/min from 300 to 100 K; 1 K/min from 100 to 55 K; 0.5 K/min from 55 to 1.8 K). The sample was held at 1.8 K for 120 minutes before isothermal magnetization measurements to account for slow thermal equilibration of the sample. Diamagnetic corrections were applied using tabulated Pascal constants[17] and measurements were corrected for the effect of the blank sample holders (flame sealed Wilmad NMR tube and straw) and eicosane matrix. Variable temperature magnetic susceptibility and isothermal magnetization measurements were conducted on calibrated blanks (sealed NMR tubes containing varying masses of eicosane) to determine the eicosane contribution, which is defined per mg of eicosane used, in addition to the contribution from the NMR tube/straw, which is kept approximately constant from sample to sample. To rule out anomalous eicosane effects, we collected magnetometric data on **4Cs** without eicosane and **4Cs** with eicosane (**4Cs-eico**) and find agreement within 5.4% which is within the measurement error of the instrument. Sample information is detailed in Supplementary Table 9.

Elemental microanalyses were carried out by Mr Martin Jennings and Mrs Anne Davies at the Micro Analytical Laboratory, Department of Chemistry, University of Manchester.

Modified Synthesis of $ThCl_4(THF)_{3.5}$

A 250 mL Young's ampoule was charged with $ThCl_4(DME)_2$ (22.047 g, 39.80 mmol)[1] and the solid dissolved in THF (~150 mL). Note - the volume of THF used is critical for success of the reaction: it must be a large excess. The solution is heated at 60 °C for 24 hours, then cooled to room temperature and all volatiles removed *in vacuo*. The reaction progress is monitored by ^1H NMR spectroscopy of the solid, which is partially soluble in C_6D_6. The solid will initially be a mixture of $ThCl_4(DME)_2$ and $ThCl_4(THF)_{3.5}$. Repeat the dissolution of the solid and heating in THF until the NMR spectrum of the solid shows that all DME has been removed. This usually requires 3-4 cycles. Once all DME has

been removed, the pale white solid is washed with hexane (2 × 30 mL), and dried *in vacuo* to give $ThCl_4(THF)_{3.5}$ as a white powder. Yield: 24.76 g, 99.4%. ^{1}H NMR (C_6D_6, 298 K): δ 4.21 (4H, s, -C*H*$_2$O), 1.39 (4H, s, -C*H*$_2$CH$_2$O) ppm. ^{13}C{^{1}H} NMR (C_6D_6, 298 K): δ 72.61, 25.48 ppm. FTIR (ATR) ν/cm^{-1}: 2960 (m), 2896 (m), 1465 (m), 1450 (s), 1348 (s), 1302 (w), 1252 (m), 1192 (w), 1071 (w), 1039 (m), 998 (s), 956 (m), 913 (s), 844 (w), 821 (s), 675 (s), 573 (m). Where applicable, this data matches that reported in the literature.[1]

Modified Synthesis of MC_8 (where M = K, Rb, Cs)

Generic method for the synthesis of the K analogue: A 250 mL round-bottomed Schlenk flask was charged with reagent grade (> 99.9%) graphite (3.55 g, 818.4 mmol) and dried under dynamic vacuum (1 × 10^{-3} mbar) at 100 °C for four hours. In an argon-filled glovebox, freshly cut potassium metal (1.45 g, 102.3 mmol) is added. The mixture is then heated under an argon atmosphere with a blowtorch whilst agitating causing the potassium metal to melt and intercalation to occur. Continue heating until the mixture has completely changed colour from black to bronze. Where M = K, this will be for approximately 3 hours. Once the reaction is complete, allow to cool to room temperature. Yield: 5.0 g, 99%. Where M = Rb and Cs, the heating step will be approximately 30-60 minutes.

Modified Synthesis of $C_8H_8K_2$

A 500 mL round-bottomed Schlenk flask was charged with freshly cut potassium metal (3.12 g, 80 mmol) and suspended in THF (200 mL). At room temperature, freshly distilled 1,3,5,7-cyclooctatetraene (4.16 g, 40 mmol) was added slowly resulting in the gradual formation of a dark-brown solution. The mixture was stirred for 12 hours before being filtered. Volatiles were removed *in vacuo* to yield a pale-brown, highly pyrophoric solid. Yield: 6.80 g, 93.2%. ^{1}H NMR (C_6D_6/THF, 298 K): δ 6.47 (s, C$_8$*H*$_8$) ppm. ^{13}C{^{1}H} NMR (C_6D_6/THF, 298 K): δ 90.61 ppm. FTIR (ATR) ν/cm^{-1}: 2987 (s), 2903 (w), 2869 (m), 1457 (w), 1425 (m), 1033 (s), 918 (w), 879 (s), 762 (w), 729 (w), 703 (s), 679 (m), 668 (s), 658 (s).

Modified Synthesis of $[Th(\eta^8\text{-}C_8H_8)_2]$

A 500 mL Young's ampoule equipped with a PTFE stirrer bar was charged with $ThCl_4(THF)_{3.5}$ (7.675 g, 12.25 mmol) and $C_8H_8K_2$ (4.47 g, 24.51 mmol). The mixture was cooled to –78 °C, then THF (100 mL) was added, and the suspension was allowed to warm slowly to room temperature. The resultant yellow reaction mixture was stirred for 48 hours before solvent was removed *in vacuo* to obtain a bright yellow powder (9.11 g). Using a 500 mL Schlenk flask containing toluene (100 mL), $[Th(\eta^8\text{-}C_8H_8)_2]$ was extracted as a bright yellow microcrystalline powder *via* Soxhlet extraction for 5 days at 120 °C. Yield: 3.607 g, 67%. ^{1}H NMR (D$_8$-THF, 298 K): δ 6.58 (s, 16H, C$_8$*H*$_8$) ppm. ^{13}C{^{1}H} (D$_8$-THF, 298 K): δ 108.06 ppm. FTIR (ATR) ν/cm^{-1}: 2961 (w), 1317 (w), 1261 (s), 1088 (w), 1025 (m), 964 (w), 897 (m), 856 (w), 801 (s), 743 (m), 696 (s). Raman ν/cm^{-1} (785 nm): 3031 (w), 3009 (w), 1491 (m), 745 (s), 714 (w), 383 (s), 259 (m), 233 (s), 217 (m), 63 (w). This Raman data matches up excellently with that reported in the literature.[18]

Modified Synthesis of $[Th(\eta^8\text{-}C_8H_8)(Cl)_2(THF)_2]$ (**1**)

A 500 mL Schlenk flask equipped with a PTFE stirrer bar was charged with $ThCl_4(THF)_{3.5}$ (5.06 g, 8.08 mmol) and $[Th(\eta^8\text{-}C_8H_8)_2]$ (3.56 g, 8.08 mmol). THF (100 mL) was added at room temperature with stirring to form a yellow suspension, which was refluxed at 100 °C for four days. The resulting golden suspension was filtered hot through a Celite-packed coarse porosity frit, and the solvent removed *in vacuo* from the resulting filtrate to yield **1** as an off-white solid which was used without further purification. Yield: 8.36 g, 94%. ^{1}H NMR (D$_8$-THF, 298 K): δ 6.63 (s, 8H, C$_8$*H*$_8$), 3.53 (8H, s, -C*H*$_2$), 1.68 (8H, s, -C*H*$_2$) ppm. ^{13}C{^{1}H} (D$_8$-THF, 298 K): δ 102.59 (s, *C*$_8$H$_8$), 68.39 (s, -*C*H$_2$), 26.53 (s, -*C*H$_2$) ppm. FTIR (ATR) ν/cm^{-1}: 2962 (s), 2900 (w), 1468 (w), 1455 (w) 1343 (w) 1209 (s), 1089 (m), 1008 (s), 904 (m), 852 (m), 797 (s), 717 (s), 669 (m). Raman ν/cm^{-1} (638 nm): 3047 (s),

2971 (s), 2902 (s), 1504 (s), 1450 (m), 1365 (w), 1233 (m), 1183 (m), 1148 (w), 1039 (m), 925 (m), 862 (m), 804 (w), 754 (s), 371 (w), 238 (s), 161 (w), 117 (w), 77 (w).

Synthesis of [M(2.2.2-cryptand)][{(η^8-C_8H_8)Th(μ-Cl)$_2$}$_3$] (M = K, **4K**; Rb, **4Rb**; Cs, **4Cs**)
*Representative procedure for **4Cs**:* Using a Young's ampoule equipped with a PTFE stirrer bar, a solution of **1** (0.50 g, 0.90 mmol) and 2.2.2-cryptand (0.3388 g, 0.90 mmol) in THF (10 mL) was added to a stirring suspension of CsC_8 (0.21 g, 0.90 mmol) in benzene (20 mL) at room temperature. The resultant mixture was stirred overnight resulting in the gradual formation of a dark blue solution, with concomitant deposition of a black solid. Volatiles were removed *in vacuo* and soluble residues were then extracted into DME (~10 mL) to obtain a dark blue solution. Removal of volatiles *in vacuo* yielded **4Cs** as a turquoise blue powder. Yield: 0.20 g, 39% (by Th content). Single crystals of **4Cs** suitable for XRD were obtained by slow diffusion of pentane into a saturated DME solution at room temperature. Compounds **4M** are completely insoluble in hydrocarbon and arene solvents and sparingly soluble with limited stability in d_8-THF (order of solubility and stability follows **4K** > **4Rb** > **4Cs**). Anal. Calc. for $C_{42}H_{60}Cl_6CsN_2O_6Th_3$: C, 29.15; H, 3.49; N, 1.62%. Found: C, 29.46; H, 3.50; N, 1.74%. ^{1}H NMR (D_8-THF, 298 K): δ 3.60 (s, 12H, CH_2-cryptand) 3.52 (t, 12H, CH_2-cryptand) 2.57 (t, 12H, CH_2-cryptand) ppm. ^{13}C{^{1}H} (D_8-THF, 298 K): δ 71.96 (s, CH$_2$-cryptand), 70.98 (s, CH$_2$-cryptand), 57.76 (s, CH$_2$-cryptand) ppm. The ^{1}H and ^{13}C signal for the Th-bound $C_8H_8^-$ is not observed, presumably due to the paramagnetic nature of **4Cs**; this is discussed in the Supplementary Text. FTIR (ATR) ν/cm^{-1}: 3027 (m), 2954 (w), 2868 (s), 2811 (w), 1471 (m), 1443 (m), 1351 (s), 1294 (m), 1256 (m), 1064 (s), 1021 (s), 940 (s), 901 (s), 815 (w), 747 (w), 741 (w), 707 (s), 506 (m). Raman ν/cm^{-1} (785 nm): 3050–2800 (w, br), 2147 (w), 2101 (w), 1497 (m), 1467 (w), 1433 (w), 1290 (w), 1116 (w), 1090 (w), 1057 (w), 1024 (w), 899 (w), 832 (w), 813 (w), 778 (w), 748 (s), 370 (s), 328 (w), 259 (w), 243 (w), 224 (s), 135 (m), 99 (m), 75 (m), 53 (m).

*Characterization Data for **4K**:* The procedure is identical to that of **4Cs**. Yield: 0.05 g, 10.1% (by Th content). Single crystals of **4K** suitable for XRD were obtained by slow diffusion of pentane into a saturated DME solution at room temperature. Anal. Calc. for $C_{42}H_{60}Cl_6KN_2O_6Th_3$: C, 30.82; H, 3.69; N, 1.71%. Found: C, 30.68; H, 3.53; N, 1.49%. ^{1}H NMR (D_8-THF, 298 K): δ 3.57 (s, 12H, CH_2-cryptand) 3.53 (t, 12H, CH_2-cryptand) 2.55 (t, 12H, CH_2-cryptand) ppm. ^{13}C{^{1}H} (D_8-THF, 298 K): δ 71.54 (s, CH$_2$-cryptand), 68.70 (s, CH$_2$-cryptand), 54.98 (s, CH$_2$-cryptand) ppm. The ^{1}H and ^{13}C signal for the Th-bound $C_8H_8^-$ is not observed, presumably due to the paramagnetic nature of **4K**; this is discussed in the Supplementary Text. FTIR (ATR) ν/cm^{-1}: 3024 (w), 2878 (m), 2814 (w), 1850 (w), 1745 (w), 1611 (w), 1472 (w), 1445 (m), 1353 (s), 1295 (w), 1257 (m), 1191 (w), 1129 (m), 1099 (s), 1032 (m), 947 (s), 900 (s), 854 (m), 777 (w), 749 (m), 706 (s), 485 (s). Raman ν/cm^{-1} (785 nm): 1503 (w), 1359 (m), 896 (w), 850 (w), 802 (w), 751 (s), 714 (w), 623 (w), 579(w), 529 (w), 435 (w), 367(m), 330 (w), 300 (w), 245 (s), 225 (s), 177 (w), 135 (w), 87 (s), 66 (w), 53 (w).

*Characterization Data for **4Rb**:* The procedure is identical to that of **4Cs**. Yield: 0.07 g, 13.8% (by Th content). Single crystals of **4Rb** suitable for XRD were obtained by slow diffusion of pentane into a saturated DME solution at room temperature. Anal. Calc. for $C_{42}H_{60}Cl_6RbN_2O_6Th_3$: C, 29.97; H, 3.59; N, 1.66%. Found: C, 29.76; H, 3.58; N, 1.37%. ^{1}H NMR (D_8-THF, 298 K): δ 3.57 (s, 12H, CH_2-cryptand) 3.53 (t, 12H, CH_2-cryptand) 2.55 (t, 12H, CH_2-cryptand) ppm. ^{13}C{^{1}H} (D_8-THF, 298 K): δ 71.68 (s, CH$_2$-cryptand), 68.77 (s, CH$_2$-cryptand), 55.33 (s, CH$_2$-cryptand) ppm. The ^{1}H and ^{13}C signal for the Th-bound $C_8H_8^-$ is not observed, presumably due to the paramagnetic nature of **4Rb**; this is discussed in the Supplementary Text. FTIR (ATR) ν/cm^{-1}: 3026 (w), 2961 (m), 2874 (m), 2810 (w), 1751 (w), 1616 (w), 1472 (m), 1443 (m), 1351 (s), 1295 (w), 1258 (s), 1093 (s), 1071 (s), 1016 (s), 944 (s), 924 (m), 900 (m), 796 (s), 748 (w), 704 (s), 516 (m), 490 (w), 450 (w). Raman ν/cm^{-1} (785 nm): 1497 (w), 1475 (w), 897 (w), 853 (w), 775 (w), 747 (s), 702 (w), 623 (w), 531 (w), 486 (w), 367 (s), 243 (s), 223 (s), 136 (w), 82 (m), 65 (w), 52 (w).

Synthesis of **4Cs** in Deuterated Solvents

Using a 20 mL scintillation vial equipped with a PTFE stirrer bar, a solution of **1** (0.0337 g, 0.06 mmol) and 2.2.2-cryptand (0.023 g, 0.06 mmol) in D_8-THF (2 mL) was added to a stirring suspension of CsC_8 (0.014 g, 0.06 mmol) in D_6-benzene (5 mL) at room temperature. The resultant mixture was stirred overnight resulting in the gradual formation of a dark blue solution, with concomitant deposition of a black solid. Volatiles were removed *in vacuo* and soluble residues were then extracted into DME (~5 mL) to obtain a dark blue solution. Removal of volatiles *in vacuo* yielded **4Cs** as a turquoise blue powder. FTIR (ATR) ν/cm^{-1} deuterio (*versus* protio) : 3026 (w) (*vs.* 3027), 2952 (w) (*vs.* 2954), 2861 (s) (*vs.* 2868), 2807 (m) (*vs.* 2811), 1475 (m) (*vs.* 1471), 1445 (m) (*vs.* 1443), 1356 (s) (*vs.* 1351), 1295 (m) (*vs.* 1294), 1260 (s) (*vs.* 1256), 1104 (s) (*vs.* 1106), 1095 (s) (*vs.* 1093), 1067 (s) (*vs.* 1064), 940 (m) (*vs.* 940), 848 (s) (vs. 852), 741 (m) (*vs.* 741), 707 (s) (*vs.* 707), 509 (m) (*vs.* 506). The infrared spectra of **4Cs** prepared in protio or deuterio solvents are extremely similar. Bridging hydrides or deuterides, respectively, would be expected to present hydride absorptions at 790–950 and 1,100–1,300 cm^{-1} with D isotopologue shifts of ~230–380 cm^{-1}.[19] Such absorptions are not observed.

Hydride Test on **4Cs**

A suspension of **4Cs** (0.01 g, 0.005 mmol) in D_6-benzene (~0.6 ml) was prepared in Young's ampoule equipped with a PTFE stirrer bar. The suspension was frozen and CCl_4 (~0.3 ml) was distilled into the vessel in vacuo at −196 °C. The suspension was allowed to warm to room temperature; the dark blue suspension gradually turned off-white after 1 hour of vigorous stirring. ^{1}H NMR (C_6D_6, 298 K): δ 5.78 (s), 5.62 (s), 3.60 (s, 12H), 3.46 (s, 12H), 2.51 (s, 12H), 2.27 (s, 1H), 0.22 (s, 1H) ppm. ^{13}C{^{1}H} NMR (C_6D_6, 298 K): δ 96.50, 71.39, 70.41, 57.04 ppm. The ^{13}C{^{1}H} NMR signal at 96.50 ppm is residual CCl_4. ^{1}H and ^{13}C{^{1}H} NMR spectroscopies find no evidence of $CHCl_3$ or CH_2Cl_2 formation ($CHCl_3$, ^{1}H δ 6.15 and ^{13}C{^{1}H} δ 77.79 ppm: CH_2Cl_2, ^{1}H δ 4.27 and ^{13}C{^{1}H} δ 53.46 ppm).[20]

Reaction of **4Cs** with Excess 1,3,5,7-Cyclooctatetraene

A blue solution of **4Cs** (0.02 g, 0.011 mmol) in D_8-THF (~0.6 ml) was prepared in a Young's NMR tube. Freshly distilled C_8H_8 (8.5 mg, 0.08 mmol, 7 equiv.) was then added to the vessel. There was a rapid stepwise colour change from blue to purple to colourless, with the precipitation of dark blue solid which rapidly became pale grey in colour. NMR spectroscopic analysis showed the presence of solely 1,3,5,7-cyclooctatetraene. ^{1}H NMR (D_8-THF, 298 K): δ 5.72 (s) ppm. ^{13}C{^{1}H} NMR (D_8-THF, 298 K): δ 132.91 ppm. The grey solid was separated from the mother liquor. FTIR (ATR) ν/cm^{-1}: 2982 (w), 2921 (w), 2874 (w), 1574 (s), 1521 (s) 1438 (m), 1362 (s), 1268 (m), 1186 (w), 1076 (w), 1022 (s), 946 (w), 927 (m), 869 (w), 820 (w), 709 (m), 676 (m), 628 (w), 518 (s). The absorptions in the FTIR data at ~1575 and 1520 cm^{-1} are indicative of aromatic C=C stretches. These values are shifted with respect to that of neutral 1,3,5,7-cyclooctatetraene (~1640 cm^{-1}).[21] The absorption in the FTIR data at ~1000 cm^{-1} is indicative of aromatic C=C-H in-plane bending. Anal. Found: C, 38.47; H, 5.23; N, 3.23%. We cannot be sure of the precise formulation, but note that [Cs(2.2.2-cryptand)]$_3$[Cs(COT)]$_3$CsCl is realistic: Anal. Calc'd: C, 38.91; H, 5.53; N, 3.49%.

Reaction of Solid **4Cs** with CO_2

Solid turquoise blue **4Cs** (0.01 g, 0.005 mmol) was loaded into a Young's NMR tube and subjected to dynamic vacuum (1 × 10^{-3} mbar) for 1 hour before being placed under an atmosphere of CO_2 (1 bar gauge pressure). After 1 hour, the powder was beginning to appear less intense in colour but still visibly blue. After 12 hours, the powder had changed colour completely to grey. ^{1}H NMR (C_6D_6, 298 K): δ 7.36 (s), 6.93 (s), 5.64 (s), 3.65 (s), 3.50 (t), 3.34 (s), 3.13 (s), 2.53 (t), 0.30 (s) ppm. ^{13}C{^{1}H} NMR (C_6D_6, 298 K): δ 124.75, 71.40, 70.42, 57.04 ppm. FTIR (ATR) ν/cm^{-1}: 2940 (w), 2863 (s), 2787 (s), 2718 (w), 1559 (m), 1489 (w), 1461 (w), 1444 (m), 1393 (w), 1354 (s), 1328 (w), 1294 (s), 1262 (s), 1213 (w), 1122 (s), 1067 (m), 1034 (w), 1018 (w), 980 (s), 941 (m), 919 (s), 850 (m), 832 (w), 802 (s), 737 (w), 709 (s), 581 (m), 527 (m), 505 (w), 473 (m). The grey solid was subsequently

washed with toluene (3 × 2 mL) to yield an insoluble pale-yellow solid which was separated from the mother liquor. FTIR (ATR) ν/cm^{-1}: 3030 (w), 2947 (w), 2862 (s), 1540 (s), 1471 (m), 1443 (m), 1353 (s), 1298 (m), 1279 (w), 1255 (m), 1232 (w), 1107 (s), 1095 (s), 1065 (m), 1021 (w), 944 (s), 905 (s), 831 (w), 816 (w), 778 (w), 744 (m), 711 (s), 506 (m), 465 (w). The absorption in the FTIR data at ~1550 cm^{-1} is indicative of carbonate formation.[22-25] The insolubility of the solid precluded the acquisition of ^{13}C NMR data hence no signals consistent with oxalate or carbonate formation could be observed.

Solution Reaction of **4Cs** with CO_2

A blue solution of **4Cs** (0.005 g, 0.003 mmol) in D_8-THF (~0.6 ml) was prepared in a Young's NMR tube. The suspension was freeze-pump-thaw degassed three times, then placed under an atmosphere of CO_2 (1 bar). The suspension was allowed to warm to room temperature leading to the immediate colour change of the solution from blue to colourless with the precipitation of a pale-yellow solid. ^{1}H NMR (D_8-THF, 298 K): δ 5.68 (s), 3.49 (s), 2.53 (s), 2.43 (s), 0.08 (s) ppm. ^{13}C{^{1}H} NMR (D_8-THF, 298 K): δ 126.13 ppm. The pale-yellow solid was separated from the mother liquor. FTIR (ATR) ν/cm^{-1}: 2866 (s), 2788 (m), 2718 (w), 1720 (w), 1673 (m), 1541 (s), 1448 (m), 1360 (s), 1328 (w), 1295 (m), 1261 (s), 1123 (s), 1068 (w), 1014 (s), 980 (s), 920 (m), 849 (w), 800 (s), 735 (m), 671 (w), 556 (s), 465 (s). The absorptions in the FTIR data at ~1640 and 1550 cm^{-1} are indicative of oxalate and carbonate formation.[22-25] The insolubility of the solid precluded the acquisition of ^{13}C NMR data hence no signals consistent with oxalate or carbonate formation could be observed.

Isolation of [{Th(η^{8}-C_8H_8)(μ-Cl)$_2$(μ$_3$-O)}$_3$(Cs)$_2$(DME)$_3$] (**5**) from attempted reduction of **1**

A Young's ampoule equipped with a PTFE stirrer bar was charged with **1** (0.2 g, 0.36 mmol) and CsC_8 (0.082 g, 0.36 mmol). Benzene and THF (2:1, 15 mL) were added at room temperature, and the resultant suspension allowed to stir for five days. Stirring was then ceased and the precipitate was allowed to settle. The colourless supernatant was decanted, the remaining solvent was removed *in vacuo* from the precipitate, and the resultant brown solid was then dried for two hours. Soluble residues were extracted into DME (~10 mL) and storage of this dark brown solution at −30 °C for one week resulted in the formation of dark brown crystals. Compound **5** is completely insoluble in hydrocarbon and arene solvents. Yield: 0.05 g, 23.5% (by Th content). Anal. Calc. for $C_{36}H_{54}Cl_6Cs_2O_7Th_3$: C, 24.38; H, 3.07%. Found: C, 24.30; H, 2.65%. ^{1}H NMR (D_8-THF, 298 K): δ 6.68 (s, 8H, Cs-bound C_8H_8), 6.45 (s, 16H, $C_8\textit{H}_8$), 3.43 (br, s, 12 H, DME $C\textit{H}_2OCH_3$), 3.27 (br, s, 18 H, DME $CH_2OC\textit{H}_3$) ppm. ^{13}C{^{1}H} (D_8-THF, 298 K): δ 102.60 (s, $\textit{C}_8H_8$), 102.44 (s, $\textit{C}_8H_8$), 72.94 (s, DME $\textit{C}H_2OCH_3$), 59.07 (s, DME $CH_2O\textit{C}H_3$) ppm. The presence of two C_8H_8 environments in solution suggests that some of the C_8H_8 bonding motifs observed in the solid-state structure are maintained in solution. FTIR (ATR) ν/cm^{-1}: 3018 (w), 2921 (w), 2878 (w), 2810 (w), 1851 (w), 1758 (w), 1630 (w),1448 (w), 1363 (w), 1316(w), 1261(w), 1236 (w), 1185 (w), 1117(w), 1074(m), 1024 (w), 900 (m), 849 (w), 790 (w), 701 (s), 476 (w). Raman ν/cm^{-1} (532 nm): 3049 (s), 2977 (w), 2917 (s, br), 2765 (w), 2712 (w), 1603 (s), 1499 (m), 1441 (m), 1284 (m), 1225 (w), 1183 (w), 1153 (w), 1070 (w), 1028 (w), 998 (m), 897 (w), 847 (w), 780 (w), 749 (s), 717 (w), 617 (w), 365 (m), 241 (s), 224 (s), 59 (s, br), 15 (s).

General Computational Details

Geometry optimizations were performed with ORCA 5.0.3.[26] Relativistic effects were treated using the zeroth-order regular approximation (ZORA).[27] The PBE0 hybrid exchange-correlation functional[28] was used with Grimme's D3 dispersion[29] with Becke-Johnson damping parameters (D3(BJ)).[30,31] The SARC-TZVPP basis set[32] was used for Th and Cs, and def2-TZVPP[33] for H, C, and Cl; the fitting basis SARC/J[34] was employed to speed up the calculation of integrals using the RIJCOSX approximation,[35] and the NOTRAH and TightSCF convergence settings were used. Harmonic vibrational frequency analyses confirmed the local true minimum geometries (i.e. lack of

imaginary frequencies). Foster-Boys and Pipek-Mezey localization methods were also conducted with ORCA 5.0.3.

The domain-based localized pair natural orbital variant of CCSD (DLPNO-CCSD)[36, 37] was employed to obtain T_1 diagnostic value as implemented in ORCA using the following settings: SARC-TZVP basis set for Th, def2-SVP for ligand atoms with NormalPNO thresholds and the 2nd order Douglas-Kroll-Hess Hamiltonian.[38,39] The fitting basis sets for RIJCOSX were generated using the AutoAux function.[40]

The Raman and IR spectra were calculated with Gaussian 16,[41] revision C.01, using the methodology as in our original publication,[42] i.e. using the small Stuttgart-Dresden effective core potential (ECP) and associated ECP basis set for Th, cc-pVTZ for C and Cl, and cc-pVDZ for H, together with the D3(BJ) dispersion corrections.[43-45] The geometry was reoptimized and the ultrafine integration grid was used. The RMSD between the ORCA and Gaussian geometries is 0.008 Å, and the Th–Th bond lengths are 4.092 and 4.103 Å, respectively. The Gaussian-reoptimized geometry was used only for the Raman and IR spectra calculations. In all other calculations, the geometry from ORCA was used.

The EPR g-values[46] were calculated using ADF 2023,[47] with the PBE0 functional and the all-electron TZ2P basis set.[48] The relativistic effects were treated using the spin-orbit ZORA Hamiltonian, with collinear spin-orbit magnetization. The numerical quality was set as "VeryGood".

UV/Vis/NIR spectra were calculated using ORCA 5.0.3 by employing time-dependent density functional theory (TD-DFT) to calculate 20 excited states. The same settings as for the geometry optimization were used.

Wavefunction analyses (ELF, bond orders, MCI) were carried out with MultiWFN 3.8[49] using molden input files from ORCA calculations (generated with orca_2molden tool). QTAIM topology analysis was performed using MultiWFN and figures with bond paths were generated using AIMAll (version 19.02).

Adaptive natural density partitioning (AdNDP)[50] and electron density of delocalized bonds[51] ($EDDB_F$) analyses were performed as described in our recent publication[52] (using Gaussian 16 and NBO7[53] single point calculations as a starting point).

Anisotropy of the induced current density (AICD) analysis was performed using the AICD 3.0.4 software provided by the Herges group[54] with the underlying Gaussian 16 calculation as we described previously.[52]

Iso-chemical surface shielding (ICSS)[55] calculations were performed by running NMR calculations in Gaussian 16 with input files generated by MultiWFN. The settings for Gaussian 16 calculations were the same as in our recent publication.[52]

The gauge-including magnetically induced currents (GIMIC 2.0)[56-58] analyses were performed using the output files from Gaussian 16 (with the same settings as for AICD calculations). The current densities were calculated numerically with a cubic grid (22 bohr long) positioned around the molecule with 50 × 50 × 50 grid points. The applied magnetic field was pointing down along the z-axis. In the ring current strength calculations, the integration planes were starting from the middle of the tri-thorium triangle and cutting through the middle of the Th–Th bond perpendicular to the bond while the magnetic field was pointing down the z-axis. The integration plane was extending 12 bohr above and below, 12 bohr outside and 2.2372 bohr (i.e., a distance between the centre of the tri-thorium triangle and the Th–Th midpoint) inside of the Th–Th midpoint with the default grid spacing.

The net isotropic magnetic susceptibilities were calculated using the NMR shielding and magnetic susceptibility module within Gaussian 16[59] with the same setting as for AICD. For **4'** the coordinates from the ORCA 5 were used, and for **6** the same coordinates as **4'** were also used but the valence-delocalized electron was omitted to obtain a $S = 0$ formulation instead of $S = 1/2$. A more accurate evaluation of the net isotropic magnetic susceptibility of **4M** awaits the implementation of magnetic susceptibility calculations that accommodate all-electron spin-orbit models with unpaired electrons and being able to include a large external magnetic field, since the calculations operate in the applied field region where experimentally and computationally the magnetization is only slightly negative.

Energy decomposition analysis (EDA)[60-62] was performed using ADF (v2023.105).[47] The geometry of $[Cs(2.2.2\text{-cryptand})]^+[\{(\eta^8\text{-}C_8H_8)Th(\mu\text{-}Cl)_2\}_3]^-$ ion pair was taken from the XRD data of **4Cs**. The EDA calculation was done with PBE-D3(BJ),[63] TZ2P basis set (without frozen cores),[48] ZORA Hamiltonian[27,64,65] and the numerical quality increased to the level of "Good".

Supplementary Text

Salient SC-XRD Details

In the solid-state structures of **4M**, there are short Cl···H and Cl···C distances between separate molecules: **4K** = 3.019(5) Å and 3.76(3) Å; **4Rb** = 2.853(3) Å and 3.627(17) Å; **4Cs** = 2.961(2) Å and 3.837(6) Å. These distances are comparable to, or longer than, the sum of van der Waal radii for the respective elements Cl-H (2.86 Å) and Cl-C (3.53 Å).[66] These intermolecular interactions may infer additional weak stabilization to the $[\{(\eta^8\text{-}C_8H_8)Th(\mu\text{-}Cl)_2\}_3]^-$ units but could also simply be a result of crystal packing with no strong interaction present. The structures of **4K**, **4Rb**, and **5** generate Level A and B Alerts in the IUCR's CheckCif routine regarding calculated residual electron density and low bond precision on C-C bonds; these are due to residual electron density close to heavy atoms that is not chemically significant and present despite applying absorption corrections and due to disorder or low data quality, respectively.

Salient NMR Details

In contrast to polymeric **3**, ^1H and $^{13}C\{^1H\}$ solution NMR spectra of the separated ion pairs **4M** could be acquired. Compounds **4M** are completely insoluble in hydrocarbon and arene solvents, and sparingly soluble with limited stability in d_8-THF (order of solubility and stability follows **4K** > **4Rb** > **4Cs**). This ordering follows that reported for the solution phase solubility and stability of alkali metal cation 222-cryptates more generally.[67-71] Compounds **4M** are more soluble and stable in dimethoxyethane (DME), but not sufficiently so to be rendered practicable, hence d_8-THF was the most appropriate compromise to obtain reliable NMR data.

In the ^1H NMR spectra, several resonances can be visualized in the region anticipated for the C_8H_8 H-atoms (6.0-7.0 ppm; note $[Th(\eta^8\text{-}C_8H_8)_2]$ and and **1** display singlet resonances at 6.58 and 6.78 ppm, respectively). However, no carbon C_8H_8 resonances could be located for **4M** in the $^{13}C\{^1H\}$ NMR spectra. Resonances attributed to the $[M(2.2.2\text{-cryptand})]^+$ cation component can be clearly observed in both ^1H and $^{13}C\{^1H\}$ solution NMR spectra.

To further investigate this, variable-temperature (VT) NMR experiments (–60 to +65 °C; starting at 25 °C before cooling to –60 °C and then warming to +65 °C) were performed which exhibit the loss of resonances associated with the $[M(2.2.2\text{-cryptand})]^+$ cation component (at ~3.60, 3.52, and 2.55 ppm, respectively) as **4M** precipitates from the solution as it is cooled. However, peaks visualized in the region anticipated for the C_8H_8 H-atoms (6.0-7.0 ppm) remain, suggesting the former are associated with the **4M** complex but the latter are not, with these resonances instead being trace diamagnetic impurities. In addition, heating >35 °C causes decomposition of **4M**, with coincident loss of blue colour and the emergence of a peak at ~5.70 ppm which can be assigned as 1,3,5,7-cyclooctatetraene. Upon decomposition, there is a slight shift of resonances associated with the

$[M(2.2.2\text{-cryptand})]^+$ cation component whereas, peaks visualized in the region anticipated for the C_8H_8 H-atoms remain.

For **4Cs**, we observed a gradual reduction in intensity of cryptand signals before full sample decomposition at −30 °C occurred (~ 2 hours of measurement time) following by an increase in intensity of presumably free cryptand and the emergence of a peak at ~5.70 ppm which can be assigned as 1,3,5,7-cyclooctatetraene, highlighting the limited stability of this analogue in THF.

Overall, the unassignable nature of the C_8H_8 resonances for these systems reflects the paramagnetic nature of **4M**, and is in common with other thorium(III)-C_8H_8, as well as titanium(III)-C_8H_8, complexes, in addition to d^1 $S = ½$ systems more generally.[72-79]

A methanol standard was used as a low-temperature NMR thermometer to ascertain the temperature within the sample during VT-NMR measurement. This method found the temperatures listed as –10, –20, –30, –40, –50, and –60 °C to be –10.57, –19.54, –29.66, –41.27, –51.37, and –61.20 °C.

Salient ATR-IR Details

Using **4Cs** as an exemplar, IR spectra on samples prepared in protio or deuterio solvents do not evidence isotopologue-shifted absorptions. Addition of CCl_4 to solutions of **4Cs** did not result in the formation of $CHCl_3$ or CH_2Cl_2. Those observations are consistent with **4M** being hydride-free, where hydrides would preclude sub-valent thorium ions and then the compounds would not be blue. Reactions with excess 1,3,5,7-cyclooctatetraene result in the precipitation of a grey solid, whose IR spectra exhibit aromatic C=C stretches and aromatic C=C-H in-plane bending bands suggestive of the formation of a cyclooctatetraenyl dianion dicesium 2.2.2-cryptand salt. For reactions with CO_2, a band at ~1550 cm^{-1} would indicate carbonate formation, whereas oxalate formation would be evidenced by an absorption at ~1640 cm^{-1}. Complex **4Cs** reacts with CO_2 as a solid and in solution. As a solid the reaction is slow and gives a complex carbonate salt whereas in solution the reaction is instantaneous producing both carbonate and oxalate salts. In contrast, **3** reacts with CO_2 only in solution to exclusively give a complex carbonate salt. This reflects the respective one- (**4Cs**) and two-electron (**3**) reduced formulations.

Salient Raman Details

The Raman spectra have common features which can be assigned as follows: (i) bands at ~250 cm^{-1} correspond to symmetric COT-metal stretching and metal-COT tilting modes; (ii) bands at ~380 cm^{-1} correspond to COT CCC in-plane bending modes; (iii) bands at ~750 cm^{-1} are attributed to COT C-C ring breathing modes; (iv) bands at ~1500 cm^{-1} correspond to CC stretching modes; (v) features at ~3000 cm^{-1} correspond to symmetric C-H stretching modes.

Salient UV/Vis/NIR Details

The peak at 16950 cm^{-1} corresponds to electronic transitions to the LUMO (NTO1, 16688 cm^{-1}, 599 nm) and LUMO+1 (NTO2, 16888 cm^{-1}, 592 nm) that are composed 56/25% Th 5f/6d character. Calculated electronic transitions to NTO3 and NTO4 are found at 19669 and 19704 cm^{-1} (508 and 507 nm), which are 57/25% Th 5f/6d character. The absorption at 24330 cm^{-1} is modelled as a composite of six transitions (22591-26203 cm^{-1}) to NTOs 16, 17, 23, 24, 26, and 27 which are predominantly 7s- or 5f-character with minority (≤22%) 6d contributions. In ideal D_{3h} symmetry, the ground state of **4'** is A_1' symmetry and the excited states for the dipole-allowed transitions are E'.

AdNDP, EDDB$_F$, MCI, and ELF Details

The electron delocalization in the trithorium core of **4'** was studied with adaptive natural density partitioning (AdNDP), electron density of delocalized bonds (EDDB$_F$), multicentre indices (MCI), and electron localization function (ELF) analyses. Since **4'** is an open shell system, with AdNDP the

bonding can be partitioned into chemically meaningful n-centre, one-electron fragments for both spins. Bonding components in **4'** were found as in **3'** with the exception of the orbital corresponding to three-centre bonding within the trithorium core, which has an occupation number of 0.92 in **4'** *vs* 1.80 in **3'**. The three-centre SOMO of **4'** is also maintained on performing Foster-Boys and Pipek-Mezey localizations. The two-centre-two-electron dative Th-Cl bonding in **4'** is analogous to that in **3'** so is not discussed. The $EDDB_F$ provides a partitioning of the delocalized electron density, and analysis of the trithorium core of **4'** using the fragment version of $EDDB_F$ yields 0.67 delocalized electrons (*cf.* 1.28 electrons for **3'**). The value of the normalized $MCI^{1/n}$ is 0.30 for **4'** (*cf.*, 0.65 for **3'**). The electron delocalization within the Th_3 core, and it being diminished for **4'** compared to **3'**, is also evident from electron localization function (ELF) analysis. Thus, the AdNDP, $EDDB_F$, $MCI^{1/n}$, and ELF results are consistent with delocalized bonding in the trithorium core of **4'** and, as with the QTAIM results, an approximate 50% reduction in their values relative to **3'** is due to their respective one- and two-electron natures. Isochemical shielding surface (ICSS) calculations reveal positive ICSS values for **4'** indicating regions of delocalized electron density, where the $ICSS_{zz}$ and ICSS values in the centre of the trithorium core of **4'** are +6.9 and +12.6 ppm, respectively (*cf.* +7.9 and +15.4 ppm for **3'**).

Salient EDA Details

Energy decomposition analysis (EDA) was performed with ADF as a single-point calculation using the experimental geometry of **4Cs** to decompose the interaction energy ΔE_{int} between the ions **4'** ($[\{(\eta^8\text{-}C_8H_8)Th(\mu\text{-}Cl)_2\}_3]^-$) and $[Cs(2.2.2\text{-cryptand})]^+$ (Supplementary Figure 123). Within the EDA scheme, the ΔE_{int} term is split into four components $\Delta E_{int} = \Delta E_{Pauli} + \Delta E_{elst.} + \Delta E_{orb.} + \Delta E_{disp.}$: Pauli, electrostatic, orbital and dispersion energy contributions, respectively. The calculated energy contributions are +34.3, −179.3, −31.4, and −38.3 kcal/mol for the Pauli, electrostatic, orbital and dispersion terms. Therefore, electrostatic interactions dominate attractive energy contributions ($\Delta E_{elst.} + \Delta E_{orb.} + \Delta E_{disp.}$), accounting for 72% of the total.

Supplementary Figures

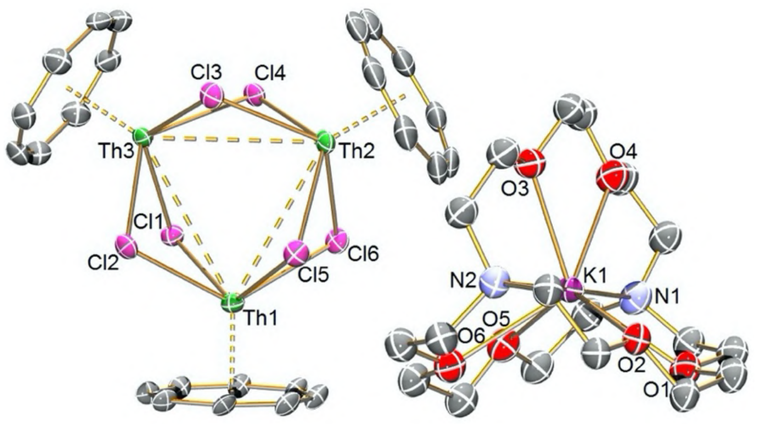

Supplementary Figure 1. Molecular structure of **4K** at 150 K with selected atom labels. Displacement ellipsoids are set at 40% and hydrogen atoms and minor disorder components are omitted for clarity. Key: thorium, green; chlorine, magenta; potassium, purple; oxygen, red; carbon, grey; nitrogen, blue. CCDC 2374489.

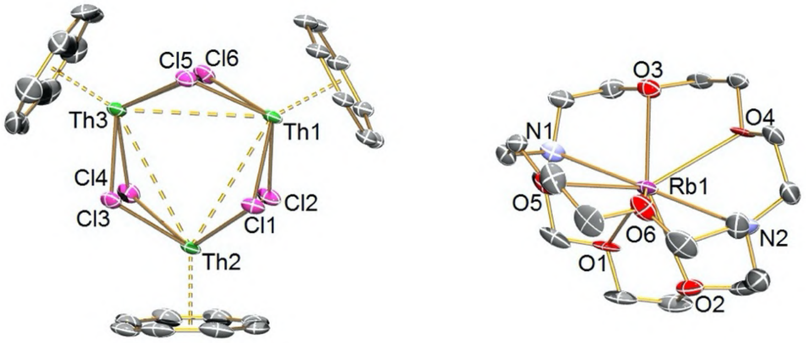

Supplementary Figure 2. Molecular structure of **4Rb** at 100 K with selected atom labels. Displacement ellipsoids are set at 40% and hydrogen atoms are omitted for clarity. Key: thorium, green; chlorine, magenta; rubidium, purple; oxygen, red; carbon, grey; nitrogen, blue. CCDC 2374490.

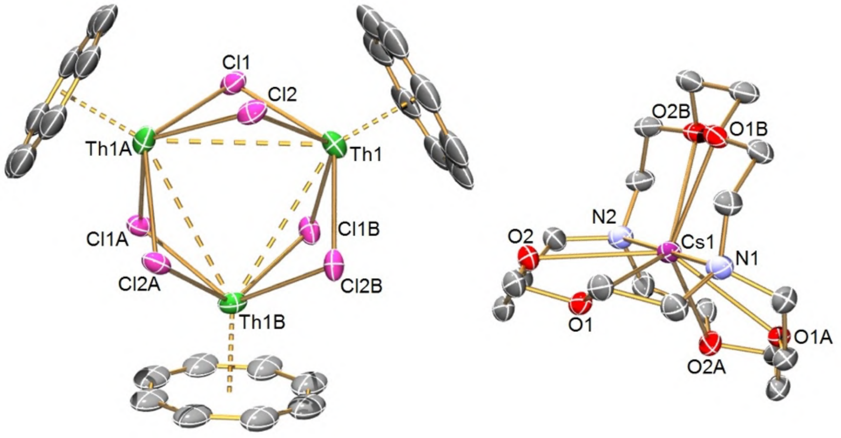

Supplementary Figure 3. Single crystal solid-state structure of 4Cs. Single crystal X-ray diffraction solid-state structure of **4Cs** determined at 150 K. Displacement ellipsoids are rendered at 40% probability and hydrogen atoms are omitted for clarity. The structures of **4K** and **4Rb** are analogous, but with the Cs^+ cation replaced by K^+ or Rb^+ cations, respectively. Key: thorium, green; chlorine, magenta; cesium, purple; oxygen, red; carbon, grey; nitrogen, blue. CCDC 2374491.

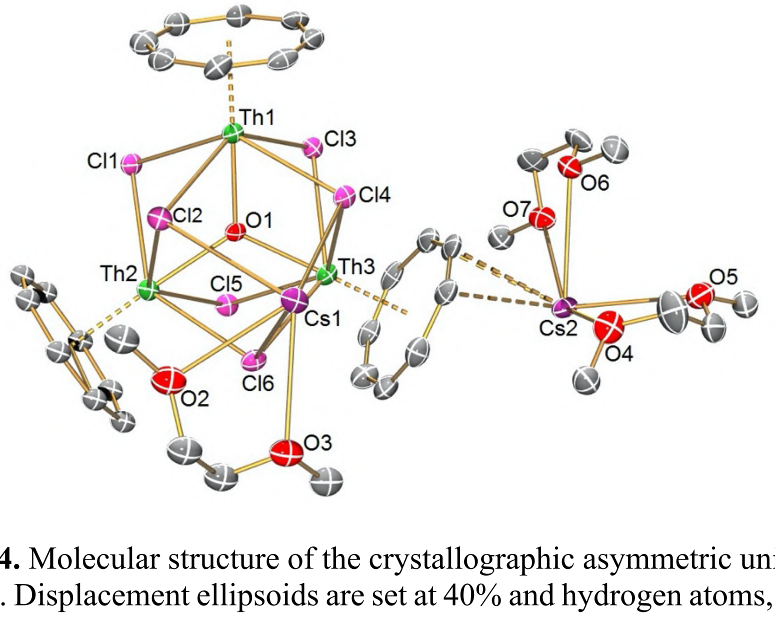

Supplementary Figure 4. Molecular structure of the crystallographic asymmetric unit of **5** at 100 K with selected atom labels. Displacement ellipsoids are set at 40% and hydrogen atoms, lattice solvent, and minor disorder components are omitted for clarity. Cs1 bridges to another three chlorides in a neighboring trithorium cluster, and Cs2 bridges to a C_8H_8 ring in another trithorium cluster via an η^8-interaction, producing a polymeric network. Key: thorium, green; chlorine, magenta; cesium, purple; oxygen, red; carbon, grey. CCDC 2376515.

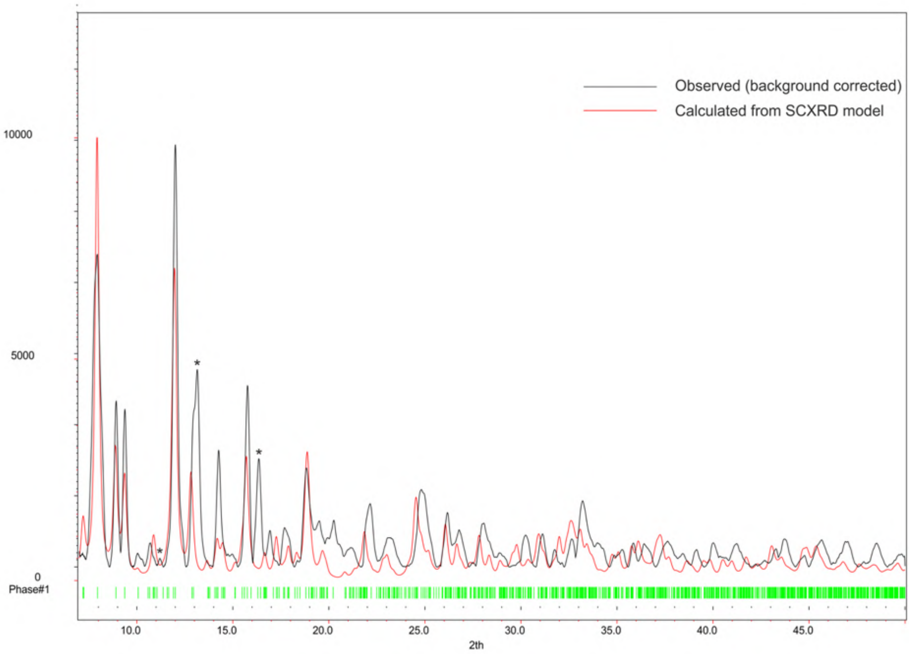

Supplementary Figure 5. Selected-range background-corrected PXRD pattern of **4K** at 100 K used for Le Bail profile fitting showing the experimental powder X-ray diffraction pattern (black), the expected diffraction pattern from the SCXRD data (red, extracted using CCDC Mercury, FWHM 0.25° 2θ), and Miller indices positions (green). The method of data collection in combination with the extremely air-sensitive nature of the complex meant that despite exhaustive efforts, sample degradation occurred during measurement. * = unknown impurity.

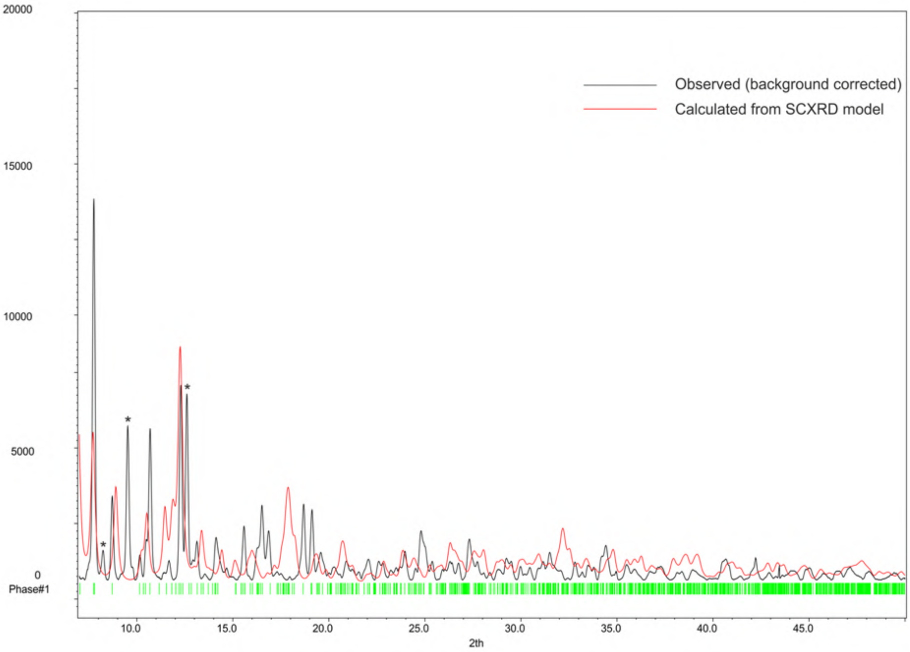

Supplementary Figure 6. Selected-range background-corrected PXRD pattern of **4Rb** at 100 K used for Le Bail profile fitting showing the experimental powder X-ray diffraction pattern (black), the expected diffraction pattern from the SCXRD data (red, extracted using CCDC Mercury, FWHM 0.25° 2θ), and Miller indices positions (green). The method of data collection in combination with the extremely air-sensitive nature of the complex meant that despite exhaustive efforts, sample degradation occurred during measurement. * = unknown impurity.

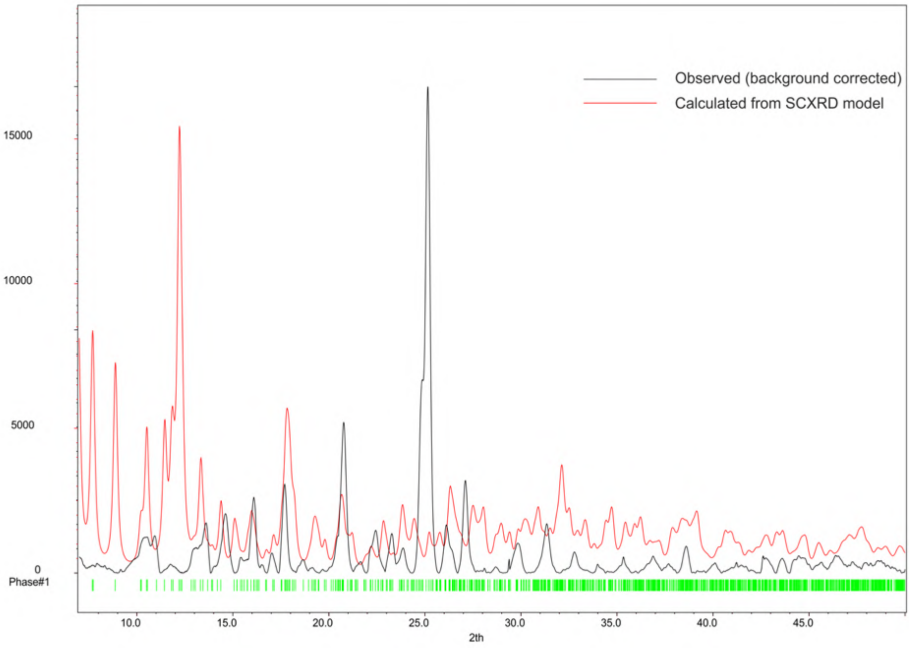

Supplementary Figure 7. Selected-range background-corrected PXRD pattern of **4Cs** at 100 K used for Le Bail profile fitting showing the experimental powder X-ray diffraction pattern (black), the expected diffraction pattern from the SCXRD data (red, extracted using CCDC Mercury, FWHM 0.25° 2θ), and Miller indices positions (green). The method of data collection in combination with the extremely air-sensitive nature of the complex meant that despite exhaustive efforts, sample degradation rapidly occurred during measurement.

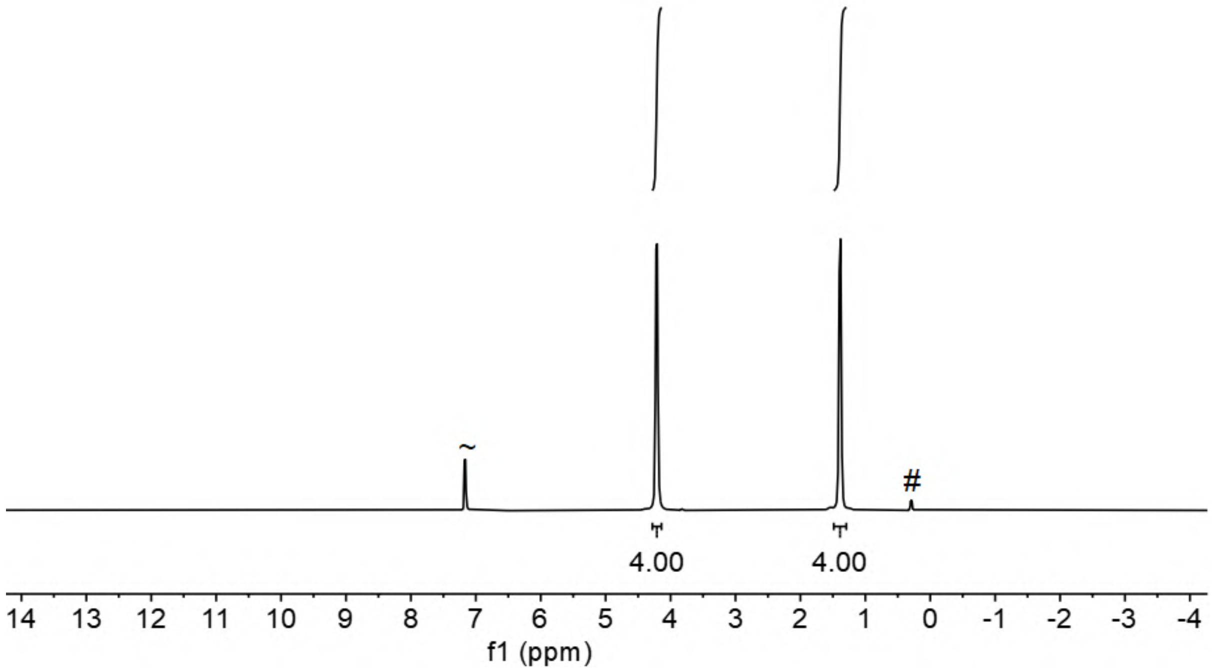

Supplementary Figure 8. ^{1}H NMR (C_6D_6, 298 K) spectrum of $ThCl_4(THF)_{3.5}$. # = silicone grease; ~ = C_6D_6 solvent.

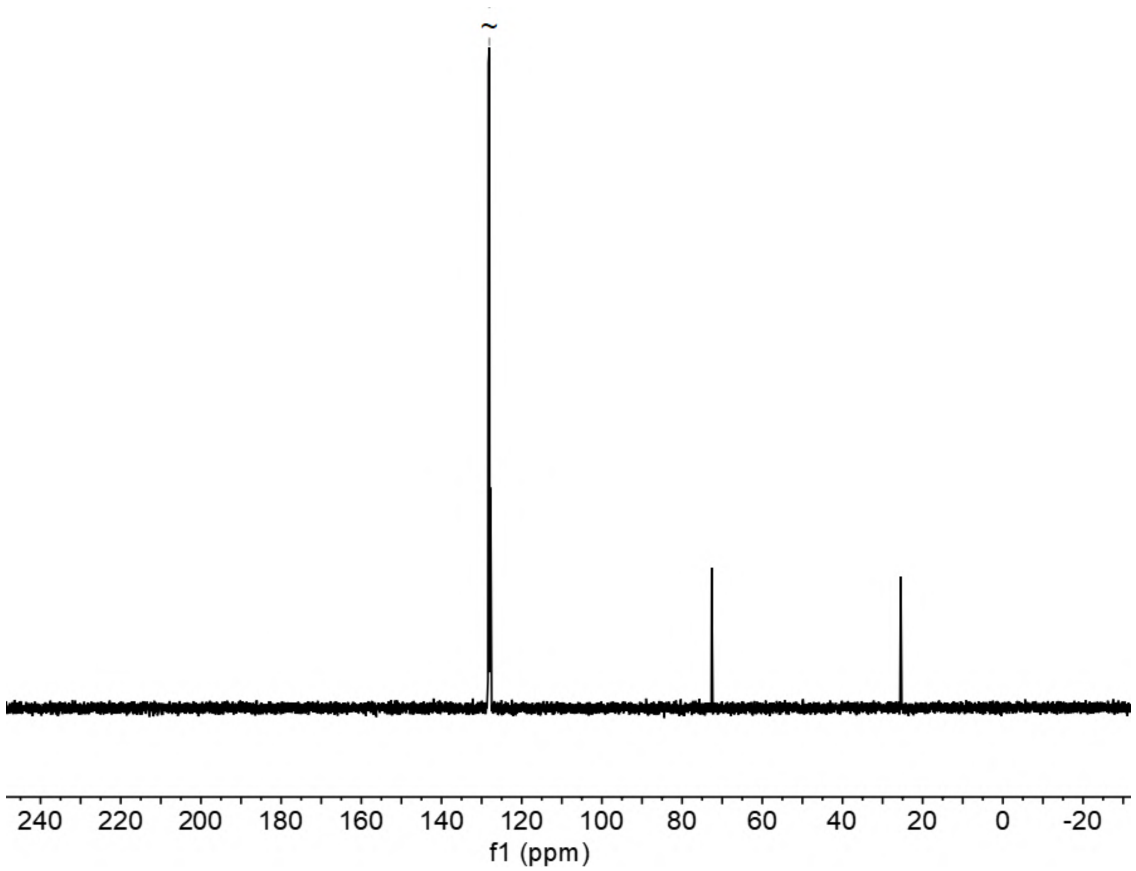

Supplementary Figure 9. $^{13}C\{^{1}H\}$ NMR (C_6D_6, 298 K) of $ThCl_4(THF)_{3.5}$. ~ = C_6D_6 solvent.

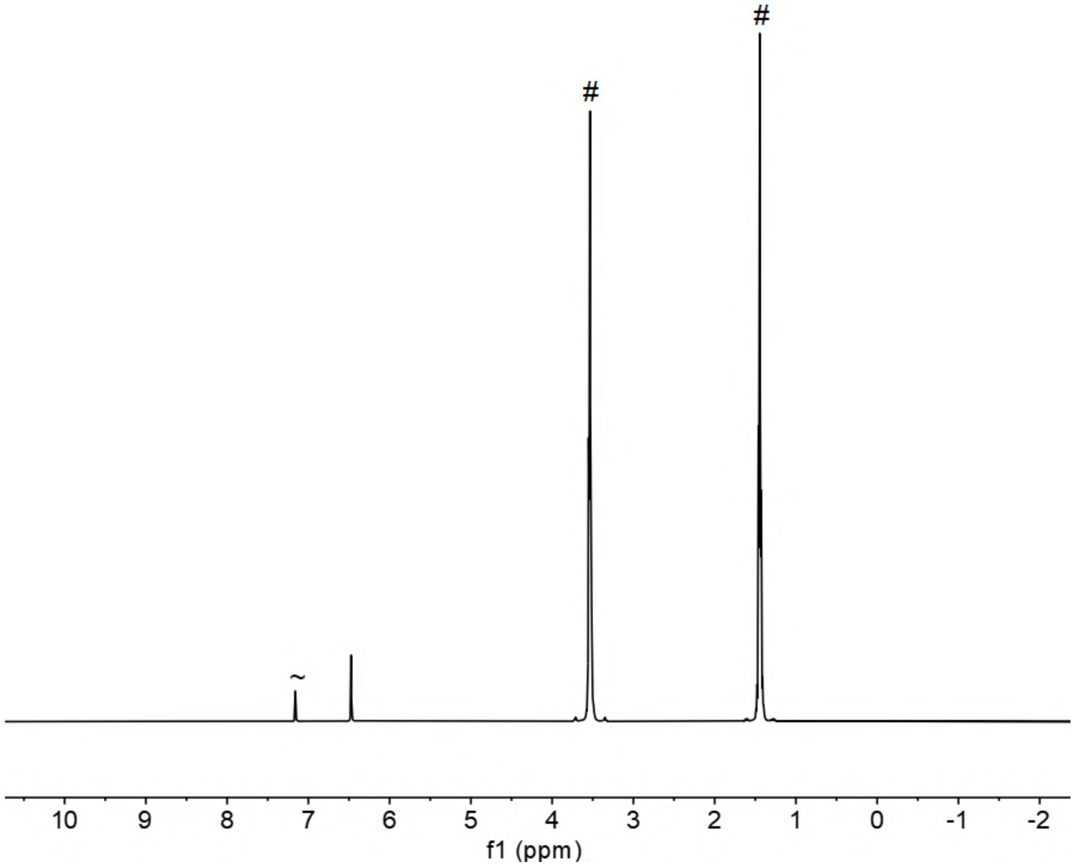

Supplementary Figure 10. ^{1}H NMR (C_6D_6/THF, 298 K) spectrum of $C_8H_8K_2$. # = THF, a drop of which was used to aid solubility; ~ = C_6D_6 solvent.

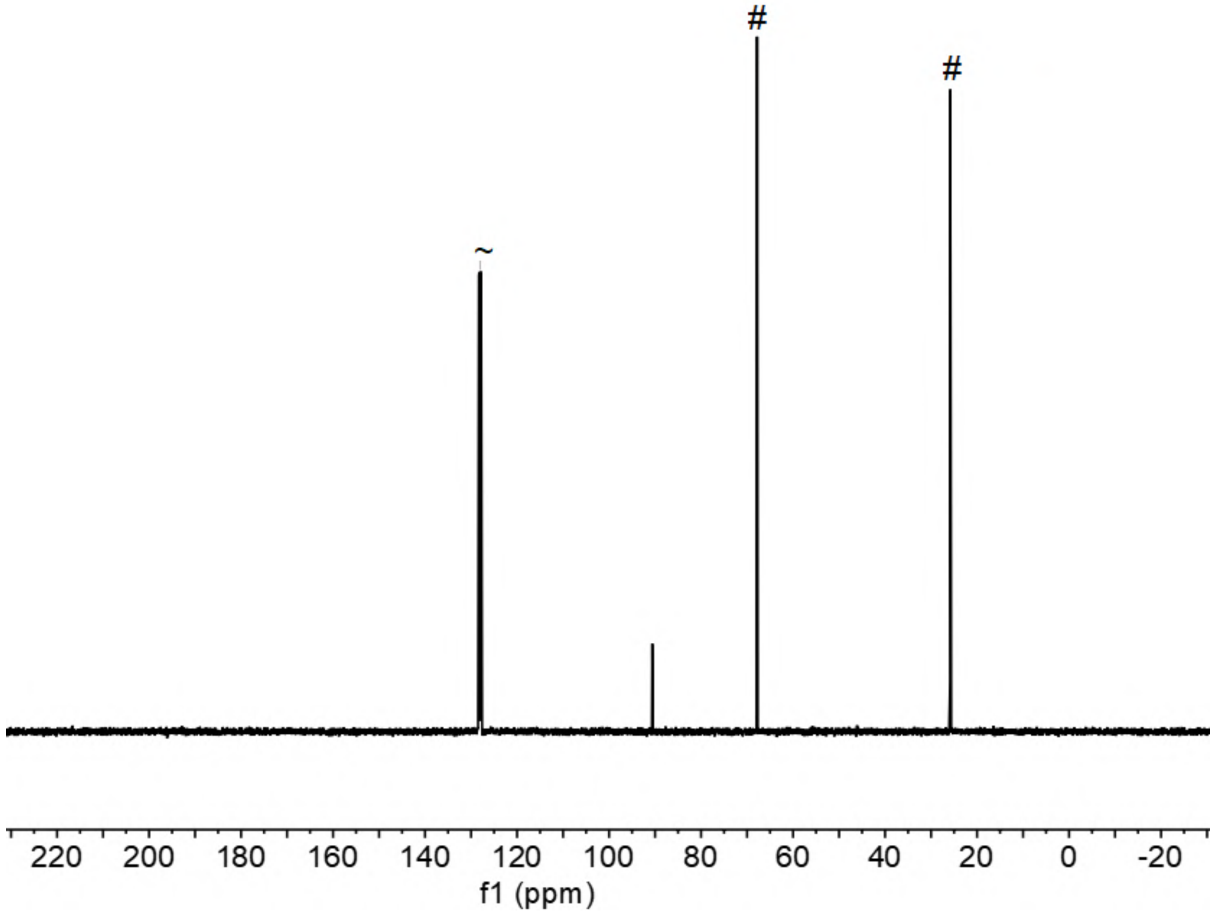

Supplementary Figure 11. $^{13}C\{^{1}H\}$ NMR (C_6D_6/THF, 298 K) spectrum of $C_8H_8K_2$. # = THF, a drop of which was used to aid solubility; ~ = C_6D_6 solvent.

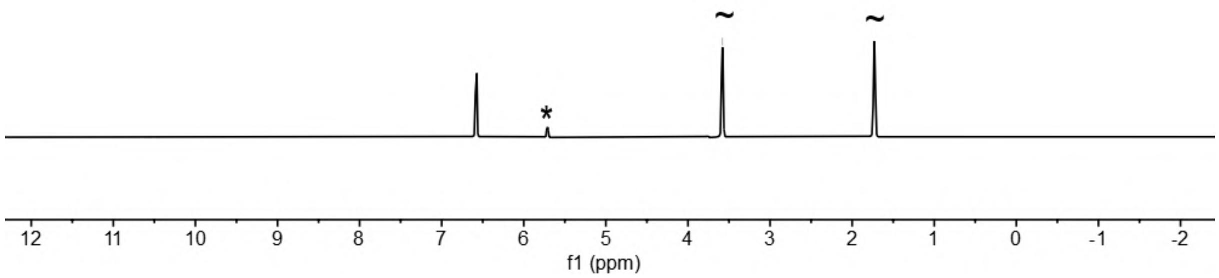

Supplementary Figure 12. ^{1}H NMR (D_8-THF, 298 K) spectrum of $[Th(\eta^{8}\text{-}C_8H_8)_2]$. The asterisk (*) at ~5.5 ppm denotes a trace impurity of C_8H_8. ~ = THF solvent.

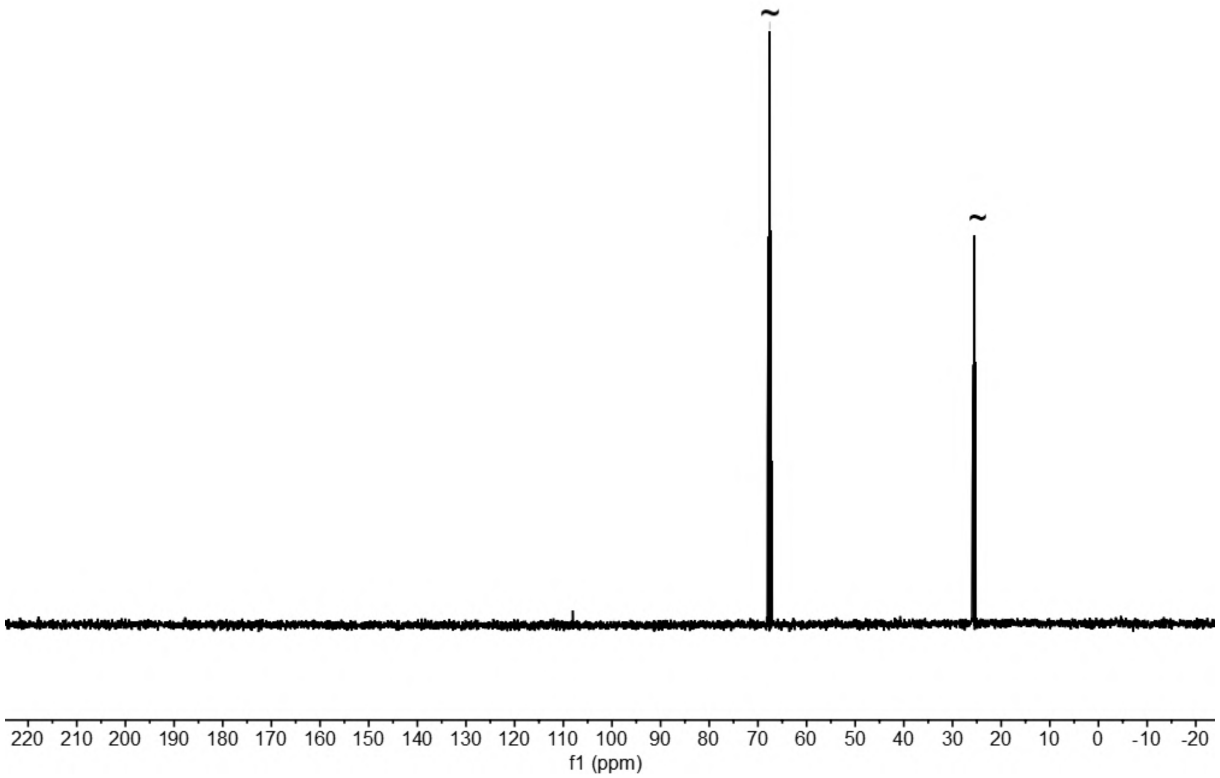

Supplementary Figure 13. $^{13}C\{^{1}H\}$ NMR (D_8-THF, 298 K) spectrum of [Th(η^8-C_8H_8)$_2$]. The weak nature of the signal arises from the poor solubility of [Th(η^8-C_8H_8)$_2$] even in d_8-THF. ~ = D_8-THF solvent.

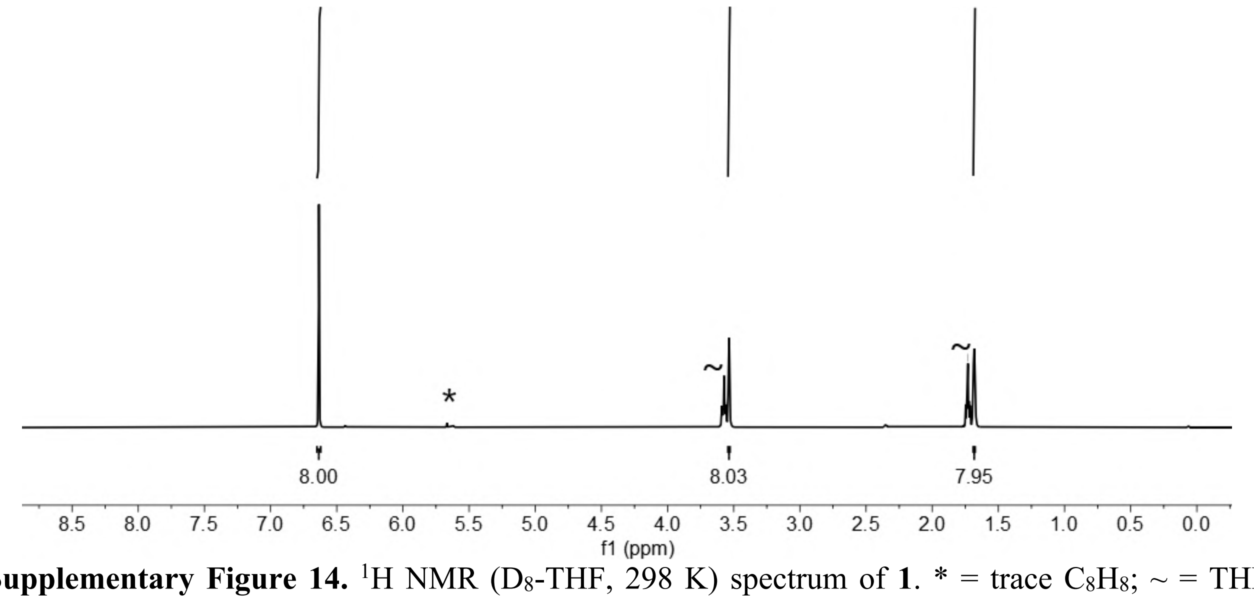

Supplementary Figure 14. ^{1}H NMR (D_8-THF, 298 K) spectrum of **1**. * = trace C_8H_8; ~ = THF solvent.

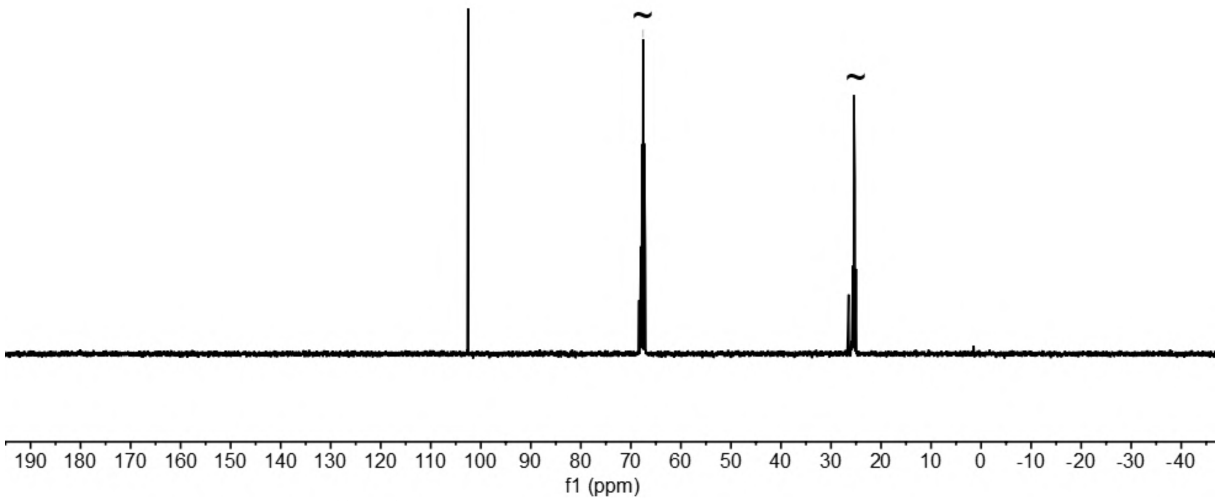

Supplementary Figure 15. $^{13}C\{^{1}H\}$ NMR (D_8-THF, 298 K) spectrum of **1**. ~ = D_8-THF solvent.

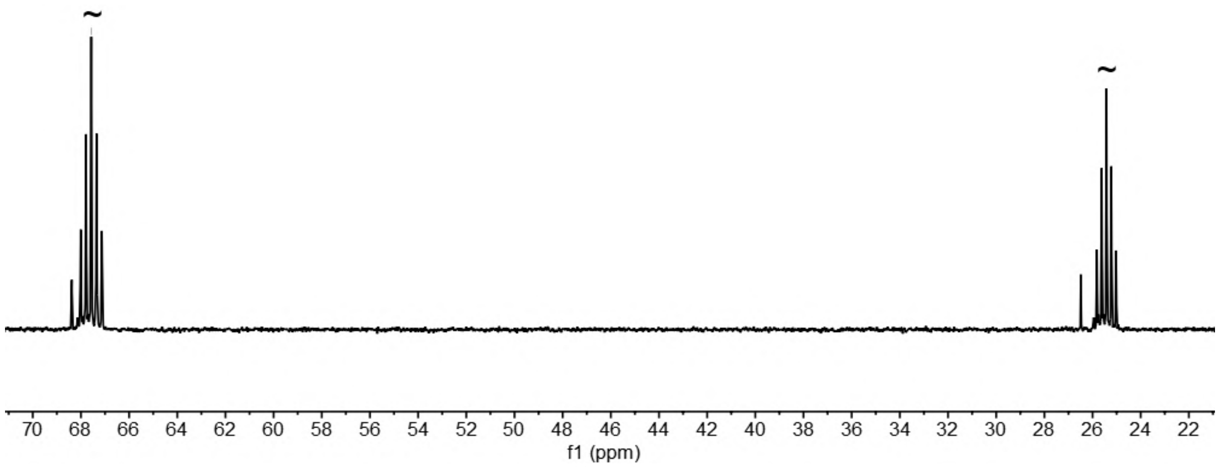

Supplementary Figure 16. Zoom-in of the $^{13}C\{^{1}H\}$ NMR (D_8-THF, 298 K) spectrum of **1** to highlight the small resonances at 68.39 and 26.53 ppm attributed to Th-coordinated THF. ~ = D_8-THF solvent.

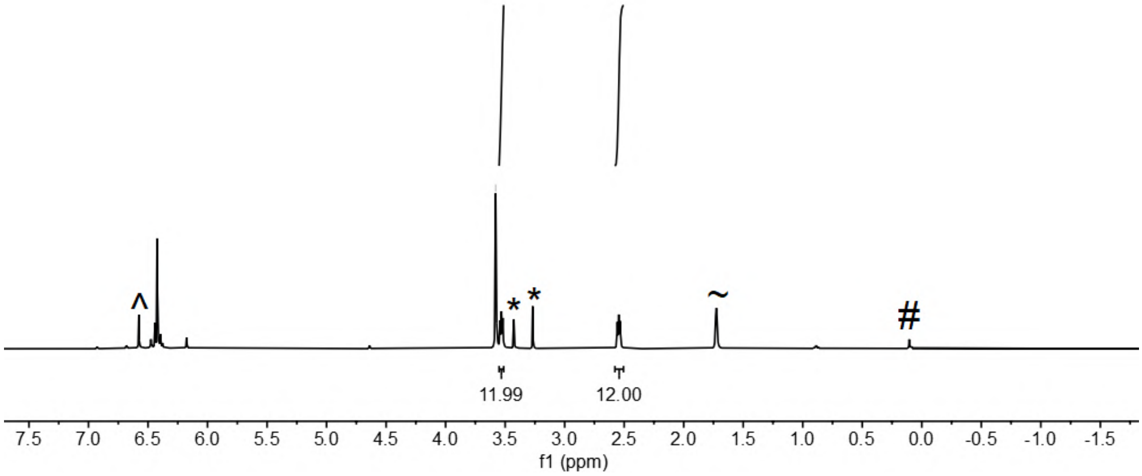

Supplementary Figure 17. Zoom-in of the ^{1}H NMR (D_8-THF, 298 K) spectrum of **4K**. # = silicone grease; ~ = THF solvent; * = trace DME; ^ = trace **1**.

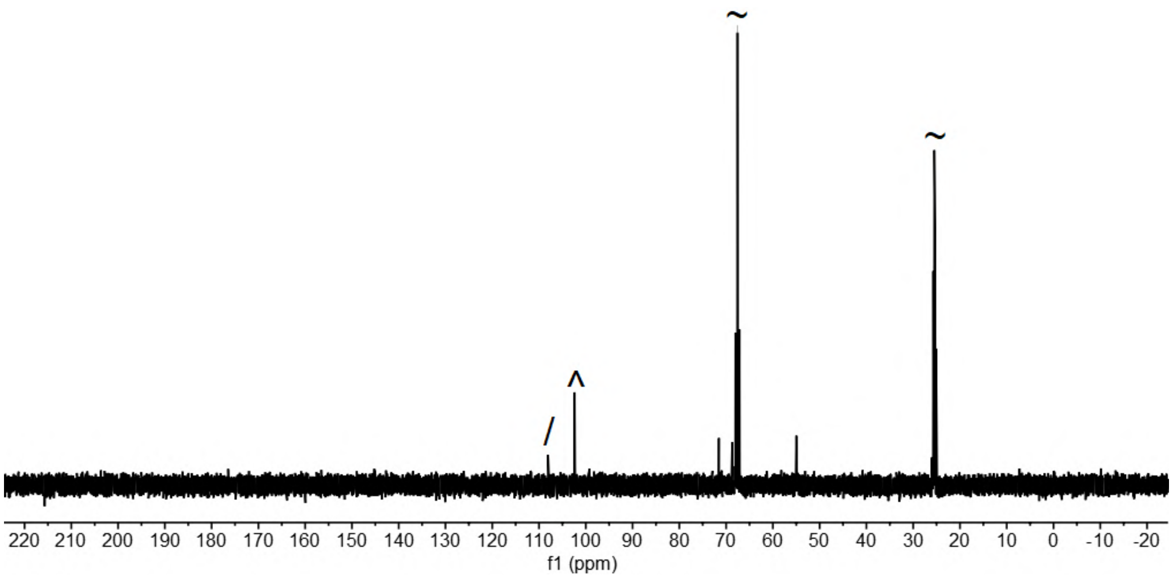

Supplementary Figure 18. $^{13}C\{^{1}H\}$ NMR (D_8-THF, 298 K) spectrum of **4K**. ~ = D_8-THF solvent; ^ = trace **1**; / = trace $[Th(\eta^8\text{-}C_8H_8)_2]$.

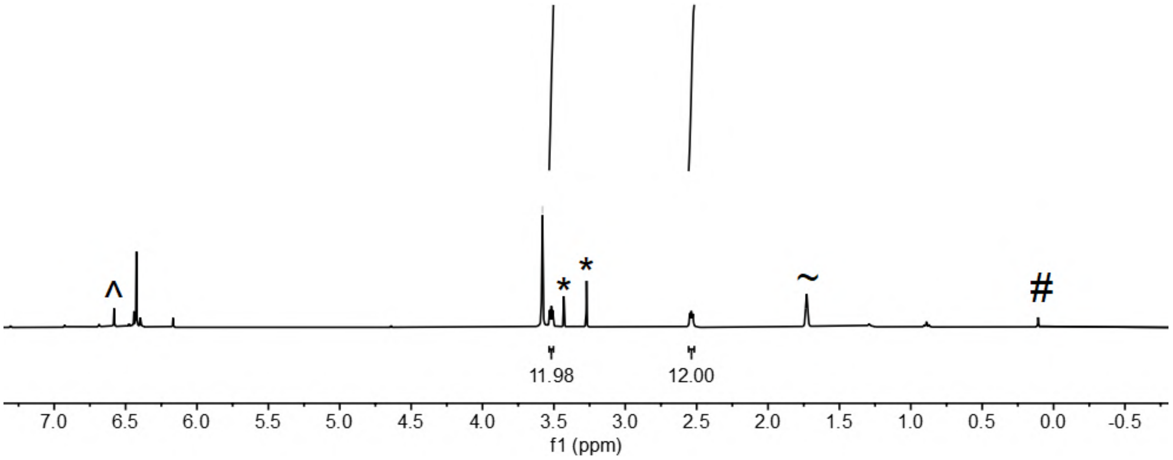

Supplementary Figure 19. Zoom-in of the ^{1}H NMR (D_8-THF, 298 K) spectrum of **4Rb**. # = silicone grease; ~ = THF solvent; * = trace DME; ^ = trace **1**.

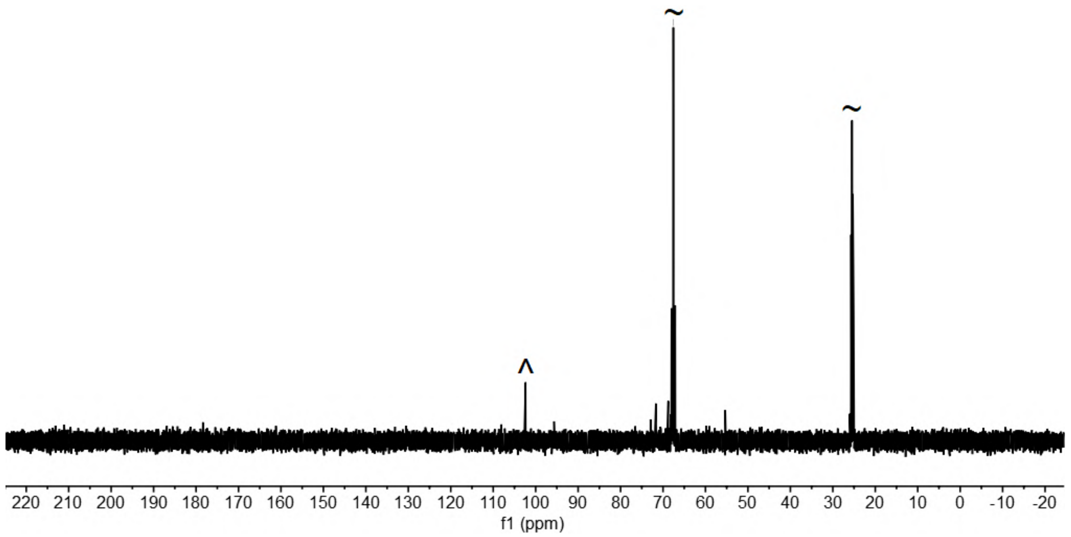

Supplementary Figure 20. $^{13}C\{^{1}H\}$ NMR (D_8-THF, 298 K) spectrum of **4Rb**. ~ = D_8-THF solvent; ^ = trace **1**; / = trace $[Th(\eta^8\text{-}C_8H_8)_2]$.

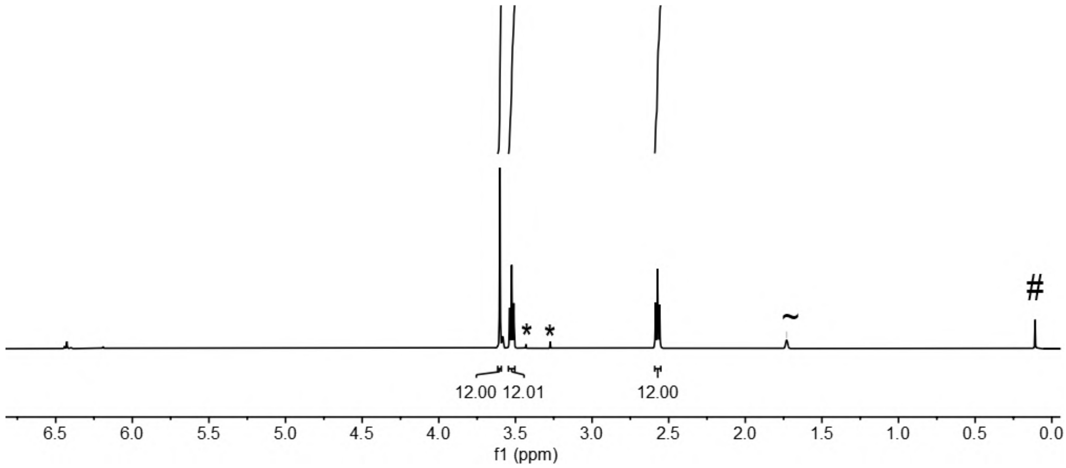

Supplementary Figure 21. Zoom-in of the ^{1}H NMR (D_8-THF, 298 K) spectrum of **4Cs**. # = silicone grease; ~ = THF solvent; * = trace DME.

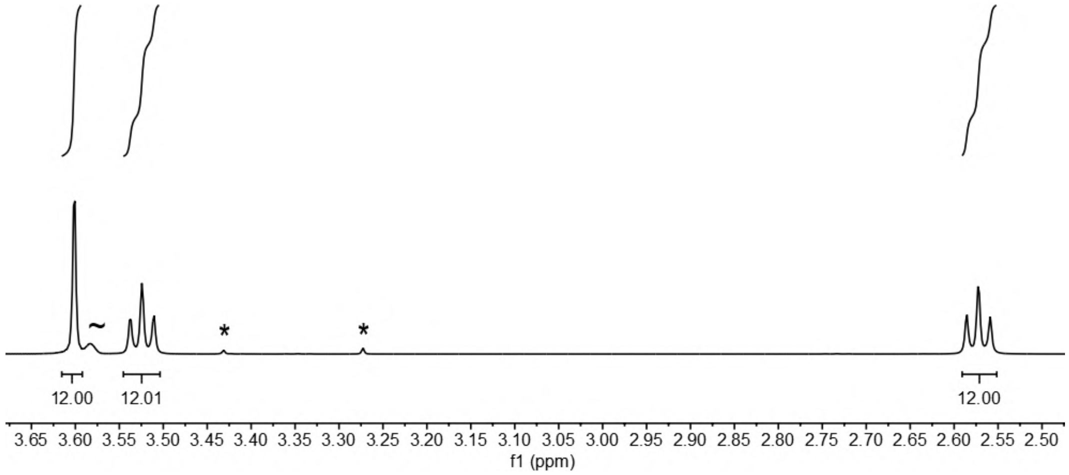

Supplementary Figure 22. Zoom-in of ^{1}H NMR (D_8-THF, 298 K) spectrum of **4Cs**. ~ = THF solvent; * = trace DME.

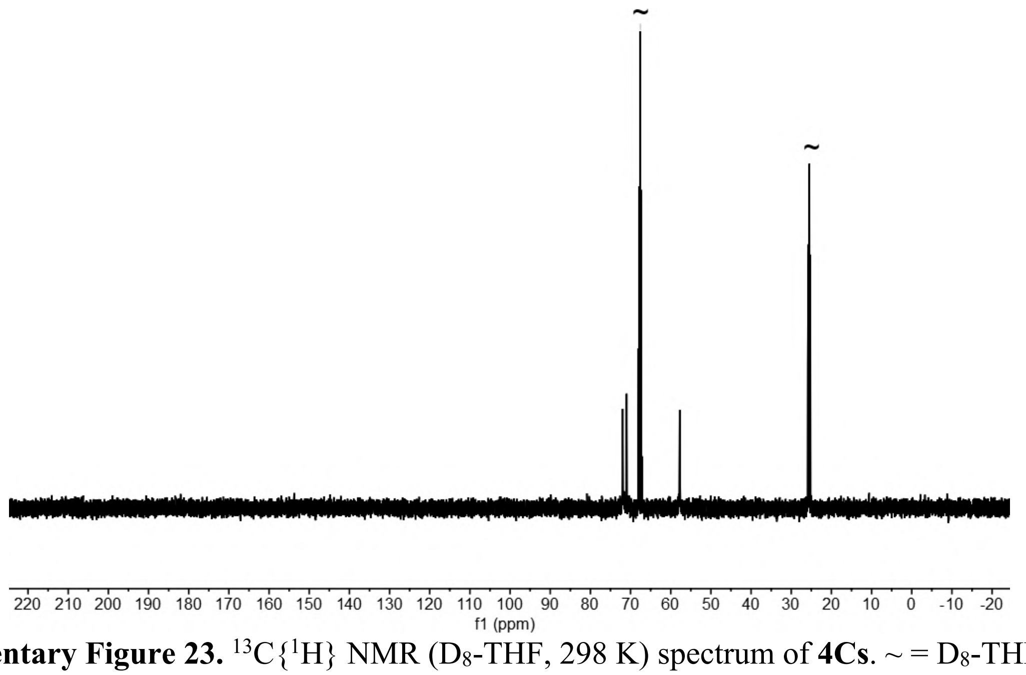

Supplementary Figure 23. $^{13}C\{^{1}H\}$ NMR (D_8-THF, 298 K) spectrum of **4Cs**. ~ = D_8-THF solvent.

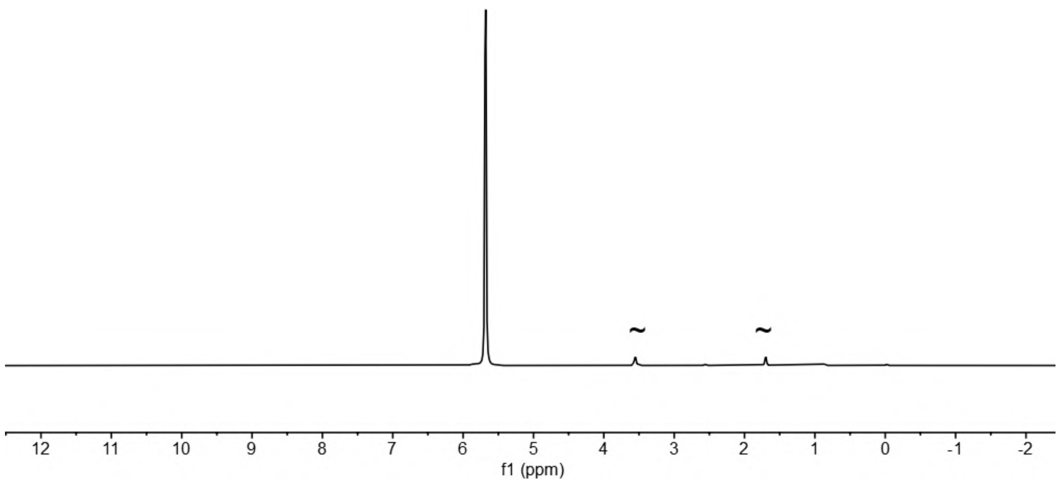

Supplementary Figure 24. ^{1}H NMR (D_8-THF, 298 K) spectrum of the reaction of **4Cs** with excess 1,3,5,7-cyclooctatetraene. ~ = THF.

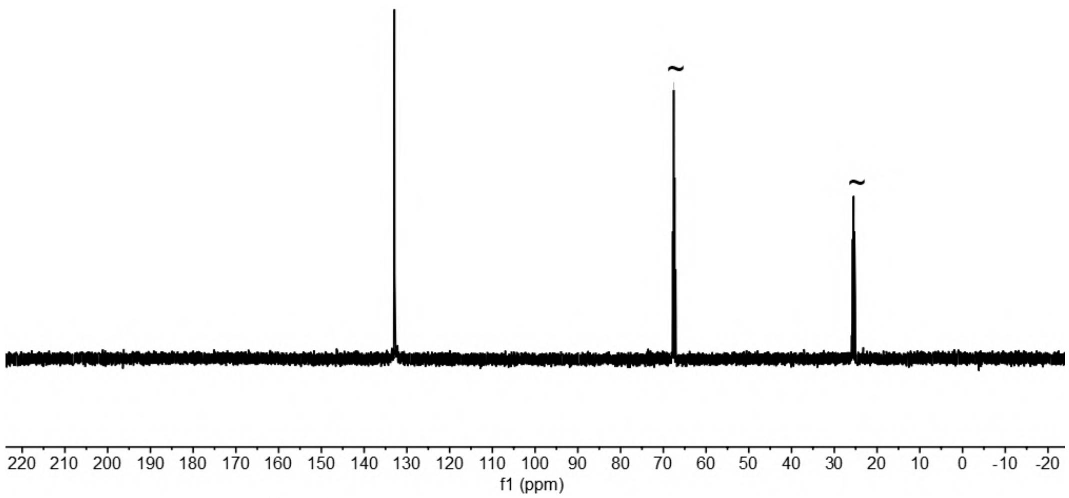

Supplementary Figure 25. $^{13}C\{^{1}H\}$ NMR (D_8-THF, 298 K) spectrum of the reaction of **4Cs** with excess 1,3,5,7-cyclooctatetraene. ~ = D_8-THF solvent.

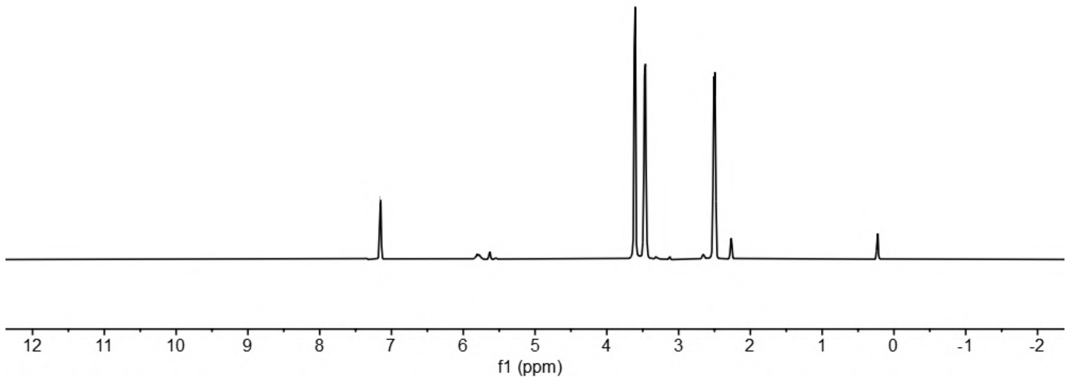

Supplementary Figure 26. ^{1}H NMR (D_6-benzene, 298 K) spectrum of the mother liquor following the reaction of **4Cs** with CCl_4.

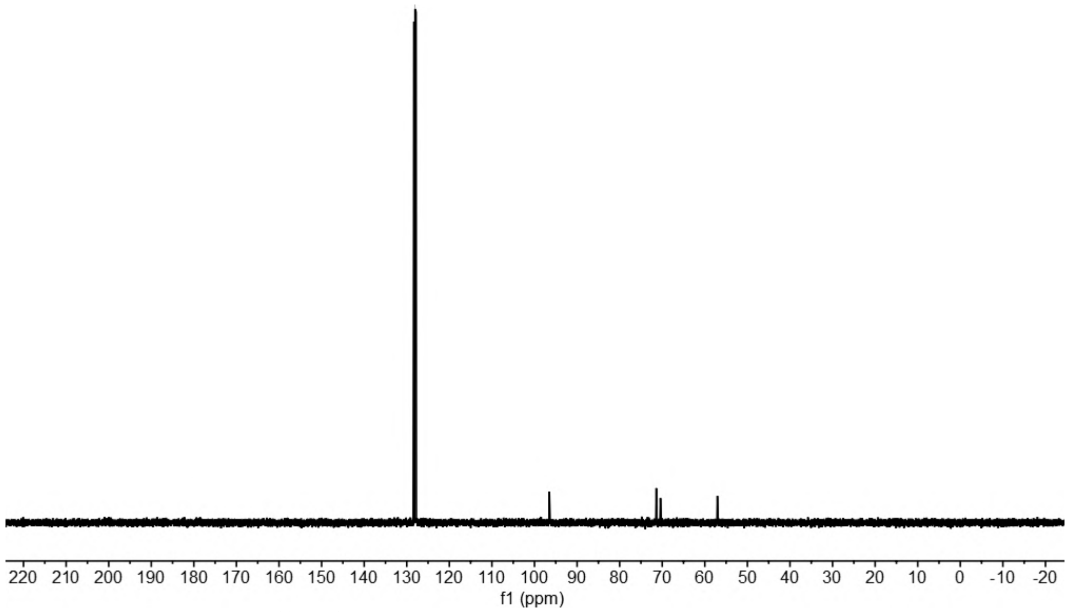

Supplementary Figure 27. $^{13}C\{^1H\}$ NMR (D_6-benzene, 298 K) spectrum of the mother liquor following the reaction of **4Cs** with CCl_4.

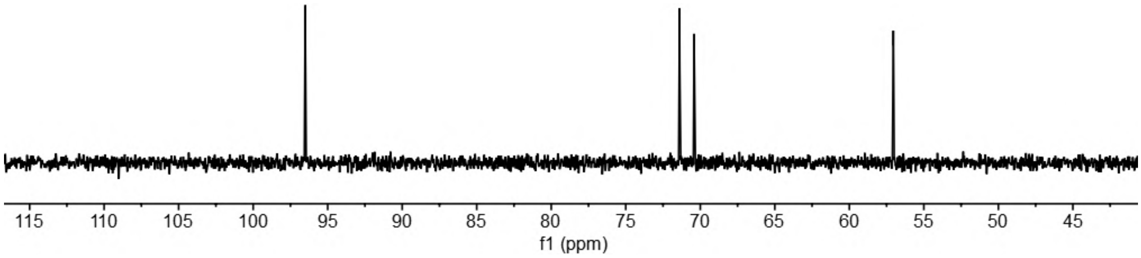

Supplementary Figure 28. Zoom-in of $^{13}C\{^1H\}$ NMR (D_6-benzene, 298 K) spectrum of the mother liquor following the reaction of **4Cs** with CCl_4.

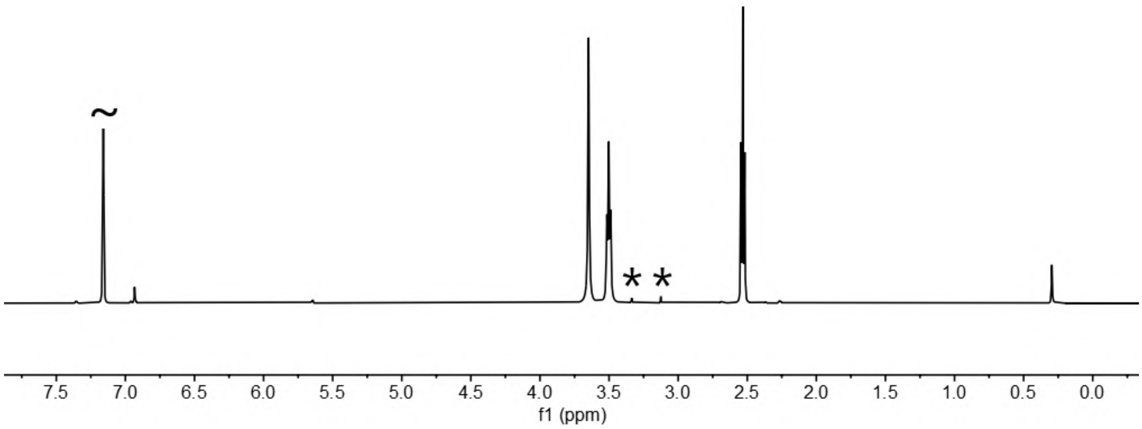

Supplementary Figure 29. ^1H NMR (D_6-benzene, 298 K) spectrum of the mother liquor following the reaction of solid **4Cs** with CO_2. ~ = D_6-benzene solvent; * = trace DME.

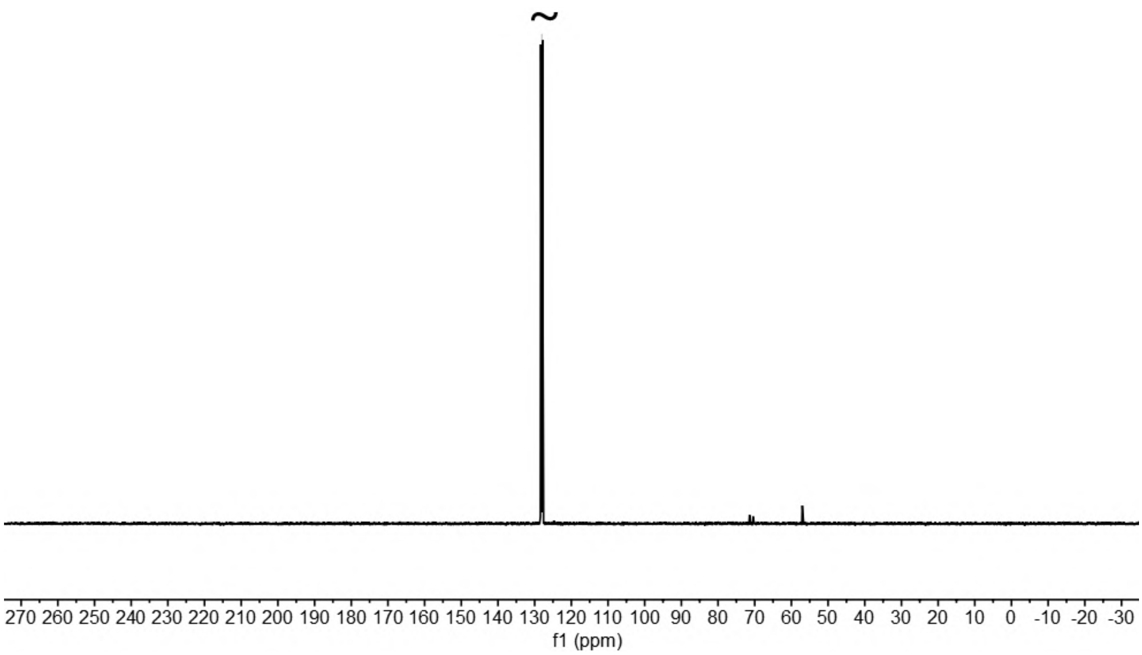

Supplementary Figure 30. $^{13}C\{^1H\}$ NMR (D_6-benzene, 298 K) spectrum of the mother liquor following the reaction of solid **4Cs** with CO_2. ~ = D_6-benzene solvent.

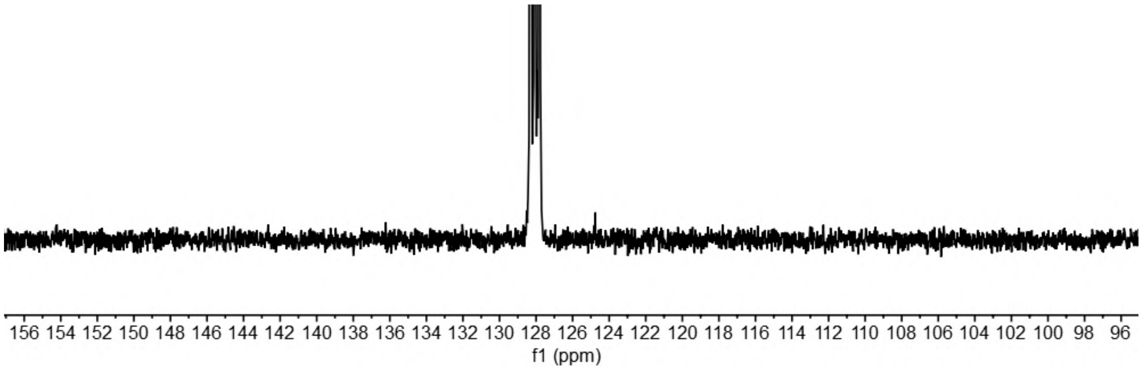

Supplementary Figure 31. Zoom-in of $^{13}C\{^1H\}$ NMR (D_6-benzene, 298 K) spectrum of the mother liquor following the reaction of solid **4Cs** with CO_2 to highlight signal at 124.75 ppm attributed to CO_2.

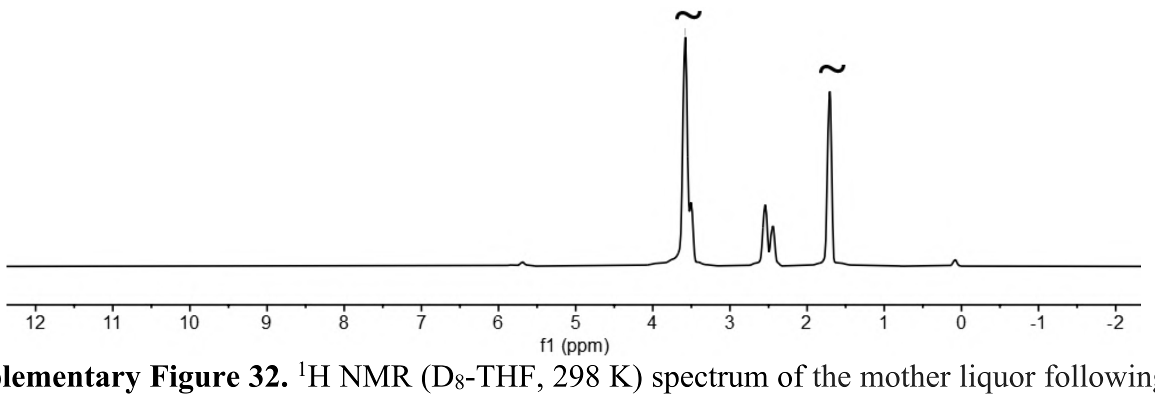

Supplementary Figure 32. ^1H NMR (D_8-THF, 298 K) spectrum of the mother liquor following the reaction of **4Cs** with CO_2. ~ = D_8-THF solvent.

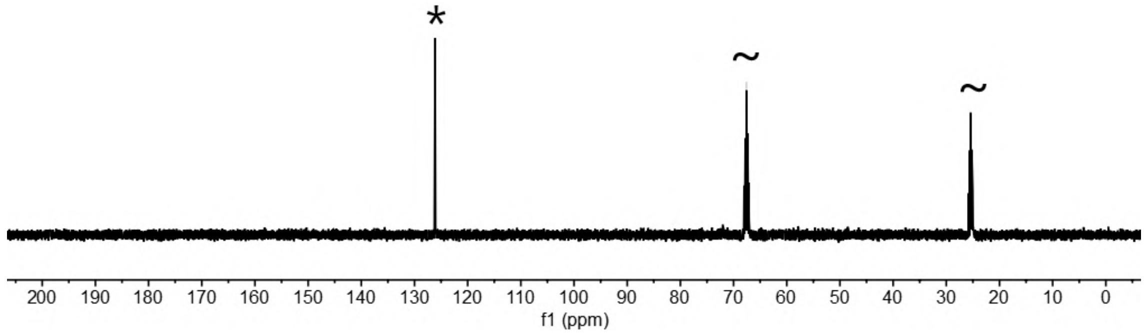

Supplementary Figure 33. $^{13}C\{^{1}H\}$ NMR (D_8-THF, 298 K) spectrum of the mother liquor following the reaction of **4Cs** with CO_2. ~ = D_8-THF solvent; * = excess CO_2.

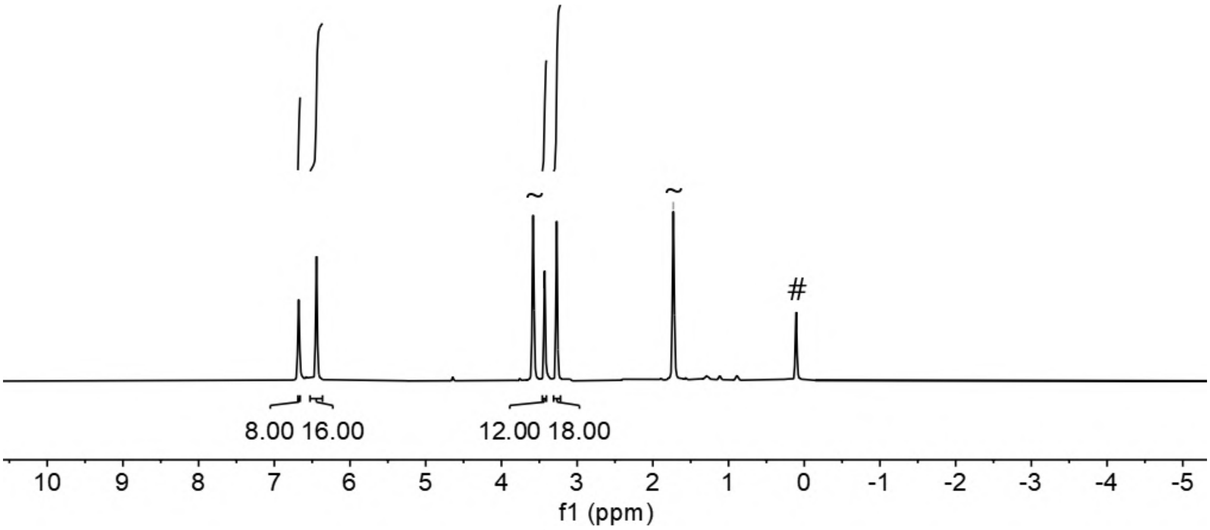

Supplementary Figure 34. Zoom-in of the ^{1}H NMR (D_8-THF, 298 K) spectrum of **5**. # = silicone grease; ~ = D_8-THF solvent.

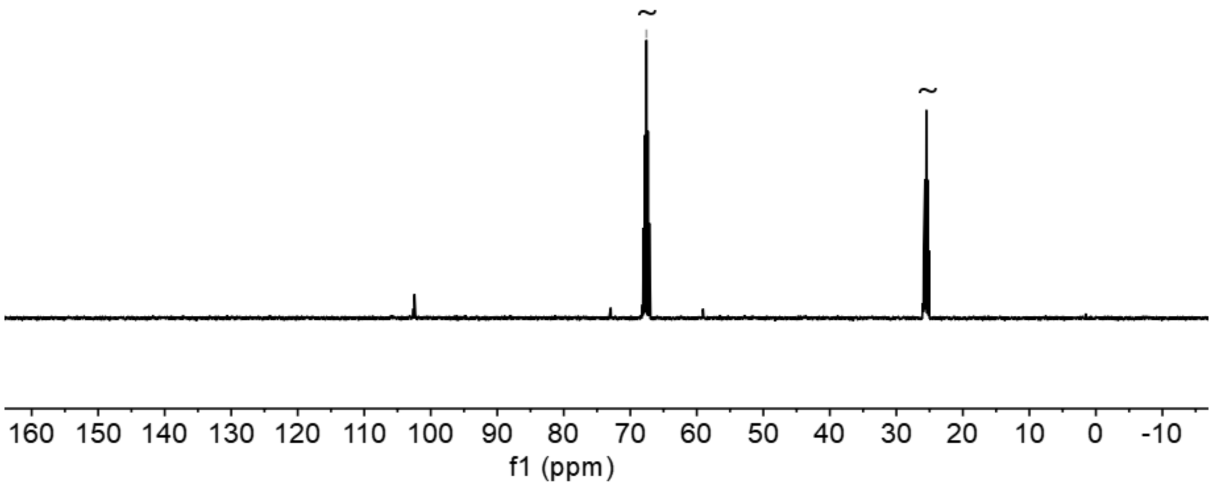

Supplementary Figure 35. $^{13}C\{^{1}H\}$ NMR (D_8-THF, 298 K) spectrum of **5**. ~ = D_8-THF solvent.

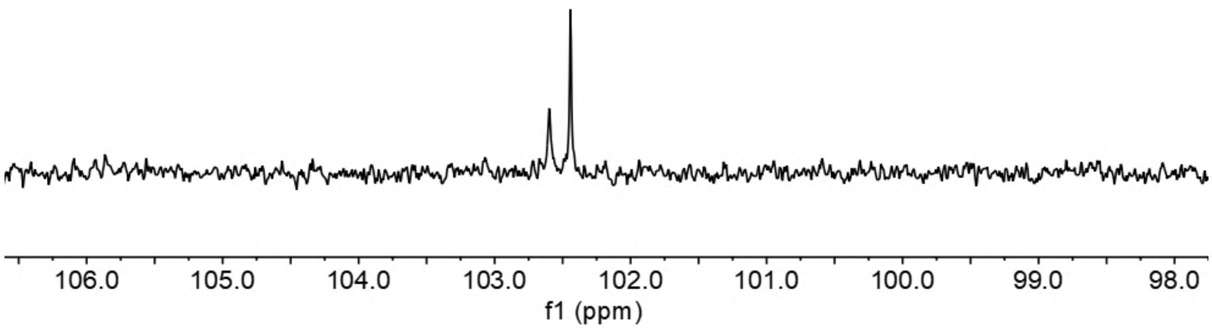

Supplementary Figure 36. Zoom-in of the $^{13}C\{^1H\}$ NMR (D_8-THF, 298 K) spectrum of **5**.

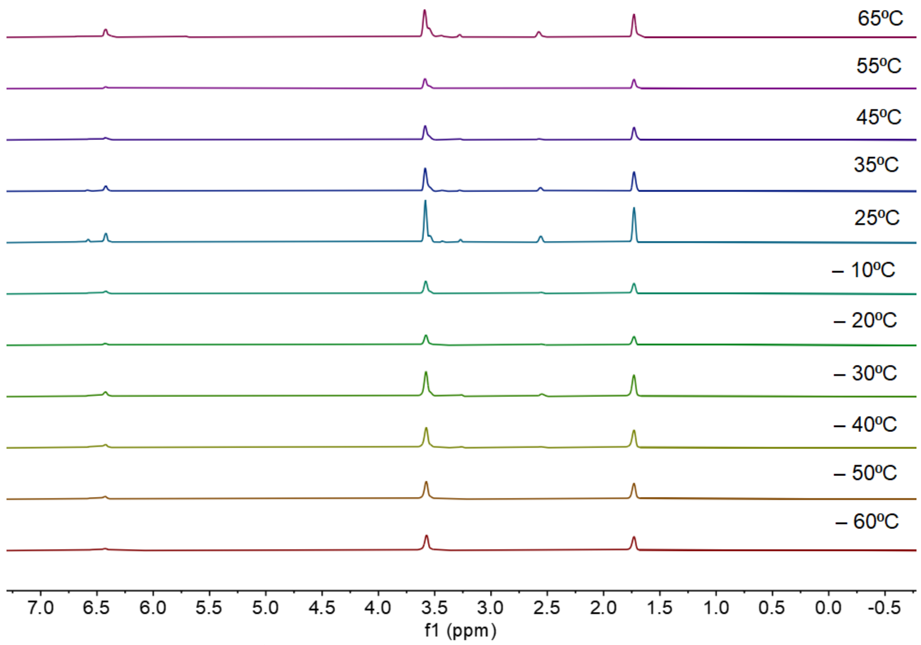

Supplementary Figure 37. VT ^1H NMR (D_8-THF, 298 K) spectrum of **4K** ranging from –60 (bottom) to +65 °C (top). Measurements were conducted in the following order: 25, –10, –20, –30, –40, –50, –60, 25, 35, 45, 55, and 65 °C. The sample was held at each temperature for 10 minutes to account for slow thermal equilibration of the sample. A low-temperature NMR thermometer determined the temperatures within the sample during VT-NMR measurement and these are listed under salient NMR details.

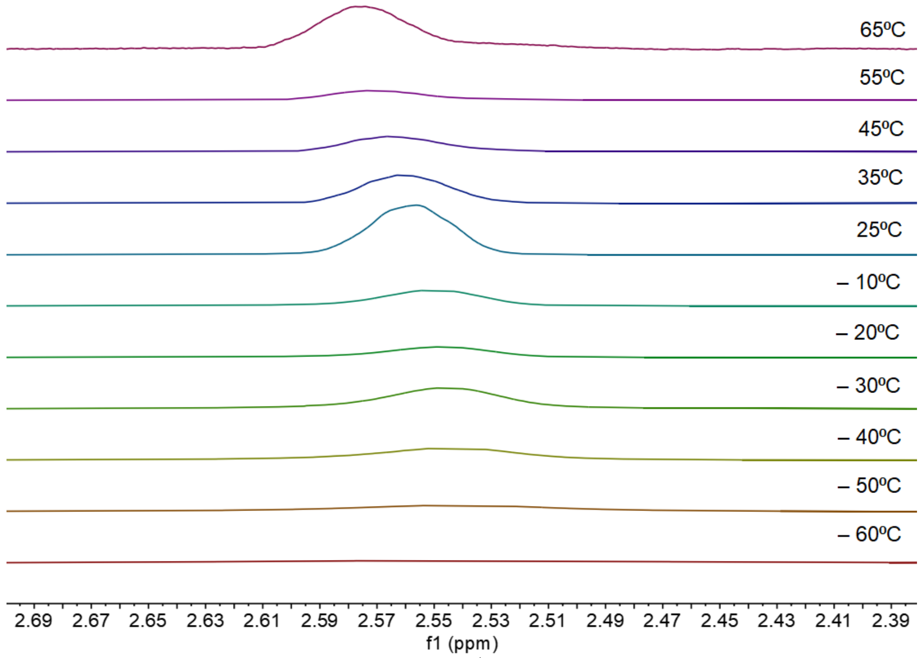

Supplementary Figure 38. Zoom-in of the VT ^{1}H NMR (D_8-THF, 298 K) spectrum of **4K** to highlight changes in the resonance at ~2.55 ppm associated with the $[M(2.2.2\text{-cryptand})]^+$ cation component. Measurements were conducted in the following order: 25, –10, –20, –30, –40, –50, –60, 25, 35, 45, 55, and 65 °C. The sample was held at each temperature for 10 minutes to account for slow thermal equilibration of the sample.

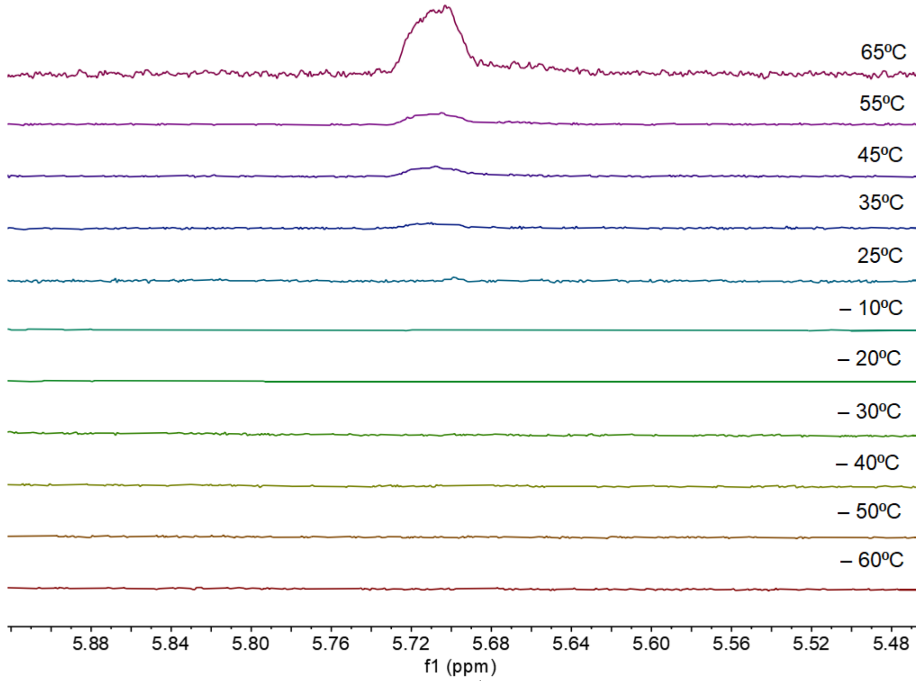

Supplementary Figure 39. Zoom-in of the VT ^{1}H NMR (D_8-THF, 298 K) spectrum of **4K** to highlight changes in the resonance associated with 1,3,5,7-cyclooctatetraene (~5.70 ppm). Measurements were conducted in the following order: 25, –10, –20, –30, –40, –50, –60, 25, 35, 45, 55, and 65 °C. The sample was held at each temperature for 10 minutes to account for slow thermal equilibration of the sample.

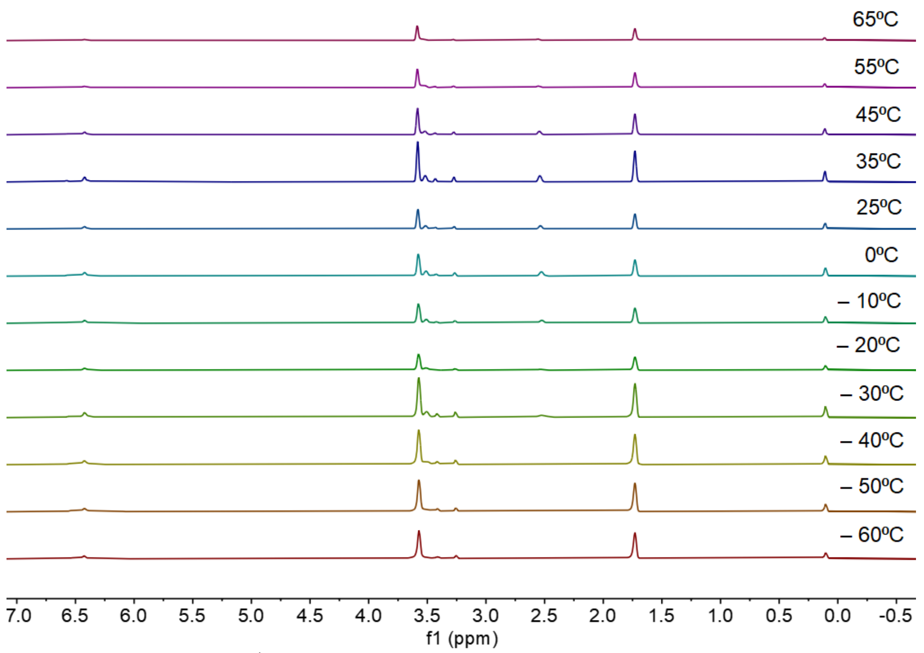

Supplementary Figure 40. VT ^{1}H NMR (D_8-THF, 298 K) spectrum of **4Rb** ranging from –60 (bottom) to +65 °C (top). Measurements were conducted in the following order: 25, 0, –10, –20, –30, –40, –50, –60, 25, 35, 45, 55, and 65 °C. The sample was held at each temperature for 10 minutes to account for slow thermal equilibration of the sample. A low-temperature NMR thermometer determined the temperatures within the sample during VT-NMR measurement and these are listed under salient NMR details.

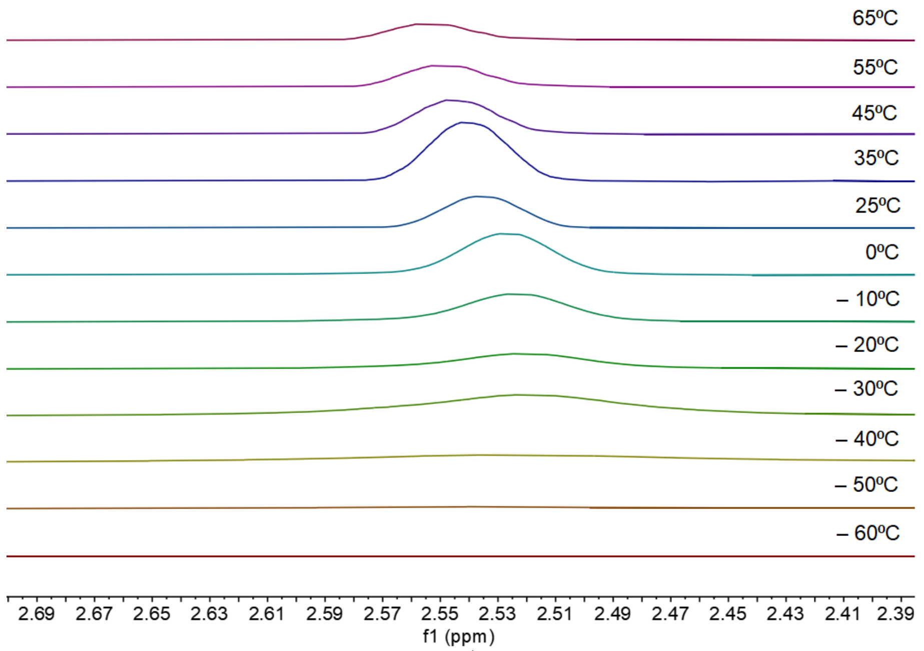

Supplementary Figure 41. Zoom-in of the VT ^{1}H NMR (D_8-THF, 298 K) spectrum of **4Rb** to highlight changes in resonance at ~2.55 ppm associated with the [M(2.2.2-cryptand)]$^{+}$ cation component. Measurements were conducted in the following order: 25, 0, –10, –20, –30, –40, –50, –60, 25, 35, 45, 55, and 65 °C. The sample was held at each temperature for 10 minutes to account for slow thermal equilibration of the sample.

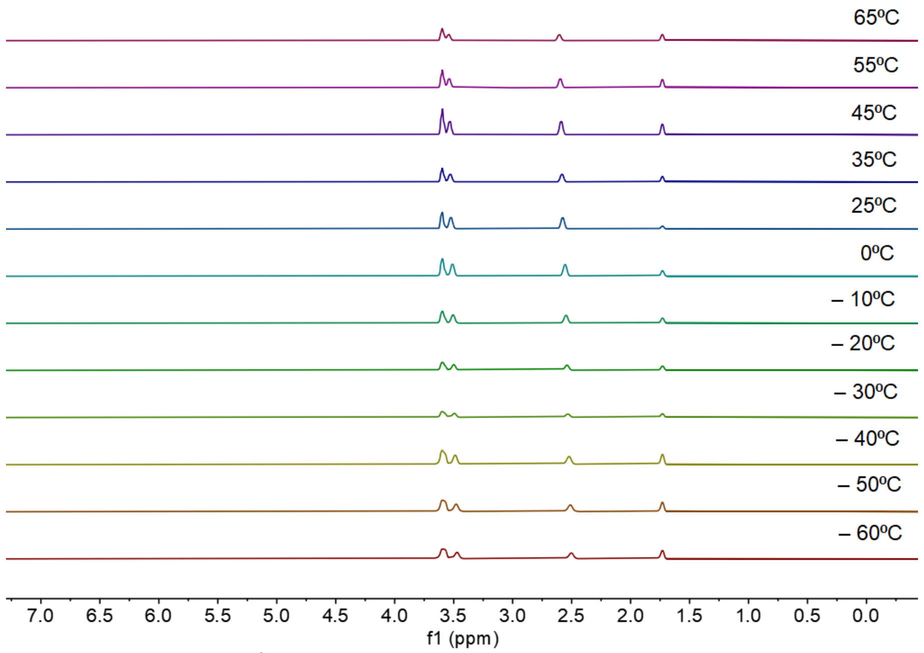

Supplementary Figure 42. VT ^{1}H NMR (D_8-THF, 298 K) spectrum of **4Cs** ranging from –60 (bottom) to +65 °C (top). Measurements were conducted in the following order: 25, 0, –10, –20, –30, –40, –50, –60, 25, 35, 45, 55, and 65 °C. The sample was held at each temperature for 10 minutes to account for slow thermal equilibration of the sample. Sample decomposed at –30 °C, which was after ~2 hours of measurement time. A low-temperature NMR thermometer determined the temperatures within the sample during VT-NMR measurement and these are listed under salient NMR details.

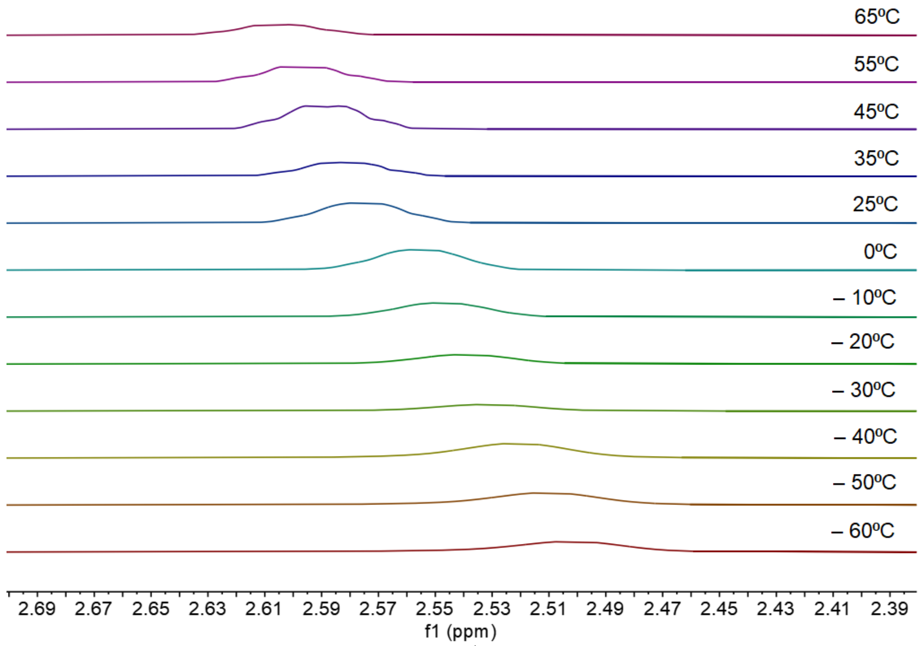

Supplementary Figure 43. Zoom-in of the VT ^{1}H NMR (D_8-THF, 298 K) spectrum of **4Cs** to highlight changes in resonance at ~2.55 ppm associated with the [M(2.2.2-cryptand)]$^{+}$ cation component. Measurements were conducted in the following order: 25, 0, –10, –20, –30, –40, –50, –60, 25, 35, 45, 55, and 65 °C. The sample was held at each temperature for 10 minutes to account for slow thermal equilibration of the sample.

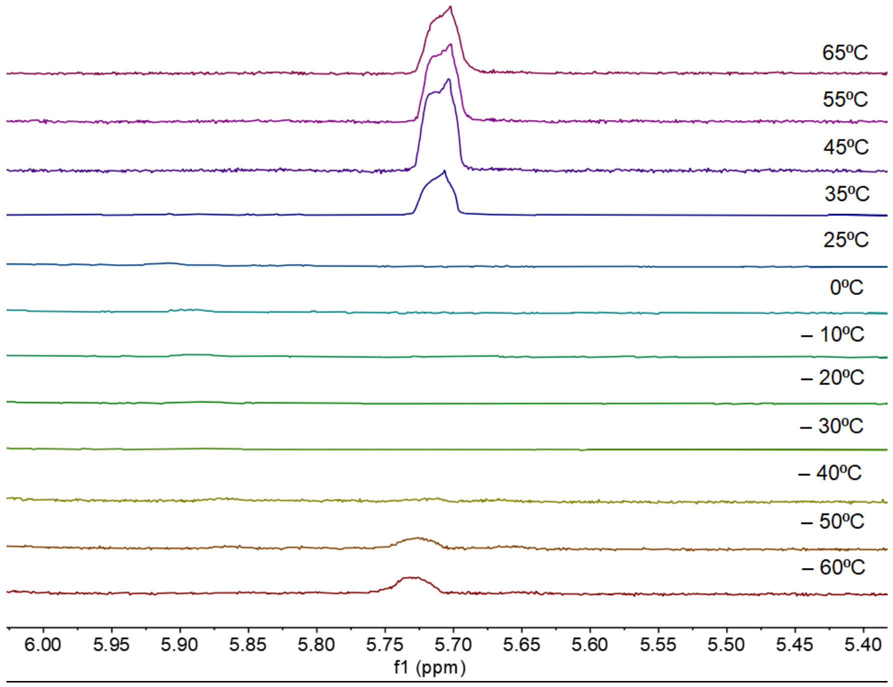

Supplementary Figure 44. Zoom-in of the VT ^{1}H NMR (D_8-THF, 298 K) spectrum of **4Cs** to highlight changes in the resonance associated with 1,3,5,7-cyclooctatetraene (~5.70 ppm). Measurements were conducted in the following order: 25, 0, –10, –20, –30, –40, –50, –60, 25, 35, 45, 55, and 65 °C. The sample was held at each temperature for 10 minutes to account for slow thermal equilibration of the sample.

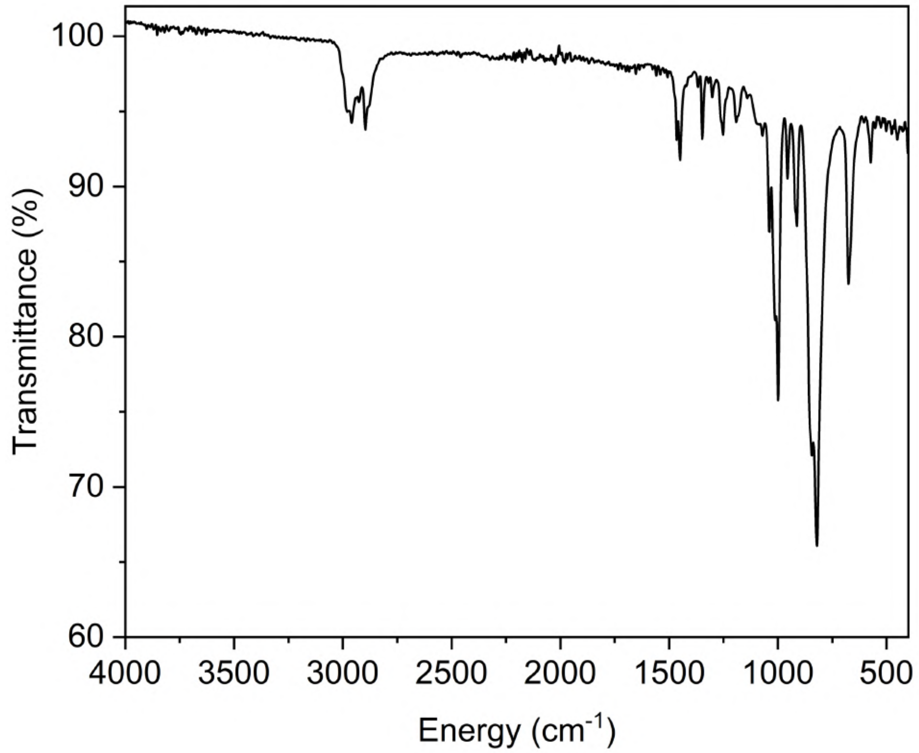

Supplementary Figure 45. ATR-IR spectrum of $ThCl_4(THF)_{3.5}$.

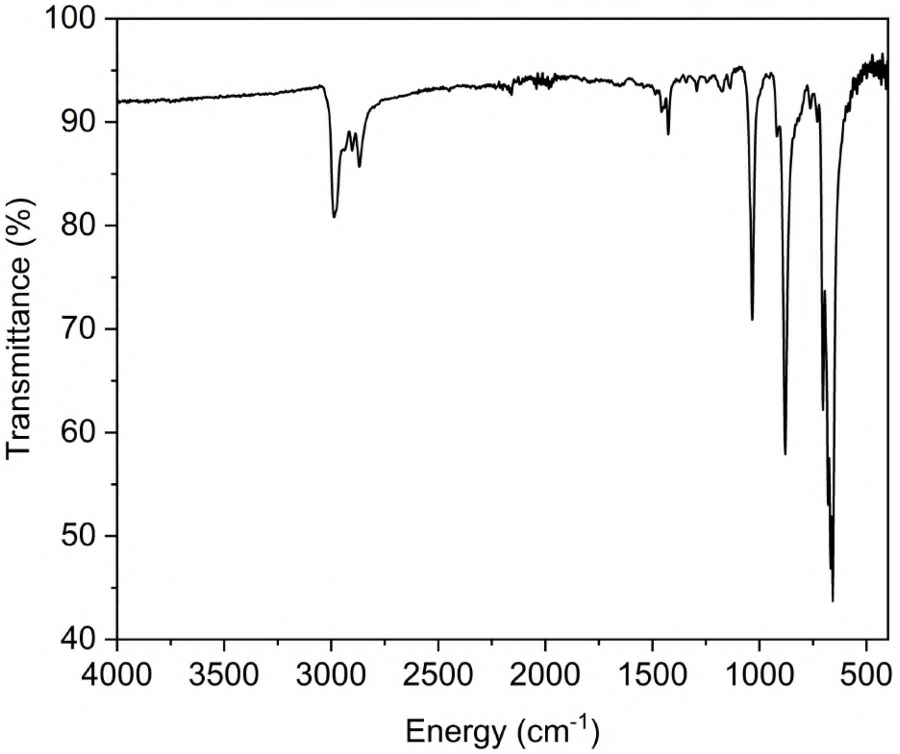

Supplementary Figure 46. ATR-IR spectrum of $C_8H_8K_2$.

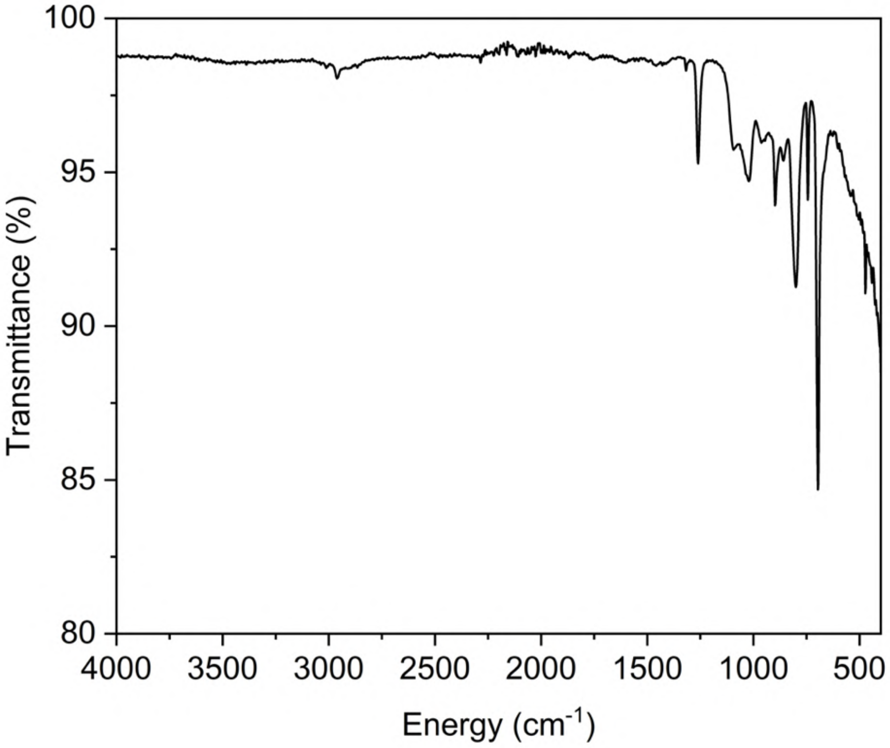

Supplementary Figure 47. ATR-IR spectrum of $[Th(\eta^8\text{-}C_8H_8)_2]$.

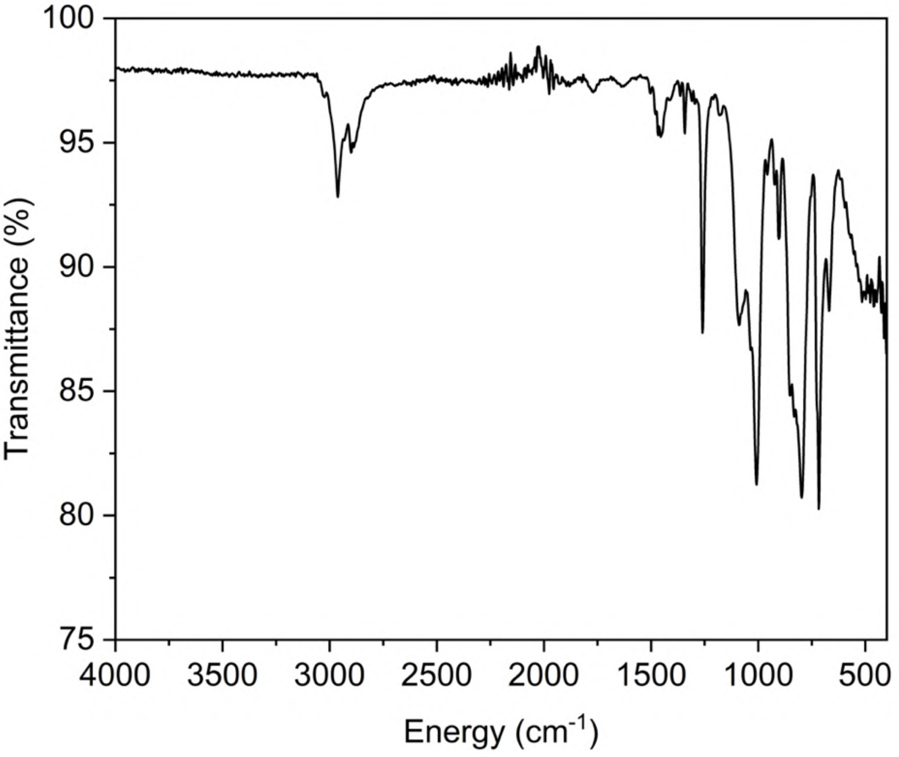

Supplementary Figure 48. ATR-IR spectrum of **1**.

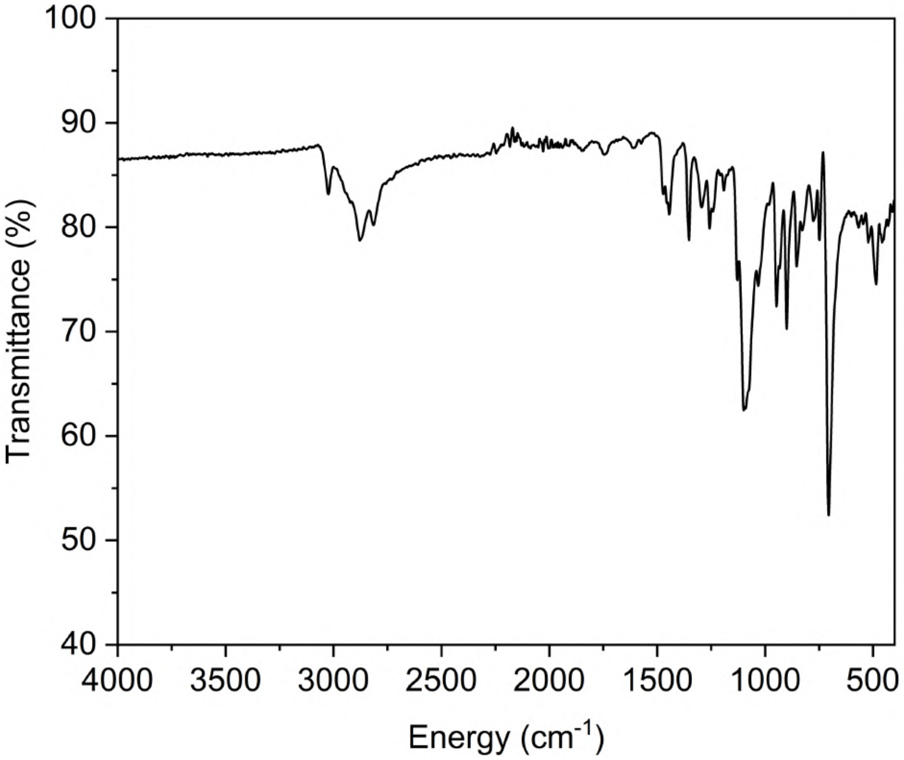

Supplementary Figure 49. ATR-IR spectrum of **4K**.

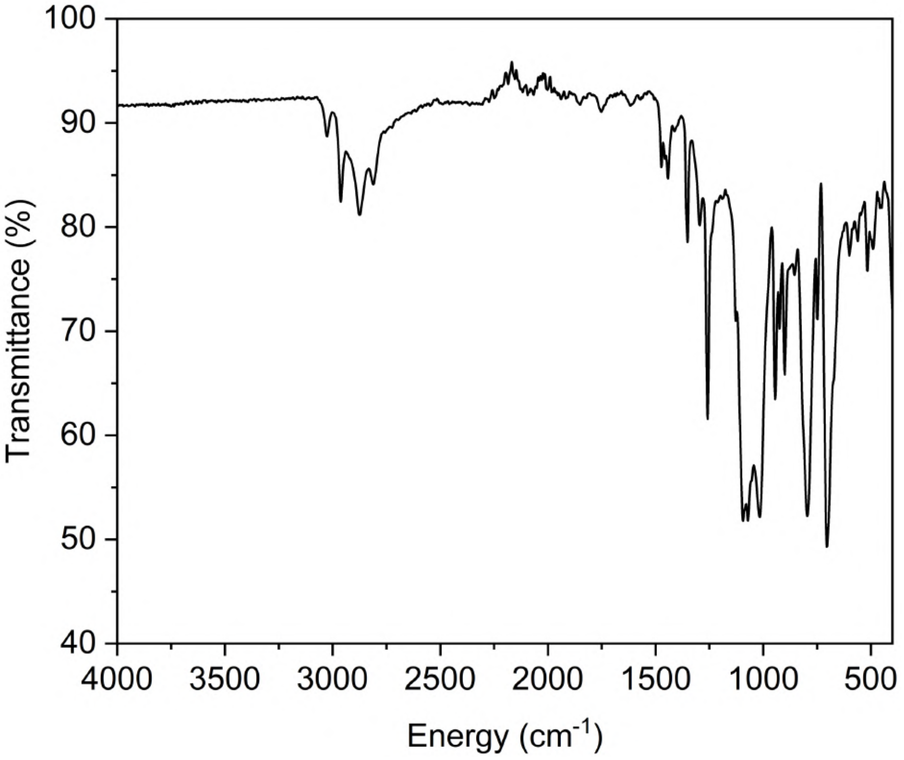

Supplementary Figure 50. ATR-IR spectrum of **4Rb**.

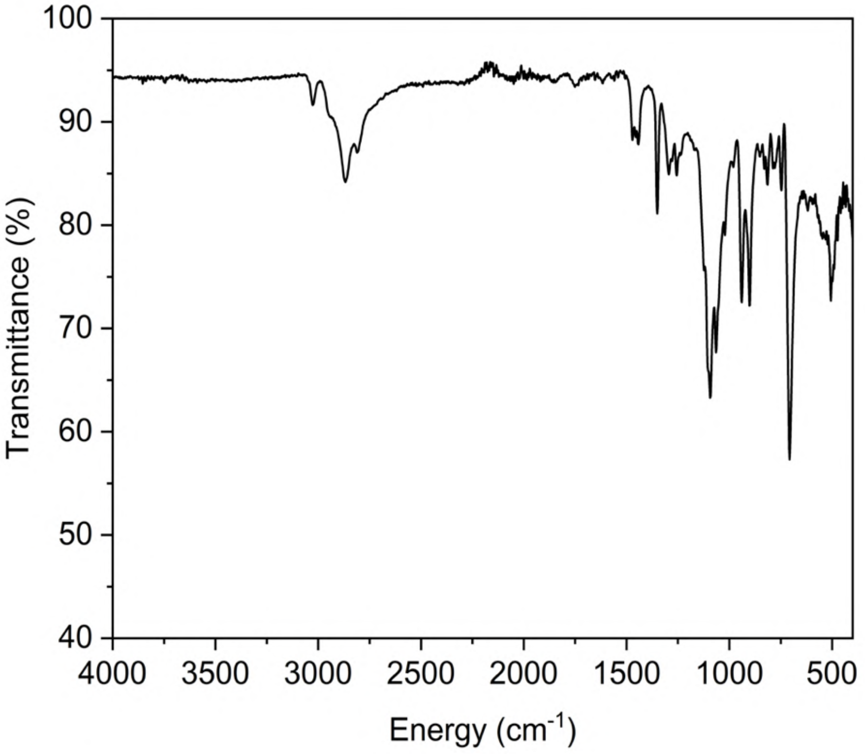

Supplementary Figure 51. ATR-IR spectrum of **4Cs**.

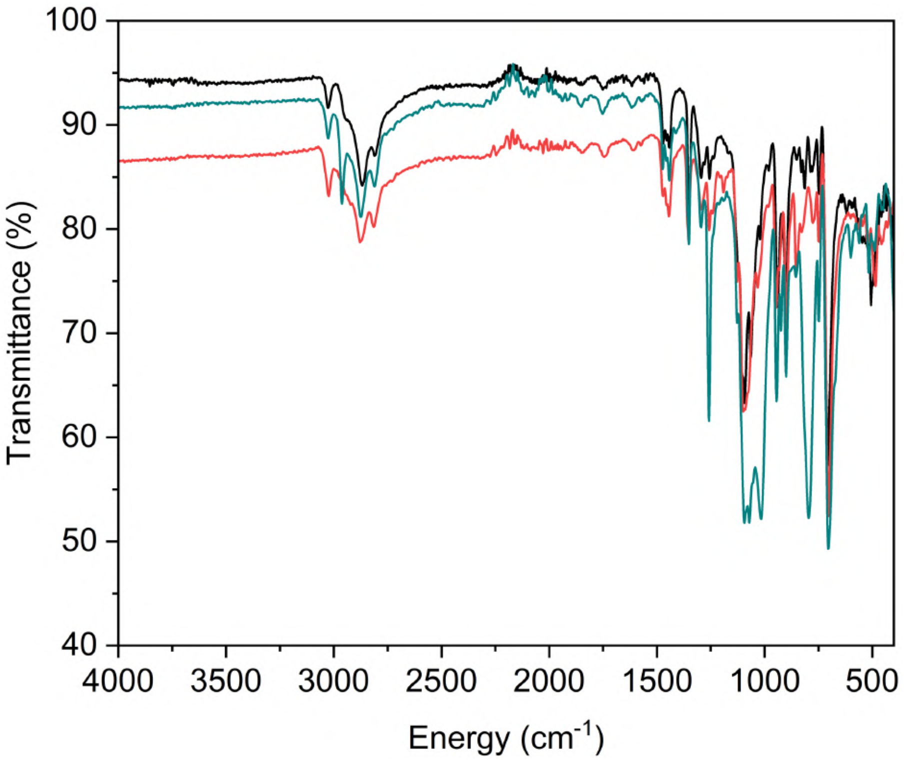

Supplementary Figure 52. Stacked ATR-IR spectra of **4K** (red line), **4Rb** (cyan line), and **4Cs** (black line).

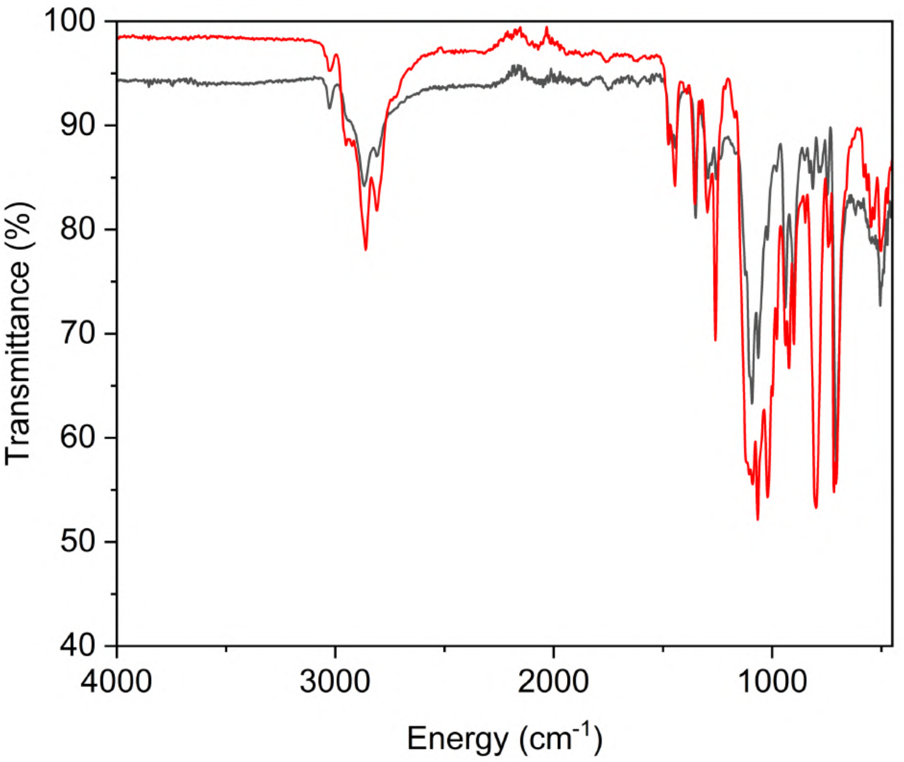

Supplementary Figure 53. Stacked ATR-IR spectra of **4Cs** prepared in protio (black) and deuterio solvents (red).

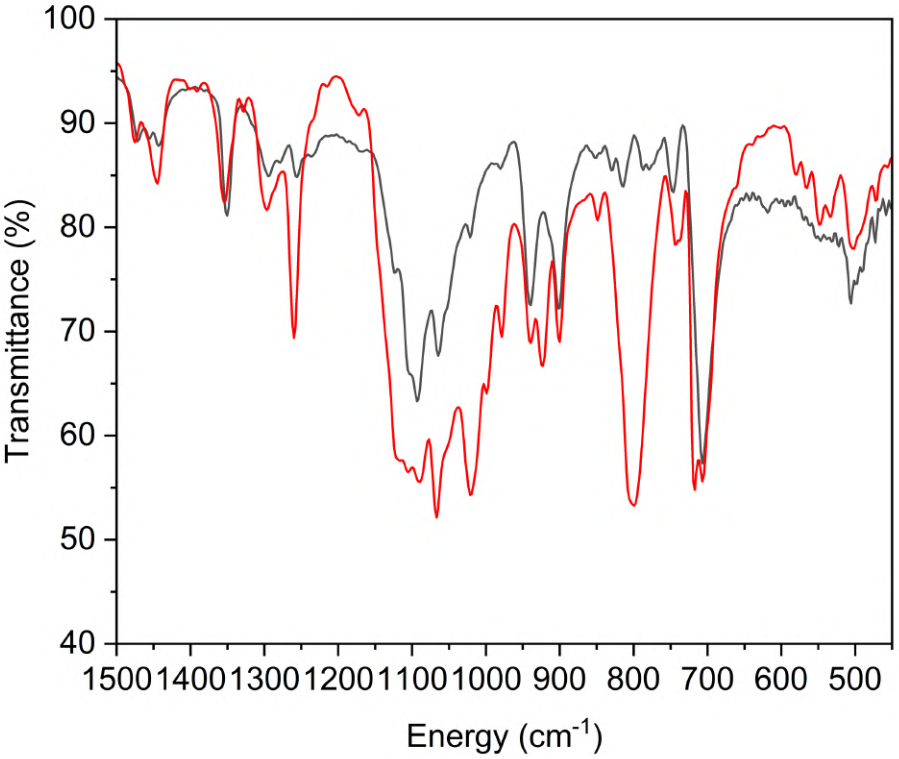

Supplementary Figure 54. Zoomed-in ATR-IR spectra of **4Cs** prepared in protio (black) and deuterio solvents (red). Bridging hydrides or deuterides, respectively, which would be expected to present hydride absorptions at 790–950 and 1,100–1,300 cm^{-1} with D isotopologue shifts of ~230–380 cm^{-1},[19] are not present in samples of **4Cs**.

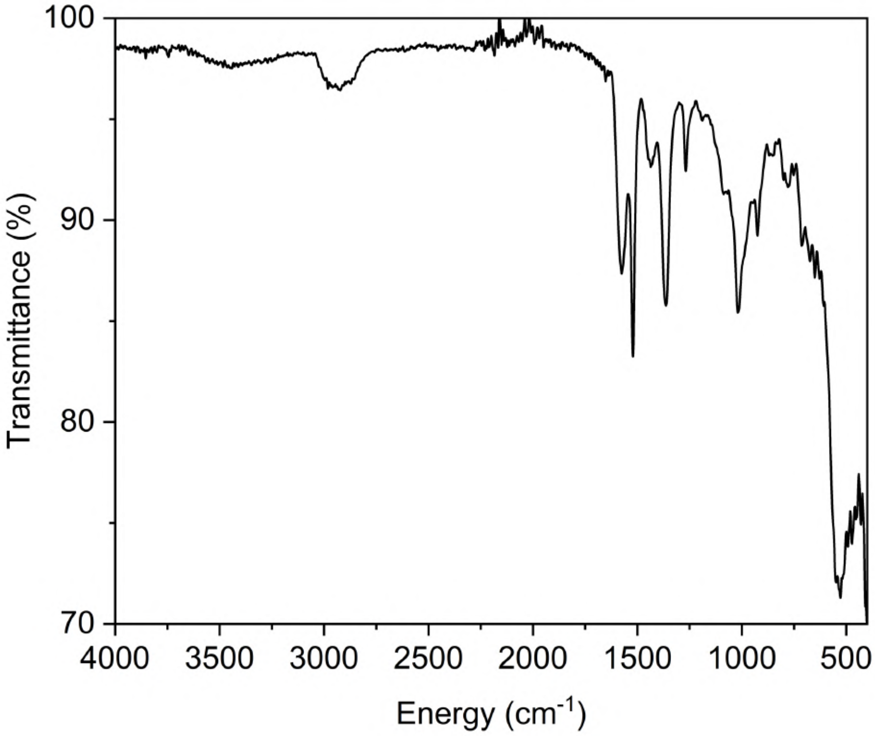

Supplementary Figure 55. ATR-IR spectrum of the grey solid resulting from the reaction of **4Cs** with excess 1,3,5,7-cyclooctatetraene.

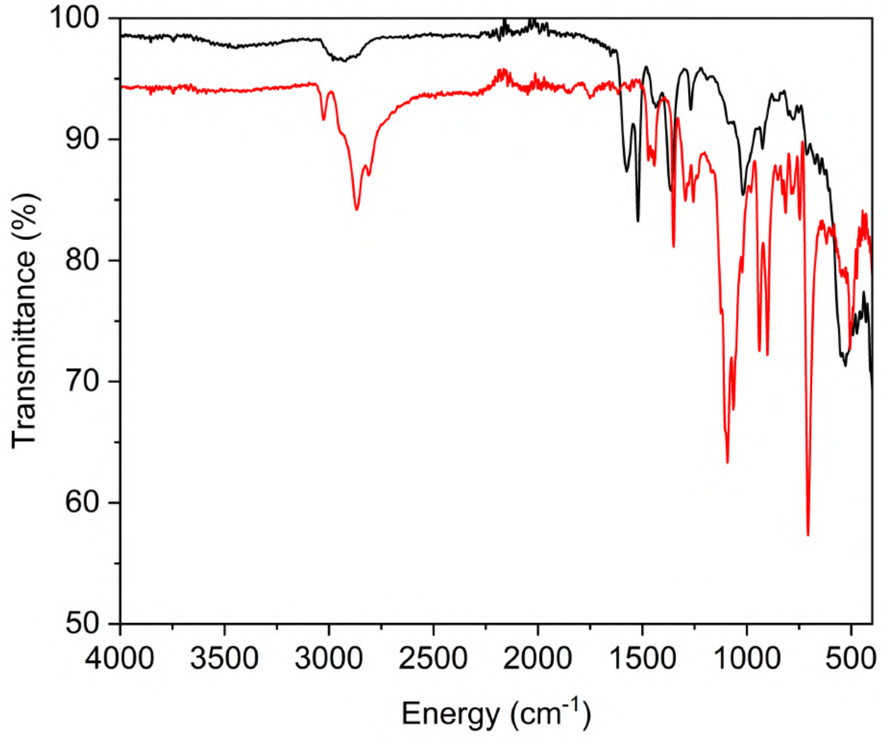

Supplementary Figure 56. Stacked ATR-IR spectra of the grey solid resulting from the reaction of **4Cs** with excess 1,3,5,7-cyclooctatetraene (black) and **4Cs** (red). The absorptions in the FTIR data at ~1575 and 1520 cm^{-1} are indicative of aromatic C=C stretches. These values are shifted with respect to that of neutral 1,3,5,7-cyclooctatetraene (~1640 cm^{-1}).[21] The absorption in the FTIR data at ~1000 cm^{-1} is indicative of aromatic C=C-H in-plane bending.

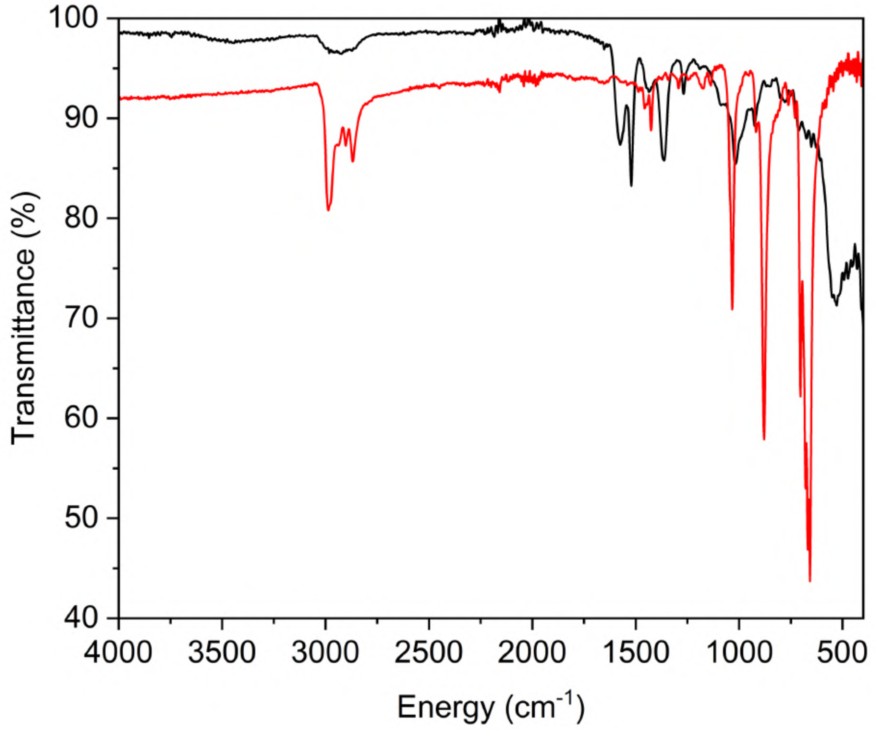

Supplementary Figure 57. Stacked ATR-IR spectra of the grey solid resulting from the reaction of **4Cs** with excess 1,3,5,7-cyclooctatetraene (black) and di-potassium cyclooctatetraene (red).

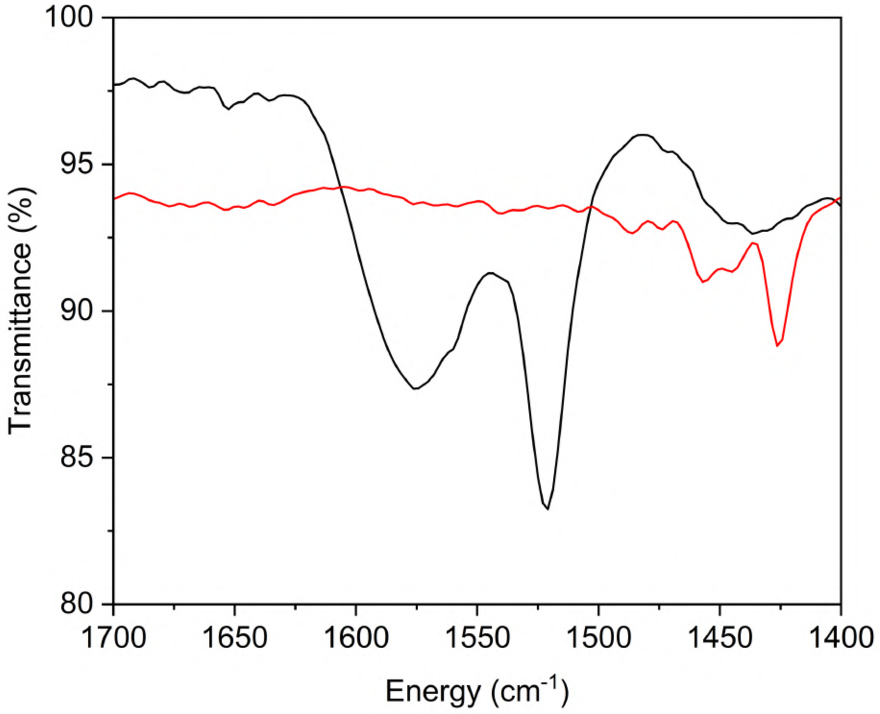

Supplementary Figure 58. Zoomed-in ATR-IR spectra of the grey solid resulting from the reaction of **4Cs** with excess 1,3,5,7-cyclooctatetraene (black) and di-potassium cyclooctatetraene (red), highlighting the shift in aromatic C=C stretches.

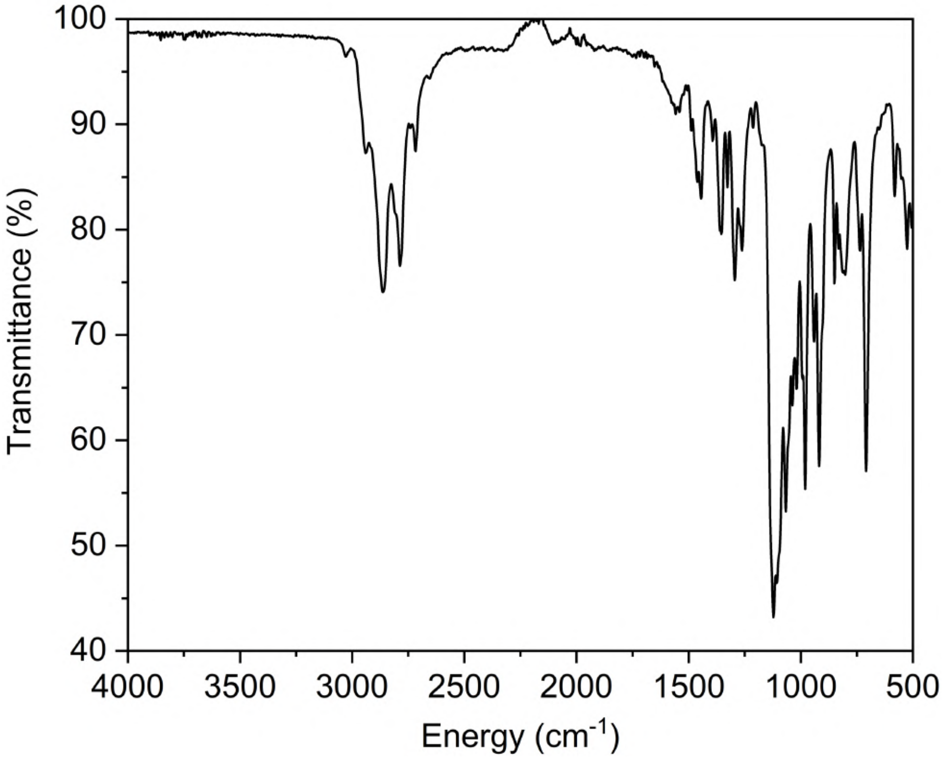

Supplementary Figure 59. ATR-IR spectrum of the grey solid from the reaction of solid **4Cs** with CO_2.

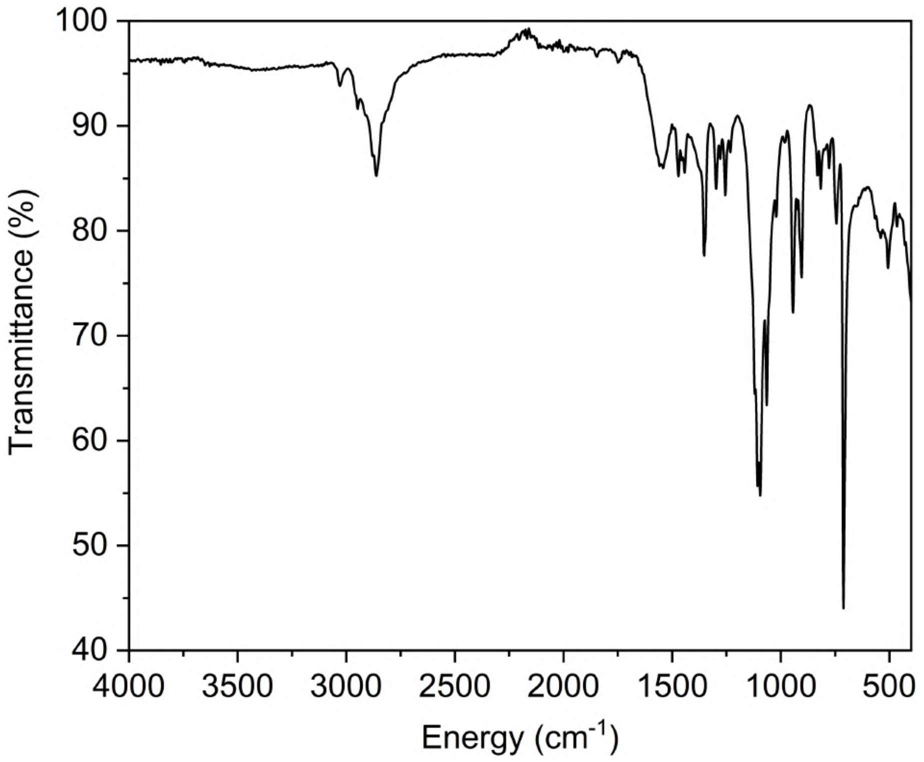

Supplementary Figure 60. ATR-IR spectrum of the pale-yellow solid from the reaction of solid **4Cs** with CO_2.

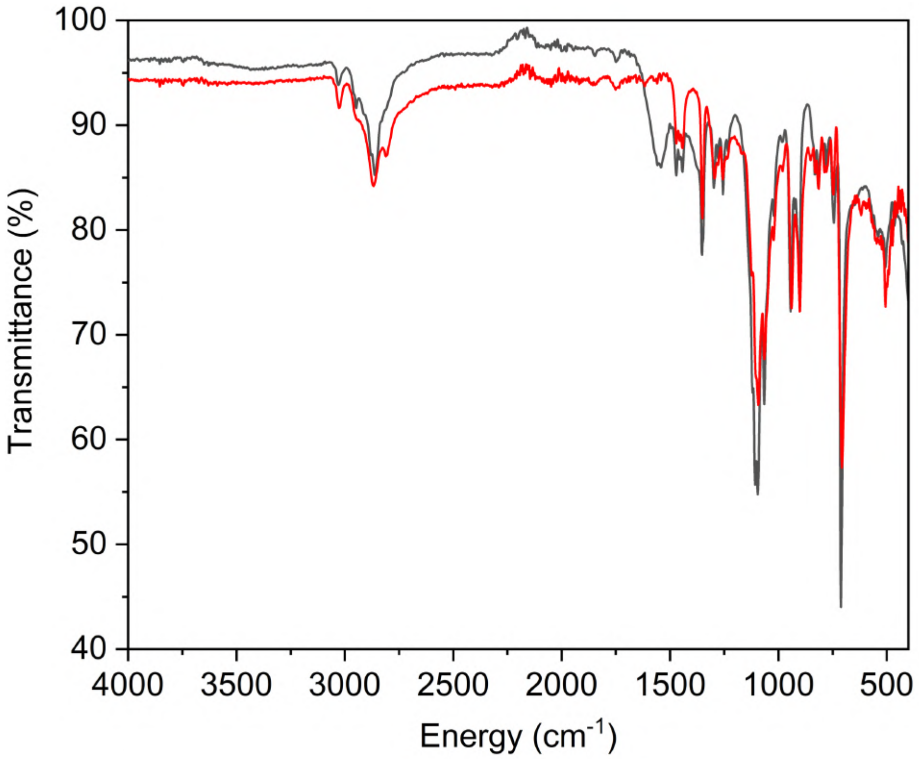

Supplementary Figure 61. Stacked ATR-IR spectra of the pale-yellow solid isolated following the reaction of solid **4Cs** with CO_2 (black), and **4Cs** (red). New absorption at ~1550 cm^{-1} indicative of carbonate formation, not oxalate which would be present at ~1640 cm^{-1}.[22-25]

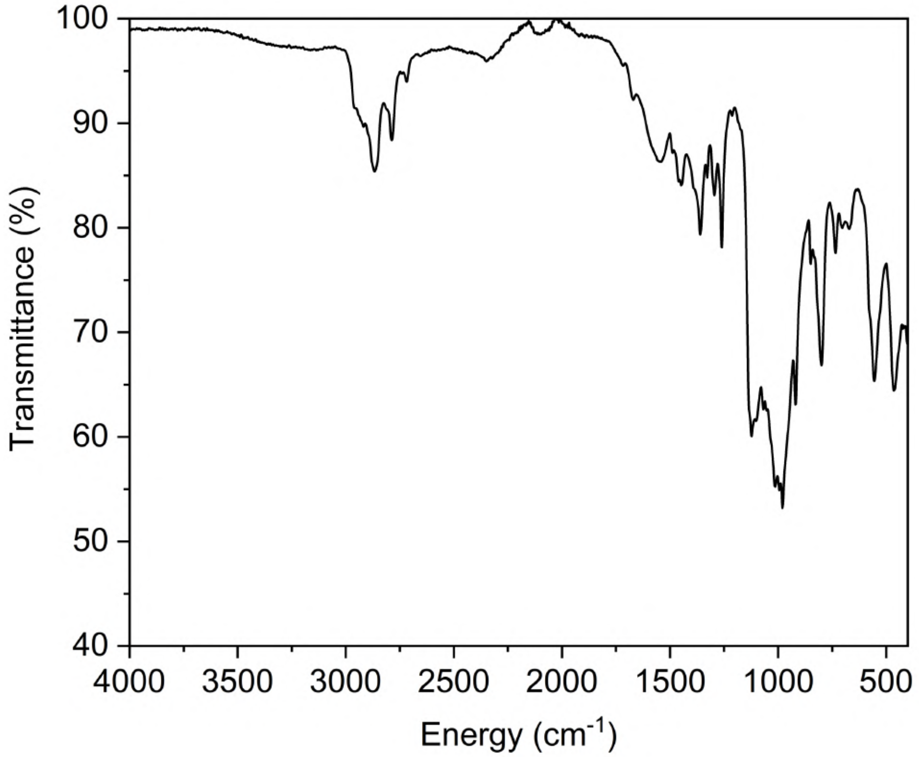

Supplementary Figure 62. ATR-IR spectrum of the pale-yellow solid from the reaction of **4Cs** with CO_2 in D_8-THF.

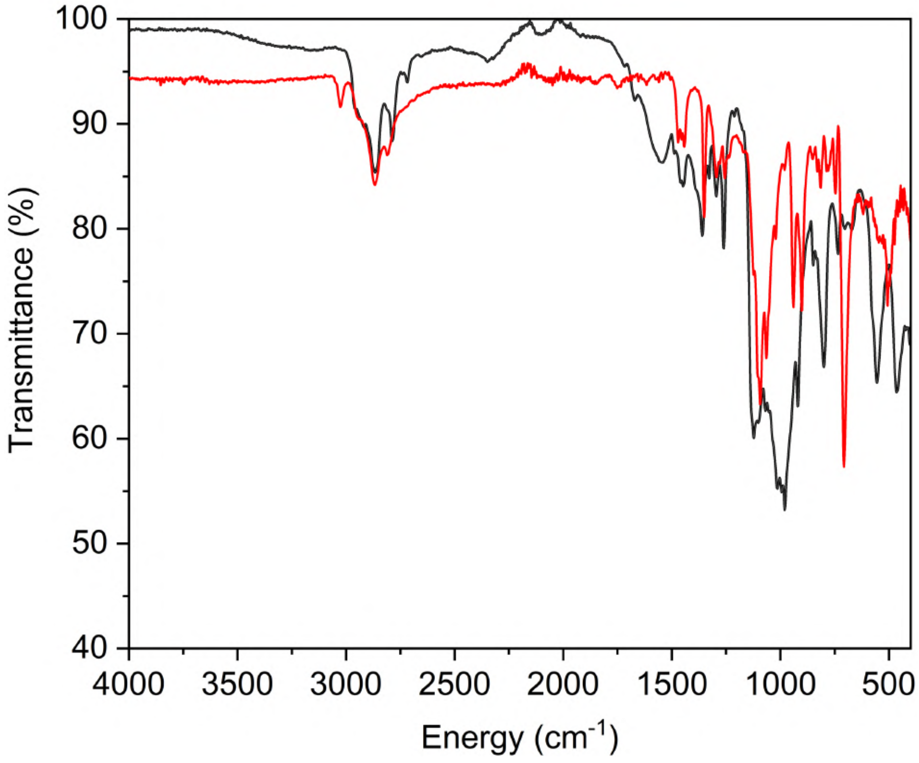

Supplementary Figure 63. Stacked ATR-IR spectra of the pale-yellow solid following the reaction of **4Cs** with CO_2 in D_8-THF (black line), and **4Cs** (red line). New absorptions at ~1550 cm^{-1} and at ~1640 cm^{-1} are indicative of carbonate and oxalate formation.[22-25]

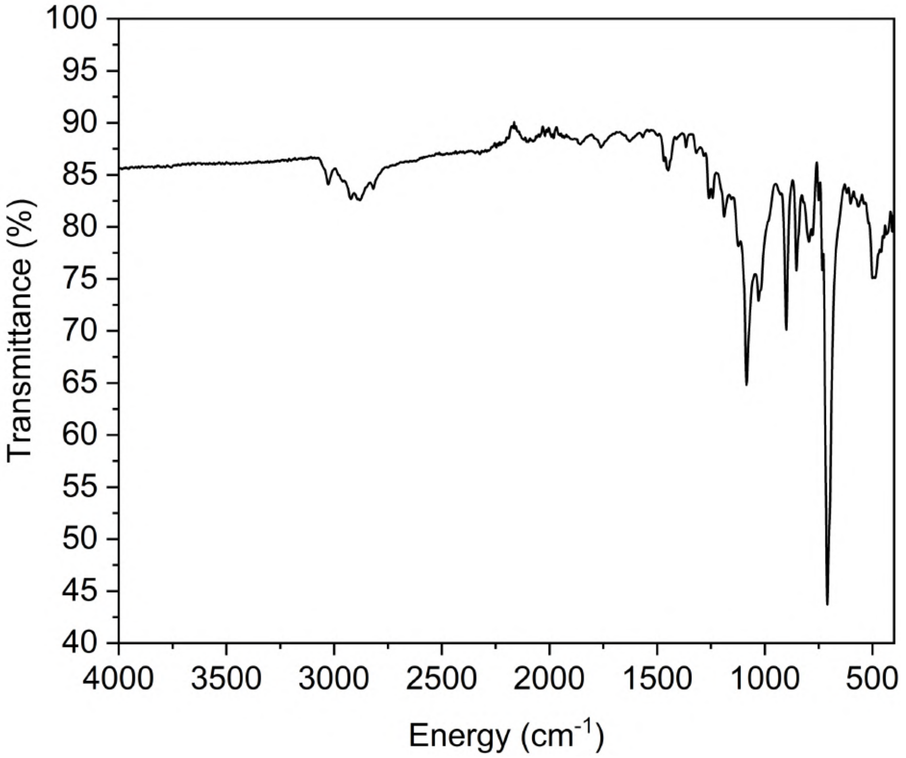

Supplementary Figure 64. Stacked ATR-IR spectra of **5**.

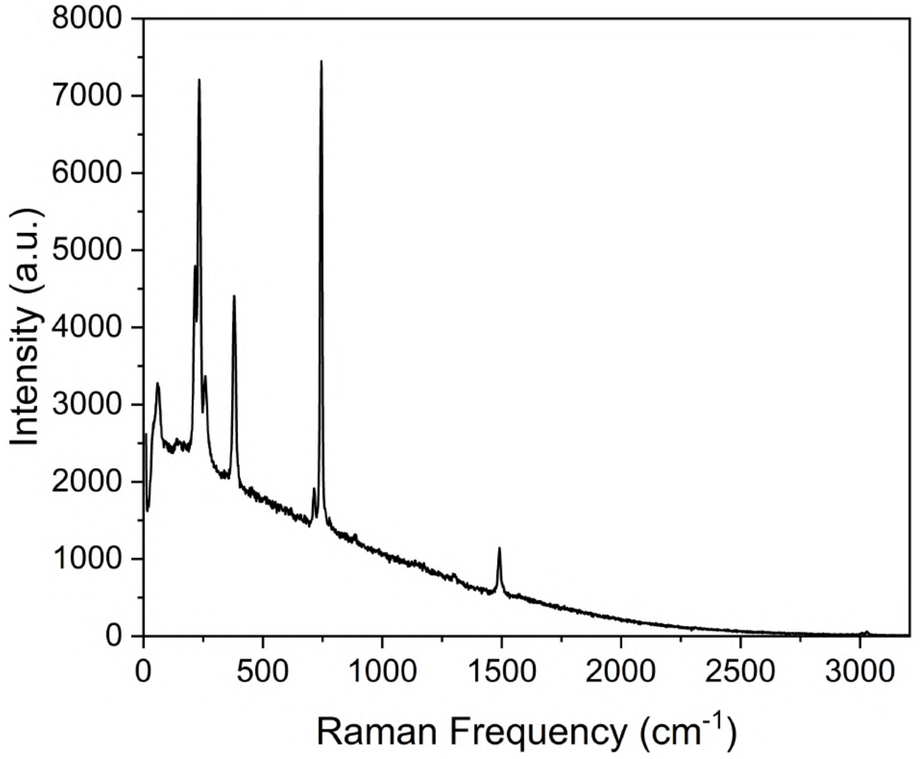

Supplementary Figure 65. Raman spectrum of [$Th(\eta^8-C_8H_8)_2$] using a 785 nm laser. This spectrum matches up excellently with that reported in the literature.[18]

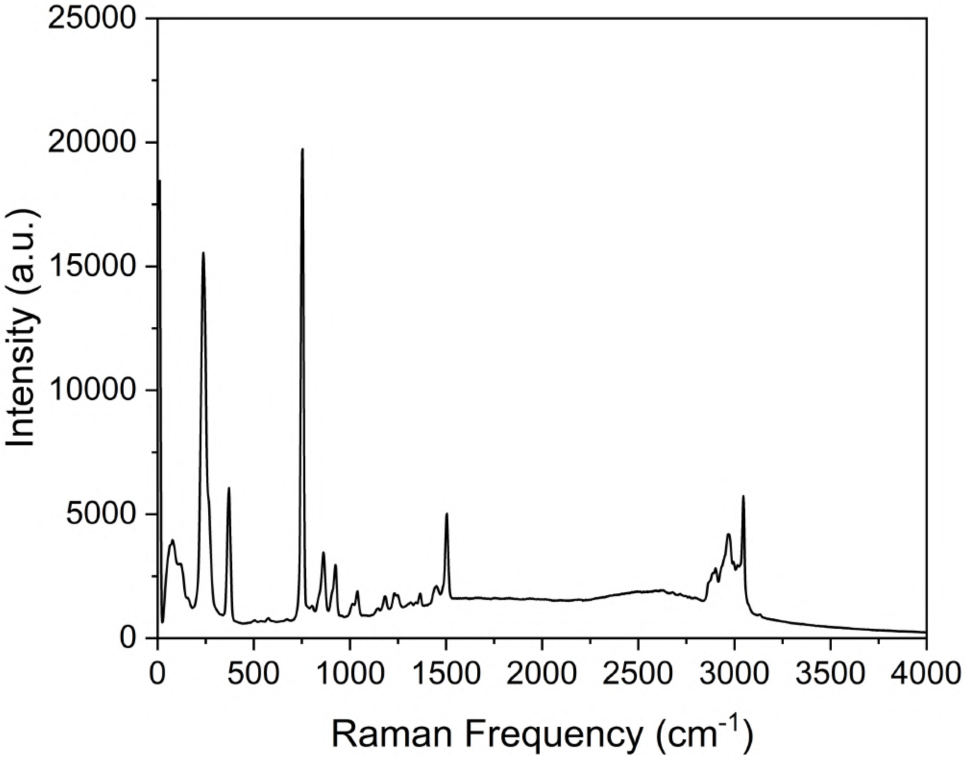

Supplementary Figure 66. Raman spectrum of **1** using a 638 nm laser.

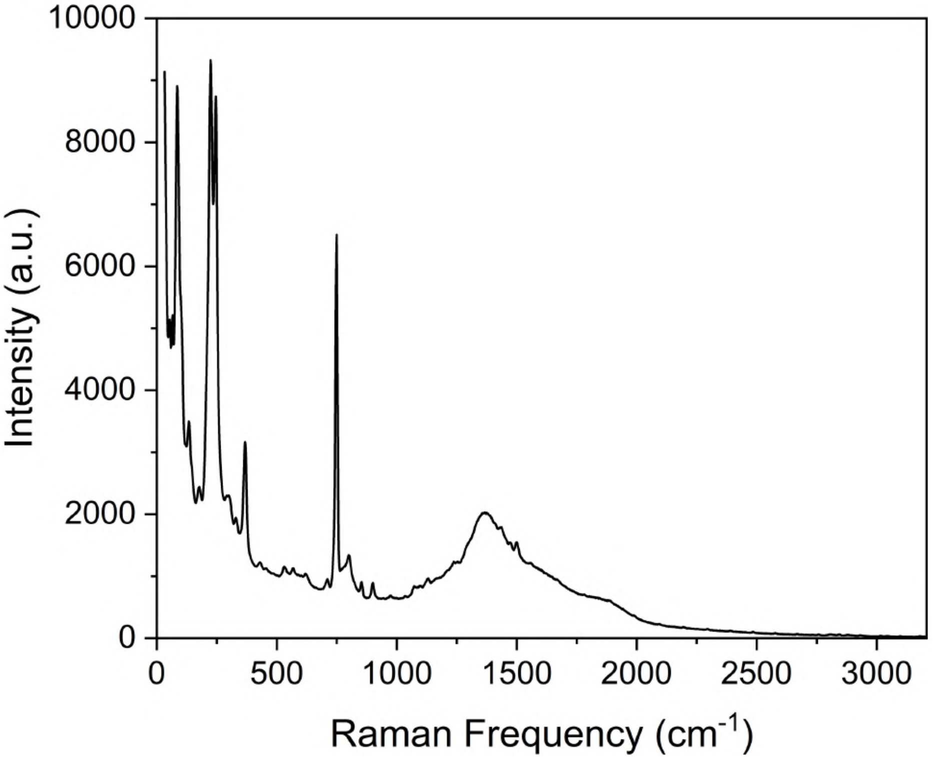

Supplementary Figure 67. Raman spectrum of **4K** using a 785 nm laser.

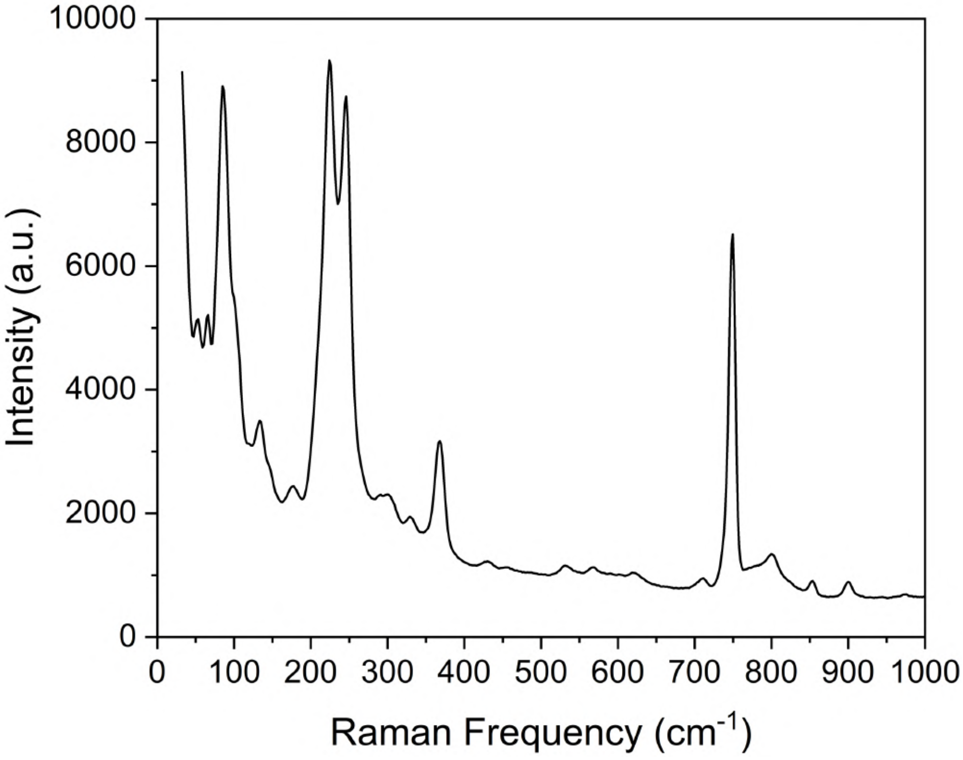

Supplementary Figure 68. Zoom-in of the Raman spectrum of **4K** using a 785 nm laser.

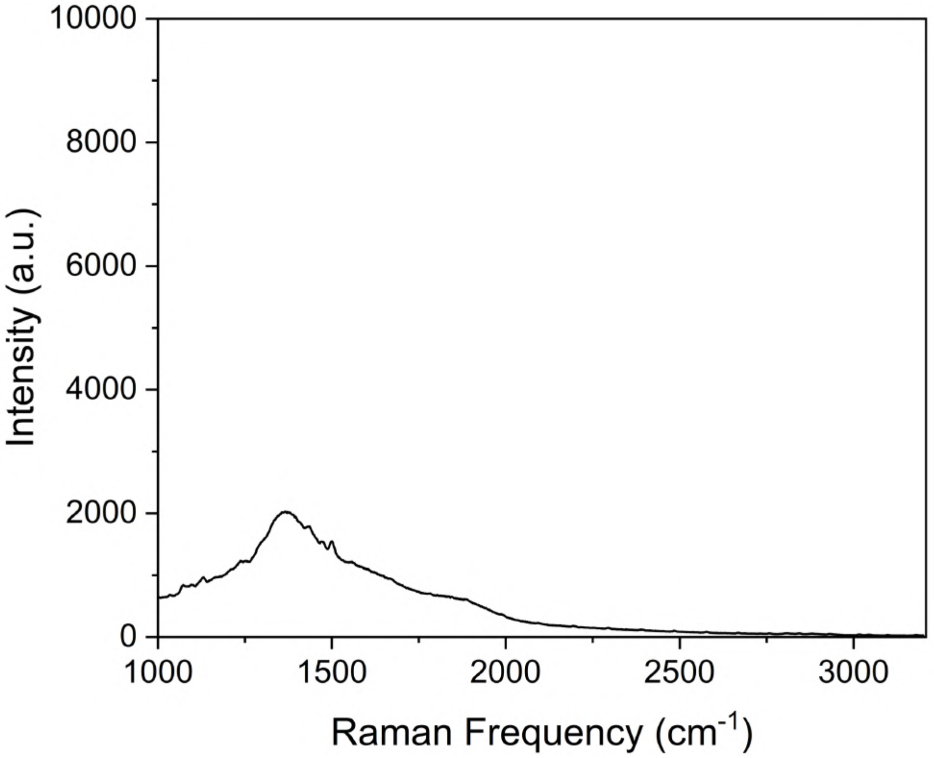

Supplementary Figure 69. Zoom-in of the Raman spectrum of **4K** using a 785 nm laser.

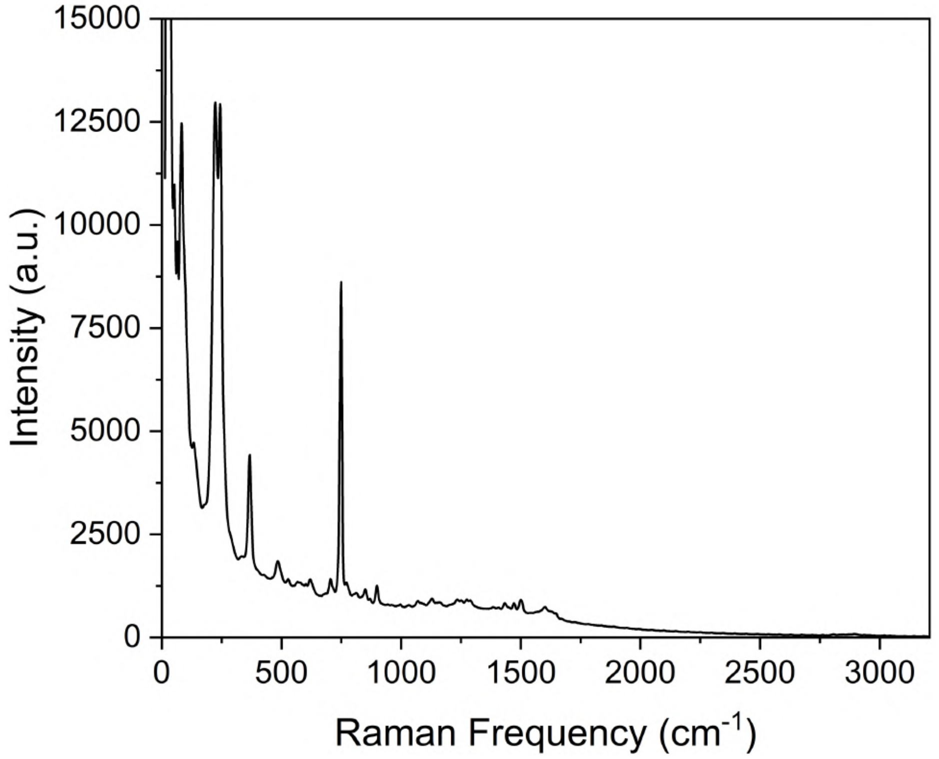

Supplementary Figure 70. Raman spectrum of **4Rb** using a 785 nm laser.

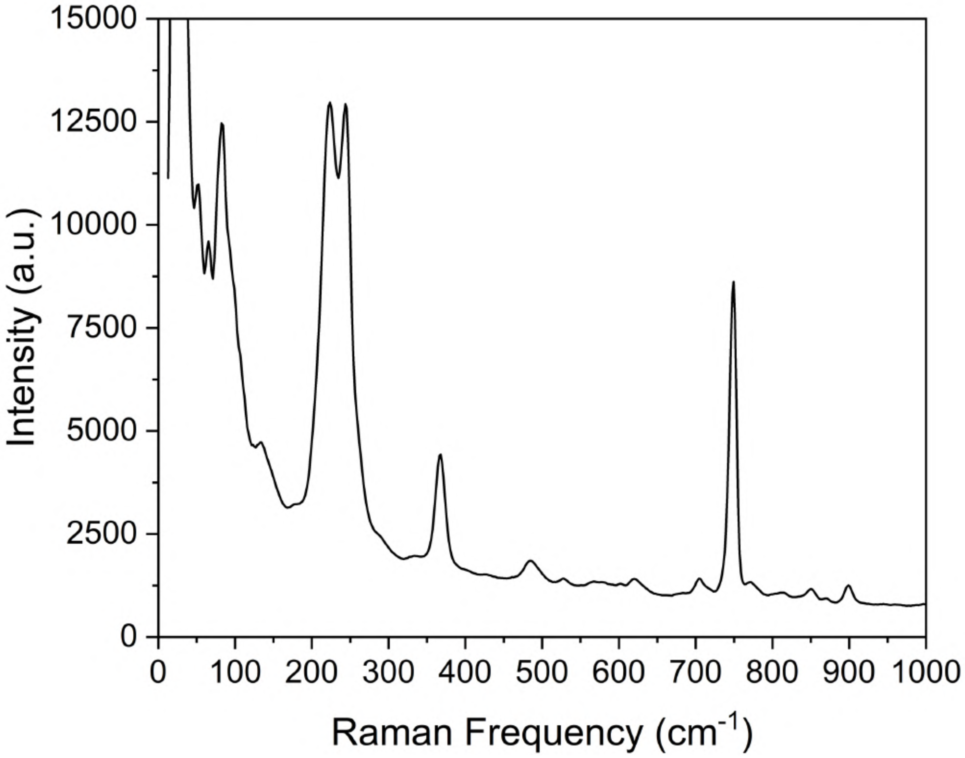

Supplementary Figure 71. Zoom-in of the Raman spectrum of **4Rb** using a 785 nm laser.

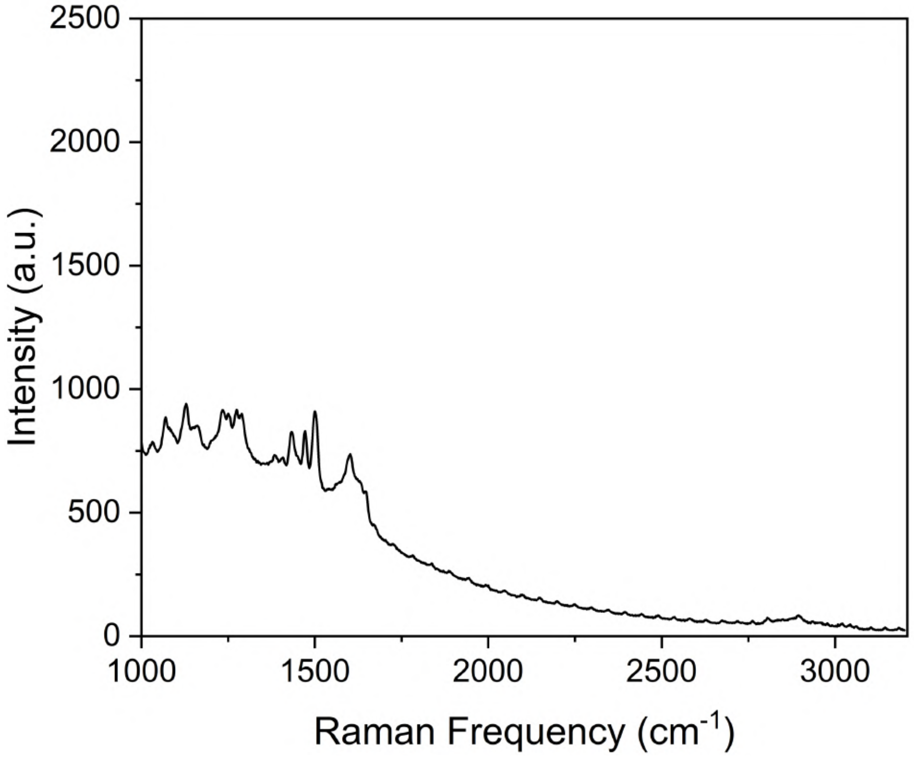

Supplementary Figure 72. Zoom-in of the Raman spectrum of **4Rb** using a 785 nm laser.

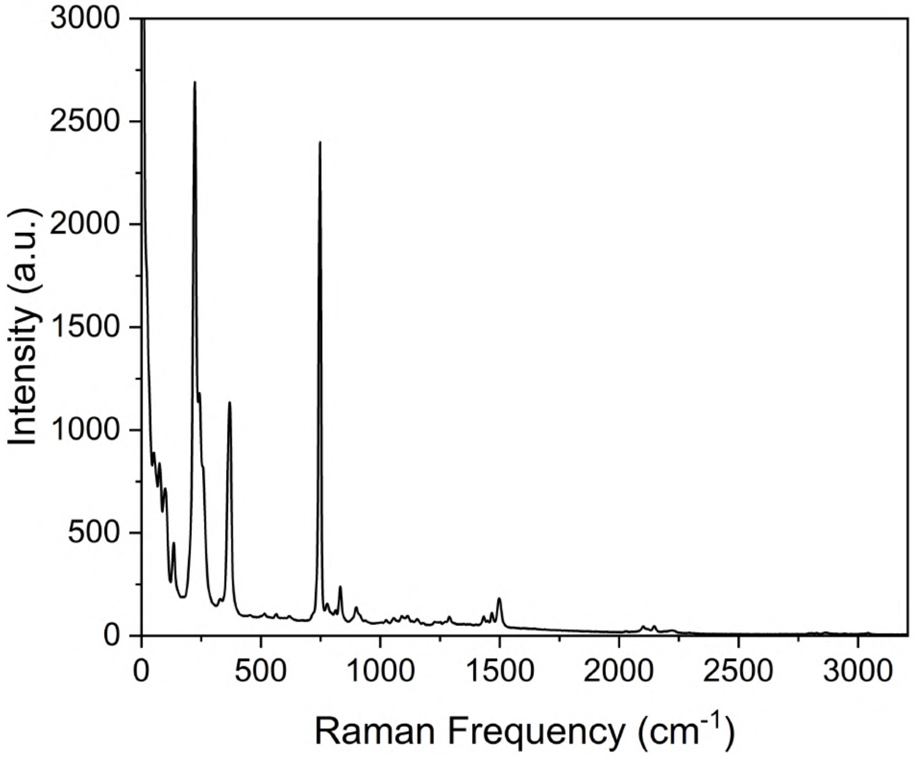

Supplementary Figure 73. Raman spectrum of **4Cs** using a 785 nm laser.

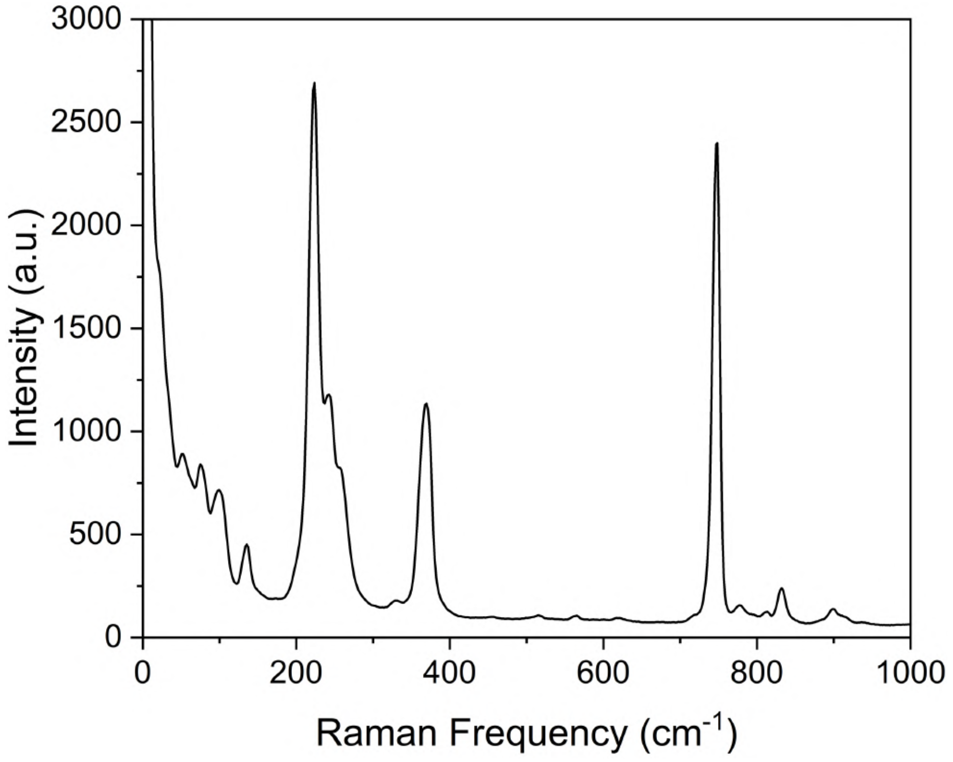

Supplementary Figure 74. Zoom-in of the Raman spectrum of **4Cs** using a 785 nm laser.

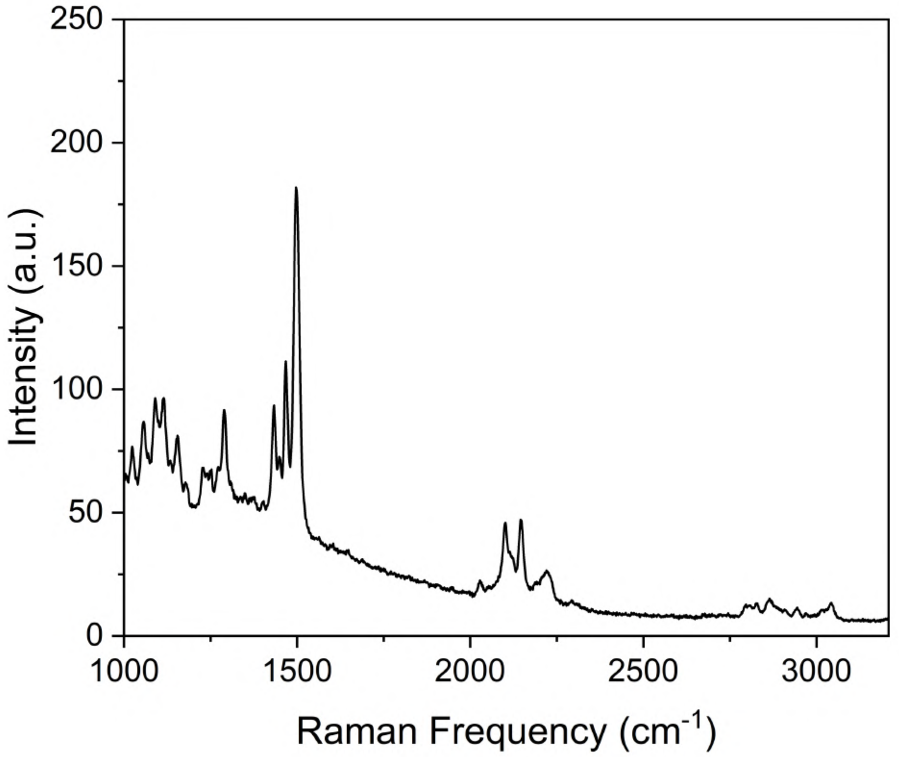

Supplementary Figure 75. Zoom-in of the Raman spectrum of **4Cs** using a 785 nm laser.

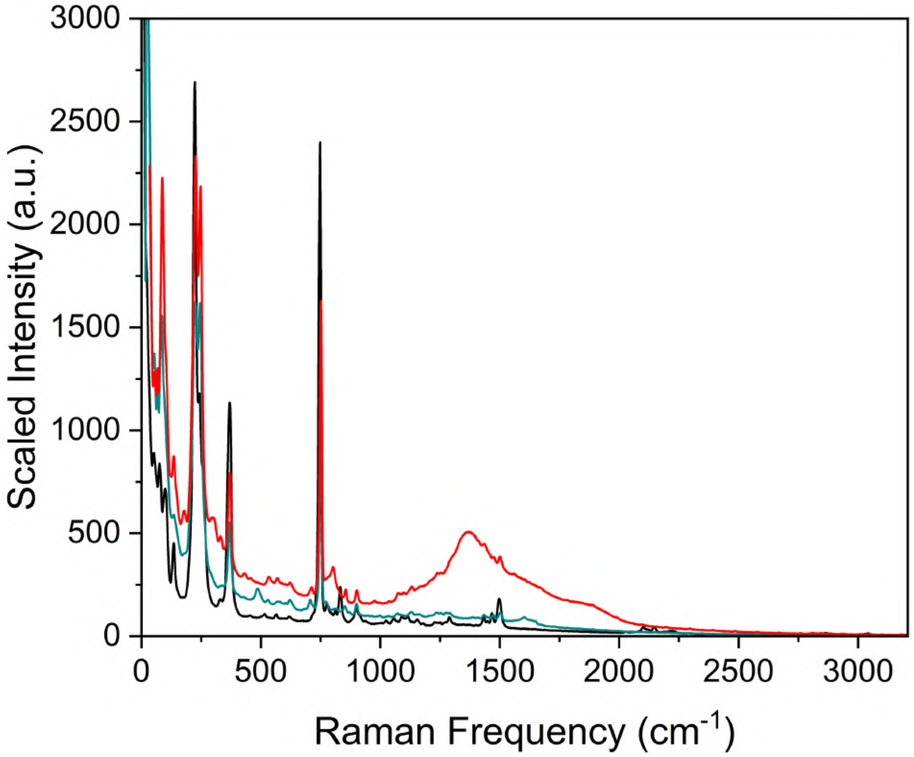

Supplementary Figure 76. Stacked Raman spectra of **4K** (red), **4Rb** (cyan) and **4Cs** (black).

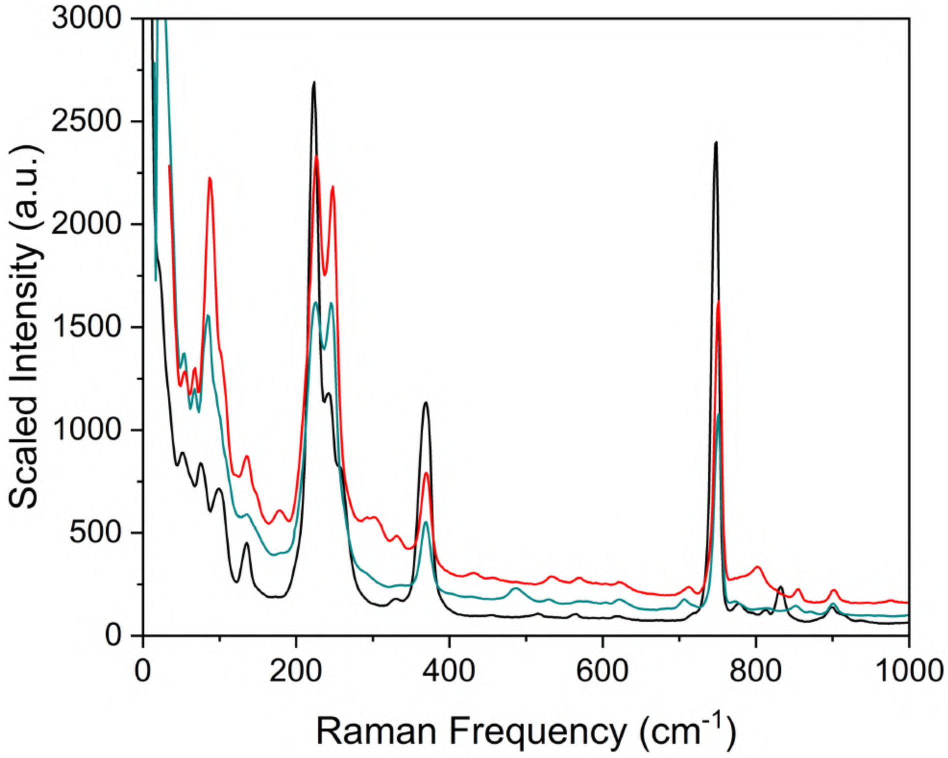

Supplementary Figure 77. Zoom-in of stacked Raman spectra **4K** (red), **4Rb** (cyan) and **4Cs** (black).

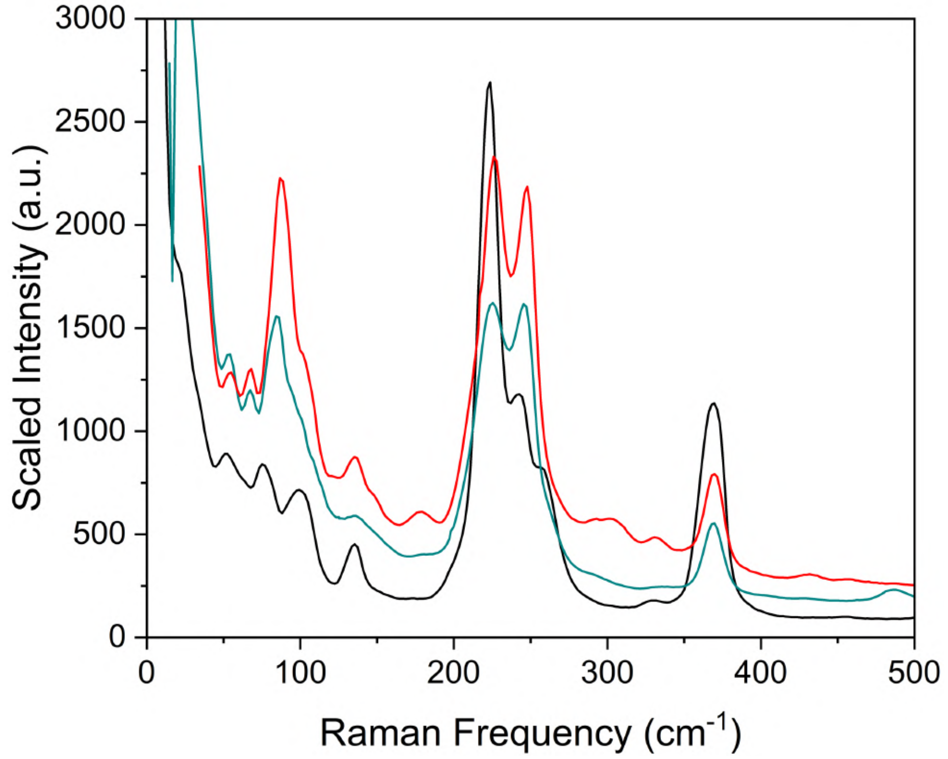

Supplementary Figure 78. Zoom-in of stacked Raman spectra of **4K** (red), **4Rb** (cyan) and **4Cs** (black).

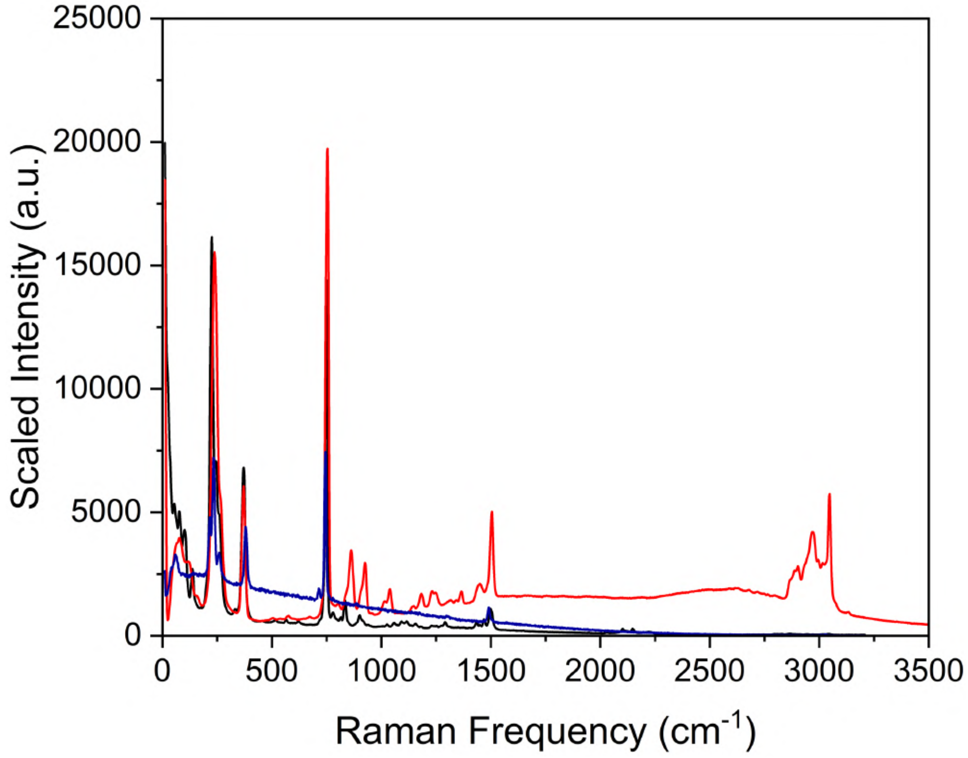

Supplementary Figure 79. Stacked Raman spectra of [Th(η^8-C_8H_8)$_2$] (blue), **1** (red), and **4Cs** (black).

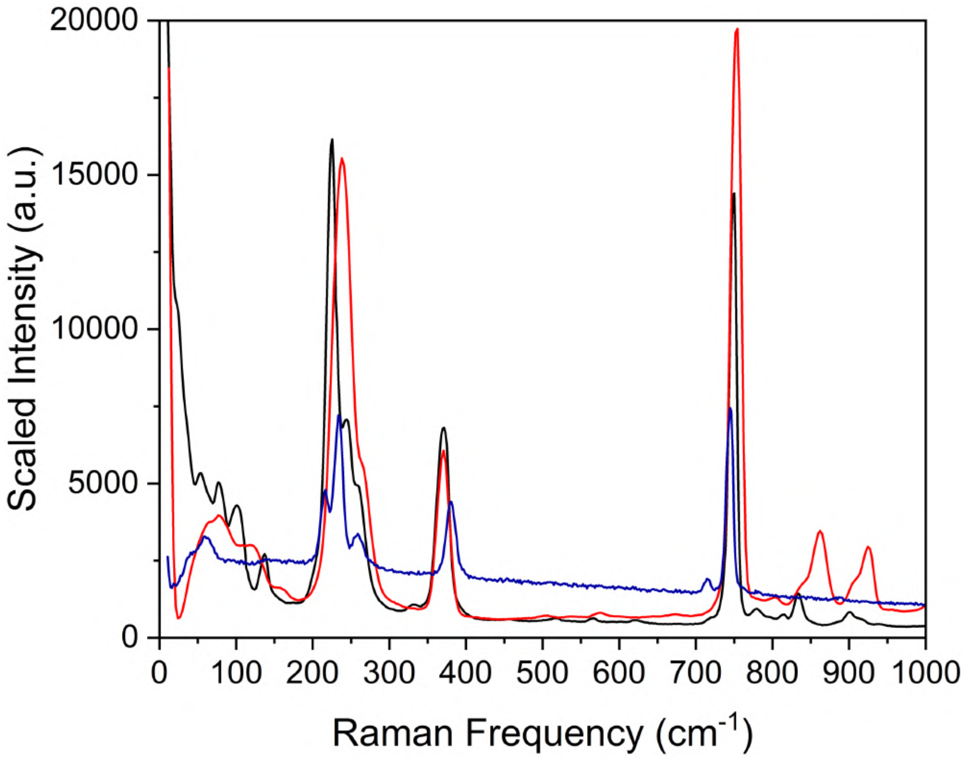

Supplementary Figure 80. Zoom-in of stacked Raman spectra of [Th(η^8-C_8H_8)$_2$] (blue), **1** (red), and **4Cs** (black).

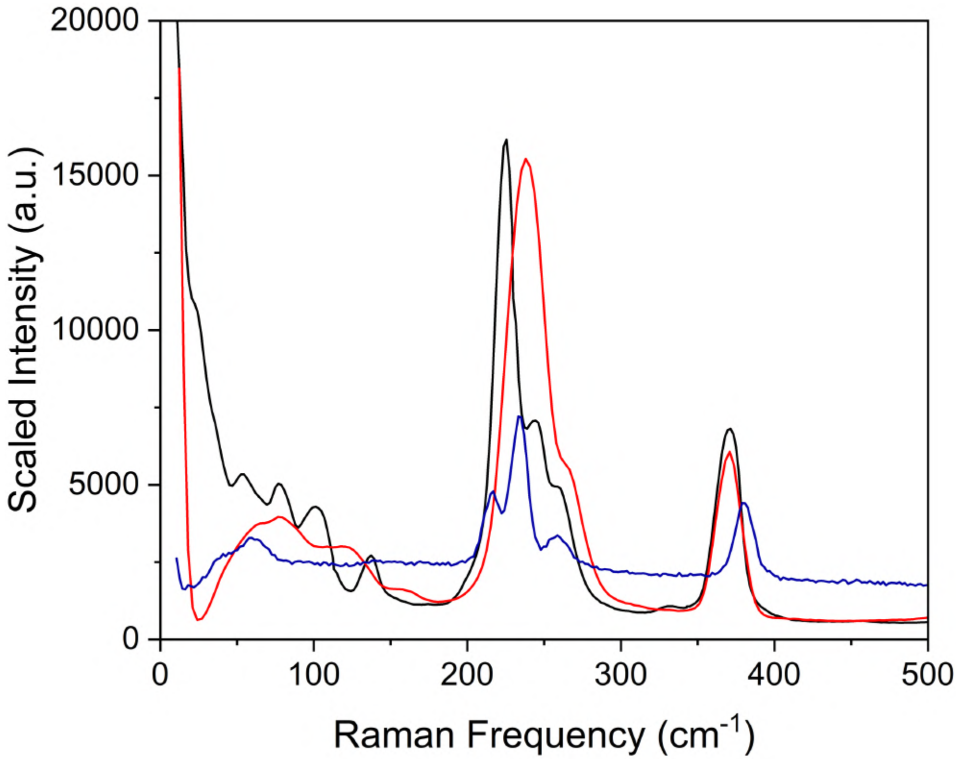

Supplementary Figure 81. Zoom-in of stacked Raman spectra of [Th(η^8-C_8H_8)$_2$] (blue), **1** (red), and **4Cs** (black).

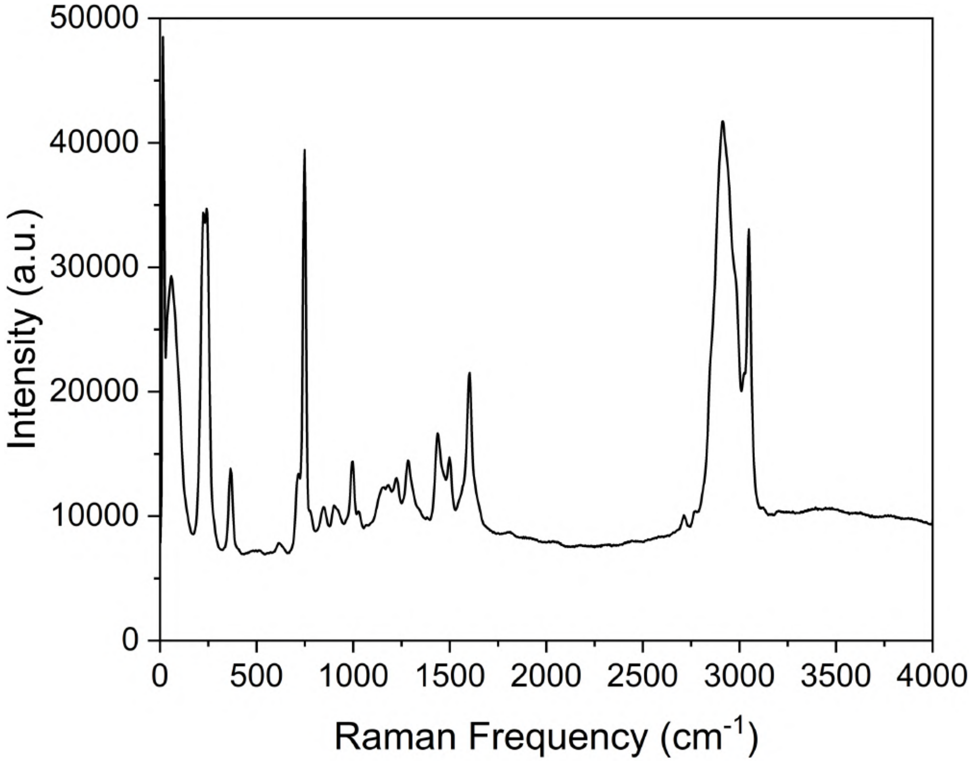

Supplementary Figure 82. Raman spectrum of **5** using a 532 nm laser.

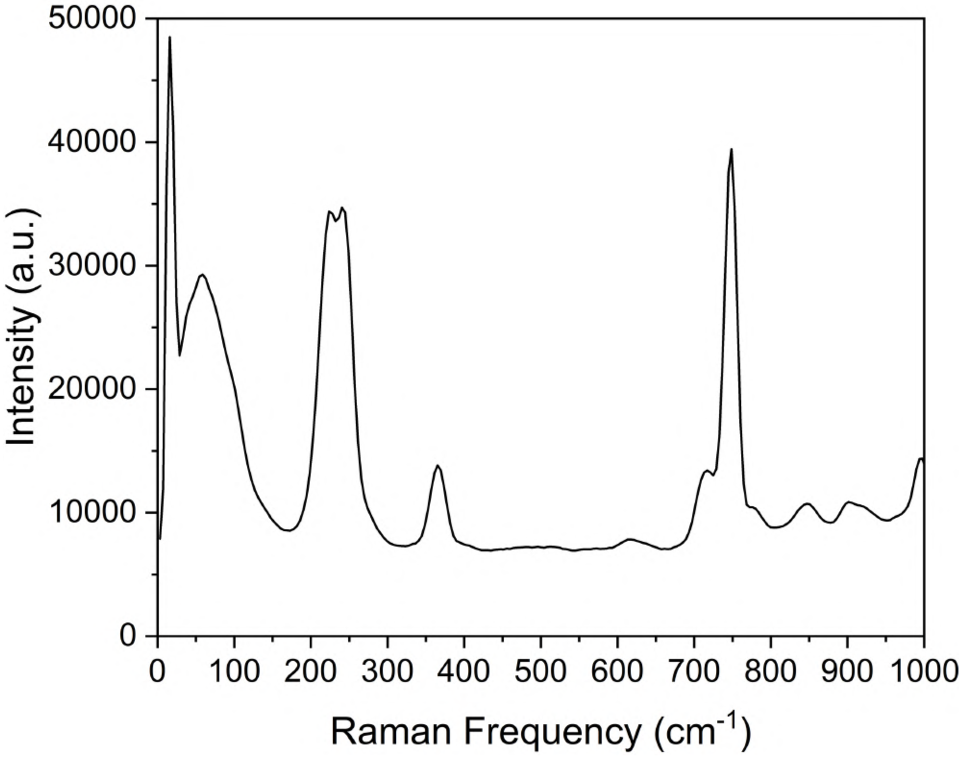

Supplementary Figure 83. Zoom-in of the Raman spectrum of **5** using a 532 nm laser.

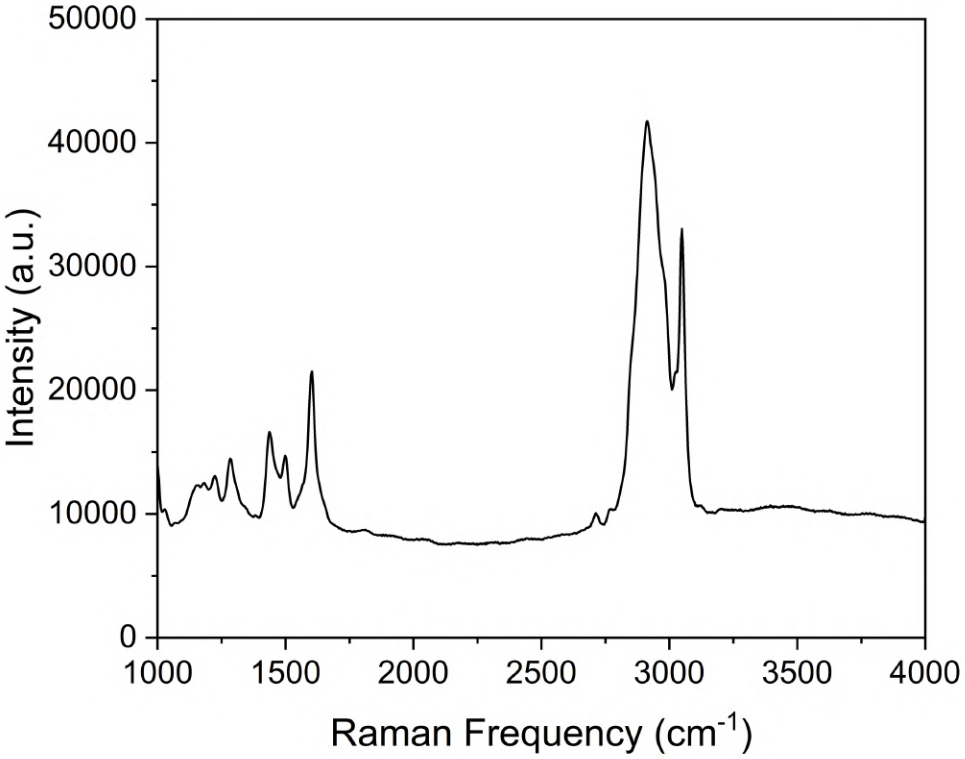

Supplementary Figure 84. Zoom-in of the Raman spectrum of **5** using a 532 nm laser.

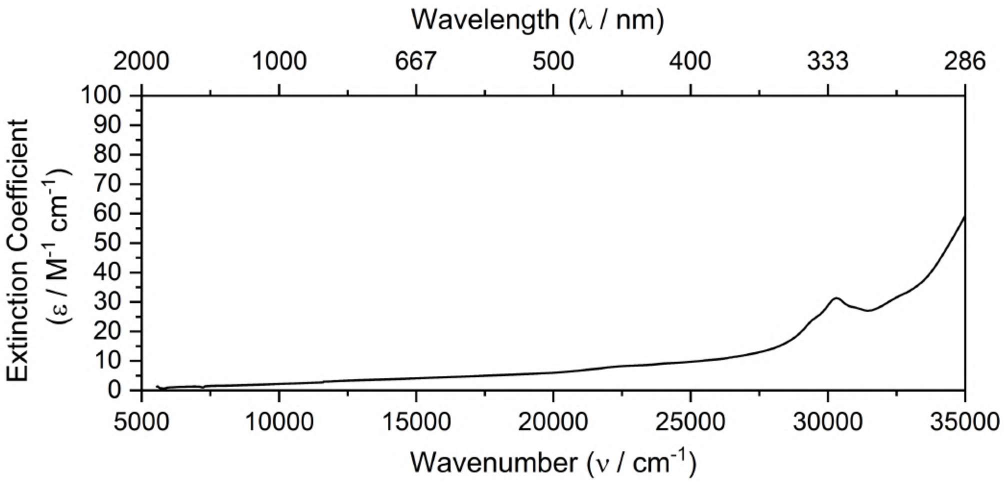

Supplementary Figure 85. UV/Vis/NIR spectrum of [Th(η^8-C_8H_8)$_2$] (15 mM in DME).

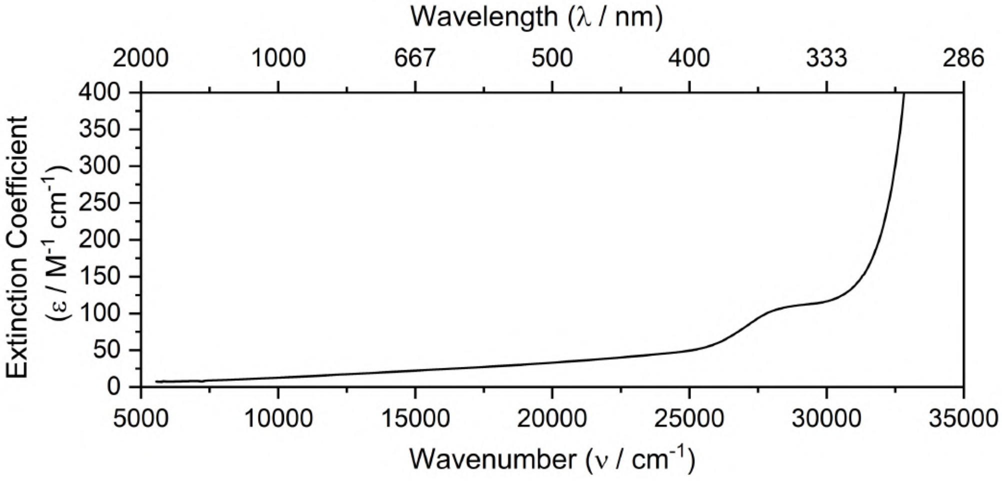

Supplementary Figure 86. UV/Vis/NIR spectrum of **1** (9 mM in DME).

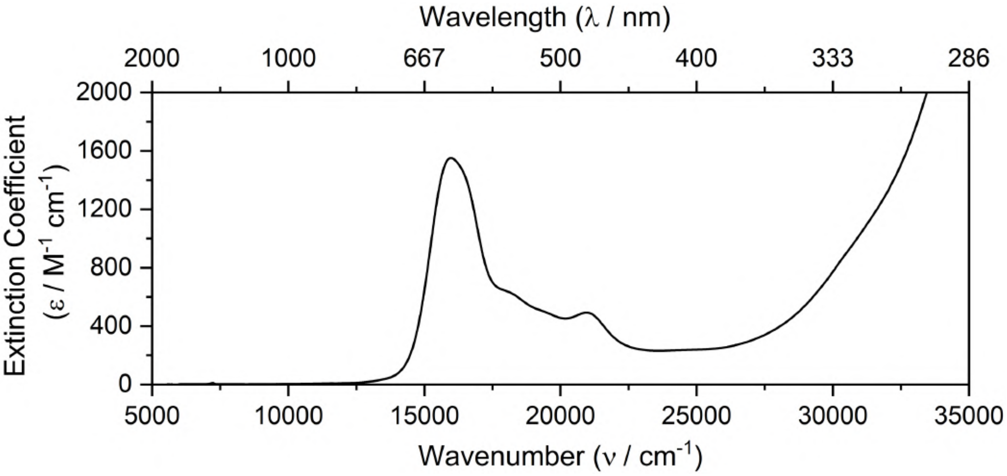

Supplementary Figure 87. UV/Vis/NIR spectrum of **4K** (0.87 mM in DME).

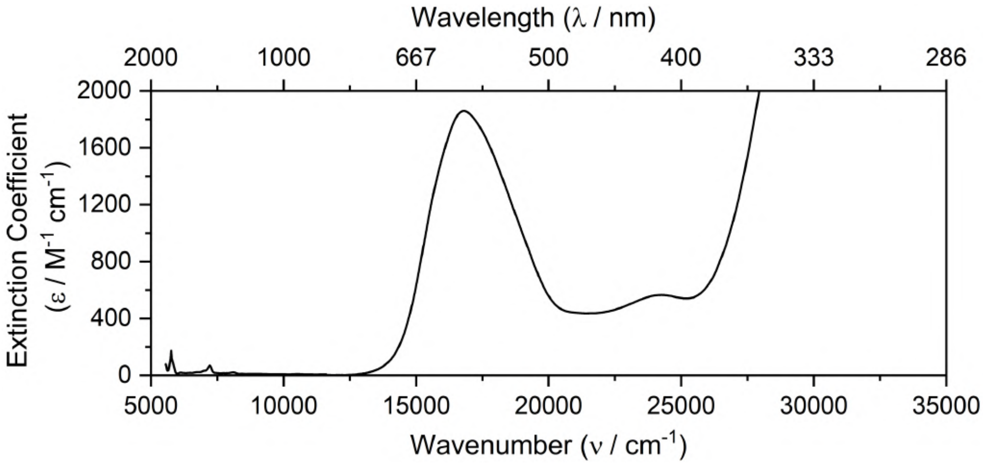

Supplementary Figure 88. UV/Vis/NIR spectrum of **4Rb** (0.23 mM in DME).

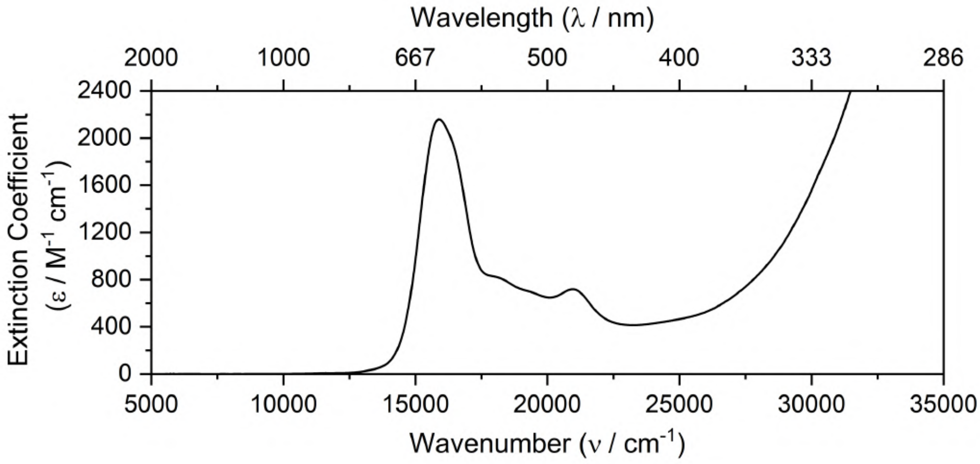

Supplementary Figure 89. UV/Vis/NIR spectrum of **4Rb** (0.79 mM in DME).

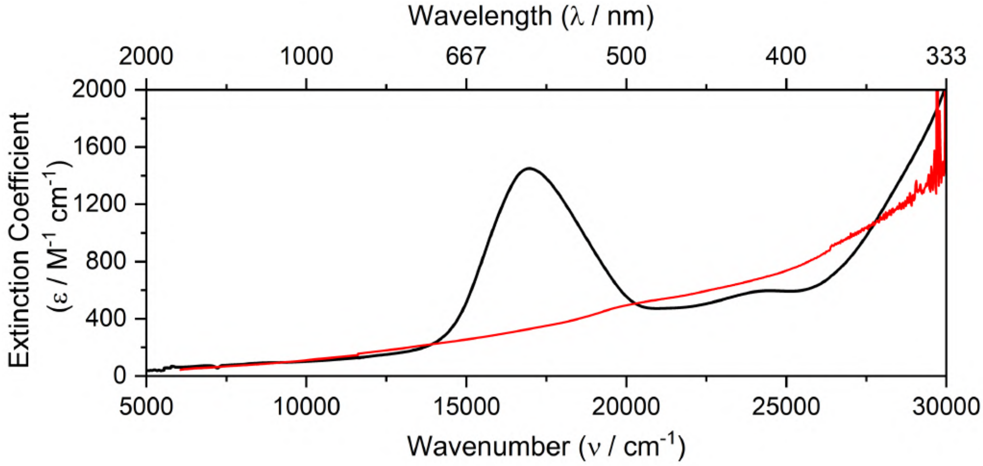

Supplementary Figure 90. UV/Vis/NIR spectrum of **4Cs** (black, 0.44 mM in DME) and after exposure to air (red).

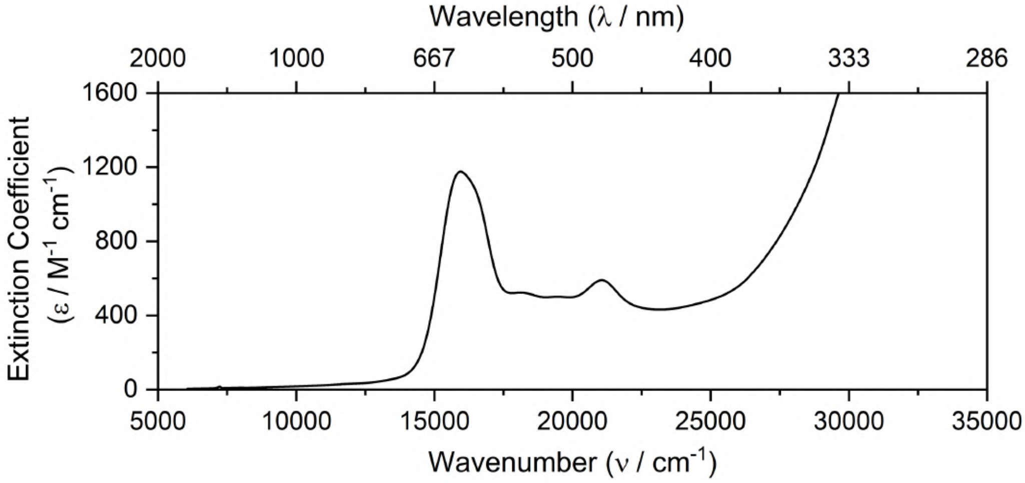

Supplementary Figure 91. UV/Vis/NIR spectrum of **4Cs** (black, 1.50 mM in DME).

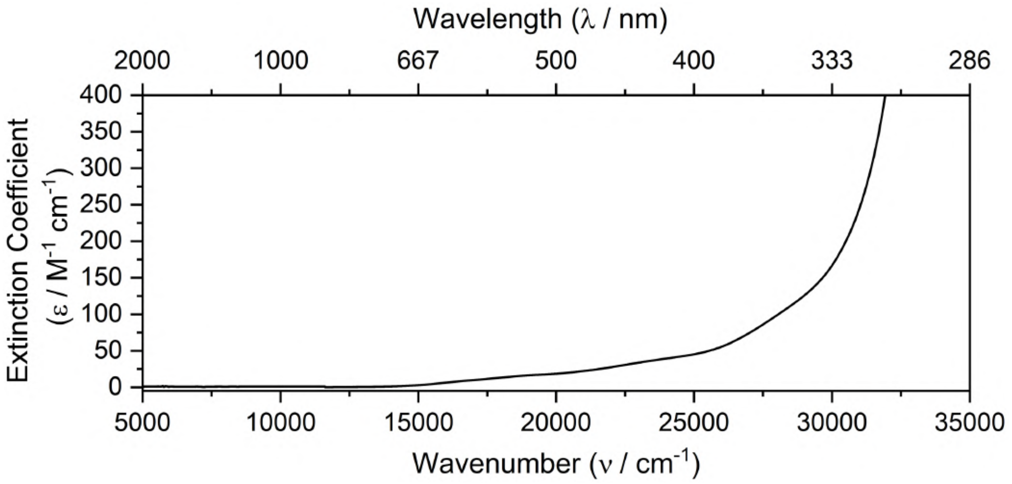

Supplementary Figure 92. UV/Vis/NIR spectrum of **5** (5.4 mM in DME).

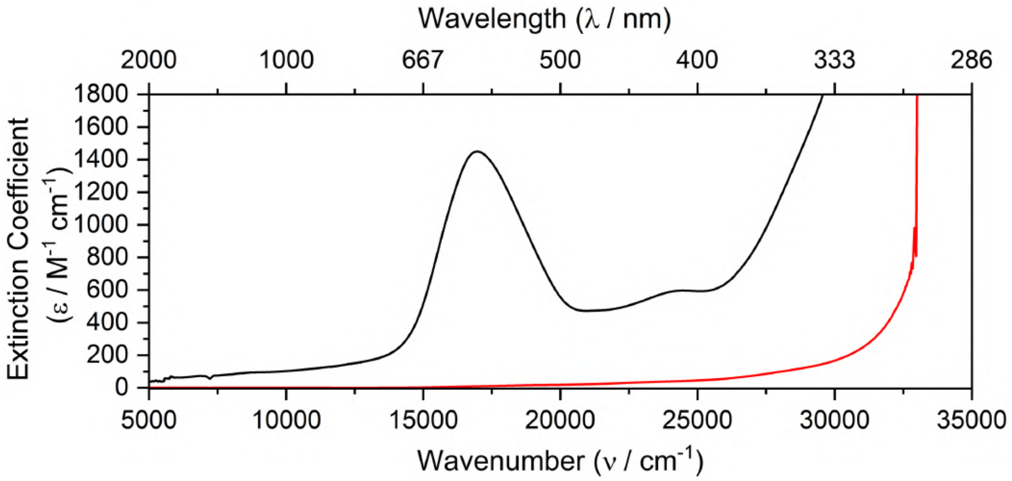

Supplementary Figure 93. Stacked UV/Vis/NIR spectra (DME) of **4Cs** (black) and **5** (red).

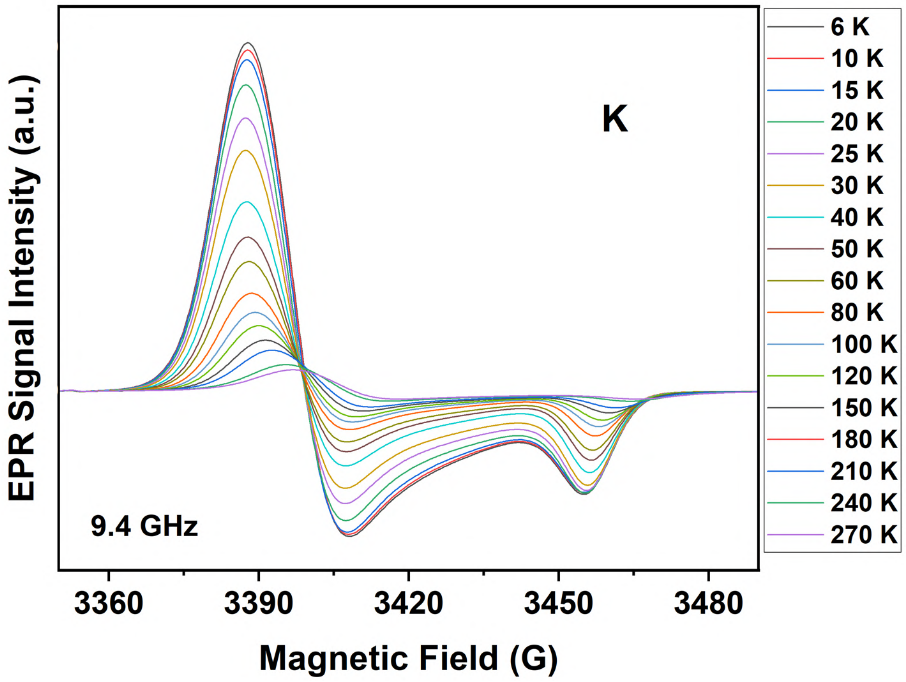

Supplementary Figure 94. X-band (9.4 GHz) EPR spectra of a powdered sample of **4K**, measured at varied temperatures between 6 and 270 K, under modulation frequency of 100 kHz, modulation amplitude of 2 G, and microwave attenuation of 25 dB.

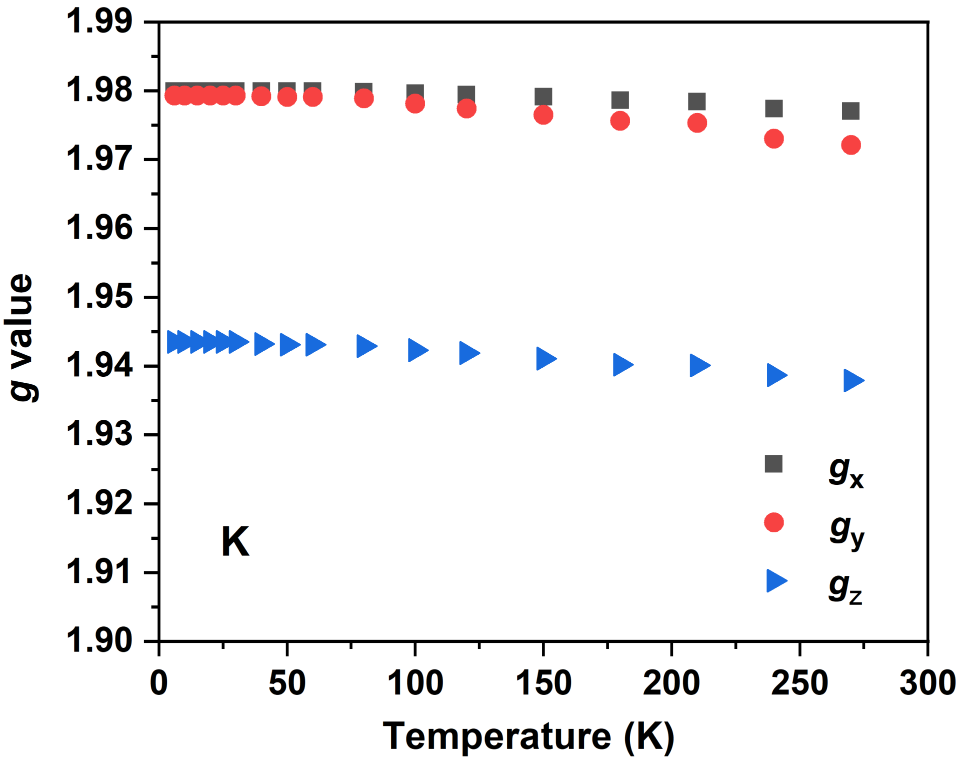

Supplementary Figure 95. Temperature dependence of the extracted *g*-values for **4K**.

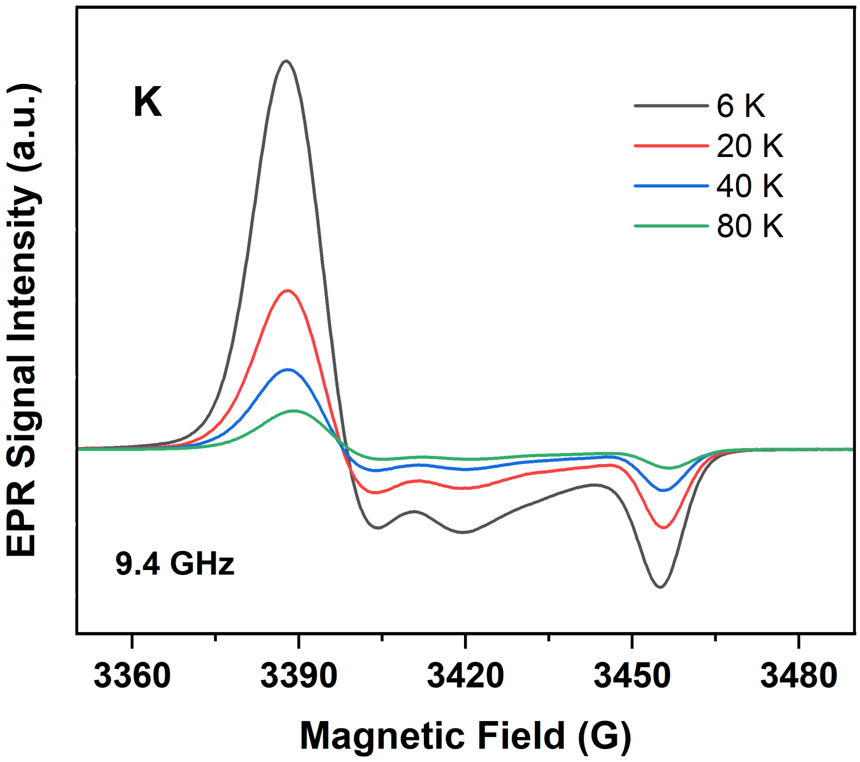

Supplementary Figure 96. X-band (9.4 GHz) EPR spectra of a frozen solution (DME:Toluene. 95:5, 3mM) of **4K** measured at 6, 20, 40 and 80 K (attenuation, 25 dB; modulation frequency, 100 kHz; modulation amplitude, 2 G).

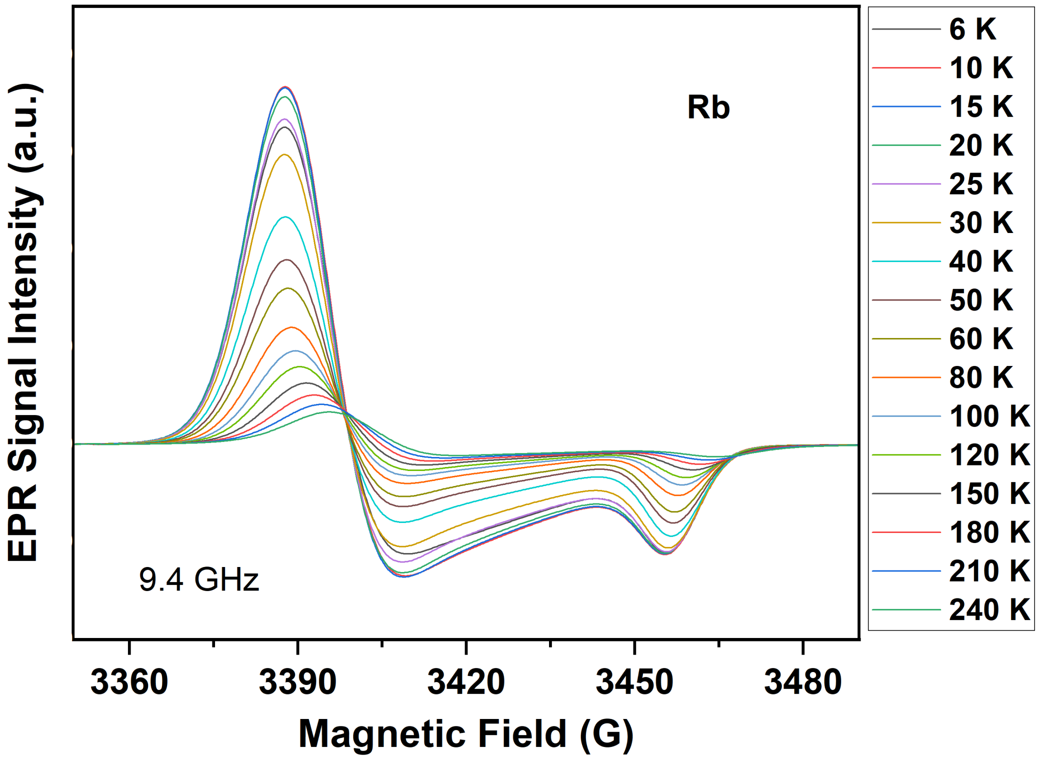

Supplementary Figure 97. X-band (9.4 GHz) EPR spectra of a powdered sample of **4Rb**, measured at varied temperatures between 6 and 240 K, under modulation frequency of 100 kHz, modulation amplitude of 2 G, and microwave attenuation of 25 dB.

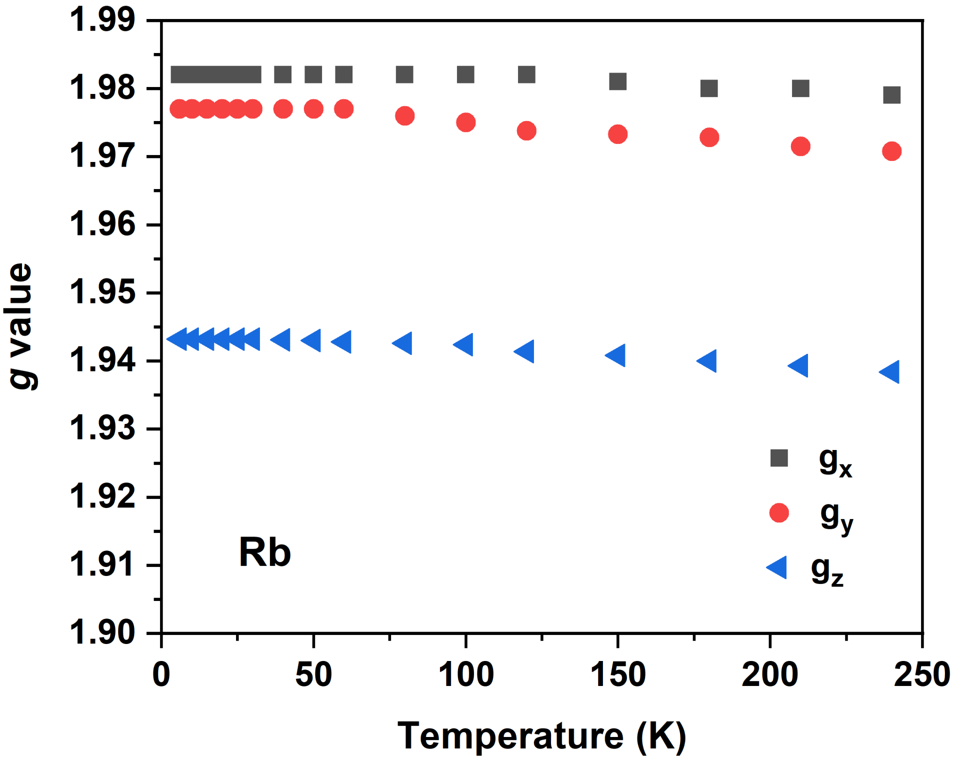

Supplementary Figure 98. Temperature dependence of the extracted *g*-values for **4Rb**.

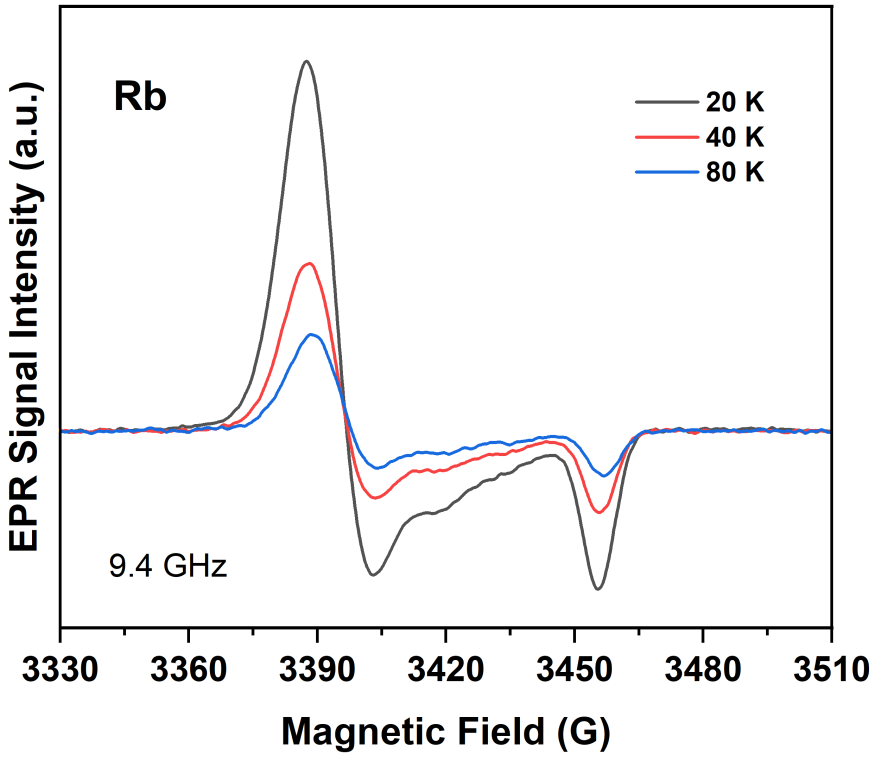

Supplementary Figure 99. X-band (9.4 GHz) EPR spectra of a frozen solution (DME:Toluene. 95:5, 3mM) of **4Rb** measured at 20, 40 and 80 K (attenuation, 40 dB; modulation frequency, 100 kHz; modulation amplitude, 2 G).

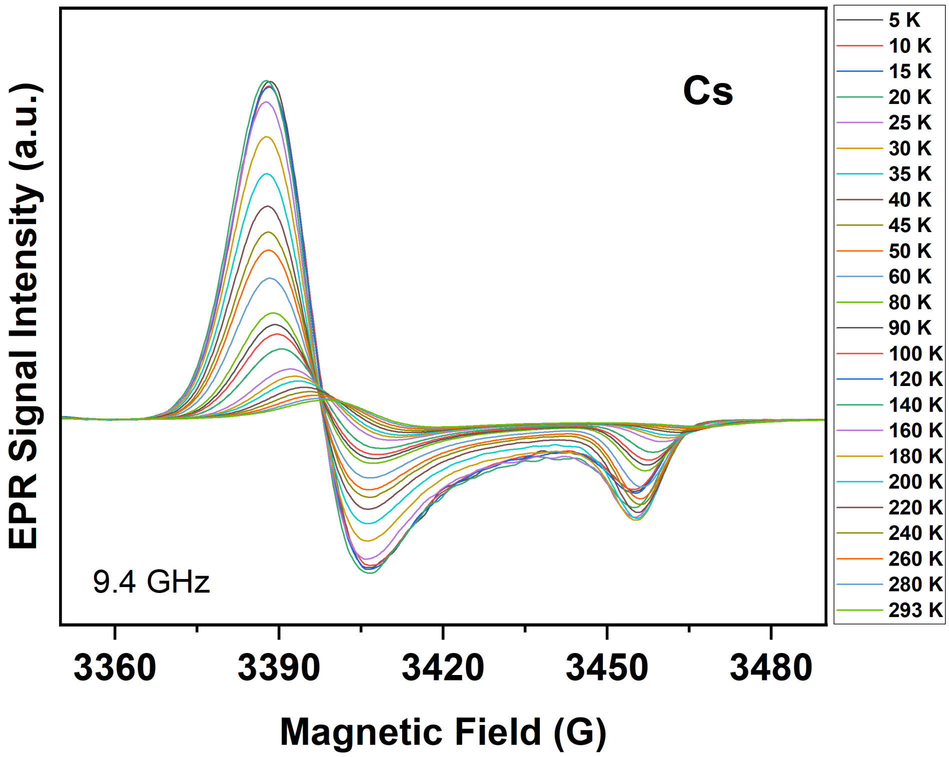

Supplementary Figure 100. X-band (9.4 GHz) EPR spectra of a powdered sample of **4Cs**, measured at varied temperatures between 5 and 293 K, under modulation frequency of 100 kHz, modulation amplitude of 2 G, and microwave attenuation of 25 dB.

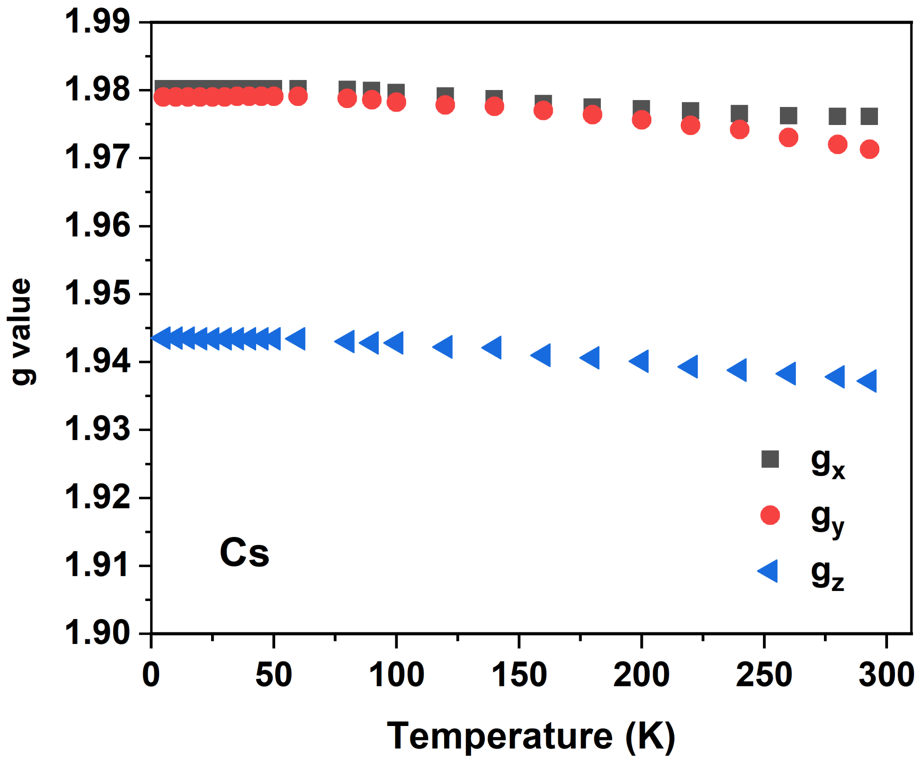

Supplementary Figure 101. Temperature dependence of the extracted *g*-values for **4Cs**.

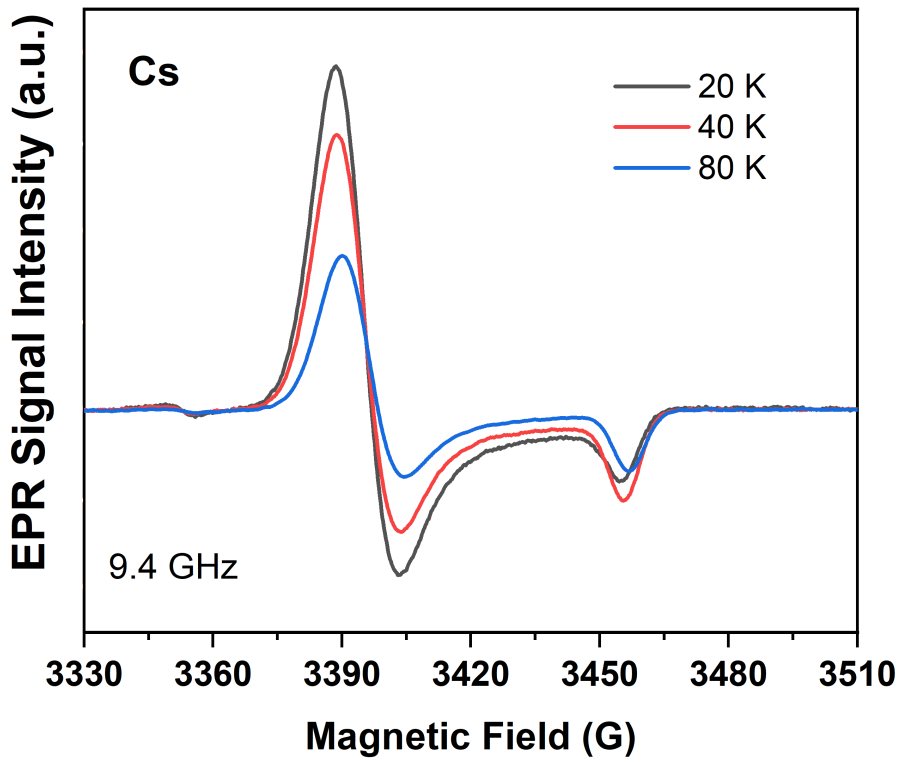

Supplementary Figure 102. X-band (9.4 GHz) EPR spectra of a frozen solution (DME:Toluene. 95:5, 3mM) of **4Cs** measured at 20, 40 and 80 K (attenuation, 20 dB; modulation frequency, 100 kHz; modulation amplitude, 2 G).

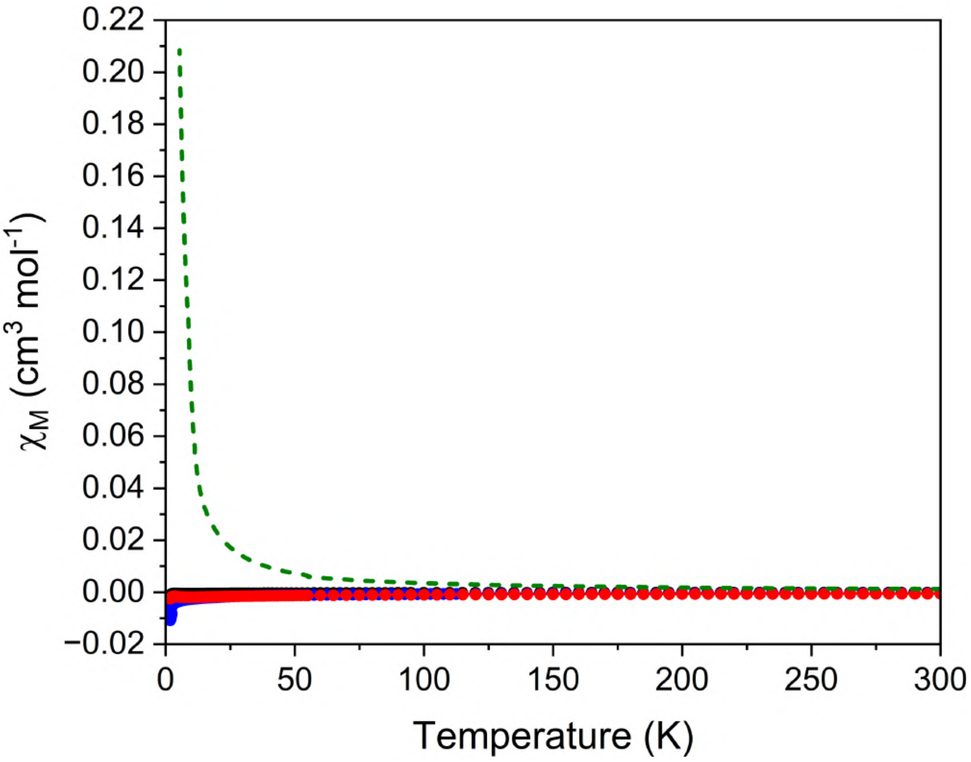

Supplementary Figure 103. Comparative variable-temperature SQUID magnetometry of **4K** (blue), **4Rb** (red), and **4Cs** (black) at 1 T plotting χ_M (cm^3 mol^{-1}) vs Temperature (K) over the temperature range 1.8 to 300 K. The green dashed lines represent the theoretical plot for a d^1 S = 1/2 (g = 2) system.

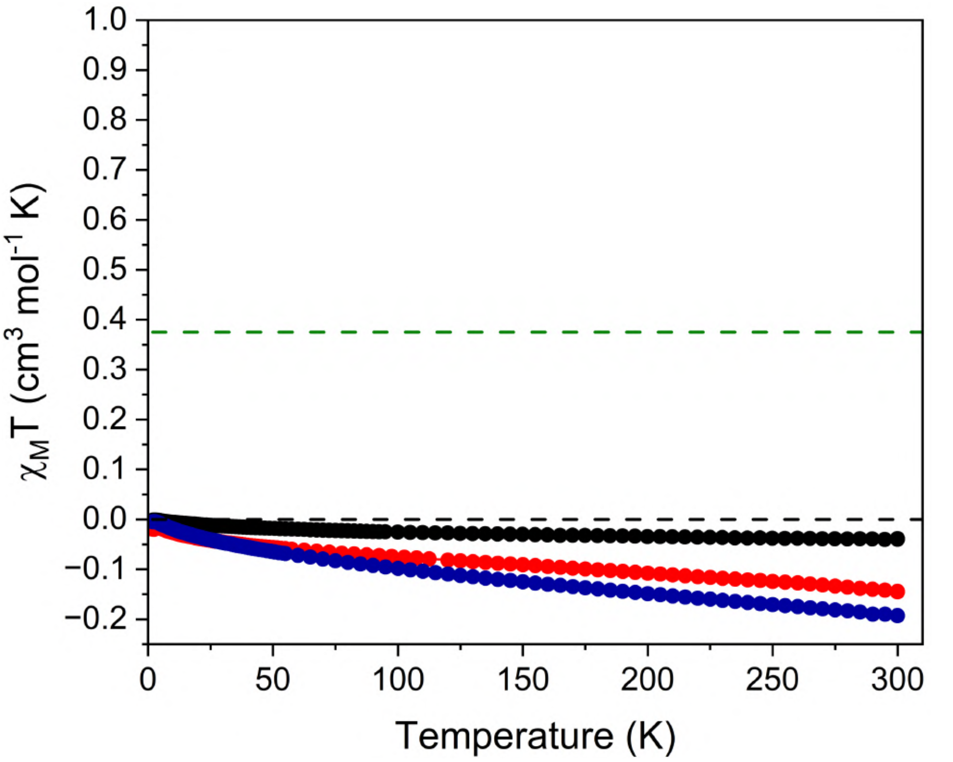

Supplementary Figure 104. Comparative variable-temperature SQUID magnetometry of **4K** (blue), **4Rb** (red), and **4Cs** (black) at 1 T plotting $\chi_M T$ (cm^3 mol^{-1} K) vs Temperature (K) over the temperature range 1.8 to 300 K. The green dashed lines represent the theoretical plot for a d^1 S = 1/2 (g = 2) system. The black dashed line is a guide to the eye only for 0 cm^3 mol^{-1} K.

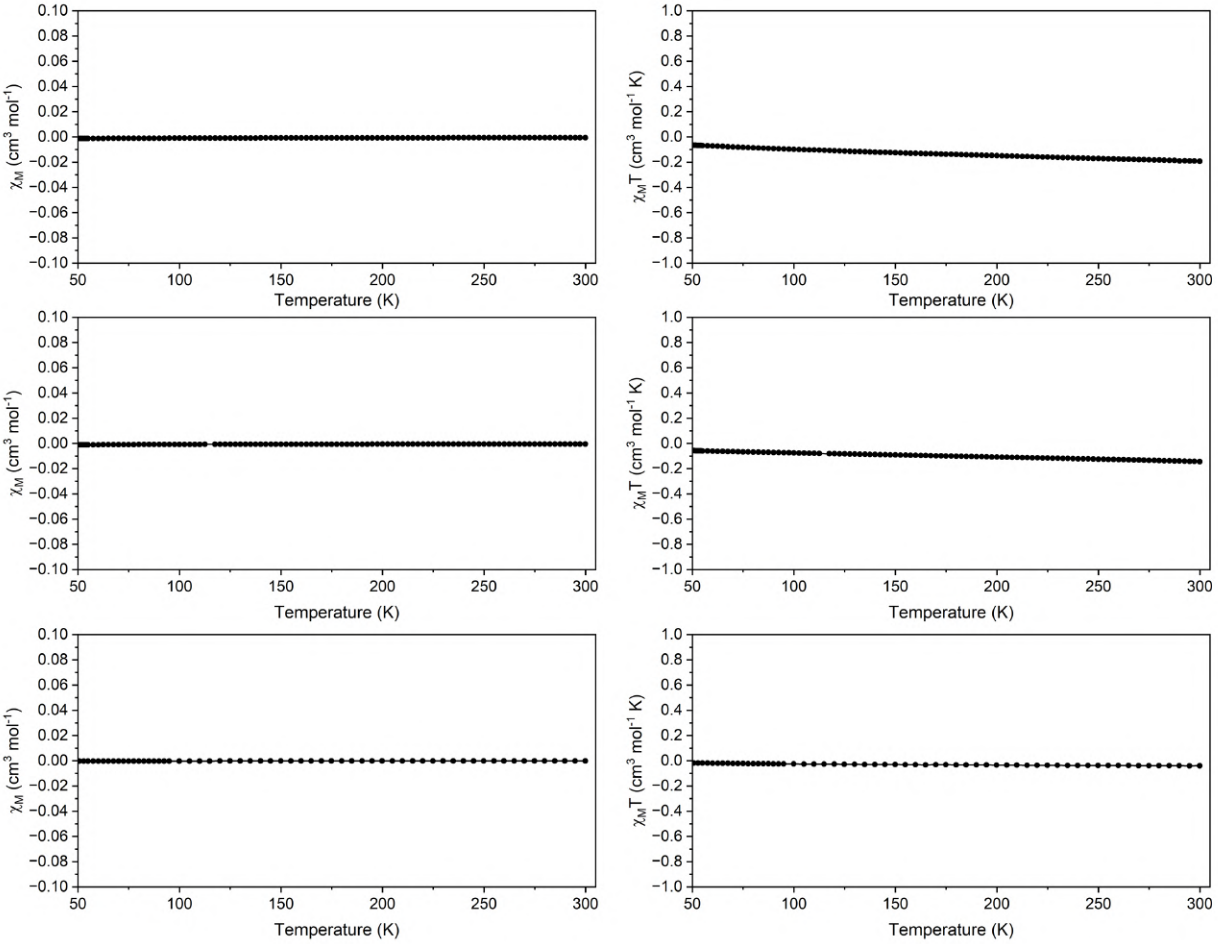

Supplementary Figure 105. Variable-temperature SQUID magnetometry of **4K** (top), **4Rb** (middle), and **4Cs** (bottom) at 1 T plotting χ_M (cm^3 mol^{-1}) vs Temperature (K) over the temperature range 50 to 300 K (left) and χ_MT (cm^3 mol^{-1} K) vs Temperature (K) over the temperature range 50 to 300 K (right).

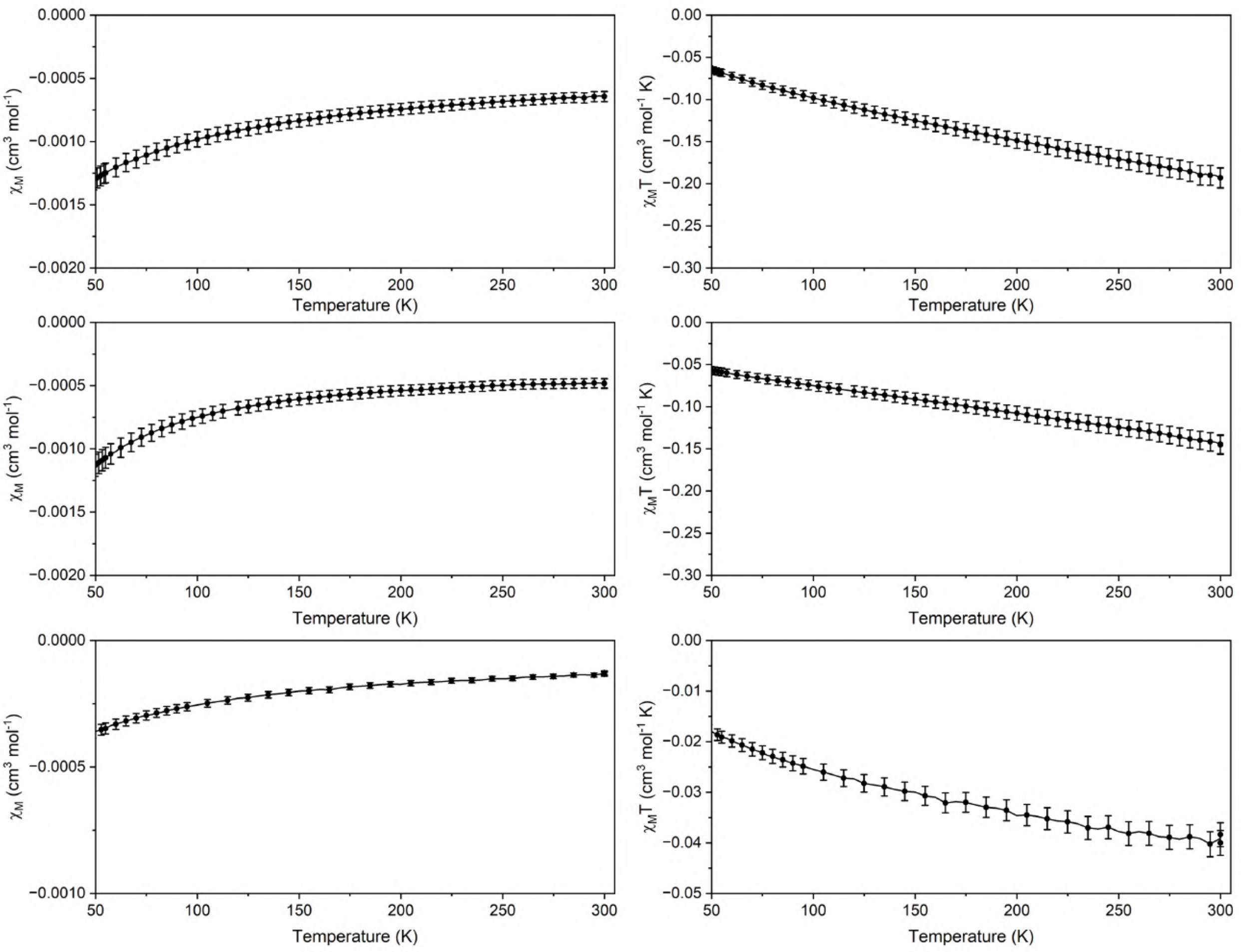

Supplementary Figure 106. Variable-temperature SQUID magnetometry of **4K** (top: average error = 6.16%), **4Rb** (middle: average error = 7.72%), and **4Cs** (bottom: average error = 6.15%) at 1 T plotting χ_M ($cm^3\ mol^{-1}$) vs Temperature (K) over the temperature range 50 to 300 K (left) and $\chi_M T$ ($cm^3\ mol^{-1}\ K$) vs Temperature (K) over the temperature range 50 to 300 K (right). **NOTE** – the shortened temperature range is due to the large number of data points below 50 K which make it very difficult to visualize errors bars effectively. Errors derive from the standard deviation of the sampling distribution of the points used (sample population divided by the square root of the number of samples) to compute the reported moment represented as a percentage.

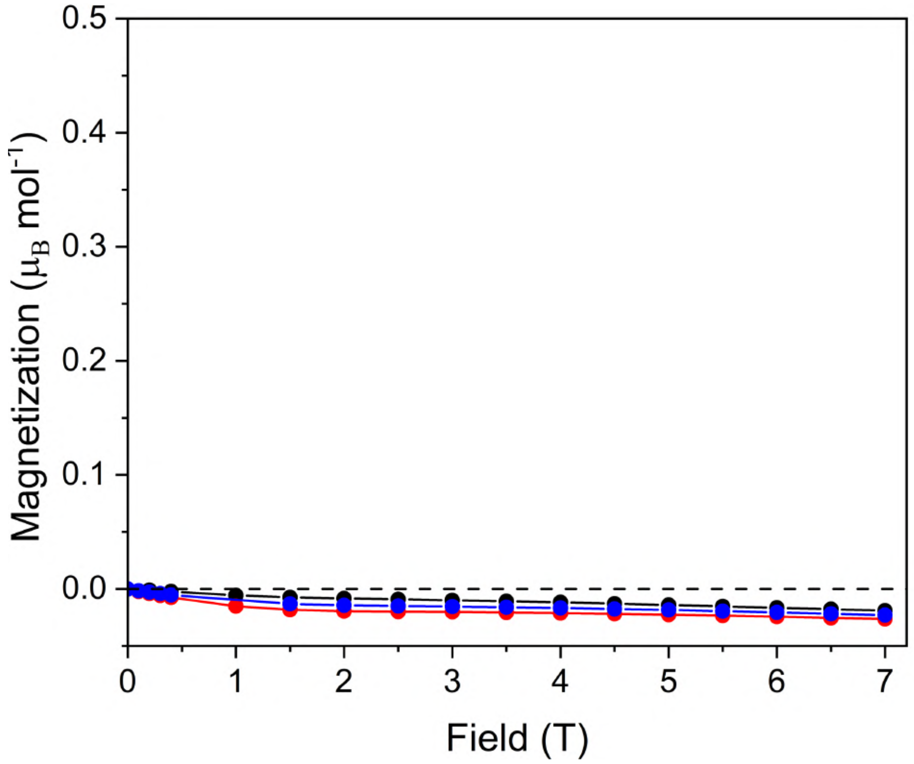

Supplementary Figure 107. Comparative Isothermal Magnetization vs Field data for **4K** (blue), **4Rb** (red), and **4Cs** (black) at 1.8 K from 0-7 Tesla.

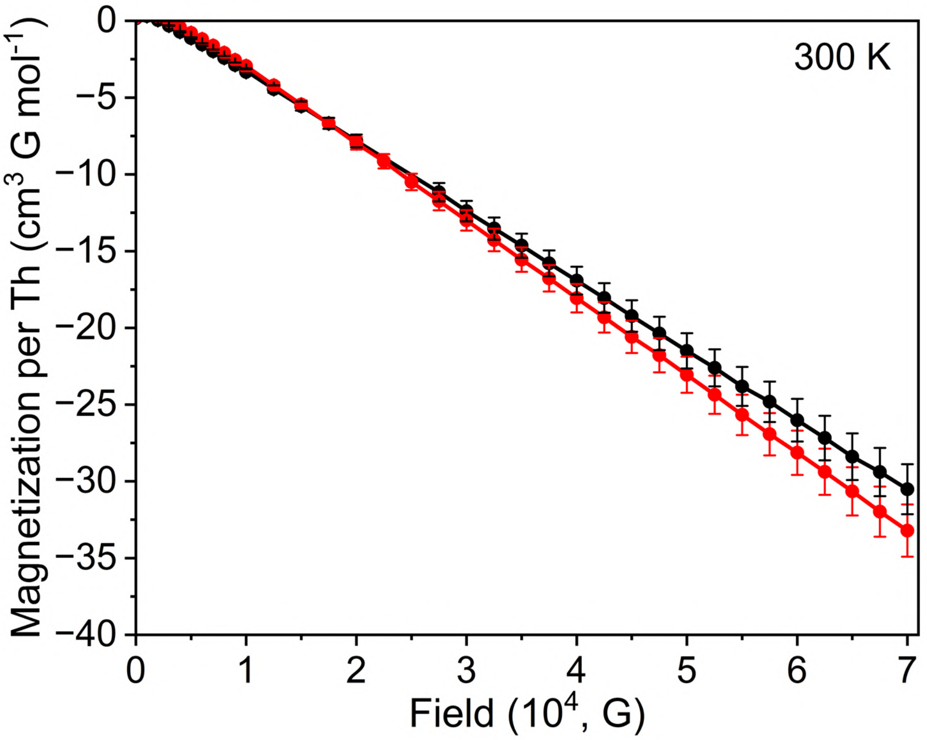

Supplementary Figure 108. Comparative Magnetization vs Field data for **4Cs** (black: average error = 5.34 %) and **4Cs-eico** (red: average error = 5.12 %) at 300 K from 0-7 Tesla. The straight lines are linear regressions of the data points. On a per molecule basis, χ_D = –1400 x10^{-6} cm^3 mol^{-1} (r^2 = 0.999) for **4Cs** and –1480 x 10^{-6} cm^3 mol^{-1} (r^2 = 0.999) for **4Cs-eico** which is within 5.40% of each other. Errors derive from the standard deviation of the sampling distribution of the points used (sample population divided by the square root of the number of samples) to compute the reported moment represented as a percentage.

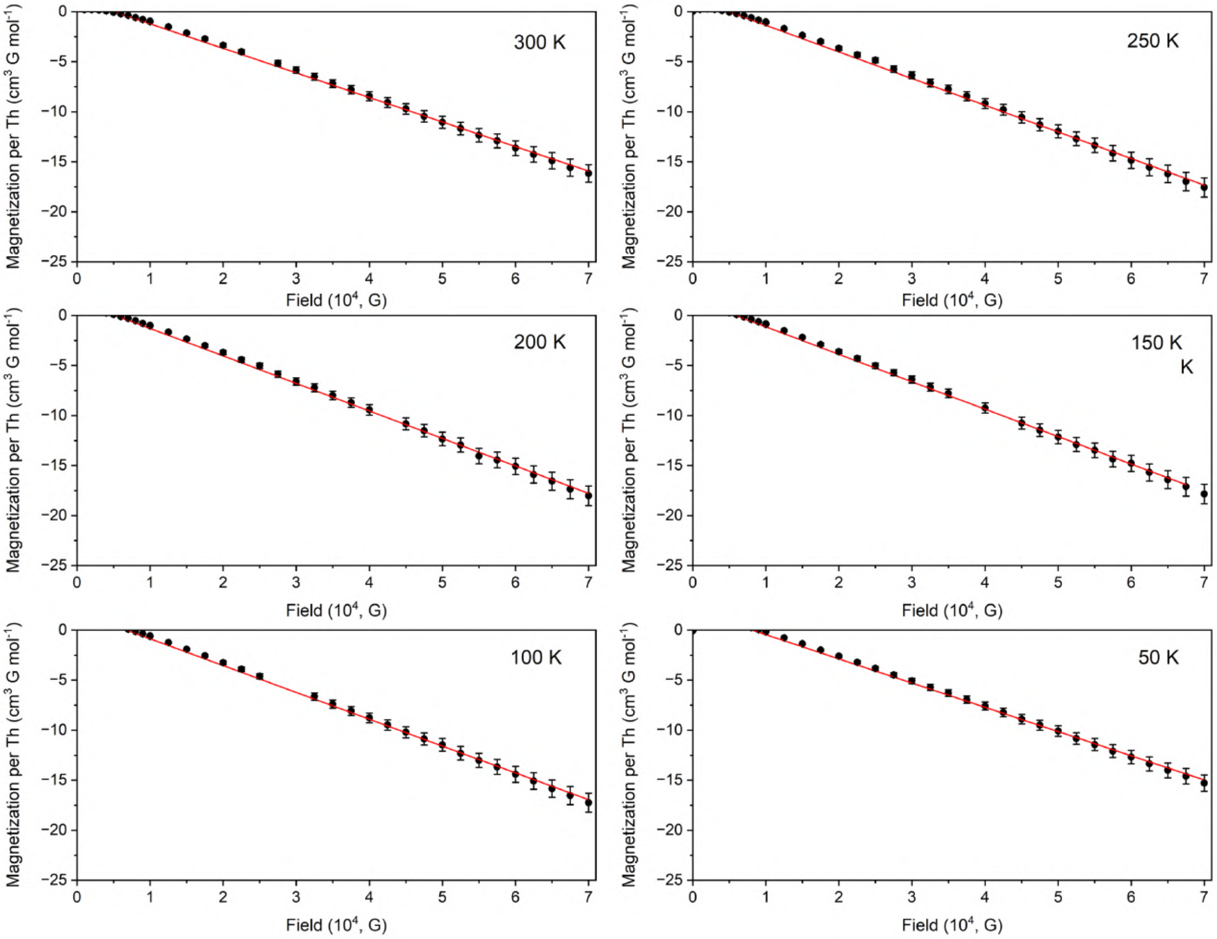

Supplementary Figure 109. Magnetization vs Field data for **1** at 300 (top left: r^2 = 0.997; average error = 5.44%), 250 (top right: r^2 = 0.998; average error = 5.45%), 200 (middle left: r^2 = 0.997; average error = 5.45%), 150 (middle right: r^2 = 0.998; average error = 5.46%), 100 (bottom left: r^2 = 0.997; average error = 5.50%) and 50 (bottom right: r^2 = 0.998; average error = 5.27%) K from 0-7 Tesla. The straight lines are linear regressions of the data points. Missing field measurements are due to measurement error. Errors derive from the standard deviation of the sampling distribution of the points used (sample population divided by the square root of the number of samples) to compute the reported moment represented as a percentage.

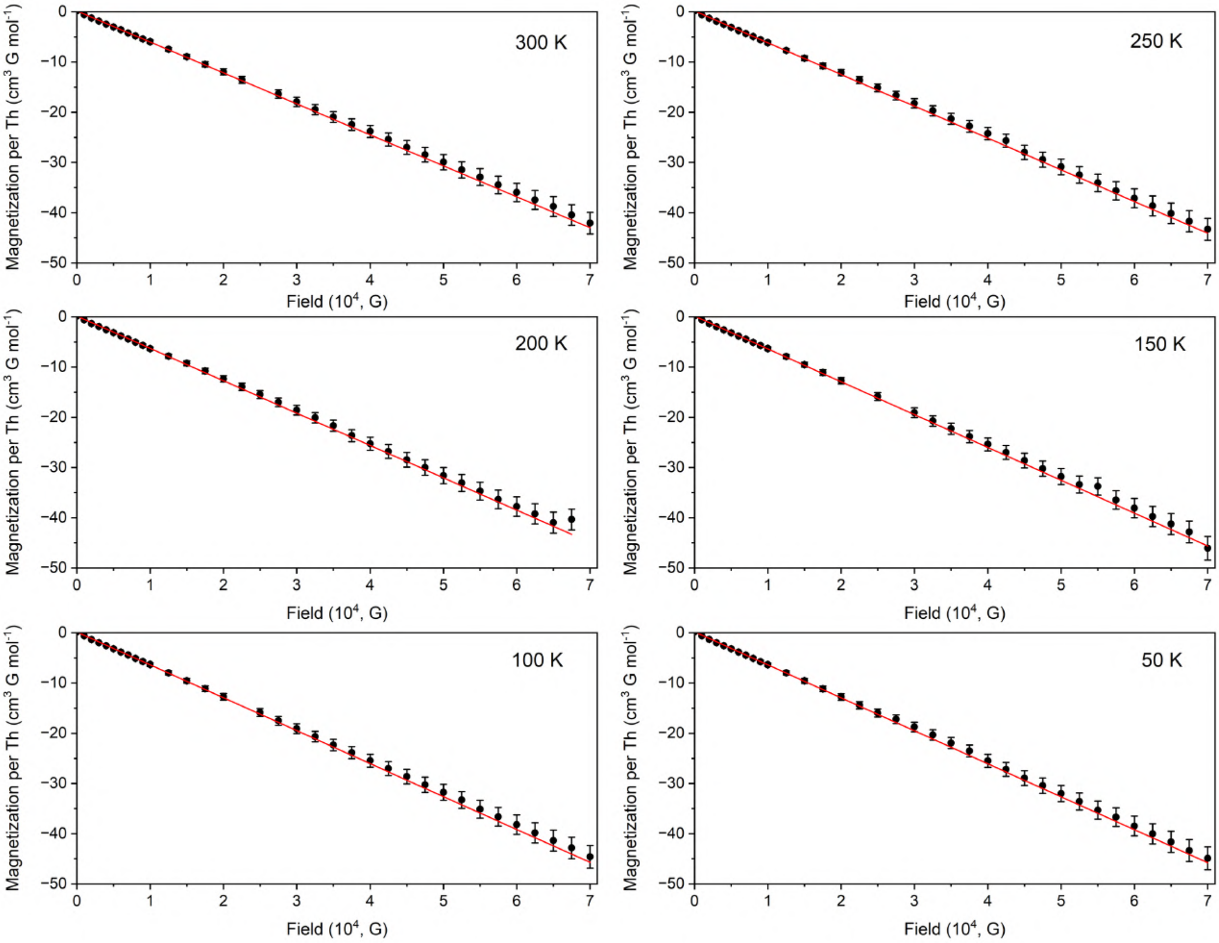

Supplementary Figure 110. Magnetization vs Field data for **4K** at 300 (top left: r^2 = 0.999; average error = 5.10%), 250 (top right: r^2 = 0.999; average error = 5.08%), 200 (middle left: r^2 = 0.999; average error = 5.13%), 150 (middle right: r^2 = 0.999; average error = 5.07%), 100 (bottom left: r^2 = 0.999; average error = 5.06%) and 50 (bottom right: r^2 = 0.999; average error = 5.05%) K from 0-7 Tesla. The straight lines are linear regressions of the data points. Missing field measurements are due to measurement error. Errors derive from the standard deviation of the sampling distribution of the points used (sample population divided by the square root of the number of samples) to compute the reported moment represented as a percentage.

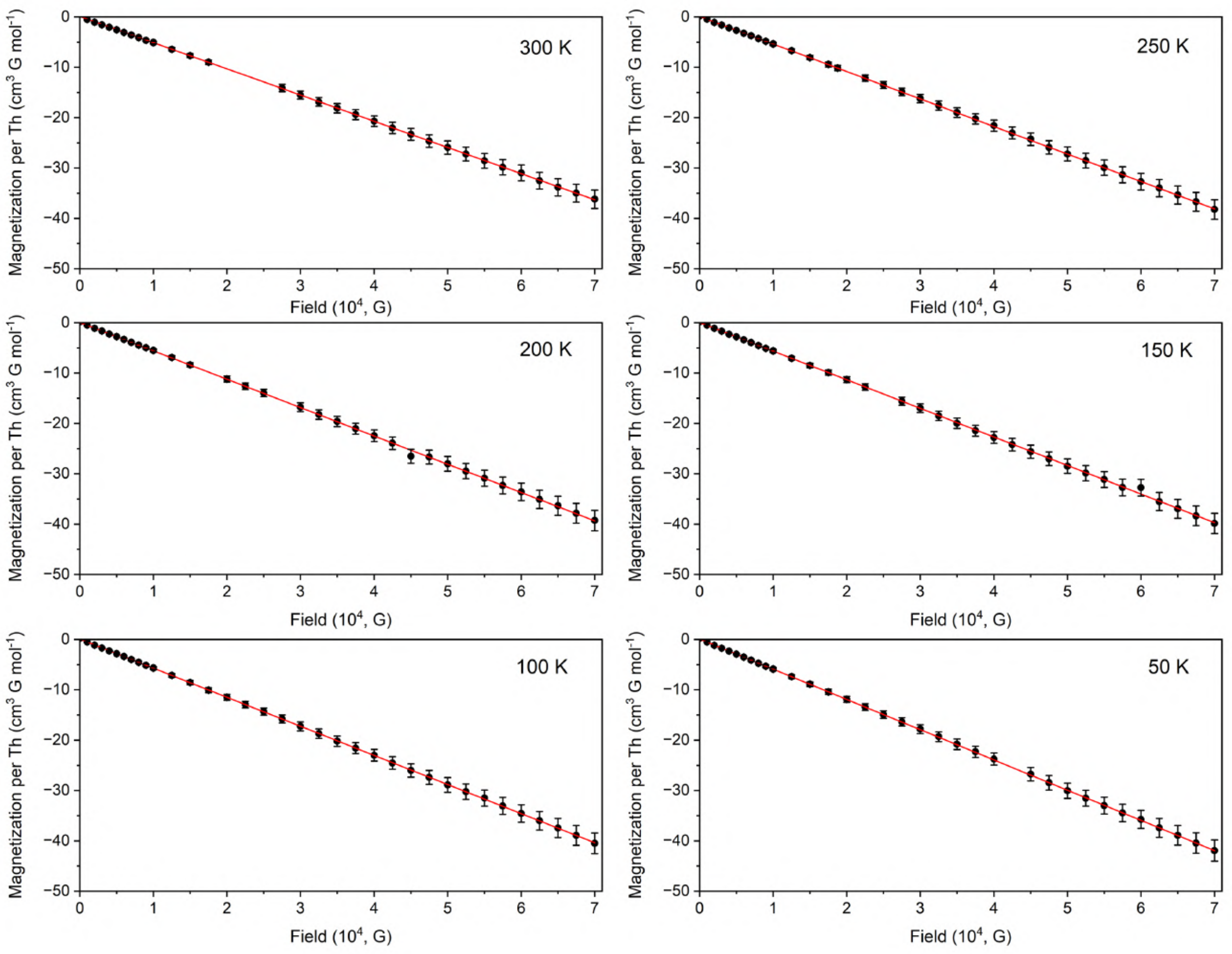

Supplementary Figure 111. Magnetization vs Field data for **4Rb** at 300 (top left: $r^2 = 0.999$; average error = 5.11%), 250 (top right: $r^2 = 0.999$; average error = 5.10%), 200 (middle left: $r^2 = 0.999$; average error = 5.18%), 150 (middle right: $r^2 = 0.999$; average error = 5.08%), 100 (bottom left: $r^2 = 0.999$; average error = 5.07%) and 50 (bottom right: $r^2 = 0.999$; average error = 5.02%) K from 0-7 Tesla. The straight lines are linear regressions of the data points. Missing field measurements are due to measurement error. Errors derive from the standard deviation of the sampling distribution of the points used (sample population divided by the square root of the number of samples) to compute the reported moment represented as a percentage.

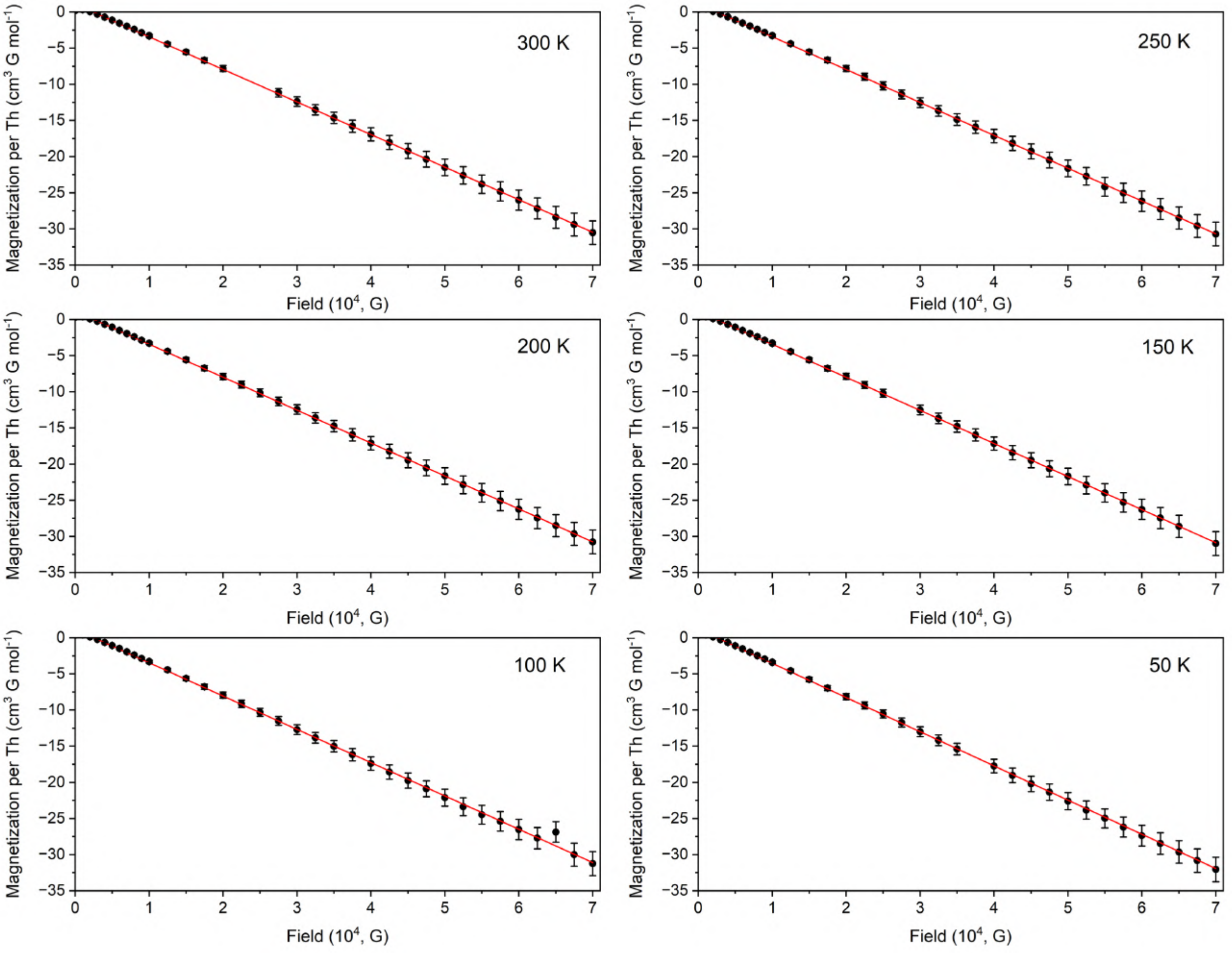

Supplementary Figure 112. Magnetization vs Field data for **4Cs** at 300 (top left: $r^2 = 0.999$; average error = 5.34%), 250 (top right: $r^2 = 0.999$; average error = 5.35%), 200 (middle left: $r^2 = 0.999$; average error = 5.33%), 150 (middle right: $r^2 = 0.999$; average error = 5.32%), 100 (bottom left: $r^2 = 0.999$; average error = 5.30%) and 50 (bottom right: $r^2 = 0.999$; average error = 5.26%) K from 0-7 Tesla. The straight lines are linear regressions of the data points. Missing field measurements are due to measurement error. Errors derive from the standard deviation of the sampling distribution of the points used (sample population divided by the square root of the number of samples) to compute the reported moment represented as a percentage.

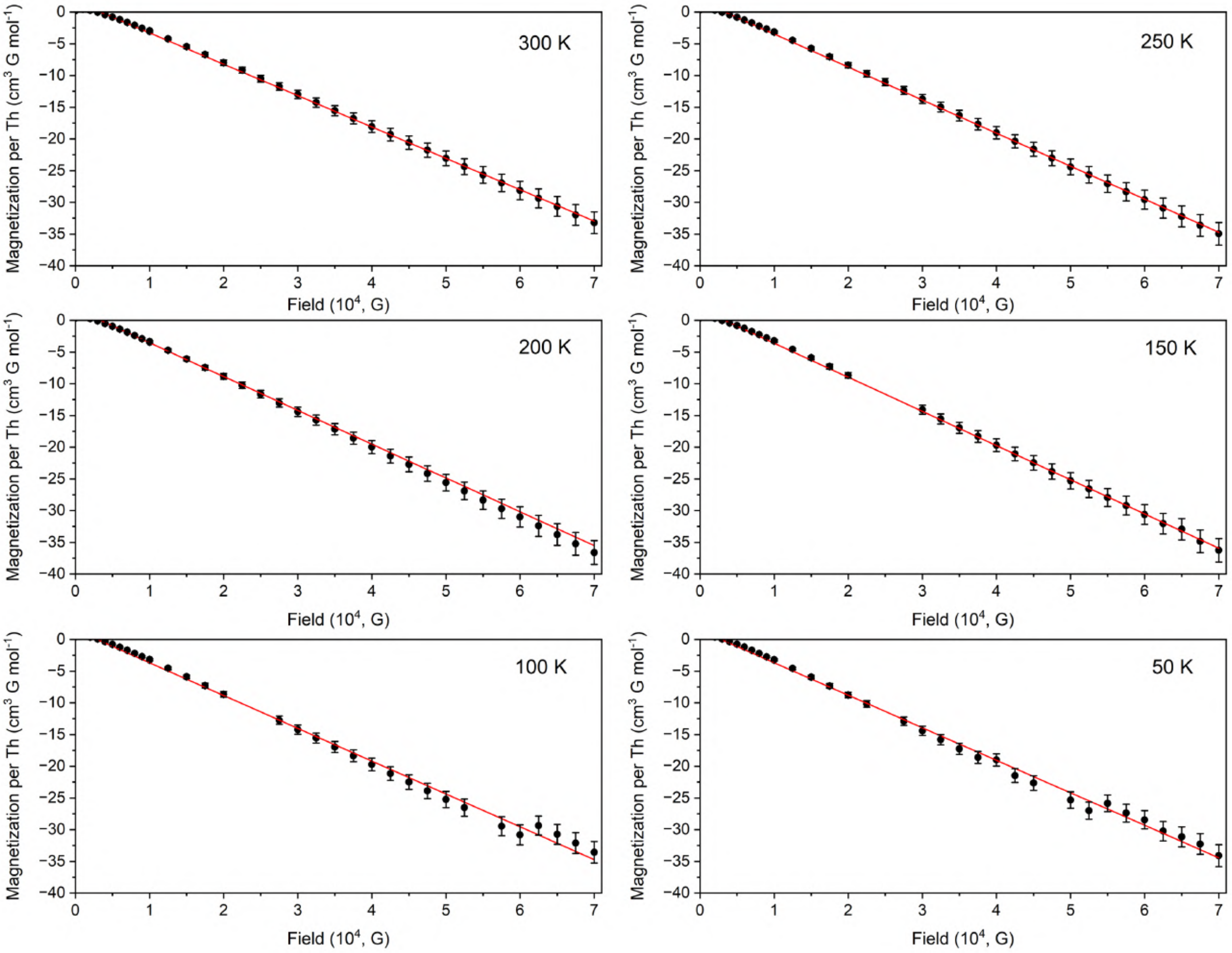

Supplementary Figure 113. Magnetization vs Field data for **4Cs-eico** at 300 (top left: r^2 = 0.999; average error = 5.12%), 250 (top right: r^2 = 0.999; average error = 5.11%), 200 (middle left: r^2 = 0.999; average error = 5.11%), 150 (middle right: r^2 = 0.999; average error = 5.10%), 100 (bottom left: r^2 = 0.996; average error = 5.09%) and 50 (bottom right: r^2 = 0.997; average error = 5.07%) K from 0-7 Tesla. The straight lines are linear regressions of the data points. Missing field measurements are due to measurement error. Errors derive from the standard deviation of the sampling distribution of the points used (sample population divided by the square root of the number of samples) to compute the reported moment represented as a percentage.

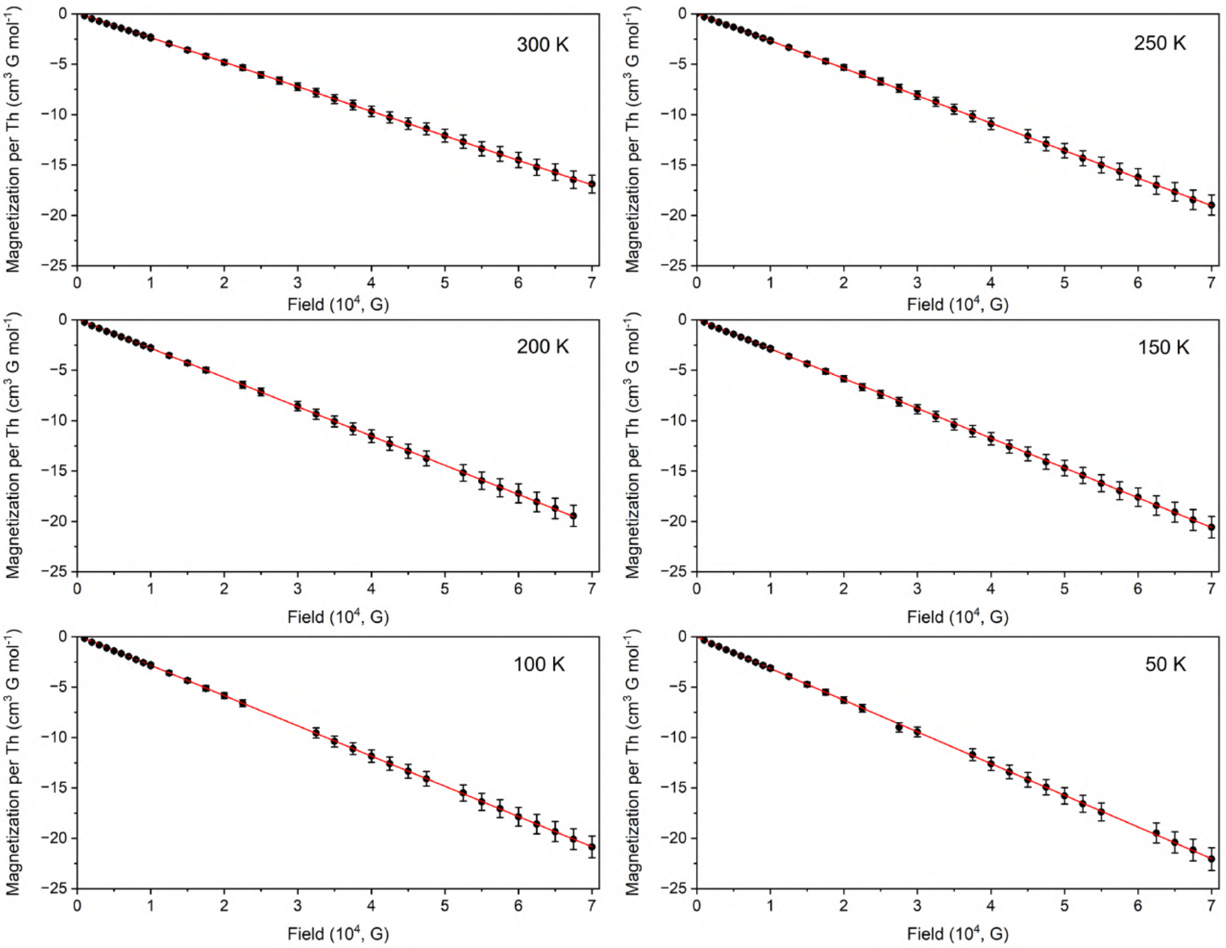

Supplementary Figure 114. Magnetization vs Field data for **5** at 300 (top left: $r^2 = 0.999$; average error = 5.25%), 250 (top right: $r^2 = 0.999$; average error = 5.23%), 200 (middle left: $r^2 = 0.999$; average error = 5.41%), 150 (middle right: $r^2 = 0.999$; average error = 5.21%), 100 (bottom left: $r^2 = 0.999$; average error = 5.18%) and 50 (bottom right: $r^2 = 0.999$; average error = 5.11%) K from 0-7 Tesla. The straight lines are linear regressions of the data points. Missing field measurements are due to measurement error. Errors derive from the standard deviation of the sampling distribution of the points used (sample population divided by the square root of the number of samples) to compute the reported moment represented as a percentage.

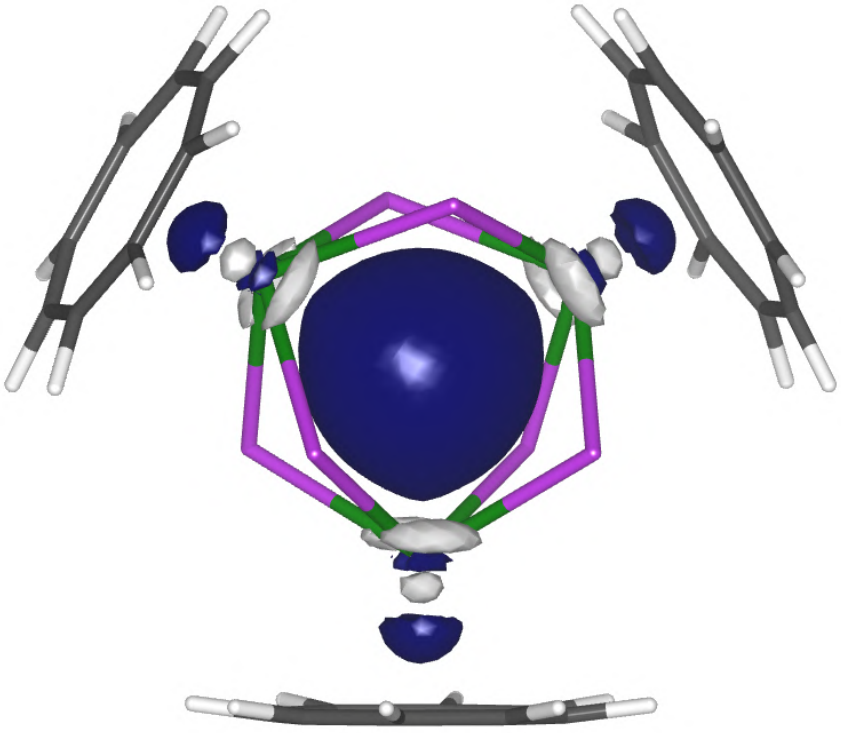

Supplementary Figure 115. The AdNDP (ON = 0.92) of **4'** plotted with an isovalue of 0.05 a.u.

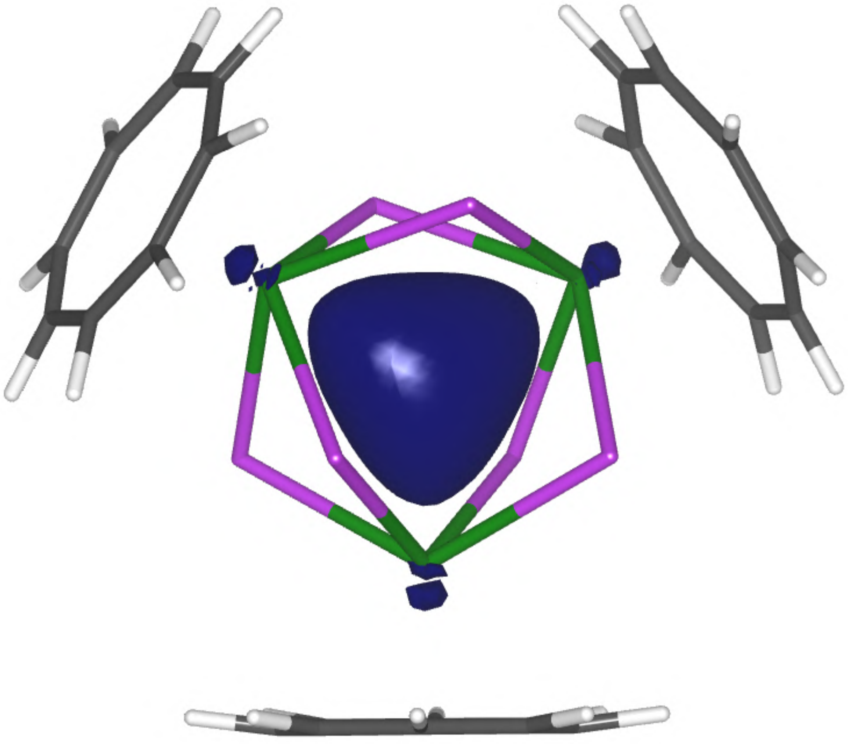

Supplementary Figure 116. The $EDDB_F$ of **4'** plotted with an isovalue of 0.003 a.u.

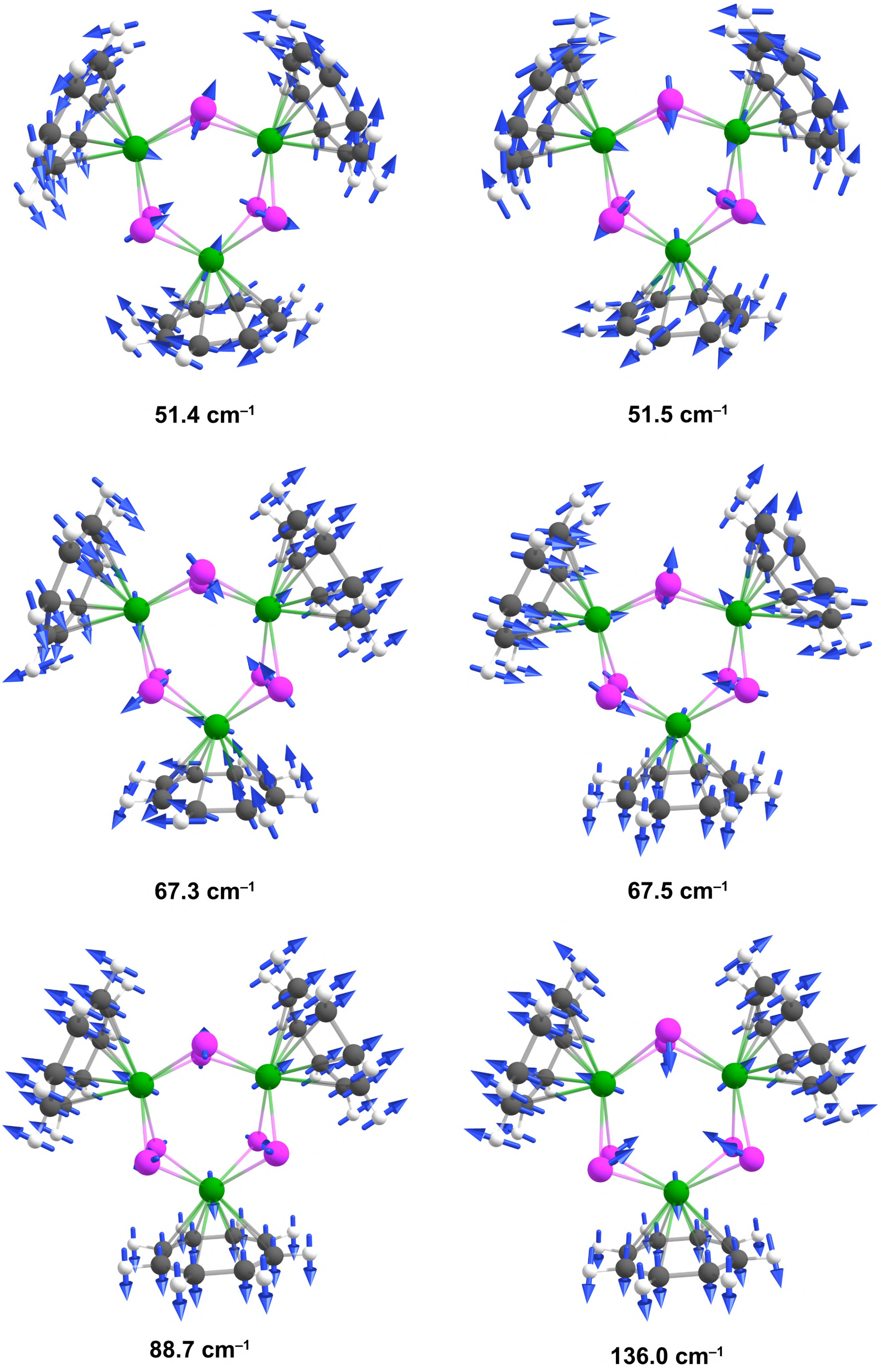

Supplementary Figure 117. Displacement vectors for six selected vibration modes below 150 cm^{-1} involving the Th_3 core with strong Raman activities for **4'**.

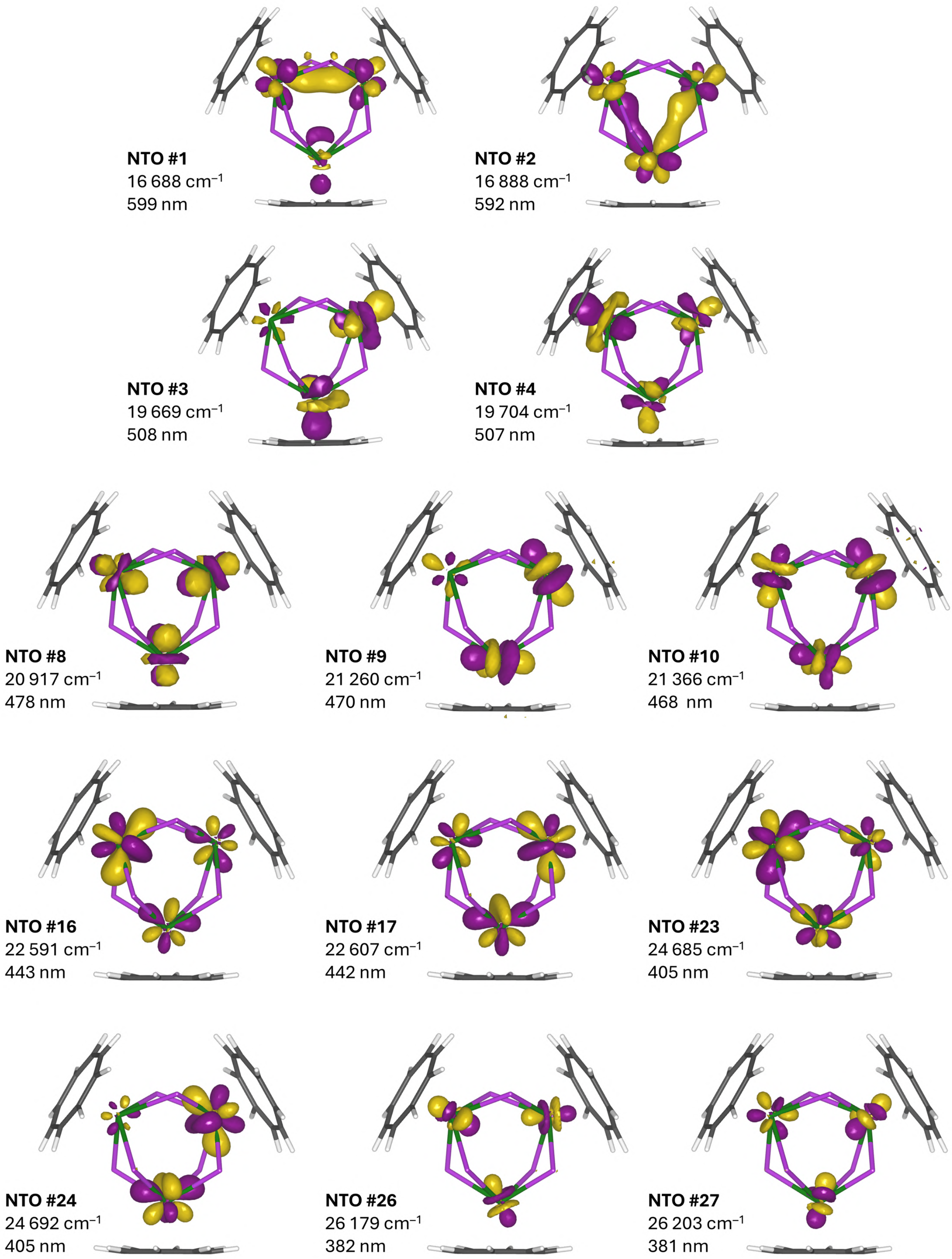

Supplementary Figure 118. Calculated final NTOs for **4'** plotted at isovalue 0.05 a.u. The excitations were started from the HOMO. Obtained from ORCA PBE0 gas phase calculation.

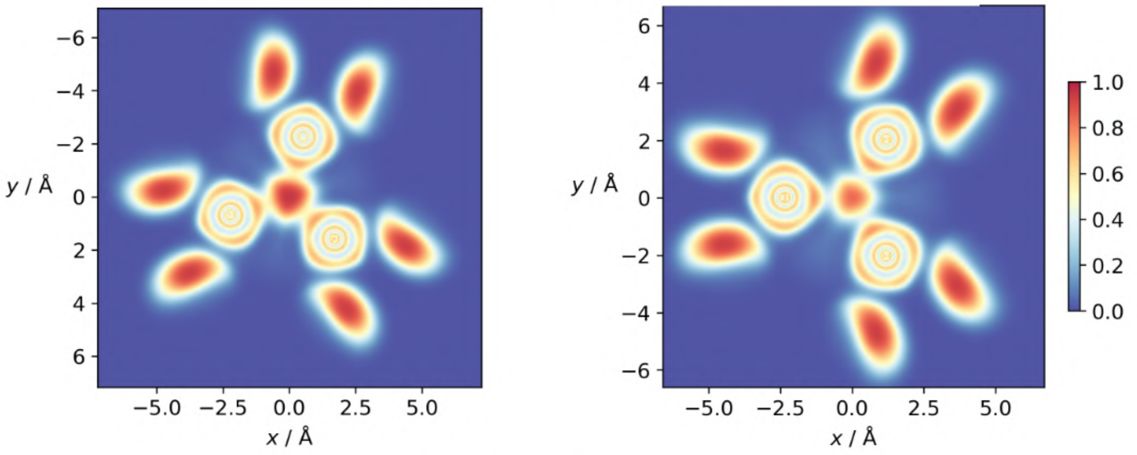

Supplementary Figure 119. Comparison of the ELF plots for **3'** (left) and **4'** (right).

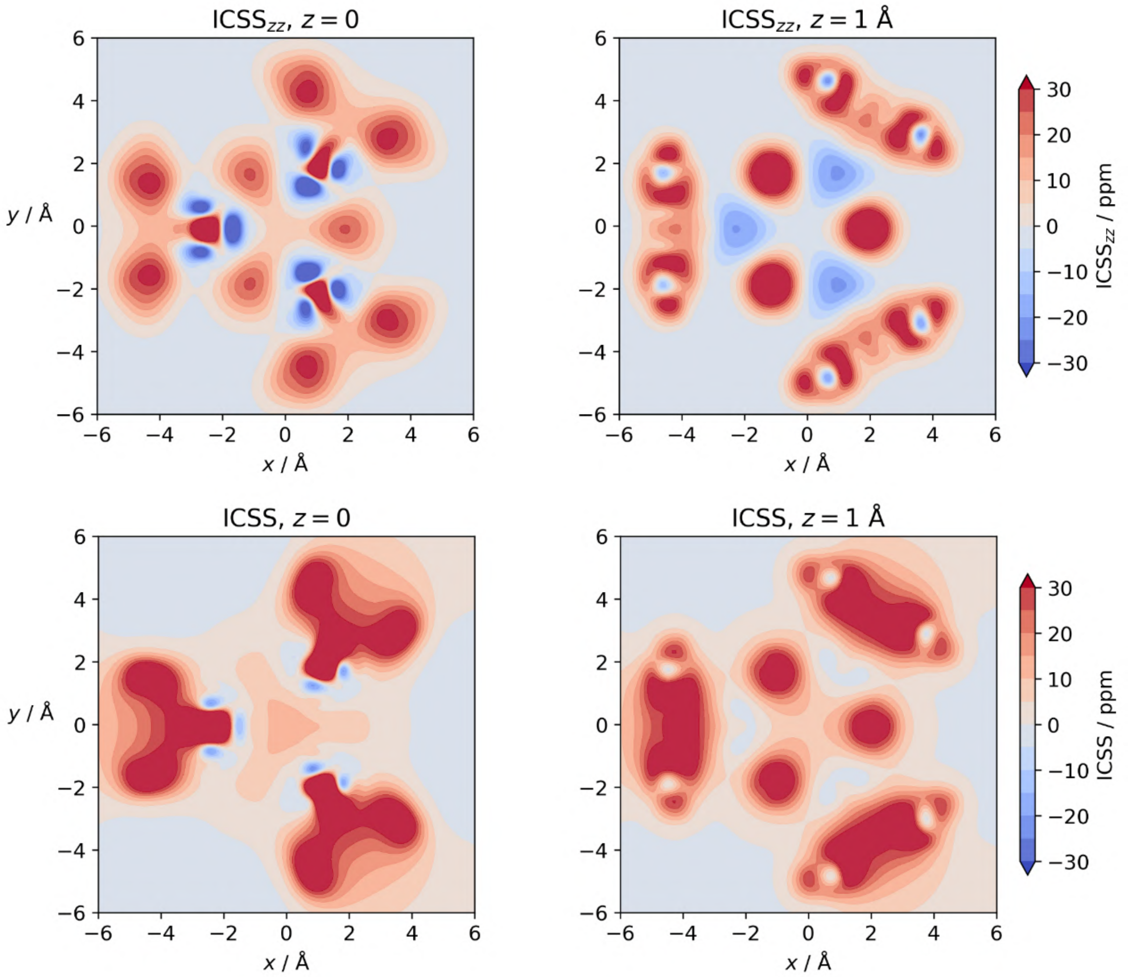

Supplementary Figure 120. The ICSS$_{zz}$ (upper) and ICSS (lower) of for **4'** in the *xy*-plane containing the Th_3 core (left) and 1 Å above the *xy*-plane (right). The red region corresponds to areas of shielding and the blue deshielding.

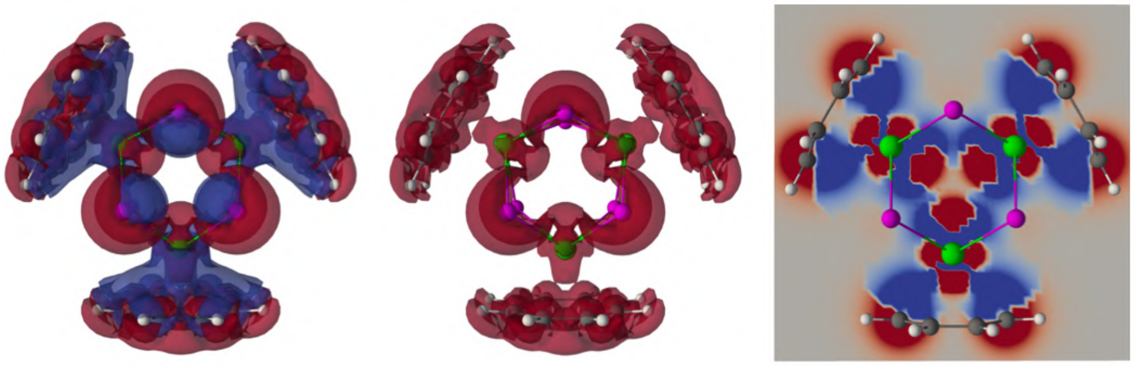

Supplementary Figure 121. Left - Modulus of the current induced densities of **4'** plotted at isovalue of 0.025 a.u. showing diatropic (red) and paratropic (blue) components; Middle - Modulus of the current induced densities of **4'** plotted at isovalue of 0.025 a.u. showing only the diatropic (red) component; Right - *xz* plane ($z = 0$) of the modulus of the current induced density of **4'** indicating the delocalized nature of the unpaired electron.

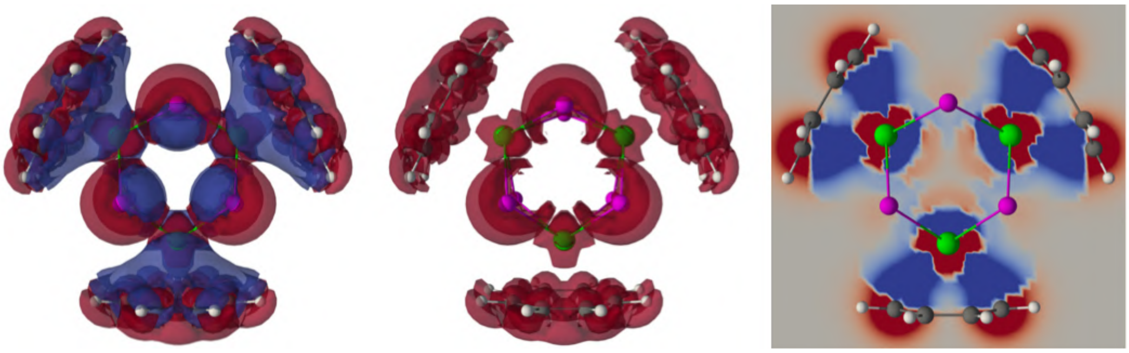

Supplementary Figure 122. Left - Modulus of the current induced densities of **6** plotted at isovalue of 0.025 a.u. showing diatropic (red) and paratropic (blue) components; Middle - Modulus of the current induced densities of **6** plotted at isovalue of 0.025 a.u. showing only the diatropic (red) component; Right – *xy*-plane of the current induced density of **6** demonstrating the absence of the unpaired electron in the center of the Th_3 triangle compared to **4'**.

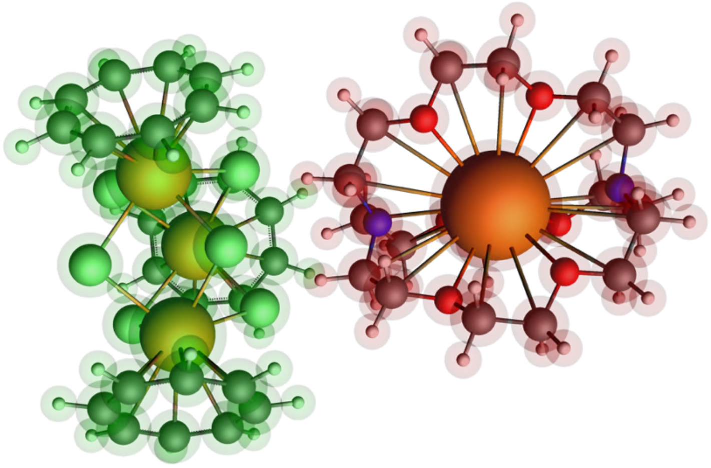

Supplementary Figure 123. The two regions used in the EDA calculation, highlighted in red ($[Cs(2.2.2\text{-cryptand})]^+$) and green ($[\{(\eta^8\text{-}C_8H_8)Th(\mu\text{-}Cl)_2\}_3]^-$) colours.

Supplementary Tables

Supplementary Table 1. Crystallographic details for **4K**, **4Rb**, **4Cs**, and **5**.

	**4K**	**4Rb**	**4Cs**	**5**
Formula	$C_{42}H_{60}Cl_6KN_2O_6Th_3$	$C_{50}H_{76}Cl_6N_2O_8RbTh_3$	$C_{50}H_{76}Cl_6CsN_2O_8Th_3$	$C_{40}H_{64}Cl_6Cs_2O_9Th_3$
Fw, g mol^{-1}	1636.84	1827.41	1874.85	1863.55
Cryst size, mm	0.26 × 0.169 × 0.099	0.412 × 0.367 × 0.246	0.193 × 0.165 × 0.101	0.237 × 0.119 × 0.078
Crystal system	monoclinic	triclinic	triclinic	monoclinic
Space group	$P2_1$/n	P-1	P-1	$P2_1$/n
Temperature (K)	150(2)	100(2)	150(2)	100(2)
a, (Å)	16.9305(3)	14.0134(2)	14.0480(3)	12.5021(2)
b, (Å)	18.8866(3)	14.9677(2)	14.9640(3)	16.5213(2)
c, (Å)	17.1190(3)	17.0175(2)	17.0625(3)	26.3442(4)
α, (°)	90	113.8150(10)	113.925(2)	90
β, (°)	108.457(2)	97.3240(10)	97.311(2)	96.7450(10)
γ, (°)	90	106.6550(10)	106.623(2)	90
V, (Å^3)	5192.39(16)	3005.62(7)	3017.97(12)	5403.76(14)
Z	4	2	2	4
ρ_{calc} g cm^{-3}	2.094	2.019	2.063	2.291
μ, mm^{-1}	31.340	27.519	8.282	39.810
Reflections measured	47376	37837	44297	37906
Unique reflections, R_{int}	9163, 0.1526	10979, 0.1404	15326, 0.0357	10201, 0.0777
Reflections with $F^2 > 2\sigma(F^2)$	6775	10491	12290	9320
Transmission range	0.20627-1.00000	0.017-0.488	0.532-1.000	0.029-0.267
R, R_w[a] ($F^2 > 2s(F^2)$)	0.1032, 0.2565	0.0926, 0.2372	0.0303, 0.0656	0.0791, 0.2022
R, R_w[a] (all data)	0.1287, 0.2774	0.0945, 0.2400	0.0452, 0.0699	0.0842, 0.2053
S[a]	1.043	1.050	1.038	1.073
Parameters, Restraints	687, 1385	704, 664	704, 461	680, 936
Max.,min. diff. map, e Å^{-3}	9.352, –3.989	6.490, –5.163	1.621, –1.691	6.195, –2.155

[a] Conventional $R = \Sigma||F_o| - |F_c||/\Sigma|F_o|$; $R_w = [\Sigma w(F_o^2 - F_c^2)^2/\Sigma w(F_o^2)^2]^{1/2}$; $S = [\Sigma w(F_o^2 - F_c^2)^2/\text{no. data} - \text{no. params})]^{1/2}$ for all data.

Supplementary Table 2. Unit cell values obtained from PXRD Le Bail refinement results for **4M** with those expected from SCXRD data.

**Entry**	**4K**	**4K**	**4Rb**	**4Rb**	**4Cs**	**4Cs**
**Method**	**SCXRD**	**PXRD**	**SCXRD**	**PXRD**	**SCXRD**	**PXRD**
a, (Å)	16.9305(3)	16.8655(11)	14.0134(2)	13.9936(7)	14.0480(3)	14.059(2)
b, (Å)	18.8866(3)	18.863(2)	14.9677(2)	14.9964(8)	14.9640(3)	15.010(3)
c, (Å)	17.1190(3)	17.029(2)	17.0175(2)	17.1481(12)	17.0625(3)	16.984(4)
α, (°)	90	90	113.8150(10)	115.365(4)	113.925(2)	114.45(14)
β, (°)	108.457(2)	108.398(11)	97.3240(10)	95.509(5)	97.311(2)	97.634(17)
γ, (°)	90	90	106.6550(10)	107.379(5)	106.623(2)	105.389(17

Supplementary Table 3. Selected experimental parameters for the Raman spectroscopic measurements.

Entry	[Th(η^8-C_8H_8)$_2$]	**1**	**4K**	**4Rb**	**4Cs**	**5**
λ (nm)	785	638	785	785	785	532
Power (mW)	1.5	1.5	1.5	1.5	1.5	1.5
Acquisition time (s)	10	1	15	25	1	5
Accumulations	1	10	20	40	50	5
Grating (gr/mm)	600	600	600	600	600	600
Filter (%)	10	50	50	50	50	50
Slit (μm)	100	100	100	100	100	100
Hole (μm)	300	300	300	300	300	300

Supplementary Table 4. Selected UV/Vis/NIR experimental details and data for [Th(η^8-C_8H_8)$_2$], **1**, **4M**, and **5**.

Entry	[Th(η^8-C_8H_8)$_2$]	**1**	**4K**	**4Rb**	**4Cs**	**5**
Solvent	DME	DME	DME	DME	DME	DME
Molarity (mM)	15	9	1.5	0.79	0.44	5.4
ν_{max} (cm^{-1})	-	-	15964.2	15873.0	15933.7	-
λ_{max} (nm)	-	-	626.4	630.0	627.6	-
ε_{max} (M^{-1} cm^{-1})	-	-	1550.8	2158.9	1176.6	-
$\Delta\nu_{1/2}$ (cm^{-1})	-	-	2219.4	2117.6	2217.2	-
$\nu°_{1/2}$ (cm^{-1})	-	-	6072	6055	6067	-
Γ[a]	-	-	0.63	0.65	0.64	-

Supplementary Table 5. Extracted *g*-parameters for **4K** from the simulation of X-band powder spectra recorded at 9.4 GHz and 6-270 K.

**Temperature (K)**	g_x	g_y	g_z
6	1.9799	1.9793	1.9435
10	1.9799	1.9793	1.9435
15	1.9799	1.9793	1.9435
20	1.9799	1.9793	1.9435
25	1.9799	1.9793	1.9435
30	1.9799	1.9793	1.9435
40	1.9799	1.9792	1.9432
50	1.9799	1.9791	1.9431
60	1.9799	1.9791	1.9431
80	1.9798	1.9789	1.9429
100	1.9796	1.9781	1.9423
120	1.9794	1.9774	1.9419
150	1.9791	1.9765	1.9411
180	1.9786	1.9756	1.9402
210	1.9784	1.9753	1.9401
240	1.9774	1.9730	1.9387
270	1.9770	1.9721	1.9379

Supplementary Table 6. Extracted *g*-parameters for **4Rb** from the simulation of X-band powder spectra recorded at 9.4 GHz and 6-240 K.

Temperature (K)	g_x	g_y	g_z
6	1.9820	1.9770	1.9432
10	1.9820	1.9770	1.9432
15	1.9820	1.9770	1.9432
20	1.9820	1.9770	1.9432
25	1.9820	1.9770	1.9432
30	1.9820	1.9770	1.9432
40	1.9820	1.9770	1.9431
50	1.9820	1.9770	1.9430
60	1.9820	1.9770	1.9428
80	1.9820	1.9760	1.9426
100	1.9820	1.9750	1.9424
120	1.9820	1.9738	1.9414
150	1.9810	1.9733	1.9408
180	1.9800	1.9728	1.9400
210	1.9800	1.9715	1.9393
240	1.9790	1.9708	1.9384

Supplementary Table 7. Extracted *g*-parameters for **4Cs** from the simulation of X-band powder spectra recorded at 9.4 GHz and 5-293 K.

Temperature (K)	g_x	g_y	g_z
5	1.9802	1.9790	1.9435
10	1.9802	1.9790	1.9435
15	1.9802	1.9790	1.9435
20	1.9802	1.9790	1.9434
25	1.9802	1.9790	1.9434
30	1.9802	1.9790	1.9434
35	1.9802	1.9791	1.9434
40	1.9802	1.9791	1.9434
45	1.9802	1.9791	1.9434
50	1.9802	1.9791	1.9434
60	1.9802	1.9791	1.9434
80	1.9801	1.9788	1.9430
90	1.9799	1.9786	1.9428
100	1.9796	1.9782	1.9428
120	1.9791	1.9778	1.9422
140	1.9787	1.9776	1.9421
160	1.9780	1.9770	1.9410
180	1.9775	1.9764	1.9406
200	1.9772	1.9756	1.9401
220	1.9769	1.9748	1.9393
240	1.9765	1.9742	1.9388
260	1.9762	1.9730	1.9383
280	1.9761	1.9720	1.9378
293	1.9761	1.9715	1.9372

Supplementary Table 8. Sample information for those used in SQUID magnetometry measurements.

Entry	1	4K	4Rb	4Cs	4Cs-eico	5
Chemical formula	$C_{16}H_{24}Cl_2O_2Th$	$C_{42}H_{60}Cl_6KN_2O_6Th_3$	$C_{42}H_{60}Cl_6RbN_2O_6Th_3$	$C_{42}H_{60}Cl_6CsN_2O_6Th_3$	$C_{42}H_{60}Cl_6CsN_2O_6Th_3$	$C_{36}H_{54}Cl_6Cs_2O_7Th_3$
Molecular mass (g mol^{-1})	551.3	1636.77	1683.14	1730.58	1730.58	1773.36
Sample mass (mg)	27.6	20.4	21.0	30.0	28.5	24.3
Eicosane mass (mg)	13.0	9.4	10.0	0	14	15.0

Supplementary Table 9. Selected data for the magnetic properties of **4M**.

Entry	4K	4Rb	4Cs	4Cs-eico
χ_D (300 K, whole molecule)[a]	−1800E-06	−1560E-06	−1400E-06	−1480E-06
R^2 value	0.9999	0.9999	0.9997	0.9999
χ_D (300 K, 1/3 molecule)	–600E-06	–520E-06	–470E-06	–493E-06
χ_D (300 K, per Th)	−392.433E-06	−309.900E-06	−252.400E-06	−279.067E-06
Th Pascal's constant	−230.000E-06	−230.000E-06	−230.000E-06	−230.000E-06
Factor increase vs expected	17.06	13.47	10.97	12.13
Expected χ_D (whole molecule)	−691.700E-06	−699.300E-06	−711.800E-06	−711.800E-06
χ_D per 1/3 molecule	−230.567E-06	−233.100E-06	−237.267E-06	−237.267E-06
χ_D $C_{42}H_{60}Cl_6MN_2O_6$	−622.700E-06	−630.300E-06	−642.800E-06	−642.800E-06
χ_D per 1/3 of $C_{42}H_{60}Cl_6MN_2O_6$	−207.567E-06	−210.100E-06	−214.267E-06	−214.267E-06
Superatom contribution (%)	61.57	55.17	49.16	51.90
χ_D Increase vs expected (%)	160.23	123.08	96.69	107.92
Λ (χ_D(expected) − χ_D(Found)	1108.3E-06	860.7E-06	688.2E-06	768.2E-06

[a] χ is given in units of cm^3 mol^{-1}.

Supplementary Table 10. Mulliken population analyses of the HOMO of **4'** (SOMO) and selected NTOs with strong oscillator strengths in terms of the 6d, 5f and 7p-character (%) of MOs and Th metal contributions.

	HOMO	NTO1	NTO2	NTO3	NTO4	NTO8	NTO9	NTO10	NTO16	NTO17	NTO23	NTO26	NTO27
6d	62	26	26	1	3	3	20	19	11	5	5	62	26
5f	14	57	56	89	73	73	71	72	77	27	27	14	57
7p	7	0	0	1	1	1	0	0	1	1	1	7	0
7s	3	1	1	0	2	2	3	2	5	48	48	3	1
Th	86	84	84	91	78	79	94	94	94	81	81	86	84

Supplementary Table 11. Ring-current strengths for **4'** (nA/T).

Parameter	4'	$[Th_3]^{11+}$	$[Cl_6]^{6-}$	$[Th_3Cl_6]^{5+}$	$[COT_3]^{6-}$
Diatropic	57.4	0.23	79.3	54.2	0.022
Paratropic	−55.6	−1.07	−77.9	−53.7	−0.027
Total integral	1.81	−0.84	1.35	0.51	−0.0052

Supplementary Table 12. Ring-current integration data for **6** (nA/T).

Parameter	5	$[Th_3]^{12+}$	$[Cl_6]^{6-}$	$[Th_3Cl_6]^{6+}$	$[COT_3]^{6-}$
Diatropic	53.9	0.11	79.3	57.9	0.022
Paratropic	–54.0	–0.05	–77.9	–58.0	–0.027
Total integral	–0.11	0.06	1.35	–0.06	–0.0052

Supplementary Table 13. Final coordinates and energy of **4'** from a single point energy calculation on geometry optimized coordinates of **4'** optimized with ORCA 5.

Th	1.18460781505047	-2.04190154698053	-0.00012576903648
Cl	-1.04534484799735	-1.80301735565832	-1.72770095512831
C	1.58008924622013	-4.13584195942551	-1.69405636505589
H	1.15503992060008	-4.22304578828621	-2.68734921874959
Th	1.18546147793605	2.04219983643933	0.00020889617213
Cl	2.09183069401667	0.00016826692892	-1.73859803984577
C	0.72383732690658	-4.63507512377013	-0.70246059744952
H	-0.20514167779254	-5.01286156789401	-1.11348884643719
Cl	2.09189036520552	-0.00008008361978	1.73840354949505
C	0.72397556785097	-4.63480275689894	0.70330834964748
H	-0.20493163232364	-5.01238312638190	1.11468280174584
C	1.58041059820484	-4.13513290843289	1.69451495685151
H	1.15554750309295	-4.22188404583105	2.68792781106023
C	2.79886520764839	-3.43251068137398	1.69419603710587
H	3.08666917935293	-3.10760754176936	2.68741581972532
C	3.65936462320919	-2.94060898950484	0.70268849984185
H	4.45238176986369	-2.32668240395680	1.11372216154381
C	3.65923225884831	-2.94089205330968	-0.70312727013237
H	4.45216090820871	-2.32713866138782	-1.11458774693859
C	2.79851806911989	-3.43316907014514	-1.69426488832699
H	3.08608703955586	-3.10865526117531	-2.68768225362573
C	1.58074130655978	4.13555867695887	1.69467106075305
H	1.15583661610041	4.22235684112127	2.68806527112029
C	3.65984943549993	2.94123560884774	-0.70276697631451
H	4.45284902930222	2.32750596272826	-1.11410970664596
C	3.65990891919105	2.94110083712230	0.70306942982200
H	4.45294472310782	2.32727261081794	1.11420377994524
C	2.79929264241109	3.43305578791565	1.69448822715645
H	3.08707870939404	3.10829332794634	2.68776225382475
C	2.79915916875285	3.43338083539079	-1.69401374303723
H	3.08681523029933	3.10881298598145	-2.68737755687428
C	0.72426569253091	4.63497312725594	0.70333549463286
H	-0.20471710443165	5.01248377292788	1.11461068481594
C	0.72423003785294	4.63515293423371	-0.70244703419598
H	-0.20477011411332	5.01277499792836	-1.11357130311345
C	1.58061646295672	4.13589340722891	-1.69393164734170
H	1.15564923786717	4.22296984570498	-2.68726377614972
Th	-2.36659365159264	-0.00035230364384	-0.00103215079620
Cl	-1.04516119349942	-1.80266112833851	1.72676624595200
C	-4.37855972789983	-0.70334618733039	1.69475653320468
H	-4.24088020879582	-1.11541413610784	2.68779047720094
Cl	-1.04499168903247	1.80232457692392	1.72674428848213
C	-4.38458627724373	-1.69426025662717	0.70288087407541
H	-4.24918475167792	-2.68790617563263	1.11388871061265
Cl	-1.04488578440603	1.80266942922291	-1.72746019213030
C	-4.38497988386997	-1.69436611231504	-0.70303345307035
H	-4.24983419843686	-2.68808862369951	-1.11392453772063
C	-4.37952963433836	-0.70358939357651	-1.69507113996761
H	-4.24241805519935	-1.11582800173739	-2.68809948303676
C	-4.37945739029895	0.70288437907163	-1.69517218896380

H	-4.24226646510835	1.11493987243564	-2.68826731142770
C	-4.38479833141758	1.69379740190815	-0.70326676293160
H	-4.24955191585747	2.68744528008654	-1.11431305742089
C	-4.38441035235265	1.69388962957858	0.70264674395973
H	-4.24895931471481	2.68758702392456	1.11351948364968
C	-4.37848602592298	0.70310205090073	1.69465387849281
H	-4.24076655439384	1.11527393727969	2.68764165097540

Energy: −86896.675871916028 a.u.

Supplementary Table 14. Final coordinates and energy of **4'** from a single point energy calculation on geometry optimized coordinates of **4'** optimized with Gaussian 16 (for Raman and IR calculations).

Th	2.36357985	-0.16255549	-0.00016789
Cl	0.91409273	-1.86922905	-1.73055633
C	4.31769208	-1.00284433	-1.69405948
H	4.15730016	-1.40586875	-2.68968170
Th	-1.04089769	2.12755062	0.00011288
Cl	1.16134358	1.72557059	-1.73022726
C	4.25409477	-1.99117789	-0.70223762
H	4.05473716	-2.97601268	-1.11387706
Cl	1.16115254	1.72486266	1.73049715
C	4.25397592	-1.99070887	0.70335082
H	4.05453138	-2.97526818	1.11560776
C	4.31729070	-1.00173305	1.69454681
H	4.15670214	-1.40418355	2.69037269
C	4.41423638	0.40108321	1.69415125
H	4.31054002	0.82243705	2.68971322
C	4.48768458	1.38876484	0.70234608
H	4.42528348	2.39166554	1.11392047
C	4.48794265	1.38831660	-0.70324696
H	4.42564952	2.39093457	-1.11551187
C	4.41482814	0.39997948	-1.69441611
H	4.31112445	0.82075063	-2.69022894
C	-2.55314990	3.62246346	1.69486426
H	-2.86584371	3.32225348	2.69062040
C	-0.40216331	4.67884674	-0.70266390
H	0.55031302	4.99826269	-1.11478503
C	-0.40183912	4.67871803	0.70292572
H	0.55083676	4.99804503	1.11465961
C	-1.28963621	4.23953833	1.69460834
H	-0.86034237	4.30176109	2.69022826
C	-1.29041913	4.23982806	-1.69400041
H	-0.86162981	4.30223782	-2.68982563
C	-3.44574139	3.19224004	0.70354005
H	-4.28302404	2.63703944	1.11562520
C	-3.44607012	3.19236800	-0.70204982
H	-4.28356464	2.63728956	-1.11385438
C	-2.55399998	3.62288356	-1.69372036
H	-2.86708545	3.32265593	-2.68934986
Th	-1.32306504	-1.96512649	-0.00014168
Cl	0.91408231	-1.86916891	1.73036131
C	-1.86015591	-4.02253002	1.69429201
H	-1.44381118	-4.14303281	2.69006805
Cl	-2.07592933	0.14415411	1.73089024
C	-1.04084573	-4.57982150	0.70297892
H	-0.14120792	-5.02696615	1.11505469
Cl	-2.07598957	0.14437213	-1.73084174
C	-1.04069112	-4.58001985	-0.70260915
H	-0.14094466	-5.02726559	-1.11434620
C	-1.85968537	-4.02300617	-1.69435692

H	-1.44297740	-4.14381273	-2.68994497
C	-3.02626567	-3.23792833	-1.69470189
H	-3.29474952	-2.89778894	-2.69052087
C	-3.85107336	-2.68880752	-0.70340050
H	-4.60409706	-2.02381005	-1.11551598
C	-3.85121070	-2.68860715	0.70219847
H	-4.60437135	-2.02354652	1.11395090
C	-3.02682295	-3.23760239	1.69394322
H	-3.29555057	-2.89695962	2.68952954

Energy: –4912.37524714 a.u.

Supplementary Table 15. Final coordinates and energy of [Cs(2.2.2-cryptand)]$^+$ from a single point energy calculation on geometry optimized coordinates taken from **4Cs** optimized with ORCA 5.

Cs	-0.02016768169141	0.01748701667312	0.04460587936179
H	0.52748691952449	3.39268614635177	3.29132451873138
C	-0.18650595410258	1.40036008675602	3.50277975702998
H	-1.23797936943147	1.66574560004796	3.38055057664638
H	0.03977252409543	1.50937452077871	4.57660111453694
C	-1.22253645853900	-0.70806500054734	-3.34687993954219
H	-1.33665218080324	-0.69890543074055	-4.44612656427919
H	0.81669242419615	4.39356812513717	-1.26823882275905
H	-1.10853972521437	-1.75462459138471	-3.06135677232458
C	-2.54297464264663	-0.20884763944717	-2.77130832664606
H	-3.34118329275682	-0.81146931734533	-3.21990321870990
H	-2.73297444209879	0.82492317435203	-3.06382103563452
O	-2.64506043336874	-0.23772477420142	-1.36114049117927
C	-3.17432005110542	-1.42187031367492	-0.79969546640498
H	-4.11317440704516	-1.68384997536252	-1.30241554398901
H	-2.48247788912886	-2.26674855149155	-0.91671768616784
C	-3.46475040335213	-1.20017815183608	0.66565998475401
H	-4.13964682200239	-1.98908873460413	1.02009782502322
H	-3.97619079792032	-0.23648534151280	0.78109698460813
O	-2.26906108634241	-1.21638270120421	1.41940768684357
C	-2.41179070478009	-0.68150321703854	2.71928494658830
H	-2.73234278335936	0.36691711926493	2.65392307962039
H	-0.59383594706238	3.38752365329307	-0.88689279292326
C	0.85185195055686	3.56070078105279	0.68722638250795
H	-3.19492894118374	-1.22381335502260	3.26433821274554
H	3.70416551466506	-2.65879900492767	1.03377763473409
C	-1.12103794098927	-0.82376219353861	3.50358253237498
H	-0.81268664198043	-1.86751179940960	3.42837185685367
H	-1.35729636213235	-0.64509455691519	4.56568937731748
N	-0.00943658010333	0.01765236775522	3.06985953380010
H	0.58632342386456	4.56093440884188	1.05238454646814
H	2.07869090730561	-3.33565452939670	0.80033659939554
O	2.14669976691163	-1.36548804454505	1.42737597236446
C	1.78065407293470	-1.72815118901986	2.74241242968601
C	2.69060084150796	-2.43301235675130	0.67920979997907
H	1.03197540945348	-2.53070066271361	2.70823576389105
H	2.65204641786984	-2.12402642162532	3.27969517842410
H	1.93604639515989	3.43401144987382	0.79979963663657
O	0.16247153108388	2.57783640311083	1.43088533161443
C	0.62815348542809	2.43951048389262	2.75632211969789
H	1.69666044961462	2.18732789900495	2.74752992420383
C	1.19889217025763	-0.67777615594953	-3.33405164485819
H	1.26809115934471	-0.77012369255770	-4.43322239049767
H	2.04607812313736	-0.06338912540113	-3.02607048448050
C	1.40814600202993	-2.07722484145781	-2.76731188886031
H	2.33431656576751	-2.46845921165187	-3.20351114593662
H	0.61022769314364	-2.75255078082006	-3.07966659974878

C	1.27313544691336	-0.52219735660341	3.50904058918043
H	2.02729911382394	0.26030508192418	3.41442705247917
H	1.24126189866552	-0.79597143931631	4.57674486941350
O	1.46139438740265	-2.16051573122764	-1.35605449920228
N	-0.02246959817365	0.00447947053504	-2.93104258637826
C	-0.04041929474872	1.40670140895478	-3.32450094905287
H	-0.00990394957872	1.51692504012149	-4.42387765668819
H	-0.99060751879440	1.83517183680757	-3.00126895235144
C	1.07986556823294	2.28277098955403	-2.77473810001939
H	0.94669108820851	3.28261481442194	-3.20271248119613
H	2.05555978972672	1.92980263125869	-3.11240399970563
C	2.75348670685356	-2.07898058431131	-0.78680886719155
O	1.15558689371499	2.36237830732722	-1.36359950062387
H	3.42310630670970	-2.79123189611395	-1.28367713588537
C	0.49591952914784	3.46726635238901	-0.77678180222531
H	3.18160139318343	-1.07479651981332	-0.90678034204989

Energy: –9183.730896268396 a.u

Supplementary Table 16. Experimental geometry of **4Cs**

Th	0.28058200	-2.31601433	0.00000000
Th	1.86543600	1.40099767	0.00000000
Th	-2.14601800	0.91501667	0.00000000
C	-3.48319700	2.57633567	1.70544800
C	4.47037100	1.36694067	-0.82627900
C	-3.41899100	3.18798367	-0.82627900
H	-1.83830900	-4.72910533	-1.31337600
H	-0.98669200	-4.30581733	2.50797200
H	5.01468000	0.77253067	-1.31337600
H	4.22229300	1.29840867	2.50797200
H	-3.17637100	3.95657567	-1.31337600
C	-0.48957400	-4.30470533	1.70544800
Cl	-1.71992900	-1.27843733	-1.78174600
H	-0.15465700	-4.50029333	-2.60265000
C	-1.30017900	-4.45761733	0.56096900
C	4.51050000	1.10282067	0.56096900
C	-3.21032000	3.35479667	0.56096900
H	-3.23560100	3.00740867	2.50797200
H	-5.19044500	-0.67068133	-0.50790500
C	0.89888900	-4.15179333	1.91873800
C	3.14611400	2.85435767	1.91873800
C	-4.04500300	1.29743567	1.91873800
H	1.10956700	-4.07325133	2.84091100
H	2.97275600	2.99753867	2.84091100
H	-2.23053000	-4.51794833	0.76870900
H	-4.08232300	1.07571267	2.84091100
H	2.72957500	3.84328167	-2.25323700
H	5.02792300	0.32727767	0.76870900
H	-4.69316700	0.44224067	-2.25323700
H	-2.79739300	4.19066967	0.76870900
C	2.04015800	-4.09618833	1.09852800
C	0.06420700	-4.45761733	-1.67596900
C	2.52732400	3.81492367	1.09852800
C	-4.56748300	0.28126567	1.09852800
H	2.84514600	-3.95884633	1.58736000
Cl	1.96712400	-0.85028333	-1.78174600
H	2.00588800	4.44339167	1.58736000
H	-4.85103400	-0.48454533	1.58736000
C	3.82830700	2.28441267	-1.67596900
H	3.97469700	2.11620967	-2.60265000
C	2.25525000	-4.17959533	-0.28872000
C	2.49201100	4.04290167	-0.28872000

C	-4.74726100	0.13669367	-0.28872000
H	2.01439600	4.83039767	-0.50790500
H	3.17605000	-4.15971633	-0.50790500
C	-3.89251300	2.17320367	-1.67596900
C	-1.05138000	-4.55492533	-0.82627900
C	3.97277100	1.72836967	1.70544800
Cl	-1.69745700	-1.30345933	1.70371500
C	1.45267000	-4.30470533	-1.45054100
C	3.00164900	3.41040167	-1.45054100
C	-4.45431900	0.89430367	-1.45054100
H	-3.82004000	2.38408367	-2.60265000
Cl	1.97755700	-0.81831133	1.70371500
Cl	-0.28010000	2.12176967	1.70371500
Cl	-0.24719500	2.12872067	-1.78174600
H	1.96359200	-4.28552133	-2.25323700
Cs	0.00000000	0.00000067	-8.63301700
C	0.66614100	-2.41415633	-5.87239300
C	-3.45109400	1.18622667	-7.98794600
C	1.75765000	1.78397367	-5.87239300
H	-1.04568100	-2.56831933	-11.49144100
H	2.74707100	0.37857267	-11.49144100
H	-1.70138900	2.18974667	-11.49144100
C	2.23919800	1.22792967	-11.45606600
H	0.28363200	-3.38306233	-11.88611300
H	2.78800200	1.93716367	-11.88611300
C	1.22794700	0.58847967	-5.04004500
H	-2.62893100	-0.32510033	-6.01285200
H	-3.07163400	1.44589867	-11.88611300
H	1.03292000	2.43927067	-6.01285200
C	-1.12361200	0.76919367	-5.04004500
H	-1.06269600	-1.61553833	-5.02166400
H	-0.98476500	-3.68332633	-9.25883900
C	0.00802600	-3.66525533	-9.37519400
C	-0.10433500	-1.35767333	-5.04004500
H	0.67047500	-4.46262133	-7.54662800
C	3.17019100	1.83957767	-9.37519400
H	1.06630800	0.98799667	-13.15024300
H	-3.17781600	1.04540867	-5.38633700
H	3.52950600	2.81195967	-7.54662800
H	1.47923500	-1.29915033	-12.06246700
C	0.88444300	1.07779867	-12.18437100
O	0.39326400	-2.46976133	-10.09136100
C	2.75284900	2.39562167	-7.98794600
C	-2.18301700	1.32523767	-11.45606600
H	1.59601000	-2.11417033	-6.01285200
O	-2.33550800	0.89430367	-10.09136100
H	-4.19998100	1.65066167	-7.54662800
O	0.01605200	-2.59487133	-7.16080000
O	1.94224400	1.57545767	-10.09136100
H	1.93044500	-0.11255333	-5.02166400
H	3.68223700	0.98883167	-9.25883900
C	0.69824400	-3.58184833	-7.98794600
H	-2.69747200	2.69449467	-9.25883900
C	-3.17821700	1.82567667	-9.37519400
O	2.23919800	1.31133667	-7.16080000
C	-1.37562200	0.22705067	-12.18437100
H	0.28459500	-4.47485433	-9.85102000
C	-2.42379100	0.63018267	-5.87239300
H	-0.86774900	1.72809067	-5.02166400
H	0.23202600	-1.38505833	-4.11492300
C	-0.05618100	-2.55316733	-11.45606600
H	1.08348300	0.89346967	-4.11492300

H	-1.31550900	0.49158867	-4.11492300
O	-2.25525000	1.28353467	-7.16080000
H	0.38547900	1.93062967	-12.06246700
H	0.68355800	-3.27477333	-5.38633700
H	3.73304000	2.48389367	-9.85102000
H	-1.86471400	-0.63148033	-12.06246700
H	2.04978900	3.07927767	-8.10551600
N	0.00000000	0.00000067	-11.80287800
H	1.64183800	-3.31480833	-8.10551600
H	-4.01763500	1.99096067	-9.85102000
H	-1.38878400	0.42945167	-13.15024300
N	0.00000000	0.00000067	-5.54292200
H	2.49425800	2.22936467	-5.38633700
H	0.32247600	-1.41744833	-13.15024300
H	-3.69162700	0.23553067	-8.10551600
C	0.49117900	-1.30484933	-12.18437100

Energy: −25.340472974734329 a.u.

Supplementary References

1. Cantat, T., Scott, B. L. & Kiplinger, J. L. Convenient access to the anhydrous thorium tetrachloride complexes $ThCl_4(DME)_2$, $ThCl_4(1,4\text{-dioxane})_2$ and $ThCl_4(THF)_{3.5}$ using commercially available and inexpensive starting materials. *Chem. Commun.* **46**, 919-921 (2010).
2. Bergbreiter, D. E. & Killough, J. M. Reactions of potassium-graphite. *J. Am. Chem. Soc.* **100**, 2126-2134 (1978).
3. Zhou, Z., Greenough, J., Wei, Z. & Petrukhina, M. A. The dinuclear scandium(III) cyclooctatetraenyl chloride complex di-μ-chlorido-bis[(η^8-cyclooctatetraene)(tetrahydrofuran-κ*O*)scandium(III)]. *Acta Cryst. C* **73**, 420-423 (2017).
4. Avdeef, A., Raymond, K. N., Hodgson, K. O. & Zalkin, A. Two isostructural actinide π complexes. Crystal and molecular structure of bis(cyclooctatetraenyl)uranium(IV), $U(C_8H_8)_2$, and bis(cyclooctatetraenyl)thorium(IV), $Th(C_8H_8)_2$. *Inorg. Chem.* **11**, 1083-1088 (1972).
5. Le Vanda, C., Solar, J. P. & Streitwieser, A. Half-sandwich cyclooctatetraenethorium compounds. *J. Am. Chem. Soc.* **102**, 2128-2136 (1980).
6. Sheldrick, G. M. SHELXT – Integrated space-group and crystal-structure determination. *Acta Cryst. Sect. A* **71**, 3-8 (2015).
7. CrysAlisPRO version 40.69, Oxford Diffraction/Agilent Technologies UK Ltd, Yarnton, England.
8. Sheldrick, G. M. Crystal structure refinement with SHELXL. *Acta Cryst. Sect. C* **71**, 3-8 (2015).
9. Dolomanov, O. V., Bourhis, L. J., Gildea, R. J., Howard, J. A. K. & Puschmann, H. OLEX2: a complete structure solution, refinement and analysis program. *J. Appl. Cryst.* **42**, 339-341 (2009).
10. Farrugia, L. J. WinGX and ORTEP for Windows: an update. *J. Appl. Cryst.* **45**, 849-854 (2012).
11. Persistence of Vision (TM) Raytracer, Persistence of Vision Pty. Ltd., Williamstown, Victoria, Australia.
12. Kabova, E. A., Blundell, C. D., Muryn, C. A., Whitehead, G. F. S., Vitorica-Yrezabal, I. J., Ross, M. J. & Shankland, K. SDPD-SX: combining a single crystal X-ray diffraction setup with advanced powder data structure determination for use in early stage drug discovery. *CrystEngComm* **24**, 4337-4340 (2022).
13. Petříček, V., Dušek, M. & Palatinus, L. Crystallographic Computing System JANA2006: General features. *Zeitschrift für Krist. - Cryst. Mater.* **229**, 345-352 (2014).
14. Coelho, A. A. An Indexing Algorithm Independent of Peak Position Extraction for X-Ray Powder Diffraction Patterns. *J. Appl. Crystallogr.* **50**, 1323-1330 (2017).

15. Macrae, C. F., Sovago, I., Cottrell, S. J., Galek, P. T. A., McCabe, P., Pidcock, E., Platings, M., Shields, G. P., Stevens, J. S., Towler, M. & Wood, P. A. Mercury 4.0: from visualization to analysis, design and prediction. *J. Appl. Crystallogr.* **53**, 226-235 (2020).
16. Stoll, S. & Schweiger, A. Easyspin, a comprehensive software package for spectral simulation and analysis in EPR. *J. Magn. Reson.* **178**, 42-55 (2006).
17. Bain, G. A. & Berry, J. F. Diamagnetic corrections and Pascal's constants. *J. Chem. Ed.* **85**, 532-536 (2008).
18. Hager, J. S., Zahardis, J., Pagni, R. M., Compton, R. N. & Li, J. Raman under nitrogen. The high-resolution Raman spectroscopy of crystalline uranocene, thorocene, and ferrocene. *J. Chem. Phys.* **120**, 2708-2718 (2004).
19. Clark, D. L., Grumbine, S. K., Scott, B. L. & Watkin, J. G. Unique molecular structure of the actinide hydrido aryloxide complex $Th_3(\mu_3\text{-}H)_2(\mu_2\text{-}H)_4(O\text{-}2,6\text{-}t\text{-}Bu_2C_6H_3)_6$. *J. Am. Chem. Soc.* **117**, 9089-9090 (1995).
20. Fulmer, G. R., Miller, A. J. M., Sherden, N. H., Gottlieb, H. E., Nudelman, A., Stoltz, B. M., Bercaw, J. E. & Goldberg, K. I. NMR Chemical Shifts of Trace Impurities: Common Laboratory Solvents, Organics, and Gases in Deuterated Solvents Relevant to the Organometallic Chemist. *Organometallics*. **29**, 2176-2179 (2010).
21. Merill, P. B. & Madix, R. J. Aromatization of 1,3,5,7-cyclooctatetraene and oxydehydrogenation of cis-cyclooctene, 1,5-cyclooctadiene and 1,3,5,7-cyclooctatetraene on clean and oxygen precovered Ag(ll0). *Surface Science* **365**, 701-728 (1996).
22. Jolivet, J. P., Thomas, Y., Taravel, B., Lorenzelli, V. & Busca, G. Infrared spectra of cerium and thorium pentacarbonate complexes. *J. Mol. Struc.* **79**, 403-408 (1982).
23. Karyakin, A. V. & Volynets, M. P. Infrared spectra of thorium-carbonate complex salts. *J. Struct. Chem.* **3**, 689-690 (1962).
24. Clavier, N., Hingant, N., Rivenet, M., Obbade, S., Dacheux, N., Barré, N. & Abraham, F. X-ray diffraction and μ-Raman investigation of the monoclinic-orthorhombic phase transition in $Th_{1-x}U_x(C_2O_4)_2{\cdot}2H_2O$ solid solutions. *Inorg. Chem.* **49**, 1921-1931 (2010).
25. Formanuik, A., Ortu, F., Inman, C. J., Kerridge, A., Castro, L., Maron, L. & Mills, D. P. Concomitant Carboxylate and Oxalate Formation From the Activation of CO_2 by a Thorium(III) Complex. *Chem. Eur. J.* **22**, 17976-17979 (2016).
26. Neese, F., Wennmohs, F., Becker, U. & Riplinger, C. The ORCA quantum chemistry program package. *J. Chem. Phys.* **152**, 224108 (2020).
27. Van Lenthe, E., Baerends, E. J. & Snijders, J. G. Relativistic regular two-component Hamiltonians. *J. Chem. Phys.* **99**, 4597-4610 (1993).
28. Adamo, C. & Barone, V. Toward reliable density functional methods without adjustable parameters: The PBE0 model. *J. Chem. Phys*. **110**, 6158-6170 (1999).
29. Grimme, S., Antony, J., Ehrlich, S. & Krieg, H. A consistent and accurate ab initio parametrization of density functional dispersion correction (DFT-D) for the 94 elements H-Pu. *J. Phys. Chem.* **132,** 154104 (2010).
30. Grimme, S., Ehrlich, S. & Goerigk, L. Effect of the damping function in dispersion corrected density functional theory. *J. Comp. Chem.* **32,** 1456-1465 (2011).
31. Becke, A. D. & Johnson, E. R. A density-functional model of the dispersion interaction. *J. Chem. Phys.* **123**, 154101 (2005).
32. Pantazis, D. A. & Neese, F. All-Electron Scalar Relativistic Basis Sets for the Actinides. *J. Chem. Theory Comput.* **7**, 677-684 (2011).
33. Weigend, F. & Ahlrichs, R. Balanced basis sets of split valence, triple zeta valence and quadruple zeta valence quality for H to Rn: Design and assessment of accuracy. *Phys. Chem. Chem. Phys.* **7**, 3297-305 (2005).
34. Weigend, F. Accurate Coulomb-fitting basis sets for H to Rn. *Phys. Chem. Chem. Phys*. **8**, 1057-1065 (2006).

35. Neese, F. An improvement of the resolution of the identity approximation for the formation of the Coulomb matrix. *J. Comp. Chem.* **24**, 1740-1747 (2003).
36. Riplinger, C. & Neese, F. An efficient and near linear scaling pair natural orbital based local coupled cluster method. *J. Chem. Phys*. **138**, 034106 (2013).
37. Saitow, M., Becker, U., Riplinger, C., Valeev, E. F. & Neese, F. A new near-linear scaling, efficient and accurate, open-shell domain-based local pair natural orbital coupled cluster singles and doubles theory. *J. Chem. Phys*. **146**, 164105 (2017).
38. Douglas, M. & Kroll, N. M. Quantum electrodynamical corrections to the fine structure of helium. *Ann. Phys*. **82**, 89-155 (1974).
39. Hess, A. Relativistic electronic-structure calculations employing a two-component no-pair formalism with external-field projection operators. *Phys. Rev. A Gen. Phys*. **33**, 3742-3748 (1986).
40. Stoychev, G. L., Auer, A. A. & Neese, F. Automatic Generation of Auxiliary Basis Sets. *J. Chem. Theory Comput.* **13**, 554-562 (2017).
41. Gaussian 16, Revision C.01, Frisch, M. J., Trucks, G. W., Schlegel, H. B., Scuseria, G. E., Robb, M. A., Cheeseman, J. R., Scalmani, G., Barone, V., Petersson, G. A., Nakatsuji, H., Li, X., Caricato, M., Marenich, A. V., Bloino, J., Janesko, B. G., Gomperts, R., Mennucci, B., Hratchian, H. P., Ortiz, J. V., Izmaylov, A. F., Sonnenberg, J. L., Williams-Young, D., Ding, F., Lipparini, F., Egidi, F., Goings, J., Peng, B., Petrone, A., Henderson, T., Ranasinghe, D., Zakrzewski, V. G., Gao, J., Rega, N., Zheng, G., Liang, W., Hada, M., Ehara, M., Toyota, K., Fukuda, R., Hasegawa, J., Ishida, M., Nakajima, T., Honda, Y., Kitao, O., Nakai, H., Vreven, T., Throssell, K., Montgomery, Jr., J. A., Peralta, J. E., Ogliaro, F., Bearpark, M. J., Heyd, J. J., Brothers, E. N., Kudin, K. N., Staroverov, V. N., Keith, T. A., Kobayashi, R., Normand, J., Raghavachari, K., Rendell, A. P., Burant, J. C., Iyengar, S. S., Tomasi, J., Cossi, M., Millam, J. M., Klene, M., Adamo, C., Cammi, R., Ochterski, J. W., Martin, R. L., Morokuma, K., Farkas, O., Foresman, J. B., Fox, D. J. Gaussian, Inc., Wallingford CT, 2016.
42. Boronski, J. T., Seed, J. A., Hunger, D., Woodward, A. W., Van Slageren, J., Wooles, A. J., Natrajan, L. S., Kaltsoyannis, N. & Liddle, S. T. A crystalline tri-thorium cluster with σ-aromatic metal-metal bonding. *Nature* **598**, 72-75 (2021).
43. Cao, X. & Dolg, M. Segmented contraction scheme for small-core actinide pseudopotential basis sets. *J. Mol. Struct. THEOCHEM* **673**, 203-209 (2004).
44. Cao, X., Dolg, M. & Stoll, H. Valence basis sets for relativistic energy-consistent small-core actinide pseudopotentials. *J. Chem. Phys*. **118**, 487-496 (2003).
45. Küchle, W., Dolg, M., Stoll, H. & Preuss, H. Energy-adjusted pseudopotentials for the actinides. Parameter sets and test calculations for thorium and thorium monoxide. *J. Chem. Phys*. **100**, 7535-7542 (1994).
46. Van Lenthe, E., Wormer, P. E. & Van der Avoird, A. D. Density functional calculations of molecular g-tensors in the zero-order regular approximation for relativistic effects. *J. Chem. Phys.* **107**, 2488-2498 (1997).
47. Te Velde, G., Bickelhaupt, F. M., Baerends, E. J., Fonseca Guerra, C., Van Gisbergen, S. J. A., Snijders, J. G. & Ziegler, T. Chemistry with ADF. *J. Comput. Chem.* **22**, 931-967 (2001).
48. Van Lenthe, E. & Baerends, E. J. Optimized Slater-type basis sets for the elements 1-118. *J. Comp. Chem.* **24**, 1142-1156 (2003).
49. Lu, T. & Chen, F. Multiwfn: a multifunctional wavefunction analyzer. *J. Comp. Chem.* **33**, 580-592 (2012).
50. Zubarev, D. Y. & Boldyrev, A. I. Developing paradigms of chemical bonding: adaptive natural density partitioning. *Phys. Chem. Chem. Phys*. **10**, 5207-5217 (2008).
51. Szczepanik, D. W. & Solà, M. The electron density of delocalized bonds (EDDBs) as a measure of local and global aromaticity. *Aromaticity, Modern Computational Methods and Applications*, 259-284 (Elsevier, 2021).

52. Tomeček, J., Liddle, S. T. & Kaltsoyannis, N. Actinide-Actinide Bonding: Electron Delocalisation and σ-Aromaticity in the Tri-Thorium Cluster [$\{Th(\eta^8-C_8H_8)(\mu-Cl)_2\}_3K_2$]. *ChemPhysChem* **24**, e202300366 (2023).
53. Glendening, E. D., Landis, C. R. & Weinhold, F. Natural bond orbital methods. *WIREs Comput. Mol. Sci.* **2**, 1-42 (2012).
54. Geuenich, D., Hess, K., Köhler, F. & Herges, R. Anisotropy of the Induced Current Density (ACID), a General Method To Quantify and Visualize Electronic Delocalization. *Chem. Rev.* **105**, 3758-3772 (2005).
55. Kloda, S. & Kleinpeter, E. *Ab initio* calculation of the anisotropy effect of multiple bonds and the ring current effect of arenes – application in conformational and configurational analysis, *J. Chem. Soc. Perkin Trans.* **2**, 1893-1898 (2001).
56. Juselius, J., Sundholm, D. & Gauss, J. Calculation of Current Densities Using Gauge-Including Atomic Orbitals. *J. Chem. Phys.* **121**, 3952-3963 (2004).
57. Fliegl, H., Taubert, S., Lehtonen, O. & Sundholm, D. The Gauge Including Magnetically Induced Current Method. *Phys. Chem. Chem. Phys.* **13**, 20500-20518 (2011).
58. Taubert, S., Sundholm, D. & Juselius, J. Calculation of Spin-Current Densities Using Gauge-Including Atomic Orbitals. *J. Chem. Phys.* **134**, 054123 (2011).
59. Cheeseman, J. R., Trucks, G. W., Keith, T. A. & Frisch, M. J. A comparison of models for calculating nuclear magnetic resonance shielding tensors. *Chem. Phys.* **104**, 5497-5509 (1996).
60. Ziegler, T. & Rauk, A. A theoretical study of the ethylene-metal bond in complexes between copper(1+), silver(1+), gold(1+), platinum(0) or platinum(2+) and ethylene, based on the Hartree-Fock-Slater transition-state method. *Inorg. Chem.* **18**, 1558-1565 (1979).
61. Ziegler, T. & Rauk, A. Carbon monoxide, carbon monosulfide, molecular nitrogen, phosphorus trifluoride, and methyl isocyanide as σ-donors and π-acceptors. A theoretical study by the Hartree-Fock-Slater transition-state method. *Inorg. Chem.* **18**, 1755-1759 (1979).
62. Bickelhaupt, F. M. & Baerends, E. J. Kohn-Sham Density Functional Theory: Predicting and Understanding Chemistry. *Rev. Comput. Chem.* 1-86 (2000).
63. Perdew, J. P., Burke, K. & Ernzerhof, M. Generalized Gradient Approximation Made Simple. *Phys. Rev. Lett.* **77**, 3865-3868 (1996).
64. Van Lenthe, E., Baerends, E. J. & Snijders, J. G. Relativistic total energy using regular approximations. *J. Chem. Phys.* **101**, 9783-9792 (1994).
65. Van Lenthe, E., Ehlers, A. & Baerends, E.-J. Geometry optimizations in the zero order regular approximation for relativistic effects. *J. Chem. Phys.* **110**, 8943-8953 (1999).
66. Rowland, R. S. & Taylor, R. Intermolecular Nonbonded Contact Distances in Organic Crystal Structures: Comparison with Distances Expected from van der Waals Radii. *J. Phys. Chem.* **100**, 7384-7391 (1996).
67. Lehn, J. M. Cryptate inclusion complexes, effects on solute-solute and solute-solvent interactions and on ionic reactivity. *Pure Appl. Chem.* **52**, 2303-2319 (1980).
68. Kauffmann, E., Dye, J. L., Lehn, J.-M. & Popov, A. I. A study of the inclusive and exclusive cesium cryptates in nonaqueous solvents by cesium-133 NMR. *J. Am. Chem. Soc.* **102**, 2274-2278 (1980).
69. Wipff, G. Molecular modelling studies on molecular recognition: Crown ethers, cryptands and cryptates. From static models in vacuo to dynamical models in solution. *J. Coord. Chem.* **27**, 7-37 (1992).
70. Leite, E.S., Santana, S.R. & Hünenberger, P.H. On the relative stabilities of the alkali cations 222 cryptates in the gas phase and in water-methanol solution. *J Mol Model*, **13**, 1017-1025 (2007).
71. Auffinger, P. & Wipff, G. Quantitative studies on molecular recognition: free energy perturbation simulations on $M^+ \subset 222$ cryptates in water and in methanol. *J. Chim. Phys.* **88**, 2525-2534 (1991).

72. Altman, A. B., Brown, A. C., Rao, G., Lohrey, T. D., Britt, R. D., Maron, L., Minasian, S. G., Shuh, D. K. & Arnold, J. Chemical structure and bonding in a thorium(III)–aluminum heterobimetallic complex. *Chem. Sci.* **9**, 4317-4324 (2018).
73. Nguyen, J. Q., Anderson-Sanchez, L. M., Moore, W. N. G., Ziller, J. W., Furche, F. & Evans, W. J. Replacing trimethylsilyl with triisopropylsilyl provides crystalline $(C_5H_4SiR_3)_3Th$ complexes of Th(III) and Th(II). *Organometallics* **42**, 2927-2937 (2023).
74. Parry, J. S., Cloke, G, Coles, S. J., Hursthouse, M. B. Synthesis and Characterization of the First Sandwich Complex of Trivalent Thorium: A Structural Comparison with the Uranium Analogue. *J. Am. Chem. Soc.* **121**, 6867-6871 (1999).
75. Van Oven, H. O. & de Liefde Meijer, H. J.Cyclopentadienylcyclooctatetraenetitanium. *J. Organomet. Chem.* **19**, 373-376 (1969).
76. Kool, L. B, Rausch, M. D. & Rogers, R. D. The formation, crystal and molecular structure of (η^5-pentamethylcyclopentadienyl)(η^7-cycloheptatrienyl)titanium and (η^5-pentamethylcyclopentadienyl)(η^8-cyclooctatetraene)titanium. *J. Organomet. Chem.* **297**, 289-299 (1985).
77. Blenkers, J., Bruin, P. & Teuben J.H. Group IV Mixed Sandwich Compounds. *J. Organomet. Chem.* **297**, 61-67 (1985).
78. Horáček, M., Kupfer, V., Thewalt, U., Polášek, M. & Mach, K. Synthesis and structures of paramagnetic binuclear (η^8-1,4-bis(trimethylsilyl)cyclooctatetraenide)titanium(III) chlorides, *J. Organomet. Chem.* **579**, 126–132 (1999).
79. MacKenzie, R. E., Hajdu, T., Seed, J. A., Whitehead, G. F. S., Adams, R. W., Chilton, N. F., Collison, D., McInnes, E. J. L. & Goodwin, C. A. P. δ-Bonding modulates the electronic structure of formally divalent nd^1 rare earth arene complexes. *Chem. Sci.*, **15**, 15160-15169 (2024).
